# Small Gold Nanoparticles Alleviate Huntington’s Disease via Modulating p38α Mitogen-Activated Protein Kinase and Pyruvate Dehydrogenase Kinase 1

**DOI:** 10.1021/acsnano.5c14751

**Published:** 2025-12-22

**Authors:** Leo Kit Cheung Lee, Lok I Leong, Moldir Shyngys, Qianqian Bai, Ying Lam Lui, Can Cui, Shaorui Liu, Yu Xiao, Cecilia Ka Wing Chan, Wing-Hoi Cheung, Kin Ming Kwan, Ho Yin Edwin Chan, Chung Hang Jonathan Choi

**Affiliations:** †Department of Biomedical Engineering, ‡School of Life Sciences, §Musculoskeletal Research Laboratory, Department of Orthopedics and Traumatology, ∥Department of Surgery, 26451 The Chinese University of Hong Kong, Shatin, New Territories, Hong Kong; ⊥ Center for Neuromusculoskeletal Restorative Medicine, Hong Kong Science Park, Shatin, New Territories, Hong Kong

**Keywords:** gold nanoparticles, nanomedicine, Huntington’s disease, neurodegenerative disease, oxidative phosphorylation, pyruvate dehydrogenase kinase 1, p38α

## Abstract

Huntington’s disease (HD) is a severe neurodegenerative disorder that causes motor impairment and ultimately death. Yet, safe and effective disease-modifying treatments of HD are scarce, and delivery to the HD brain is challenging. Here, we present a small, ∼11 nm polyethylene glycol-coated gold nanoparticle (NP) for brain delivery and treating HD in R6/2 mice. Upon intravenous injection, this NP crosses brain barriers, preferentially accumulates in the brain of R6/2 mice than healthy littermates without pronounced liver or kidney sequestration, diffuses to the cortex and striatum, and enters neurons and microglia. Devoid of known chemical or biological drugs, this NP improves motor deficit as effectively as tetrabenazine (standard therapy for symptomatic relief) and prolongs survival, without long-term brain retention or systemic toxicity. This NP activates the oxidative phosphorylation pathway, blocks p38α mitogen-activated protein kinase phosphorylation and pyruvate dehydrogenase kinase 1 activity, and inhibits pyroptosis. Our data offer molecular insights into gold nanomedicines against neurodegenerative diseases.

## Graphical abstract

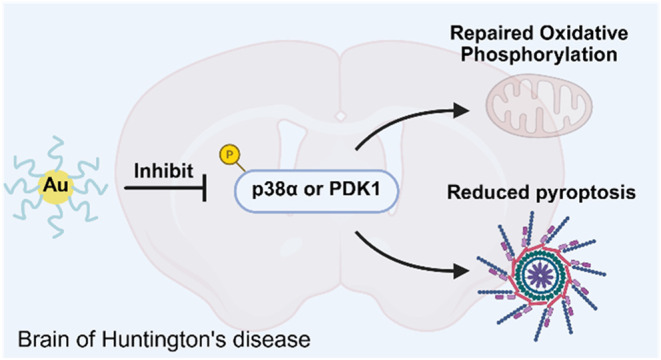

Huntington’s disease (HD) is a fatal neurodegenerative disorder marked by progressive neuronal loss in the striatum and cortex,^1^ causing impaired motor coordination. Emerging evidence also revealed the onset of neuroinflammation in the brain, marked by activated microglia.^2^ Currently, there is no approved disease-modifying treatment targeting the key pathway proteins of HD, and many drugs merely relieve symptoms (*e.g*., tetrabenazine for mitigating HD-linked chorea). Past disease-modifying drug candidates faced clinical setbacks due to their unfavorable benefit-risk profile [*e.g*., antisense oligonucleotides for inhibiting the mutant *huntingtin* (m*HTT*) gene].^3^ Exploration of alternative disease-modifying mechanisms^4^ and therapeutic approaches^5^ is critical.

Nanoparticles (NPs) are emerging drug carriers to the brain for alleviating HD,^6^ with only <30 animal-based studies published thus far (Figure S1 and Table S1). Typically, NPs promote the delivery of free drugs to the brain^7^ by preventing their clearance out of the body via kidney filtration^8^ and, when endowed with targeting ligands, by engaging specific receptors on the blood-brain barrier (BBB) for transcytosis.^9^ Yet, with carrier, drugs, and targeting ligands all included, the tricomponent NP becomes so large (mostly >100 nm; Table S1) that causes liver sequestration^8^ and impedes diffusion in the extracellular brain space.^10^

We present a small ∼11 nm poly­(ethylene glycol) (PEG)-coated gold NP for intravenous (i.v.) delivery to the brain and alleviating HD. The 3 nm gold core enables the dissection of bio–nano interactions in the HD brain and keeps the overall NP small for crossing brain barriers and reducing liver clearance. [We select gold NP over other metal-based NP types for reducing HD because of its more advanced use for alleviating other types of neurodegenerative disorders in preclinical and clinical settings (Figure S2 and Table S2).] The dense shell of 1000-Da PEG strands endows the gold core with stability in blood and cerebrospinal fluid (CSF) and promotes diffusion within the brain.^11^ Upon i.v. injection into R6/2 transgenic mice whose brain contains overexpressed exon 1 of human mHTT (an established model of HD^12^), the NP crosses the BBB and/or blood-CSF barrier, accumulates selectively in the HD brain over the healthy brain, and distributes diffusely to neurons and activated microglia in the cortex and striatum (key sites of HD pathogenesis). Brain delivery depends on NP size and disease stage. Repeated injections of the NP reduce motor deficit as effectively as tetrabenazine and prolong survival, without the aid of known chemical or biological drugs. This self-therapeutic NP^13^ inhibits p38α mitogen-activated protein kinase (MAPK) phosphorylation and pyruvate dehydrogenase kinase 1 (PDK1) activity in the HD brain, upregulates the oxidative phosphorylation pathway (downstream of PDK1), and suppresses pyroptosis-induced cell death (downstream of both kinases). Collectively, these mechanistic results move beyond past phenomenological description of the anti-inflammatory and antioxidative properties of gold NP (Figure S2 and Table S2). We prove NP clearance and limited systemic toxicity up to 3 months postinjection.

## Results and Discussion

### Size- and Disease Stage-Dependent Delivery to the HD Brain

We previously reported that, upon i.v. injection, <10 nm PEG-coated gold NPs entered kidney tubules and were cleared out of the body via urine^14^ whereas that >30 nm PEG-coated gold NPs accumulated inside liver macrophages.^15^ To prevent NP clearance by the kidney and liver, we reasoned that an optimal NP size for brain delivery is 10–30 nm. We prepared PEG-coated gold NPs in this size range (Au_*x*_@PEG_1k_ NPs) by reacting citrate-capped gold cores of two different diameters (*x* = 3 or 13 in nm; Figure S3) with excess thiolated PEG strands of 1000 Da in molecular weight. By negatively staining the NP for transmission electron microscopy (TEM),^16^ the overall NP physical diameter (gold core plus PEG shell; Figure S3) matches the hydrodynamic size as measured by dynamic light scattering (∼11 nm for Au_3_@PEG_1k_ NP and ∼23 nm for Au_13_@PEG_1k_ NP; Table S3), implying a dense PEG coating. We confirmed the dense PEG coating [>3.5 PEG strands per nm^2^ of gold surface^17^] by (i) indirect measurement of excess thiolated PEG strands unattached to the gold core during synthesis of Au_*x*_@PEG_1k_ NP by the Ellman’s test and (ii) direct measurement of the organic content of Au_*x*_@PEG_1k_ NP by thermogravimetric analysis (TGA).^14^ Au_*x*_@PEG_1k_ NPs were stable in artificial CSF^18^ and 50% fetal bovine serum (FBS) at 37 °C 24 h postincubation (Figures S4–S7 and Tables S4–S5); with CSF treatment and serum adsorption, the hydrodynamic size of both NPs only became slightly larger. Both NPs entered SK-N-MC neuroblastoma and bEnd.3 brain endothelial cells by inductively coupled plasma mass spectrometry (ICP-MS) measurements of the gold contents in cells and label-free confocal reflectance imaging (as gold NPs can reflect light) without cytotoxicity (Figures S8–S10).

Initially, we assessed the optimal NP size for delivery to the HD brain. We i.v. injected both NP types into R6/2 mice at the age of Week 10, keeping the gold dose constant at 700 μg per animal; this dose was used for i.v.-injected siRNA-coated gold NPs for treating brain glioblastoma in mice.^19^ By ICP-MS analysis, the blood circulation half-lives of Au_3_@PEG_1k_ and Au_13_@PEG_1k_ NPs were ∼35 h and ∼15 h, respectively (Figure S11). Blood plasma isolated from the NP-injected mice revealed the red color characteristic of gold NPs 24 h postinjection (Figures S12–S13 and Table S5), echoing the *in vivo* stability of NPs. On the organ-level distribution of both NP types, upon 24 h postinjection, ICP-MS analysis of the brain showed progressively higher accumulation of Au_3_@PEG_1k_ NP than Au_13_@PEG_1k_ NP from the age of Week 6 (onset of early stage HD symptoms) to Week 10 (near late-stage HD)^20^ (Figure 1A); we did not detect any evident difference in brain accumulation between the two NP types at Week 4 (presymptomatic stage).^20^ The liver showed more abundant accumulation of Au_13_@PEG_1k_ NP than Au_3_@PEG_1k_ NP (Figures S14–S15), matching the blood pharmacokinetics data. At the tissue level, ICP-MS measurements revealed more abundant accumulation of Au_3_@PEG_1k_ NPs in various brain compartments (hippocampus, cortex, striatum, cerebellum, and others) than Au_13_@PEG_1k_ NPs from Week 6 onward (Figures 1B and S16). Thus, we selected Au_3_@PEG_1k_ NP for downstream delivery and efficacy studies.

**Figure 1:**
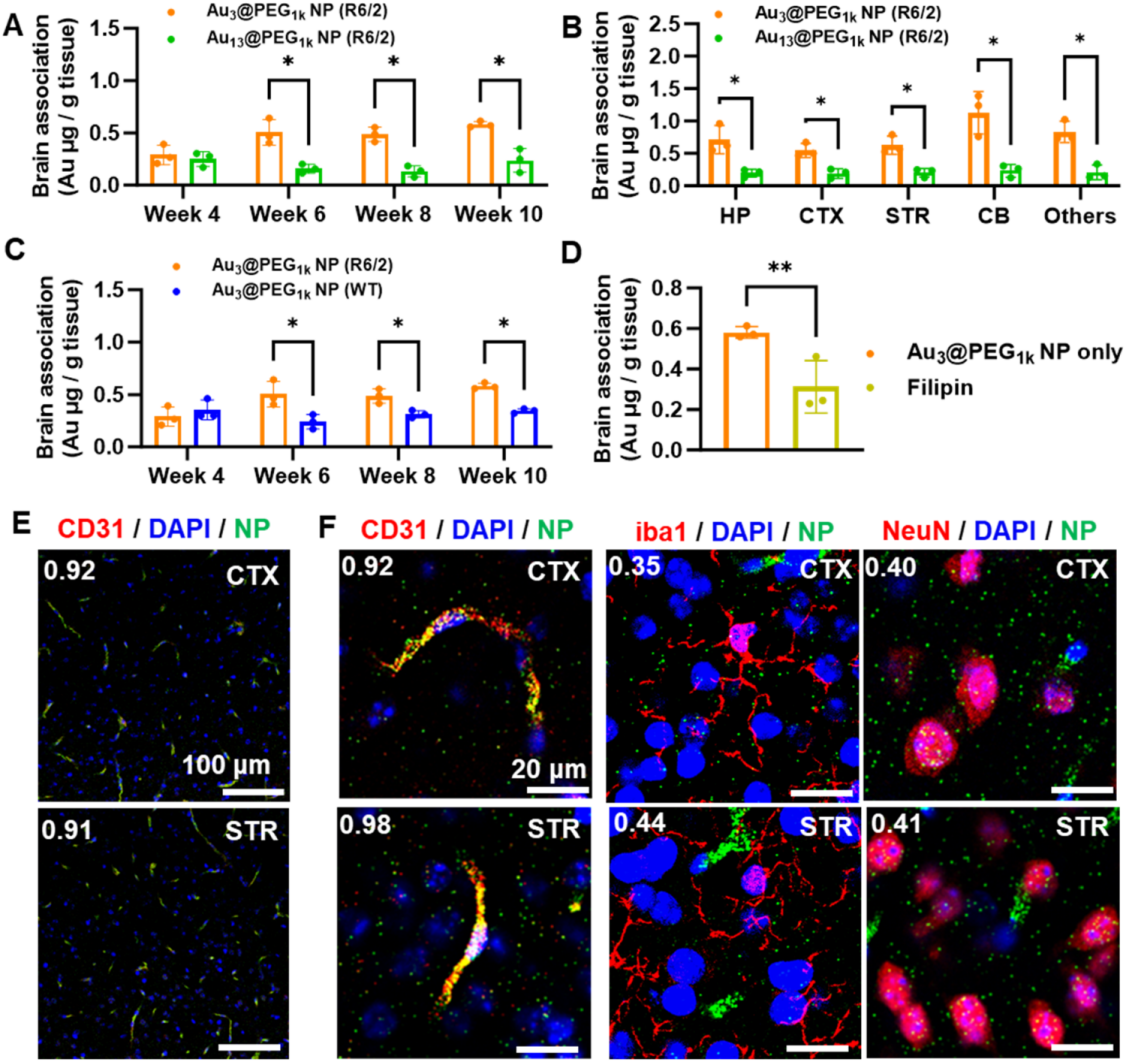
Delivery of Au*_x_* @PEG*_y_* NPs to the brain of R6/2 Huntington’s disease (HD) mice 24 h post intravenous (i.v.) injection. (A) Delivery of polyethylene glycol-conjugated gold NP (Au_*x*_@PEG_*y*_ NPs) to the brain as a function of NP size [Au_3_@PEG_1k_ NP (orange) or Au_13_@PEG_1k_ NP (green)] and age. *x* = NP core diameter (in nm); *y* = PEG molecular weight (in Da). Data are from 3, across 4 experiments. (B) Delivery of Au_3_@PEG_1k_ NP (orange) and Au_13_@PEG_1k_ NP (green) to the brain of Week 10 R6/2 HD mice. Hippocampus (HP), cortex (CTX), striatum (CTX), cerebellum (CB) and other regions (Others). There was more abundant uptake of Au_3_@PEG_1k_ NPs than Au_13_@PEG_1k_ NPs across all brain regions. Data are from *n* = 3, across 1 experiment. (C) Distribution of Au_3_@PEG_1k_ NPs in R6/2 mice (orange) or healthy littermates (blue) as a function of age. Data are from *n* = 3, across 2 experiments. (D) Pharmacological inhibition of caveolin-mediated endocytosis by filipin (olive) reduced the accumulation of Au_3_@PEG_1k_ NPs in the brain of Week 10 R6/2 mice. Orange: untreated mice. Data are from *n* = 3, across 2 experiments. For (A–D), statistical significance was evaluated using unpaired Student’s *t* test and *P < 0.05, **P < 0.01. Gold content in the brain was measured by ICP-MS. All bars and error bars represent mean ± SD (E) Confocal reflectance images of the cryosections of CTX and STR of Week 10 R6/2 HD mice 24 h postinjection of Au_3_@PEG_1k_ NPs. Red: brain endothelial cells (CD31); Blue: nucleus (DAPI); Green: Gold NPs (green); White (top left): Manders' coefficient (MOC) between gold reflectance (green) and CD31 (red). (F) Enlarged confocal reflectance images of the cryosections of CTX and STR of Week 10 R6/2 HD mice. Red: brain endothelial cells (CD31), neurons (NeuN) or activated microglia (iba1); Blue: nucleus (DAPI); Green: Gold NPs (green); White (top left): Manders' coefficient (MOC) between gold reflectance (green) and CD31 (red).

To explore the effect of disease stage on brain delivery, we injected Au_3_@PEG_1k_ NPs to R6/2 HD mice and healthy wild-type (WT) littermates of the same age. 24 h postinjection, ICP-MS analysis of the brain revealed progressively more abundant accumulation of NPs in R6/2 mice than healthy WT mice from Weeks 6 to 10 (Figure 1C; Week 4:0.29 vs 0.35 μg/g; Week 6:0.50 vs 0.24 μg/g; Week 8:0.49 vs 0.31 μg/g; Week 10:0.58 vs. 0.34 μg/g), indicating selective delivery to the HD brain. At the tissue level, we detected enhanced delivery to the cerebellum of R6/2 mice at Week 8 and to most brain compartments at Week 10 (Figure S17). Such selective delivery prompted us to ask whether the NP entered the HD brain via passive or active transport. Previous reports documented BBB impairment or leakage in R6/2 HD mouse models^21^ and HD patients.^22^ Here, i.v. injection of Evans blue, a chemical dye impermeable to the healthy intact BBB, yielded mildly stronger fluorescence of the brain of Week 10 R6/2 mice than WT mice; direct visualization of blue color was infeasible (Figure S18). Western blot analysis revealed the downregulation of tight junction protein ZO-1 in the brain parenchyma of Week 10 R6/2 mice relative to healthy WT mice (Figure S19). These results suggest some BBB leakiness to facilitate passive transport of Au_3_@PEG_1k_ NPs in R6/2 mice, contrary to the visible Evans blue color in the brain of mice with glioblastoma [whose compromised BBB enabled the passage of larger ∼25 nm RNA NPs^19^]. Next, we explored the possibility of active transport of Au_3_@PEG_1k_ NP across the BBB using three *in vitro* models, including basic Transwell setups seeded with mouse bEnd.3 cells and human hCMEC/D3 cells [both commonly used in the field of brain nanomedicine^23^] and an advanced fluid-flow microchip^24^ seeded with human embryonic stem cell-induced brain microvascular endothelial cells (iBMECs) that gives a 6-fold higher transendothelial resistance than the two basic models. For the three BBB models of different species (human vs. mouse) and levels of tightness (bEnd.3 vs iBMEC), pharmacological inhibition with filipin (caveolin-mediated endocytosis)^25^ and sodium azide (energy-dependent cellular uptake) reduced NP permeation of the BBB by 31–58% and 56–98%, respectively (Figures S20–S22), proof of the key role of active cellular transport in BBB penetration without the confounding factor of passive leakage through paracellular gaps. As no *in vitro* BBB model can fully recapitulate the *in vivo* characteristics of the brain, we further showed that treatment of Week 10 R6/2 mice with filipin attenuated brain accumulation (Figure 1D), implying active NP uptake by brain cells *in vivo*; such pharmacological treatment did not induce cellular toxicity (Figures S20–S22). To verify the cellular entry of gold NPs in the brain tissue, we used confocal imaging because it enables the capturing of reflectance signals of gold NP on specific focal planes along the *z*-direction.^26^ A representative large, stitched confocal reflectance image of silver-enhanced coronal brain sections captured NPs throughout the tissue (including extracellular space and cells) 24 h postinjection (Figure S23). Magnified confocal reflectance images depicted strong localization of NPs in endothelial cells [CD31; Manders’ coefficient (MOC) ∼ 0.9] in both cortex and striatum (Figures 1E–F and S24–S25), the most severely affected compartments in HD.^27^ Tissues of uninjected mice had no silver stain reflectance signals (Figures S26–S28), excluding the possibility of false signals that arose from the reflection of cellular components in the brain. [During silver enhancement, silver ions will be catalytically reduced to metallic silver by gold NPs and deposited on their surface, forming amplified microscale silver clusters for visualization under a microscope].^28^ Confocal reflectance imaging detected moderate colocalization of NPs in neurons (NeuN; a marker present in both nucleus or perinuclear cytoplasm;^29^ Figures 1F and S29 and Videos S1 and S2) and activated microglia [ionized calcium-binding adaptor molecule (iba) 1], with MOC of ∼0.4 (Figure 1F). These data suggested ample, initial NP residency in the BBB as transient depots^9,30^ with some localization inside neurons 24 h postinjection before ultimate entry to neurons en masse at the point of efficacy evaluation (Figure 2); they did not imply indefinite entrapment in the BBB.

**Figure 2:**
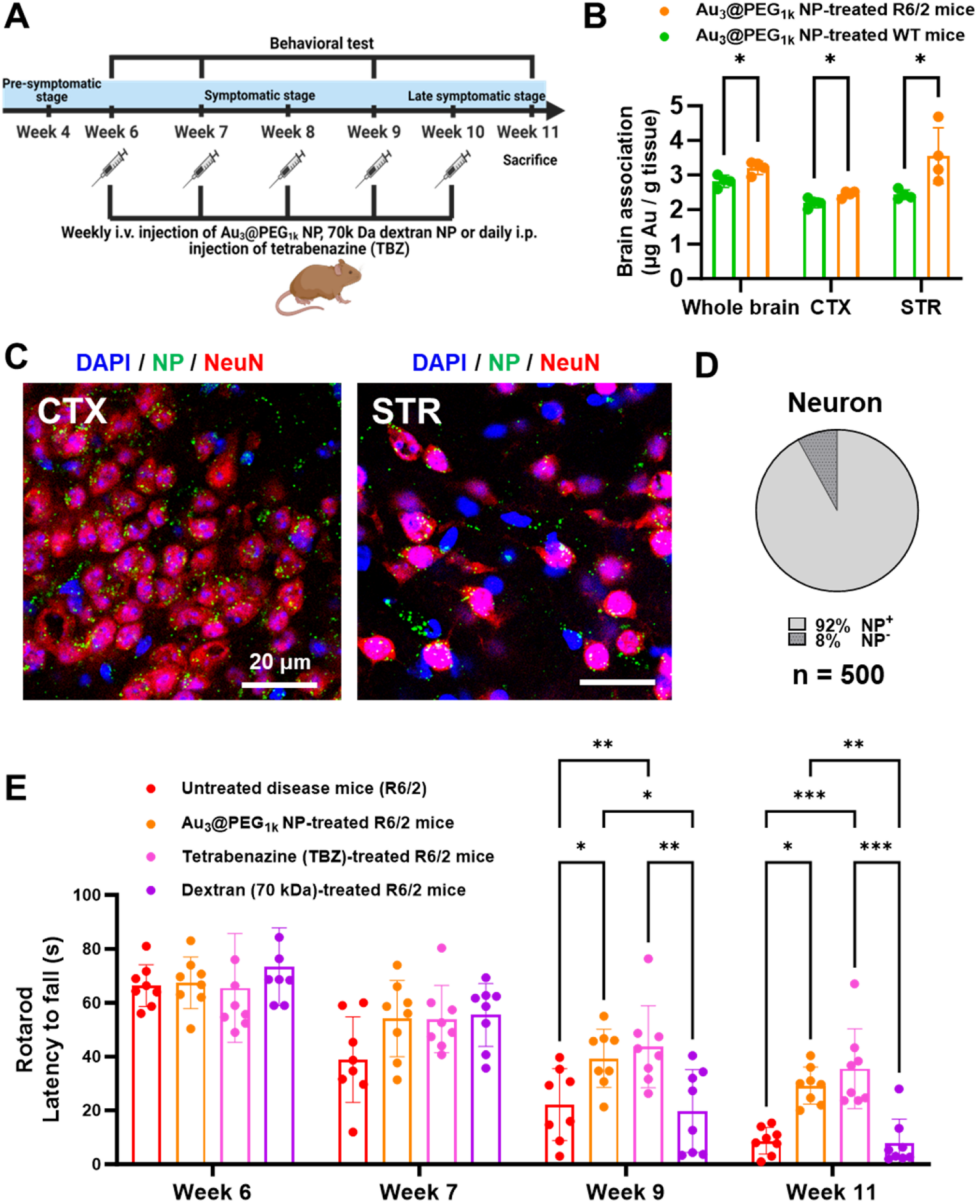
Au_3_@PEG_1k_ NP entered the brain and alleviated motor deficit in R6/2 HD mice. (A) From the age of Weeks 6 to 10, R6/2 mice received weekly i.v. injections of Au_3_@PEG_1k_ NPs or similarly sized dextran (70 kDa), or daily intraperitoneal injections of tetrabenazine (TBZ), all sacrificed at Week 11. Behavioral tests took place at the age of Weeks 6, 7, 9, and 11. (B) ICP-MS results showed higher brain accumulation of Au_3_@PEG_1k_ NPs in R6/2 mice (orange) than healthy wildtype (WT) littermates (green) at the age of Week 11, including the cortex (CTX) and striatum (STR). Data are from *n* = 4. (C) Confocal reflectance images of the cryosections of CTX and STR from Au_3_@PEG_1k_ NP-treated R6/2 mice at the age of Week 11. Red: neurons (NeuN) Blue: nucleus (DAPI); Green: Gold NPs (green). (D) Manual counting of Au_3_@PEG_1k_ NP-containing neurons in multiple brain cryosections. By counting 500 neurons in both cortex and striatum of Au_3_@PEG_1k_ NP-treated R6/2 mice sacrificed at the age of Week 11, we found that 92% of the neurons contained gold reflectance signals. This result indicates the presence of gold NPs in the brain parenchyma. (E) Rotarod test of untreated (red), Au_3_@PEG_1k_ NP-treated (orange), tetrabenazine (TBZ)-treated (pink), 70 kDa dextran-treated (purple) R6/2 mice as a function of age. At the age of Weeks 9 and 11, Au_3_@PEG_1k_ NPs and TBZ improved the latency to fall relative to untreated control, while 70 kDa dextran did not for all ages tested. Data are from *n* = 8, across four experiments. Statistical significance was evaluated using Two-Way ANOVA with Tukey’s post hoc test for multiple comparisons. *P < 0.05; **P < 0.01; ***P < 0.001. All bars and error bars represent mean ± SD. There was little difference in efficacy between gold NPs and TBZ.

Besides the BBB, Au_3_@PEG_1k_ NP may also enter the brain by undergoing filtration at the blood-CSF barrier^31^ because similarly sized proteins can cross the blood-CSF barrier.^32^ ICP-MS measurements revealed detectable gold levels in the choroid plexus (site of the blood-CSF barrier) 24 h postinjection of Au_3_@PEG_1k_ NP into Week 10 R6/2 HD mice (Figure S30). Confocal imaging captured gold reflectance signals in the choroid plexus (with its epithelial cells immunostained by E-cadherin) and adjacent BBB (with its endothelial cells stained by CD31) (Figure S31). Similarly, the data only proved the existence of NPs in the choroid plexus 24 h postinjection, while not suggesting indefinite confinement in the blood-CSF barrier.

### Entry to Neurons and Alleviation of HD without Toxicity

In our treatment regimen, we injected Au_3_@PEG_1k_ NP weekly into R6/2 mice between the age of Weeks 6 and 10 (Figure 2A) and sacrificed them at Week 11. After 5 weeks of treatment, ICP-MS measurements revealed higher gold contents in the whole brain, cortex, or striatum of R6/2 mice than WT mice, confirming selective delivery to the HD brain (Figure 2B; whole brain: 3.2 vs 2.8 μg/g; cortex: 2.3 vs 2.2 μg/g; striatum: 3.6 vs 2.3 μg/g). Confocal imaging portrayed less prominent NP accumulation in endothelial cells (MOC: 0.1; Figure S32) or paracellular space; rather, NPs were found in neurons and microglia in the cortex and striatum (Figures 2C and S33). By counting 700 cells (neurons and microglia combined) in multiple brain sections, we found that 92% of neurons (Figure 2D) and 78% of microglia (Figure S34), in the cortex and striatum combined, contained gold reflectance signals. Therefore, our data indicated initial NP localization in the brain capillaries 24 h after a single injection (Figure 1E), but such strong NP localization was no longer detectable after five weekly injections (Figure S32); therefore, most NPs eventually exited the BBB and entered neurons in the adjacent brain tissue in the cortex and striatum as revealed by confocal reflectance imaging (Figures S35–S40). Similarly, confocal images revealed the presence of gold NPs in neurons near the blood-CSF barrier after five weekly injections (*e.g*., vermal Purkinje cells around the fourth ventricle and hippocampal cortical neurons in the periventricular region; Figures S41–S46), possible evidence of brain entry via the blood-CSF barrier. Note that (i) we saline-perfused the brain to remove any free gold NP in the blood capillary lumen before ICP-MS measurements, (ii) the blood circulation half-life of gold NPs is 35 h (Figure S11), which implies a limited level of gold NP in blood after five weekly injections, (iii) our confocal images of the HD brain in Figure S32 depicted pronounced clearance of gold NPs from the BBB endothelium at the same time point, (iv) the injection route was i.v., not intranasal, intrathecal, or intracranial, (v) the background level of gold in the body is virtually nil. Thus, the gold contents in the brain after five weekly injections could only result from gold NP crossing the brain barriers and their release into the adjacent brain tissues.

Initially, we used the rotarod test to evaluate the “latency to fall” of R6/2 mice, or the time duration for staying on an accelerating rotating rod.^33^ As negative control, 70 kDa dextran NP, similarly sized as Au_3_@PEG_1k_ NP (Table S3), was i.v. injected at the same NP concentration weekly. As positive control, tetrabenazine was intraperitoneally (i.p.) injected daily at the same cumulative weekly dose as literature precedent (2.5 mg/kg^34^). Relative to their behavior at Week 6, the same group of mice at Week 7 showed 20% lower average latency to fall after receiving a single i.v. injection of Au_3_@PEG_1k_ NP or 40% lower average latency if left untreated. Therefore, one injection of NP ameliorated motor deficit by 50% (*i.e*., 20% ÷ 40%) over a week (Figure 2E), justifying a weekly injection schedule. At Week 11, repeated injections of NPs ameliorated motor deficit by 67% (Figure 2E), similarly effective to tetrabenazine, but 70 kDa dextran NP did not show improvement (Figure 2E). Next, we conducted the open field test to observe the exploration of R6/2 mice in an open field. Five injections of Au_3_@PEG_1k_ NP yielded a ∼ 40% longer mean total walking distance at Week 11; again, tetrabenazine and Au_3_@PEG_1k_ NP showed similar efficacies, but 70 kDa dextran NP showed no improvement (Figures S47–S48). Our ICP-MS measurements revealed the presence of gold in the muscles of forelimbs and hindlimbs 24 h postinjection, most abundantly in the triceps surae muscles including the gastrocnemius and soleus (Figure S49). Such NP distribution to peripheral skeletal muscles might suggest a therapeutic effect on improving motor function by locally acting on skeletal muscles or the neuromuscular junction rather than the HD brain, but this scenario is unlikely for two reasons. First, we verified the limited degeneration of muscles and neuromuscular junctions in Week 11 R6/2 mice. *Ex vivo* functional tests^18^ revealed no difference in the twitch force, tetanic force, and intratetanic fatigue in the triceps surae muscle and the triceps surae-sciatic nerve complex (Figure S50) between Week 11 R6/2 HD mice and age-matched healthy littermates. This result matches past reports on the emergence of muscle atrophy or neuromuscular abnormalities only for Week 12 R6/2 mice or older.^35^ Also, we verified that Au_3_@PEG_1k_ NP did not alter the motor performance of healthy WT mice (Figures S51–S52). In short, the chances of any remote pharmacological effect from distal locations are slim. At Week 11, confocal imaging revealed less mHTT aggregates in neurons of the cortex and striatum upon Au_3_@PEG_1k_ NP treatment (Figure S53), matching reports on inhibiting Alzheimer’s amyloid-β fibrillization^36^ and Parkinson’s α-synuclein aggregation^37^ by gold NPs. These results indicate the anti-HD efficacy of Au_3_@PEG_1k_ NP.

We further evaluated the immunogenicity of Au_3_@PEG_1k_ NP after the treatment. First, we performed cytokine profiling of 21 chemokines and 9 cytokines in the blood upon five repeated injections of Au_3_@PEG_1k_ NP into healthy littermates (Figure S54) because PEG immunity is a valid concern for nanomedicine.^38^ Profiling a library of 30 chemokines/cytokines in blood revealed significant upregulation of only 3 chemokines (CXCL5, CCL1, and CCL5) and 2 cytokines (IL-4 and GM-CSF), with other markers not significantly changed. The three chemokines play a role in recruiting immune cells, such as neutrophil (CXCL5 ^39^), macrophage (CCL5 ^40^), and T cell (CCL1 ^41^ and CCL5 ^40^). GM-CSF promotes the differentiation and activation of macrophages and dendritic cells, enhancing adaptive immune responses.^42^ IL-4, an anti-inflammatory cytokine, inhibits the production of TNF-α and IL-1β,^43^ and its upregulation matches Au_3_@PEG_1k_ NP-induced upregulation of IL-4 in R6/2 mice (Figure S75B). Also, Au_3_@PEG_1k_ NP increased the levels of IgG antibodies against PEG in serum (Figure S55), matching the cytokine profiling data and proof of mild PEG immunogenicity. Still, Au_3_@PEG_1k_ NP did not affect liver function (Figure S56) by serum biochemistry or increase the levels of pro-inflammatory cytokines (IFN-γ, TNF-α, and IL-6) in the liver or spleen (Figure S57) by immunohistochemistry. Furthermore, in R6/2 HD mice, Au_3_@PEG_1k_ NP did not change hematology, liver and kidney functions, or morphology of major organs (Figures S58–S60), suggesting limited short-term systemic toxicity.

Further, we evaluated the toxicity of Au_3_@PEG_1k_ NP 14 weeks post-treatment (at the age of Week 24 when R6/2 mice with end-stage HD became terminally ill; Figure 3A). Au_3_@PEG_1k_ NPs prolonged the survival of R6/2 mice without affecting the motor behavior of healthy mice (Figures 3B and S61). There was no change in liver and kidney functions or tissue morphology of major organs in R6/2 and healthy mice (Figures S62–S65). ICP-MS measurements showed reduced gold contents in the whole brain, all brain compartments (Figure 3C–D), and other major organs at Week 24 relative to Week 11 in R6/2 (Figure 3E–F) and healthy mice (Figure S66). In short, Au_3_@PEG_1k_ NP exhibits *in vivo* clearance and limited long-term toxicity.

**Figure 3:**
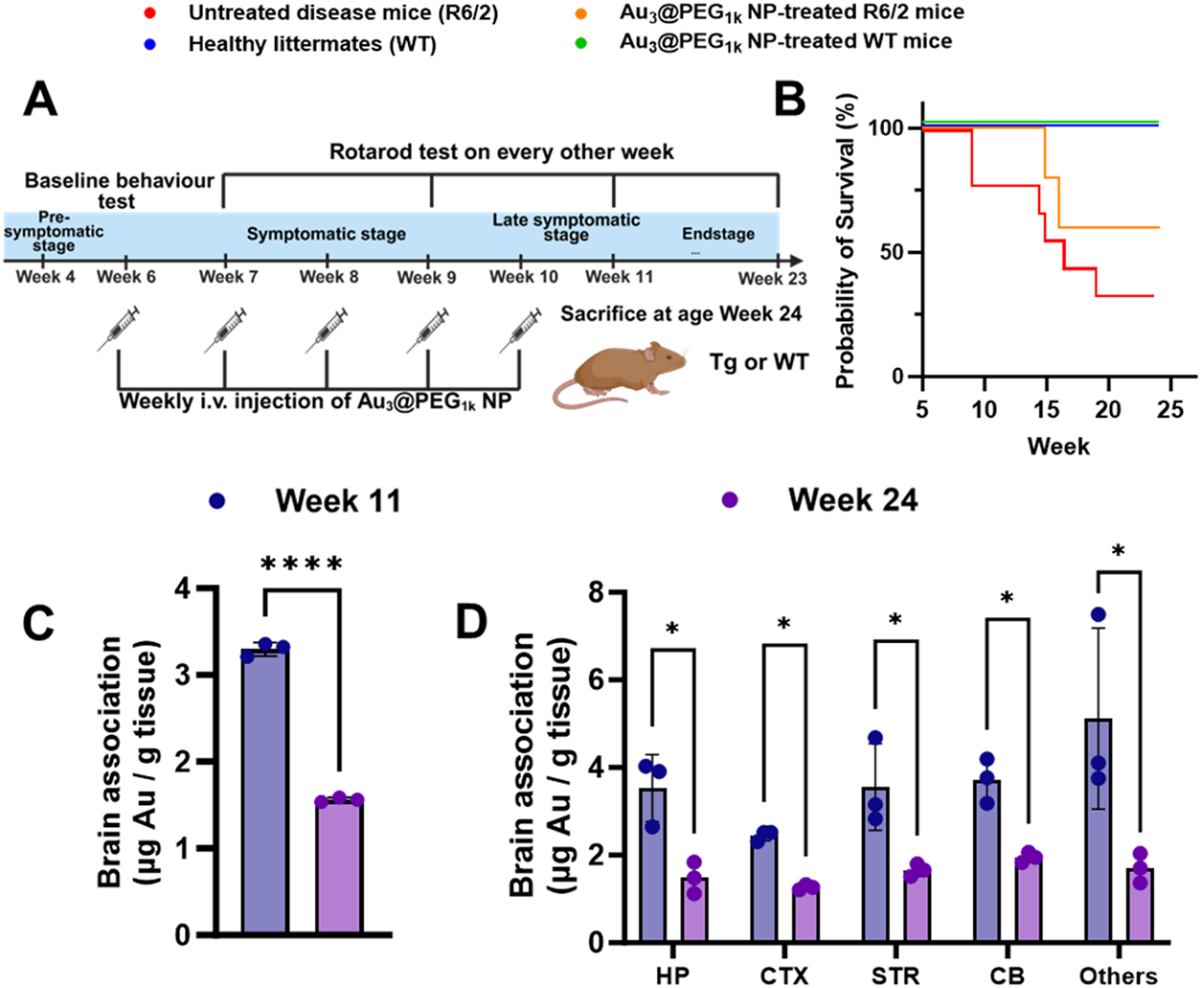
Clearable Au_3_@PEG_1k_ NP prolonged survival rate in R6/2 HD mice without significant toxicity. (A) Schematic illustration for evaluating the long-term behavior of untreated and Au_3_@PEG_1k_ NP-treated healthy littermates (WT) and R6/2 HD (Tg) mice. From the age of Weeks 6 to 10, R6/2 mice received weekly i.v. injections of Au_3_@PEG_1k_ NPs. Behavioral tests started at the age of Week 6 and starting from Week 7, the behavioral test was performed every other week. After the weekly NP injections, the mice were returned to the cage for up to 12 additional weeks and eventually sacrificed at the endstage period due to the suffering of the animals, following the guidance of the CUHK Animal Experimentation Ethics Committee. The figure was created using BioRender. (B) Long-term survival of untreated mice [WT (blue); R6/2 (red)] and Au_3_@PEG_1k_ NP-treated mice [WT (green); R6/2 (orange)]. Data are from *n* = 4–9, across 2 experiments. *P* value is 0.047. Statistical significance was determined by log-rank Mantel–Cox test. (C–D) ICP-MS results showed that, when compared to treatment completion at the age of Week 11, the gold content in the brain of Au_3_@PEG_1k_ NP-treated R6/2 mice was reduced at the age of Week 24, across all compartments. Data are from *n* = 3, across 2 experiments. Statistical significance was evaluated using unpaired Student’s *t* test. *P < 0.05; **P < 0.01; ***P < 0.001, ****P < 0.0001. All bars and error bars represent mean ± SD.

### Upregulation of Oxidative Phosphorylation

To elucidate the anti-HD mechanism of Au_3_@PEG_1k_ NP, we utilized unbiased proteomics analysis to identify changes in protein expression in the HD brain of R6/2 mice at Week 11, based on the treatment regimen in Figure 2A. We detected 52 differentially expressed proteins (DEPs) in the pairwise comparison between the Au_3_@PEG_1k_ NP and untreated groups (Au_3_@PEG_1k_ NP vs. untreated), with a cutoff fold change (FC) ≥ 1.5 or FC ≤ 1/1.5 and *P* < 0.05 (Figures 4A and S67). Of the enriched Kyoto Encyclopedia of Genes and Genomes (KEGG) pathways, “oxidative phosphorylation” was the most enriched (Figures 4B and S68–S69). The “HD” KEGG pathway was also enriched (Figure 4B); 8 out of 9 of its constituent DEPs were related to oxidative phosphorylation in KEGG and were all upregulated (Figure 4C), including cytochrome c oxidase (Cox4i1, Cox5a, Cox5b, Cox6b1, Cox7c, Mtco2) and nicotinamide adenine dinucleotide hydride (NADH) dehydrogenase (Ndufb11, Ndufs6). In oxidative phosphorylation, the electrons derived from NADH (which regulates cellular energy metabolism in glycolysis) break down oxygen to release energy and fuel the synthesis of adenosine triphosphate (ATP). Recent *in vitro* evidence showed that gold NPs catalyze the oxidation of NADH to NAD^+^ for activating ATP production,^44^ echoing our *in vivo* result that Au_3_@PEG_1k_ NP upregulates oxidative phosphorylation (Figure 4B). The KEGG pathways of “Parkinson’s disease” and “Alzheimer’s disease” were also enriched as they share similar upregulated DEPs as the “HD” KEGG pathway (Table S6). This result matches the reported efficacy of gold NPs against both neurodegenerative disorders (Table S2).

**Figure 4:**
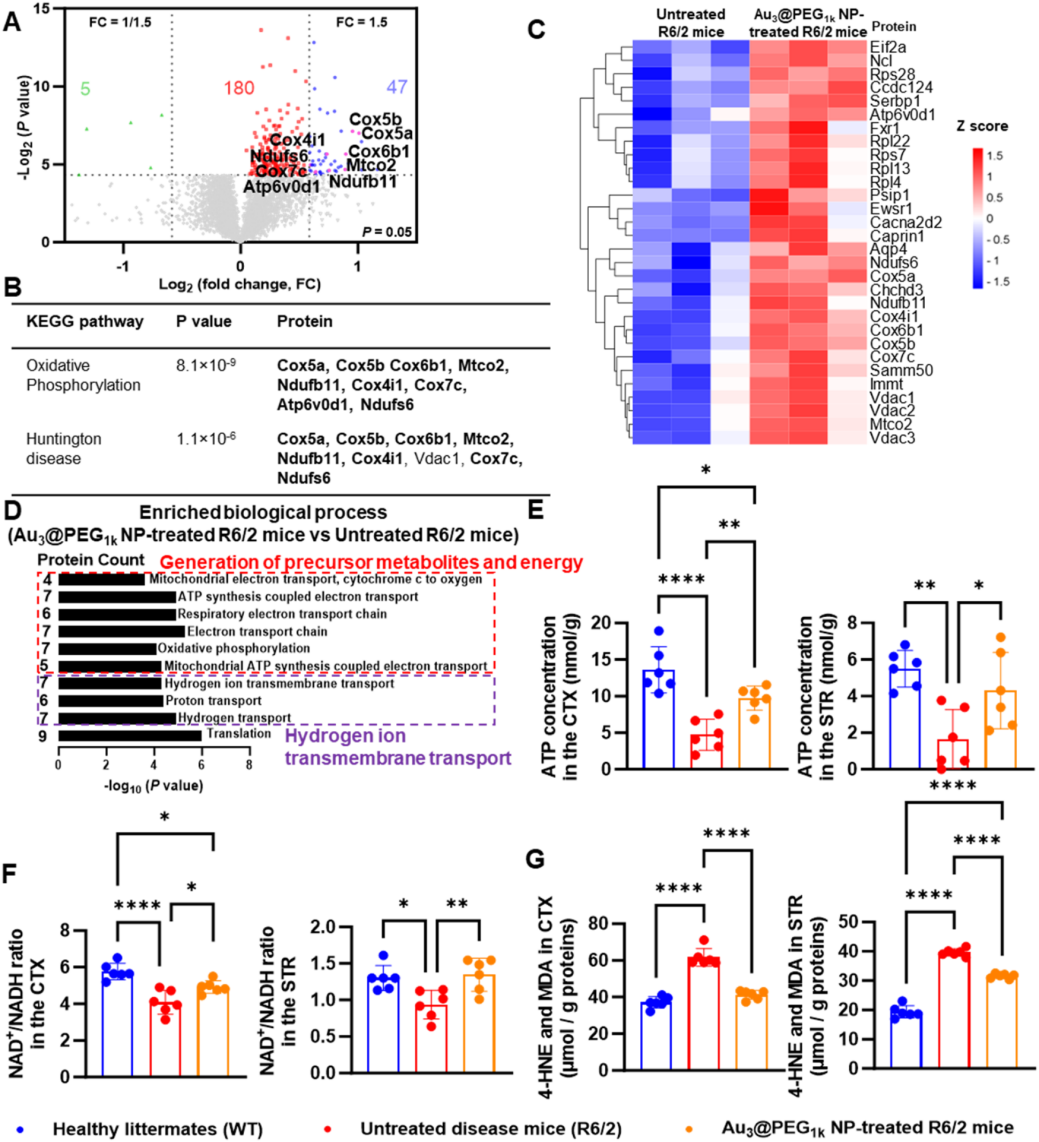
Treatment of Au_3_@PEG_1k_ NP activated oxidative phosphorylation in the brain of R6/2 mice. (A) Volcano plot of the differentially expressed proteins (DEPs) by comparing “Au_3_@PEG_1k_ NP-treated R6/2 mice” against “untreated R6/2 mice” groups at Week 11. The cutoff fold change (FC) was FC ≥ 1.5 or FC ≤ 1/1.5 with *P* < 0.05. Bolded proteins are related to oxidative phosphorylation. Data are from *n* = 3, across one experiment. (B) Enriched HD-related Kyoto Encyclopedia of Genes and Genomes (KEGG) pathways from the same comparison, with DEPs related to oxidative phosphorylation bolded. (C) Heat map of all DEPs linked to top 10 enriched gene ontology (GO) terms (biological process, cellular component, and molecular function) from the same comparison. (D) Top 10 enriched biological processes from the same comparison. The dashed box surrounds the specific child terms of the higher-order GO term (red) per the ancestor chart. Data are from *n* = 3, across 1 experiment. (E) In CTX and STR, ATP production was higher for Au_3_@PEG_1k_ NP-treated (orange) than untreated (red) R6/2 mice. Data are from *n* = 6, across two experiments. (F) In CTX and STR, the NAD^+^ to NADH ratio was higher for Au_3_@PEG_1k_ NP-treated (orange) than untreated R6/2 mice (red). Data are from *n* = 6, across 2 experiments. (G) In CTX and STR, the levels of malondialdehyde (MDA) and 4-hydroxynonenal (4-HNE; markers of lipid peroxidation) were lower for Au_3_@PEG_1k_ NP-treated (orange) than untreated (red) R6/2 mice. Data are from *n* = 6, across 1 experiment. Statistical significance was evaluated using one-way ANOVA with Tukey’s post hoc test for multiple comparisons. *P < 0.05; **P < 0.01; ***P < 0.001, ****P < 0.0001. All bars and error bars represent mean ± SD.

Next, 31 out of the 52 DEPs were upregulated and related to the top ten significantly enriched gene ontology (GO) terms (Figures 4C and S70). On biological process, most GO terms were clinically relevant to HD,^45^ such as “ATP synthesis coupled electron transport” and “respiratory electron transport chain” (Figures 4D and S70). On cellular component, the GO terms were related to “mitochondrion” and “mitochondrial respiratory chain”. On molecular function, the GO terms were related to “oxidoreductase activity” and “transmembrane transporter activity”. Similar to the KEGG pathway, the associated proteins in all GO terms were Cox, NADH, and voltage-dependent anion-selective channel (Vdac1, Vdac2, Vdac3, Tables S7–S9), linked to oxidative phosphorylation. As oxidative phosphorylation was found downregulated in HD mice^46^ and patients,^47^ we hypothesized that the therapeutic action of Au_3_@PEG_1k_ NP stems from the upregulation of oxidative phosphorylation and ATP metabolism in the HD brain. As proof, Au_3_@PEG_1k_ NP enhanced the level of ATP (Figure 4E) and the ratio of NAD^+^ to NADH (Figure 4F) in the striatum and cortex of R6/2 mice, consistent with the biological process of “generation of precursor metabolites and energy” (Figure 4D). Lastly, we considered “lipid peroxidation”, whereby oxidants (*e.g*., free radicals) attack lipids containing C–C double bond (*e.g*., polyunsaturated fatty acids^48^). Au_3_@PEG_1k_ NP attenuated the levels of 4-hydroxynonenal (HNE) and malondialdehyde (MDA), markers of lipid peroxidation, in the cortex and striatum (Figure 4G). This result suggests improved mitochondrial function,^49^ matches the biological processes of “generation of precursor metabolites and energy” and “hydrogen ion transmembrane transport”^50^ (Figure 4D), and reinforces the restoration of the KEGG pathway of oxidative phosphorylation in the HD brain.

### Inhibition of p38α Phosphorylation and PDK1 Activity

Consequently, we conducted kinome profiling to unbiasedly identify pharmacological targets of Au_3_@PEG_1k_ NP to account for the upregulation of oxidative phosphorylation. Of the 281 kinases screened, ∼200 nM Au_3_@PEG_1k_ NPs inhibited 16 kinases by >85%, with 7 of them related to HD (Figure 5A and Table S10). The three most documented kinases were MAPK14 (p38α), PDK1, and MKK6 (an upstream kinase of p38), and their respective half-maximal inhibitory concentrations (IC_50_’s) for Au_3_@PEG_1k_ NP were 0.789, 2.42, and 3.62 nM. These IC_50_ values, pronouncedly lower than those of the negative control 70 kDa dextran NP, underscore the potency of Au_3_@PEG_1k_ NP (Figures 5B–D and S71). p38α is involved in neuroinflammation^51^ and its phosphorylation (p-p38) was elevated in the striatum of HD patients and mouse models, an event associated with neuronal death and excitotoxicity.^52^ A past clinical trial (NCT03980938) used neflamapimod, a p38α inhibitor, to treat HD. PDK1 activation blocks pyruvate dehydrogenase (PDH), which converts pyruvate into acetyl-coenzyme A in the Kreb cycle. Activated PDK1 causes anaerobic glycolysis and inefficient ATP generation. A past preclinical study validated that dichloroacetate, a PDK1 inhibitor, treated HD in mice by restoring oxidative phosphorylation.^46,53^

**Figure 5:**
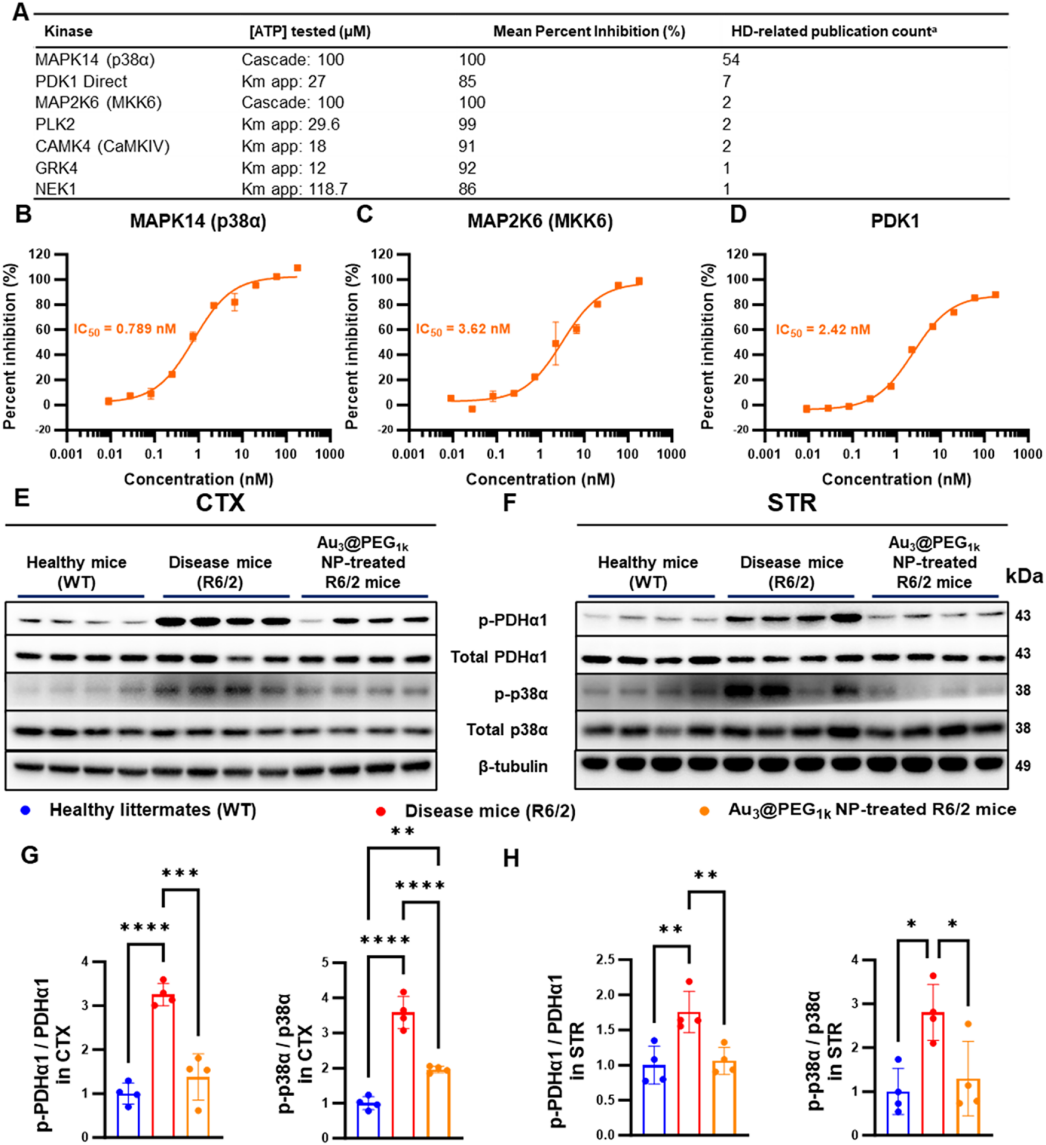
Au_3_@PEG_1k_ NPs inhibited p38α phosphorylation and PDK1 activity in the brain of R6/2 mice. (A) Profiling 281 kinases by the Z-lyte assay revealed that ∼200 nM Au_3_@PEG_1k_ NPs inhibited 7 HD-related kinases by >85%. Two most HD-related kinases identified were PDK1 and p38α, based on cumulative publication count as of August 2024 derived from defined keyword searches of “Huntington’s disease” in PubMed. (B–D), 10-point titration curves and IC_50_ values of Au_3_@PEG_1k_ NP on PDK1, MAPK14 (p38α), and MAP2K6 (MKK6; upstream of p38α) respectively. (E–F), Western blot analysis of the brain at Week 11 revealed the (i) activation of p-PDHα1 (a downstream kinase of PDK1) and p-p38α in R6/2 mice (red) when compared to age-matched healthy WT littermates (blue) and (ii) inhibition of both kinases upon Au_3_@PEG_1k_ NP treatment of R6/2 mice (orange) when compared to untreated R6/2 mice (red) in CTX and STR. Data are from *n* = 4, across 1 experiment. (G–H), Quantification of the Western blot data in (E–F) for the expression of p-PDHα1 (relative to PDHα1) and p-p38α (relative to total p38α). Statistical significance was evaluated using one-way ANOVA with Tukey’s post hoc test for multiple comparisons. *P < 0.05; **P < 0.01; ***P < 0.001, ****P < 0.0001. All bars and error bars represent mean ± SD.

To validate the kinome profiling data *in vivo*, we proved by Western blot that Au_3_@PEG_1k_ NP inhibited p-p38α and p-PDH (a downstream target of PDK1) in the cortex and striatum of R6/2 mice at Week 11 (Figure 5E–H), matching our previous report of p-p38α inhibition by gold NPs in the kidney.^14^ Additionally, i.p. injection of their respective inhibitors, p38α MAPK-N-1 and dichloroacetate, daily into R6/2 mice led to improved motor behavior at Weeks 9 or 11 by the rotarod test (Figure 6A–B) and longer total walking distances at Week 11 by the open field test (Figures S72–S73). Western blot confirmed the inhibition of p-p38α and p-PDH by their inhibitors in the cortex and striatum (Figure 6C–F). Therefore, we verified p-p38α and PDK1 as therapeutic targets of Au_3_@PEG_1k_ NP in the HD brain.

**Figure 6:**
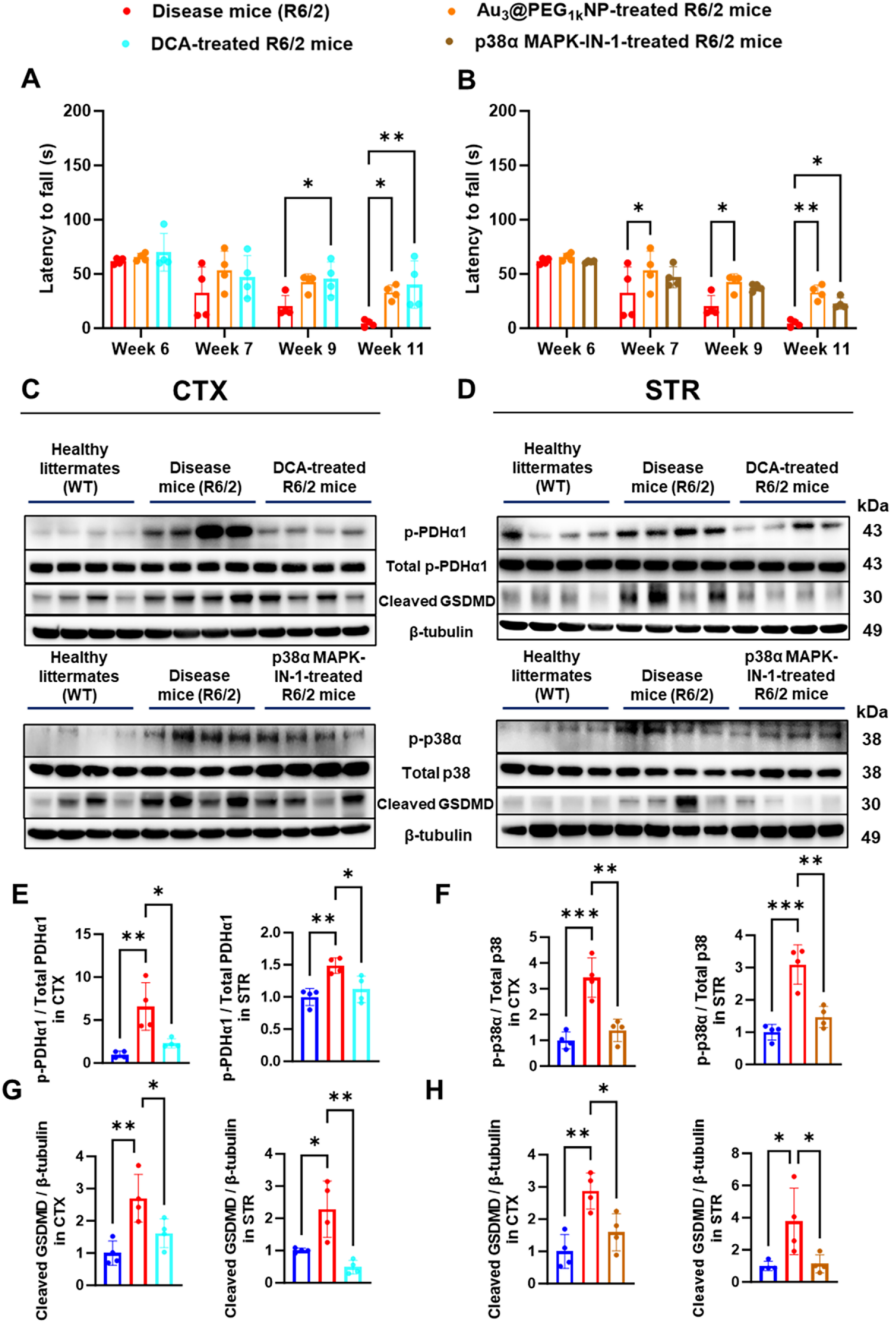
Inhibition of PDK1 activities and phosphorylation of p-p38α by their respective inhibitors in the cortex (CTX) and striatum (STR) alleviated HD and reduced pyroptosis. (A) Rotarod test of untreated (red), Au_3_@PEG_1k_ NP-treated (orange), and dichloroacetate (DCA; an inhibitor of PDK1)-treated (cyan) R6/2 mice as a function of age, based on the treatment plan in Figure 2A. At Week 9, DCA treatments showed improved motor activity. At Weeks 11, both Au_3_@PEG_1k_ NP- and DCA treatments improved motor activity than untreated. Data are from *n* = 4, across four experiments. Statistical significance was evaluated using Two-Way ANOVA with Tukey’s post hoc test for multiple comparisons. (B) Rotarod test of untreated (red), Au_3_@PEG_1k_ NP-treated (orange), and p38α MAPK-IN-1 (an inhibitor of p38α)-treated (brown) R6/2 mice as a function of age, based on the treatment plan in Figure 2A. At Week 7 and 9, Au_3_@PEG_1k_ NP-treated R6/2 mice showed improved motor activity. At Weeks 11, both Au_3_@PEG_1k_ NP- and p38α MAPK-IN-1 treatments improved motor activity more than untreated. Data are from *n* = 4, across four experiments. Statistical significance was evaluated using Two-Way ANOVA with Tukey’s post hoc test for multiple comparisons. (C–H) Western blot analysis revealed the (i) activation of p-PDHα1 (a downstream kinase of PDK1) in R6/2 mice (red) when compared to healthy WT littermates (blue) and (ii) inhibition of p-PDHα1 upon dichloroacetate (DCA; pharmacological inhibitor of PDK) treatment of R6/2 mice (cyan) when compared to untreated R6/2 mice (red) in CTX and STR. (iii) activation of p-p38α in R6/2 mice (red) when compared to healthy WT littermates (blue) and (iv) inhibition of p-p38α upon p38α MAPK-IN-1 (pharmacological inhibitor of p38α) treatment of R6/2 mice (brown) when compared to untreated R6/2 mice (red) in CTX and STR. All data are from *n* = 4, across one experiment. Statistical significance was evaluated using One-Way ANOVA with Tukey’s post hoc test for multiple comparisons. *P < 0.05; **P < 0.01; ***P < 0.001. All bars and error bars represent mean ± SD.

### Inhibition of Pyroptosis-Mediated Cell Death

Past reports documented a linkage of p38α^54^ and PDK1 ^55^ to pyroptosis, a pathway of cell death in HD pathogenesis^2^ whereby activated caspases cleave gasdermins to form pores in the cell membrane.^56^ We verified that i.p. injection of their respective inhibitors, p38α MAPK-N-1 and dichloroacetate, daily into R6/2 mice led to reduced cleavage of gasdermin D shown by WB analysis (Figure 6C–D,6G–H). Further, we proved that Au_3_@PEG_1k_ NPs inhibited pyroptosis; Western blot data revealed that Au_3_@PEG_1k_ NP inhibited nucleotide-binding domain, leucine-rich-containing family, pyrin domain-containing protein (NLRP)-3, gasdermin D cleavage, and caspase 1 activity in the striatum and cortex of R6/2 mice (Figure 7A–D,F). As NLRP-3 inflammasome and PDK1 activities trigger the secretion of proinflammatory cytokines,^55,57^ we verified that Au_3_@PEG_1k_ NP reduced inflammation in the striatum and cortex. Western blot (Figure 7A,B,E) and confocal immunofluorescence (Figure S74) showed the inhibition of iba1, and enzyme-linked immunosorbent assay (ELISA) showed reduced cytokine markers of HD, including interleukin (IL)-1β, tumor necrosis factor (TNF)-α, IL-4, and IL-6, in the striatum and cortex (Figures 7G–H and S75). Collectively, Au_3_@PEG_1k_ NP reduced pyroptosis and neuroinflammation in the HD brain, matching the alleviation of neuroinflammation via PDK1 inhibition using dichloroacetate.^58^

**Figure 7:**
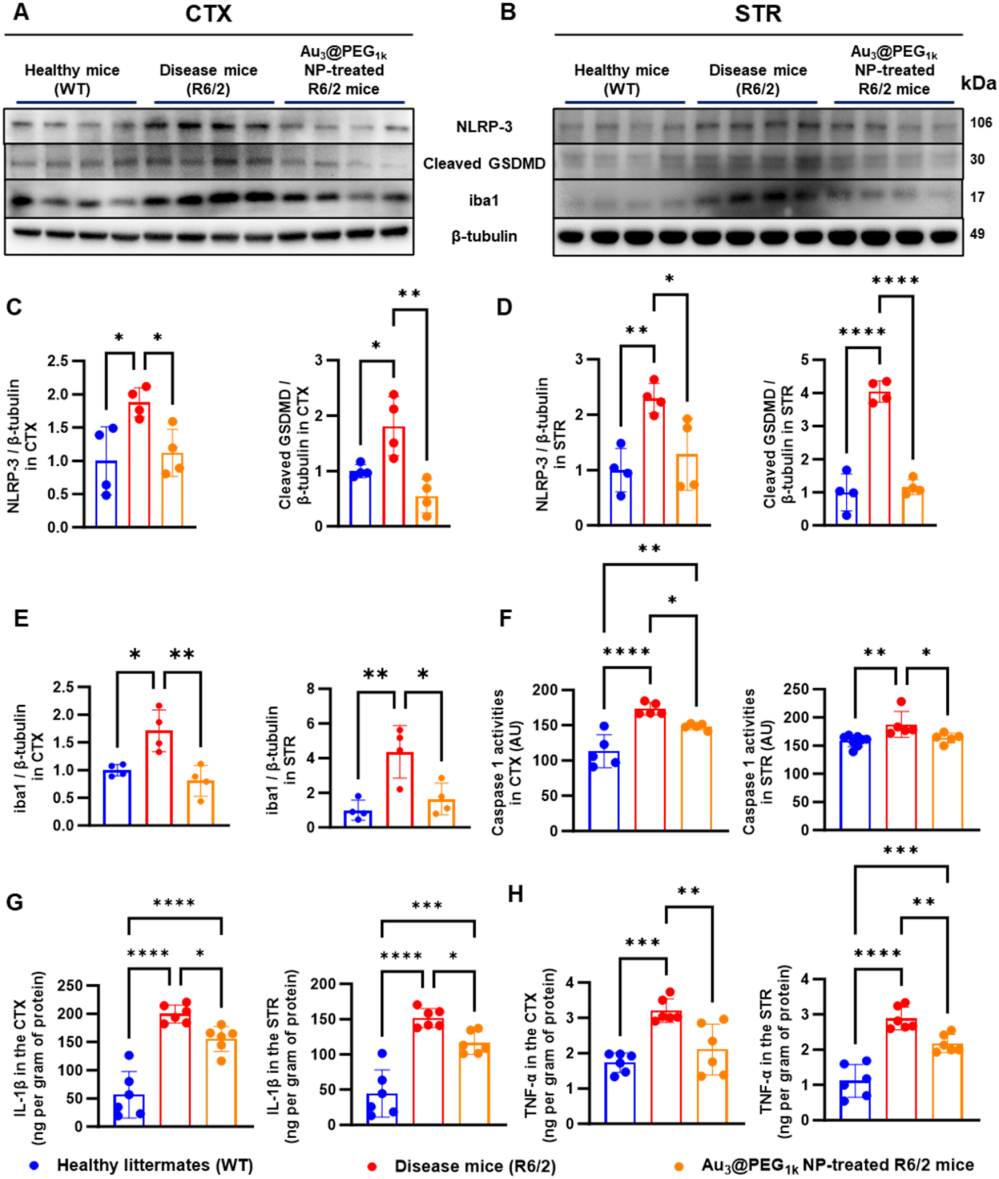
Au_3_@PEG_1k_ NP reduced pyroptosis-related programmed cell death and inflammation in the brain of R6/2 mice. (A–B), Western blot analysis revealed inhibition of NLRP-3, cleaved gasdermin D (GSDMD), caspase 1 (markers of pyroptosis) and iba1 in the CTX and STR in Au_3_@PEG_1k_ NP-treated mice (orange) relative to untreated mice (red) in CTX and STR; blue indicates the levels of uninjected healthy littermates. Data are from *n* = 4, across 1 experiment. (C–E), quantification of the Western blot data in (A)–(B) for the expression of NLRP-3, cleaved GSDMD, and iba1 relative to β-tubulin. (F) Au_3_@PEG_1k_ NP (orange) reduced caspase 1 activities in CTX and STR when compared to untreated control (red). Data are from *n* = 5, across 1 experiment. AU = arbitrary unit. (G–H), ELISA analysis revealed inhibition of proinflammatory cytokines in CTX and STR in Au_3_@PEG_1k_ NP-treated mice (orange) when compared to untreated R6/2 mice (red). Data are from *n* = 6, across 1 experiment. All statistical significance was evaluated using one-way ANOVA with Tukey’s post hoc test for multiple comparisons. *P < 0.05; **P < 0.01; ***P < 0.001, ****P < 0.0001. All bars and error bars represent mean ± SD.

## Conclusion

We show that 11 nm Au_3_@PEG_1k_ NPs selectively accumulate in the HD brain over healthy brain and enter neurons and microglia in the cortex and striatum. Such small NPs are tiny enough to reduce liver sequestration and cross brain barriers yet large enough to bypass kidney clearance. Adding targeting ligands may promote specific delivery to neurons and microglia, but this will enlarge the NP size and risk liver sequestration. NP surface chemistry may be another key parameter for brain delivery. Lipka et al.^59^ i.v. injected three gold NP types into healthy rats, but they observed inefficient brain delivery. The phosphine-capped gold NP is similarly sized as Au_3_@PEG_1K_ NP (11 nm), but it lacks a PEG coating to extend blood circulation and prevent liver clearance (both factors favoring brain delivery^60^). Au_5_@PEG_750_ NP (21 nm) is larger than our Au_3_@PEG_1K_ NP as its PEG coating contains dodecane and poly­(maleic anhydride), but it is unclear if both coating components cause liver clearance and restrict brain delivery. Au_5_@PEG_10K_ NP (31 nm), while also containing both coating components, showed improved blood circulation and reduced liver uptake due to its longer PEG chains, but its larger NP size disfavors brain delivery. In absolute mass, the accumulation of our Au_3_@PEG_1K_ NP in the HD brain (0.58 μg/g) is orders of magnitude higher than that of Au_5_@PEG_10K_ NP in the healthy brain as reported by Xu et al.,^61^ possibly due to a smaller NP size for prolonged blood circulation, absence of non-PEG coating components, HD pathology that favors brain delivery (Figure 1A), and a ∼200-fold higher gold NP dose to mitigate liver clearance in terms of percent injected dose (%ID) (Figure S76)^62^ and achieve therapeutic efficacy; our total gold dose is similar to that of chiral gold NP for treating Alzheimer’s disease (∼100 mg-gold/kg-mouse).^63^ Recently, the nanomedicine field has advocated for designing NPs with careful consideration of the physiological, anatomical, and molecular traits of the disease site.^64^ We reported NPs to access challenging disease sites by tuning NP size and surface chemistry, say psoriatic skin,^13,65^ fibrotic kidney,^14^ and atherosclerotic plaque.^66^ Others bypassed biological barriers using combinatorial NP libraries for organ-^67^ or cell-specific^68^ delivery. These efforts will enrich our understanding of bionano interactions at diseased sites.

Another major finding is that Au_3_@PEG_1k_ NPs are self-therapeutic agents for HD; no chemical drugs, biologics, or physical forces are needed. Our work adds to the list of applications of gold NP^69^ as treatments, from cancer^70^ to cardiovascular disease.^71^ For neurodegenerative disorders, recent clinical evidence suggested that uncoated gold NPs alleviate Parkinson’s disease (NCT03815916) and amyotrophic lateral sclerosis (NCT04414345) (Figure S2 and Table S2), citing their general anti-inflammatory,^72^ antioxidative,^73^ or surface catalytic^44^ properties as the primary therapeutic mechanism. Here, we provide molecular details of a therapeutic mechanism of PEG-coated gold NP distinct from uncoated gold NP at three levels: the pathway (activation of oxidative phosphorylation) by as revealed by proteomics (Figure 4), therapeutic targets (inhibition of p38 and PDK1) as revealed by kinome profiling (Figure 5), and mode of cell death (inhibition of pyroptosis) (Figures 7 and S77).

While R6/2 is a well characterized model for studying HD pathogenesis over a short time duration, potential limitations such as fragmented mutant HTT exon 1 protein overexpression, accelerated disease course and phenotypic differences from human pathology could affect the translatability of results to humans.^74^ For example, the neuronal inclusions shown in Figure S53 originate from aggregation of fragmented mutant HTT exon 1 proteins, so the ability of gold NP to deaggregate full-length mutant HTT protein will need additional proof. Also, overexpression of HTT N-terminal exon 1 fragments accelerates disease progression, limiting studies on the long-term toxicity of NP. Here, Au_3_@PEG_1k_ NP prolongs the survival of HD mice without toxicity 3-month post-treatment (Week 24), yet we cannot ethically extend the end point beyond Week 24 due to the cumulative death of 75% of the untreated R6/2 mice (Figure 3B). While it is reassuring to witness a 50% reduction in gold NP density in the HD brain by Week 24 (Figure 3C), a longer time duration is required for complete clearance albeit ethically unacceptable. Conducting the same study for a longer time duration (say 1 year) on healthy mice is feasible, but the safety data will differ from those on R6/2 mice because Au_3_@PEG_1k_ NPs enter the HD brain more profusely than healthy brain. Further, neuronal loss (a hallmark of human HD) in R6/2 HD mice is not as pronounced as in HD patients.^12,74^ Therefore, exploration of efficacy in other disease models will improve the translatability of gold NP.^4^ Knock-in mouse models that express endogenous full-length HTT protein with polyQ expansion, such as HdhQ150 ^75^ and CAG140 KI,^76^ will allow for full length HTT protein deaggregation studies, longer-term efficacy and toxicity evaluation up to 24 months,^74^ and a more relevant studies for neuron loss. Further, we may probe the effect of Au_3_@PEG_1k_ NP on HD-induced cognitive deficits (a major feature of HD) using the Morris water maze and two-choice swim tank for learning and memory performance assessment.^77^

It is imperative that the long-term safety of repeated doses of gold NP be comprehensively elucidated in the future. Past researchers reported the long-term safety of PEG-coated gold nanorods in mice 15 months postinjection^78^ and PEG-coated gold nanoshells in mice 404 days postinjection,^79^ but these results involved only a single i.v. injection. Another report demonstrated the safety of Spike/PEG-coated gold NP in hamsters 1-year postinhalation, but there were only three inhalations.^80^ Other researchers also reported the tolerability of PEG-coated gold nanoshells in beagle dogs^79^ and PEG-coated gold NPs in cancer patients as part of a dose-escalating Phase 1 trial,^81^ but there were only two i.v. infusions. Due to concerns over the side effects of gold salt-based therapies,^82^ longer-term toxicity, aggregation, or biotransformation of gold NPs will warrant investigations because gold NPs take 5–6 months for intracellular degradation^83^ or eliciting cellular responses.^84^ Notably, past studies on the safety of gold NP were not entirely consistent.^85^ For example, 1.9 nm gold NP (Aurovist) reduce proliferation of hypoxic breast cancer cells,^86^ and citrate-capped 20 nm gold NP induces autophagy and oxidative stress in lung fibroblasts.^87^ By contrast, our 11 nm Au_3_@PEG_1k_ NP not only is safe to brain endothelial cells and neurons *in vitro* (Figures S8 and S20–S22) but also activates the oxidative phosphorylation pathway in the brain of R6/2 HD mice (Figure 4), ultimately improving motor deficit. Such discrepancies may stem from the specific experimental conditions (*e.g*., dose, exposure time, age, gender, and cell type) and NP properties (*e.g*., size, shape, surface charge, and coating) tested. Therefore, transparent and detailed reporting of experimental parameters^88^ is crucial to enable objective comparisons between various studies and to propel the field of gold nanomedicine forward. Still, when compared to organic NP carriers (liposome and micelle) and drugs (small molecules and biologics), gold NP offers a more convenient chemical handle for tracking its fate in the brain (and the body).

## Materials and Methods

### Animal Models of Huntington’s Disease (HD)

Transgenic B6.CBA-Tg­(HDexon1)­62Gpb/3J­(22) mice (R6/2; Jackson Laboratory) were bred by mating transgenic male mice with F1 female mice. They were maintained in a controlled environment at 22 ± 1 °C and a humidity range of 40–60%, following a 12 h dark/12 h light cycle at the Laboratory Animal Services Centre of The Chinese University of Hong Kong (CUHK). Transgenic female mice of 4–10 weeks old were used for the experiments, whereas transgenic male mice were reserved for mating. Genotyping was performed based on 100 ng of genomic DNA (originally extracted from <2 mm of tail biopsies from each mouse). For the polymerase chain reaction (PCR; ABI PCR System 9700), the forward primer was 5′-CCGCTCAGGTTCTGCTTTTA-3′ and the reverse primer was 5′-TGGAAGGACTTGAGGGACTC-3′. For the internal positive control for genotyping, the forward primer was 5′-CTA GGCCACAGA ATTGAA AGATCT-3′ and the reverse primer was 5′-GTAGGTGGAAATTCTAGCATCATCC-3′. Nontransgenic littermates were used as control subjects. All animal experiments were performed following the protocols evaluated and approved by the CUHK Animal Experimentation Ethics Committee (Ethics Approval Number: 22–317-GRF).

### Efficacy Evaluation by Behavioral Tests^33^

The day before the first evaluation at Week 6, healthy littermates and R6/2 mice were trained on the rotarod apparatus (Panlab) at a constant speed of 4 rpm for at least 1 min. On a given day at Weeks 6, 7, and 9, the mice took the open field test, rested for 1 h, took the rotarod test, rested for 1 h, and finally received an i.v. injection of 0.7 mg of Au_3_@PEG_1k_ NPs (formulated in 0.1 mL of D5W), all during the animal’s light cycle phase. (At Weeks 8 and 10, the mice only received NP treatment but did not take the behavior test.) Equivalently, the average body weight of R6/2 HD mice aged between Weeks 6 and 10 is 25.6 g (over *n* = 42 animals). Per our treatment protocol, each mouse received a weekly i.v. injection of 700 μg of gold NP for 5 consecutive weeks. Therefore, the total dose is 0.7 mg-gold/dose-mouse ÷ 0.0256 kg-mouse × 5 doses = 136 mg/kg. The negative treatment control entailed weekly i.v. injections of 70 kDa dextran (Sangon Biotech, A600375; formulated in 0.1 mL of D5W) from Weeks 6 to 10. The positive treatment control entailed daily i.p injections of tetrabenazine (Sigma; formulated in 0.1 mL of D5W containing 9% DMSO and 0.1 M citric acid^89^) from Weeks 6 to 11. At Week 11, the mice took the last open field test and rotarod test before sacrifice. At sacrifice, after dissecting the brain into CTX and STR on an ice-cooled metal plate within 5 min, the CTX and STR tissues were weighed, snap-frozen in liquid nitrogen, and stored at −80 °C.

In an open field test, each mouse was placed at the center of an activity chamber (44 × 44 × 50 cm^3^) made of white, high-density, nonporous plastic equipped with a video camera (Panlab). The track of the mouse was recorded for 30 min, under low-stress and quiet conditions. Raw video data were analyzed using the Smart 3.0 Video Tracking Software (Panlab). A longer total distance traveled over 30 min indicates improved spontaneous locomotion due to NP treatment.

In a rotarod rest, the mice were placed onto the rotarod, one animal per lane. The rotation speed was initially 4 rpm and then linearly accelerated from 4 to 40 rpm over 5 min. Each mouse was tested five times in a 10 min interval with its staying time on the rotarod recorded, and the maximum and minimum staying time durations were removed before data analysis. A prolonged staying time on the rotarod indicates improved motor behavior due to NP treatment.

### Therapeutic Role of Kinases

#### Part 1: Kinase Panel Screening and IC_50_

The effect of Au_3_@PEG_1k_ NPs on the activity of various kinases was assessed by SelectScreen Kinase Profiling Service (Thermo Fisher Scientific). The concentration of NP stock solution (20 μM; 100×) was verified by ICP-MS, followed by dialyzing NPs in water against absolute DMSO. Negative control 70 kDa dextran NPs were also prepared to be 20 μM (100×) in absolute DMSO. NP samples were sent for testing using the Z’LYTE biochemical assay. Briefly, Z’LYTE uses a fluorescence-based, coupled-enzyme format based on the differential sensitivity of phosphorylated and nonphosphorylated peptides to proteolytic cleavage. The NP sample was screened in 1% DMSO (final) in the well at a single concentration of 200 nM (1×). To calculate the IC_50_ value of a screened high-performing kinase (with at least 85% inhibitory activity), the NP sample was subject to 10-point, 3-fold serial dilutions from the starting NP concentration of 200 nM (1×) for constructing the dose–response curve. Assays were conducted using the ATP concentration as indicated in the corresponding tables, depending on the format of detection. For “Direct Format” that operates through phosphorylation and activation of a synthetic peptide substrate for a given kinase, [ATP] was set at its apparent *K*_m_ value (*K*_m_, apparent), as previously determined by the Z’-LYTE assay. For “Cascade Format” that operates through phosphorylation and activation of the inactive downstream kinase of a given kinase, [ATP] was set at 100 μM. Percent phosphorylation (% Pho) was determined with reference to the 0% phosphorylation (or 100% inhibition) control (which contains no ATP and therefore exhibits no kinase activity) and the 100% phosphorylation control (which contains the phosphorylated peptide as the same sequence as the peptide substrate). Control wells do not include any kinase inhibitors. Percent inhibition values were calculated by this equation: % Inhibition = [1 – % PhoNP/%Pho0% inhibition ctrl] × 100, whereby the 0% inhibition control contains the active kinase. IC_50_ values were fitted from the dose–response curves based on model number 205 of XLfit from IDBS.

#### Part 2: Roles of p38α and Pyruvate Dehydrogenase Kinase 1 (PDK1)

To test the therapeutic role of selected kinases, R6/2 mice received daily i.p. injections of either (i) 0.1 mL of a vehicle (10% DMSO, 25% PEG_400_ and 5% dextrose) containing an inhibitor of p38α called “p38-α MAPK-IN-1” (MedChemExpress) at a dose of 2.6 mg/kg, or (ii) 0.1 mL of D5W containing an inhibitor of PDK1, sodium dichloroacetate (DCA; Sigma, 347795) at a dose of 100 mg/kg. At Weeks 6, 7, and 9, R6/2 mice underwent the open field test and rotarod test as described above. At the age of Week 11, mice took the open field test and rotarod test again before sacrifice. The excised brain was dissected and processed for Western blot analysis as described above.

### Effect of NP Treatment on Protein Expression in the HD Brain

#### Part 1: Protein Extraction and Digestion

Untreated and Au_3_@PEG_1k_ NP-treated R6/2 mice were sacrificed and perfused with 20 mL of PBS. Half of the brain was harvested, snap frozen in liquid nitrogen, stored at −80 °C, and later sent to Beijing Biotech-Pack Scientific for protein extraction, protein digestion, Tandem Mass Tag (TMT) labeling, peptide fractionation, and analysis by liquid chromatography tandem mass spectrometry (LC-MS/MS; Thermo Fisher Scientific) and bioinformatics. Then, after adding 100 μL of RIPA lysis buffer to the sample, the sample was sonicated on ice, with 3 s of sonication and 3 s of pause, for a total of 5 min. After centrifuging the mixture at 10,000*g* for 10 min at 4 °C, the supernatant was transferred to a prechilled 1.5 mL microcentrifuge tube. Next, 50 μg of the sample was transferred to a new microcentrifuge tube and the volume was adjusted to 100 μL with 50 mM tetraethylammonium bromide (TEAB; Thermo Fisher Scientific). After adding 6 μL of 200 mM tris­(2-carboxyethyl)­phosphine (TCEP; Thermo Fisher Scientific), the sample was incubated at 56 °C for 1 h. After adding 6 μL of 375 mM iodoacetamide, the sample was incubated in the dark at RT for 30 min, mixed with six times the volume of precooled (−20 °C) acetone, and frozen at −20 °C overnight to precipitate out the proteins. After centrifugation at 10,000*g* at 4 °C for 10 min and discarding the acetone, the white precipitate (extracted protein) was air-dried for 2–3 min and resuspended in 50 μL of 50 mM TEAB. Lastly, to enzymatically digest the proteins, 1 μg of trypsin was added and the protein sample was incubated overnight at 37 °C.

#### Part 2: Tandem Mass Tag (TMT) Labeling and Peptide Fractionation

At RT, 20 μL of anhydrous acetonitrile (ACN; Fisher Chemical) was added to each vial of TMT labeling reagent (Thermo Fisher Scientific) and dissolved for 5 min. After adding the TMT solution to the digested protein solution from Part 1, the reaction was incubated at RT for 1 h and later quenched by adding 1 μL of 5% hydroxylamine incubation for 15 min. Equal amounts of the labeled samples were combined in a new microcentrifuge tube and dried under vacuum at 45 °C. For peptide fractionation, the protective white tip from the bottom of a chromatography column was removed, and the column was placed in a 2 mL sample tube, centrifuged at 5000*g* for 2 min to pack the resin (with liquid discarded), rinsed twice with 300 μL of ACN (with centrifugation to discard the ACN), and equilibrated twice with 0.1% trifluoroacetic acid (TFA; Thermo Fisher Scientific). The labeled protein sample was dissolved in 300 μL of 0.1% TFA, loaded onto the column, and centrifuged at 3000*g* for 2 min. To elute the protein, the column was added 300 μL of 5% ACN–0.1% TEA and centrifuged at 3000*g* for 2 min to remove unreacted TMT. The elusion steps continued using sequentially higher concentrations of ACN 10%, 12.5%, 15%, 17.5%, 20%, 22.5%, 25% and 50%, followed by a similar centrifugation step. Each eluted sample was evaporated using a vacuum concentrator until dry.

#### Part 3: LC-MS/MS and Data Analysis

LC-MS/MS entailed the use of a 50 μm i.d. × 150 mm column, packed with Acclaim PepMap Reversed-Phase Liquid Chromatography (RPLC; C18, 1.9 μm, 100 Å). The mobile phases A and B were 0.1% formic acid in water and 0.1% formic acid and 80% ACN, respectively. The total flow rate was 300 nL/min, and a 120 min gradient involved 4–10% B for 5 min; 10–22% B for 80 min; 22–40% B for 25 min; 40–95% B for 1 min; and 95–95% B for 9 min. All MS and MS/MS spectra were acquired in data-dependent acquisition mode, and the full mass scan was acquired at 350–1500 *m*/*z* with a resolution of 60,000. Raw data were analyzed based on the species of the samples using Proteome Discover 2.5. Identification of differentially expressed proteins (DEPs) was achieved by comparing the “Au_3_@PEG_1k_ NP-treated R6/2 mice” group to the “untreated R6/2” mice group. Proteins with a fold change ≥ 1.5 (*P* < 0.05) were considered upregulated, while proteins with a fold change ≤ 1/1.5 (*P* < 0.05) were considered downregulated. Pathway mapping of DEPs was performed using the Kyoto Encyclopedia of Genes and Genomes (KEGG) database (http://www.genome.jp/kegg/). Distribution of proteomics data in biological processes, cellular components, and molecular functions was obtained by mapping to the Gene Ontology database (https://geneontology.org).

### Sample Size and Study Design

All animal procedures followed the guidelines stipulated by the Animal Experimentation Ethics Committee (AEEC) at The Chinese University of Hong Kong (CUHK). To ensure statistical power, at least three replicates and repeats were used in all *in vitro* experiments. For behavior tests, we used the Dunnett’s test to deduce the required size of each treatment group (*N*). Dunnett’s test is a multiple comparison procedure that compares the efficacy of each treatment group with the same control group. Here, we tested “H_0_: All treatment groups are equivalent to the control group” against “H_1_: There exists one group that is superior to the control group.” We compared the treatment groups and the control group in a way that (i) the chance of committing type 1 error is <5% and that (ii) our comparison is of power 80%. Dunnett’s formalism states that *p* = √*N*·δ/σ, where *p* is the correlation coefficient that depends on *N*. There are three treatment groups (Au_3_@PEG_1k_ NP, tetrabenazine, and 70 kDa dextran) and a control group (*i.e*., untreated), so p is 4.3. If the superior treatment group gives an outcome (δ) of 1.5 standard deviation (SD; σ) better than the control group, the required *N* is at least (4.3/1.5)^2^ = 8.22 ≈ 8. R6/2 HD mice were randomly assigned to each group. For mechanistic studies, at least *n* = 4 was used. Toxicity studies were conducted with 3–4 independent biological replicates. Researchers were not blinded to the identity of the analysis of histological images, nor were they blinded to the identity of animals in behavioral studies. Blinded approach was used for blood chemistry tests.

### Data Processing and Statistical Analysis

The Prism (GraphPad Software) software and ImageJ were used for data analysis and graph construction. Statistical analysis was indicated under the figures. To determine the statistical significance in the comparison of two groups, an unpaired two–tail *t* test was performed. To determine the statistical significance in the comparison of multiple groups, an unpaired one–way analysis of variance (ANOVA) was performed with Tukey’s Test for post hoc analysis. To determine the statistical significance in the comparison of multiple groups with 2 variables (*e.g*., NP treatment and time), two–way ANOVA with Šidák Test (2 groups) or Tukey’s Test (>2 groups) for post hoc analysis. Log-rank Mantel–Cox test was used for survival curve. Normality of sampling distribution of means was validated by Shapiro–Wilk test. Homogeneity of variance was validated by Bartlett’s test. Results are considered significant at *P* < 0.05.

## Supplementary Material







## Notes

Additional detailed materials and methods, background of nanomedicine and gold NP for HD, NP synthesis and characterization, *in vitro* cellular uptake, organ/tissue-level distribution and NP stability, mechanism of brain transport, presence of NP in the brain parenchyma, efficacy of motor improvement, toxicology, proteomics analysis, kinome profiling and *in vivo* validation, alleviation of neuroinflammation upon NP treatment (PDF)
Reconstructed 3D confocal reflectance image of a brain cryosection of Au_3_@PEG_1k_ NP-treated R6/2 mice. White arrow points to the gold NPs inside NeuN^+^ zone (red). Blue: nucleus (DAPI); Green: gold NP (green) (Au) (Video S1) (MP4)
Reconstructed 3D confocal reflectance image of a brain cryosection of untreated R6/2 mice. Blue: nucleus (DAPI); Red: neuron (NeuN) (Ctrl) (Video S2) (MP4)
