# Supplementary material for: Small Gold Nanoparticles Alleviate Huntington’s Disease via Modulating p38α Mitogen-Activated Protein Kinase and Pyruvate Dehydrogenase Kinase 1
Source: ACS Nano. 2025 Dec 22;20(1):683–99. doi: 10.1021/acsnano.5c14751 (PMC12810479; doi:10.1021/acsnano.5c14751)
Supplement: Supplementary file 1 [file nn5c14751_si_001.pdf]

**Supporting information**

# Small gold nanoparticles alleviate Huntington's disease via modulating p38α mitogen-activated protein kinase and pyruvate dehydrogenase kinase 1

*Leo Kit Cheung Lee[1], Lok I Leong[2], Moldir Shyngys[1], Qianqian Bai[1], Ying Lam Lui[2], Can Cui[3], Shaorui Liu[1,4], Yu Xiao[1], Cecilia Ka Wing Chan[1,4], Wing-Hoi Cheung[3], Kin Ming Kwan[2], Ho Yin Edwin Chan[2], Chung Hang Jonathan Choi[1,2,5*]*

[1]Department of Biomedical Engineering, [2]School of Life Sciences, [3]Musculoskeletal Research Laboratory, Department of Orthopedics and Traumatology, [4]Department of Surgery, The Chinese University of Hong Kong, Shatin, New Territories, Hong Kong. [5]Center for Neuromusculoskeletal Restorative Medicine, Hong Kong Science Park, Shatin, New Territories, Hong Kong.

*Corresponding author: Chung Hang Jonathan Choi: jchchoi@cuhk.edu.hk.

**Table of content**

**Materials and Methods** 3

Preparation of $Au_x@PEG_y$ NPs 3

Physicochemical characterization of NPs 3

Quantification of PEG loading on the gold NP 4

In vitro cellular uptake 6

*In vitro* model for measuring active cellular transport 7

*In vivo* organ-level distribution of NPs 9

*In vivo* intracranial tissue-level distribution of NPs 9

Entry of NPs to the HD brain 11

Efficacy and mechanistic evaluation by imaging and molecular assays 13

*In vivo* toxicity 15

Ex vivo skeletal muscle and neuromuscular junction (NMJ) functional test 16

Skeletal muscle mass measurement 17

**SI Data** 18

Appendix I: Background 18

Appendix II: Nanoparticle Characterization 33

Appendix III: In Vitro Cellular Uptake 37

Appendix IV: Organ/Tissue-level Distribution and Nanoparticle Stability 40

Appendix V: Transport Mechanism of the Nanoparticle 46

Appendix VI: Nanoparticle in the Brain Parenchyma 50

Appendix VII: Efficacy 72

Appendix VIII: Toxicology 78

Appendix IX: Proteomics 90

Appendix X: Kinome Profiling and Validation 98

Appendix XI: Neuroinflammation 101

Appendix XII: Others 102

**SI References** 104

## Materials and Methods

### Preparation of $Au_x@PEG_y$ NPs

Citrate-capped gold nanoparticles (Au NPs) of 3 nm in diameter were synthesized as described previously [1]. Briefly, 150 mL of freshly prepared sodium citrate (2.2 mM) containing 0.1 mL of tannic acid (2.5 mM, Sigma) and 1 mL of potassium carbonate ($K_2CO_3$, 150 mM; Sigma) was heated in a three-necked round bottom flask under vigorous stirring. When the temperature reached 70 °C, 1 mL of $HAuCl_4$ (25 mM; Sigma) was injected and the reaction mixture was stirred for 5 min. The resultant 3 nm Au NP solution was stored at 4 °C. Gold NPs of 13 nm in size were synthesized based on a modified Frens' method[2]. After bringing 50 mL of $HAuCl_4$ (1 mM) to a boil, 5 mL of sodium citrate (1% w/v) (Alfa Aesar) was added under vigorous stirring and kept boiling for 15 min. The product was slowly cooled down to room temperature (RT). For both sizes, citrate-capped Au NPs were functionalized with thiolated PEG ($PEG_{1k}$-SH, BiochemPEG) at a concentration of 10 PEG molecules per $nm^2$ of NP surface under stirring overnight. To remove free PEG strands, NPs were washed three times by centrifugation at 2000 × g for 5 min, using centrifugal filters with a membrane size cutoff of 50 kDa (Merck Millipore).

### Physicochemical characterization of NPs

Concentration of Au NPs was determined by inductive coupled-plasma mass spectrometry (ICP-MS) (Agilent 7900) with reference to a standard curve of known gold concentration (Au 197 isotope) in parts per billion (ppb). To obtain an equivalent mass of gold in the NP solution for injection into animals and to quantify the gold contents in tissues and cells, we first converted the ICP-MS raw data from ppb to μg/L using the equation 1 ppb = 1 μg/L, followed by multiplying the volume of dilute 2% nitric acid ($HNO_3$) that contains the digested tissues.

To characterize the morphology and measure the physical diameter of the entire PEG-coated gold NP (Au core + PEG), the NPs were negatively stained for TEM imaging. In brief, 10 μL of aqueous NP solution was dropped onto a plasma-treated (Harrick Plasma), formvar/carbon-coated copper grid (200 mesh; Beijing Zhongjingkeyi Technology) and left for 30 min. Then, the NP droplet was drawn off from the edge of the grid with filter paper. Next, EM Stainer solution[3] (Nisshin-EM; catalog no.: 336) was diluted with Nanopure water twice, and 10 μL of the diluted solution was added to each TEM grid for another 10 min. (EM Stainer is an electronic stain alternative to uranyl acetate.) After removing the EM Stainer solution, the grid was allowed to dry at RT for at least 4

h before visualization under TEM at 100 kV (Hitachi H7700) and magnification of 30,000–50,000. At higher magnifications, the PEG shell does not have enough contrast to be imaged, especially for $Au_3@PEG_{1k}$ NP with a smaller gold core. At lower magnifications, the entire $Au_3@PEG_{1k}$ NP appears too small to be imaged. Then, the length measuring function of the imaging software installed on-site at the Hitachi TEM instrument was used to obtain an initial measurement of physical size. After this confirmatory step, more TEM images of a given NP sample were taken and analyzed using the Fiji image processing program as part of the ImageJ software. The physical diameters of the gold core and the PEG-coated gold NP were measured by manually drawing a straight line through the center of the NP from one end of the stained PEG shell circumference to another. Measurements were taken only where staining boundaries were clearly defined. At least 500 NPs for each NP type were counted.

The hydrodynamic diameters and zeta potential of NPs were measured by the DelsaMax PRO dynamic light scattering (DLS) analyzer (Beckman Coulter). Reported DLS data represent the values from three independent measurements. The DLS data quality was interpreted by analyzing the autocorrelation function and its fit, where the sum of square (SOS) threshold was set to be under 100. (Only measurements with an SOS value <100 were counted). As independent samples were used for different assays or experiments, a slight batch-to-batch variation was observed. To test colloidal stability, 50 µL of NP solutions were mixed with 2 mL of artificial cerebrospinal fluid (CSF) buffer (128 mM NaCl, 1.9 mM KCl, 1.2 mM $KH_2PO_4$, 26 mM $NaHCO_3$, 0.85 mM $CaCl_2$, 6.5 mM $MgSO_4$, and 10 mM glucose; pH 7.4±0.05)[4] or 50% fetal bovine serum (FBS), and incubated at 37°C for 24 h, followed by analysis using UV-vis spectrometry and DLS. Reported DLS values represent mean ± SD from three independent measurements. Further, NPs were intravenously (i.v.) injected into R6/2 mice at the age of Week 10. After sacrificing the mice 24 h post-injection, ~0.5 mL of blood plasma was collected via an intracardiac puncture using an ethylenediaminetetraacetic acid (EDTA) tube (Sarstedt) and then centrifuged at 2000 × g for 10 min. The Au NPs in the plasma supernatant were analyzed for their stability using DLS.

**Quantification of PEG loading on the gold NP**

*Method I: Indirect measurement of excess thiolated PEG strands unattached to the gold core during synthesis of $Au_x@PEG_y$ NP*

Unmodified $Au_x$ NPs were functionalized with $PEG_y$-SH at a concentration of 10 PEG molecules per $nm^2$ of NP surface under stirring overnight. The as-synthesized $Au_x$@$PEG_y$ NPs were collected without washing to retain the unbound $PEG_y$-SH strands. To collect the free PEG strands, 10 mL of PEG standard and NPs were subjected to centrifugation at 2000 × g for 5 min, using centrifugal filters with a membrane size cutoff of 50 kDa (Merck Millipore). PEG was detected as previously reported.[5] Briefly, 5 mL of the filtrate was collected and concentrated by freeze drying for 5 times. 20 µL of the concentrated PEG sample or standard was mixed with 100 µL of Ellman's assay buffer [1 mM ethylenediaminetetraacetic acid (EDTA; Sigma) in 0.1 M $Na_2HPO_4$ (pH 8)]. The resultant PEG sample or standard was mixed with 50 µL of Ellman's detection buffer [2 mg/mL Ellman's reagent 5,5-dithio-bis(2-nitrobenzoic acid) (JenKem Technology) in the assay buffer]. After incubation at RT for 10 min, the absorbance was measured at 412 nm by the SpectraMax M3 microplate reader (Molecular Devices). Subtraction of the amount of unbound PEG-SH strands from the known total amount of PEG-SH strands initially added to the unmodified gold cores will infer the loading of PEG on the gold core. PEG loading per NP was calculated by dividing the concentration of $PEG_y$-SH attached to the gold core by the concentration of gold NPs (as determined by ICP-MS).

*Method II: Thermogravimetric analysis (TGA)*

A concentrated solution of $Au_x$@$PEG_y$ NP in Nanopure water was dried in an open alumina crucible at 80 °C for at least 2 h. Drying continued until the dried NP material on the crucible was at least 1 mg in weight. The sample was analyzed by PerkinElmer TGA6, with a heating rate of 10 °C/min from RT to 650 °C and nitrogen gas purging at a flow rate of 20 mL/min. The organic component of the NP (PEG) was found from the percent mass loss over the temperature range of 100–500°C, leaving behind gold (inorganic component) in the crucible after heating. Loading of PEG strands on the gold core ($\sigma_{TGA}$) was calculated using the following equation[6]:

$$\sigma_{TGA} = \frac{\frac{wt\%_{shell}}{wt\%_{core}} \rho_{core} \frac{4}{3} \pi r_{core}^3 N_A}{MW 4 \pi r_{core}^2}$$

$\sigma_{TGA}$: Loading of PEG strands on gold NP. Unit: Number of strands per surface area.

$wt\%_{shell}$: Relative mass of PEG determined from TGA data.

$wt\%_{gold}$: Relative mass of gold determined from TGA data.

$\rho_{core}$: Density of gold = 19.6 g/cm$^3$.

$r_{core}$: Radius of gold core.

$N_A$: Avogadro constant.

MW: Molecular weight of PEG strand.

**In vitro cellular uptake**

*Cell line*

SK-N-MC cells or bEnd.3 cells (American Type Culture Collection; ATCC) were maintained in a humidified cell culture incubator supplemented with 5% $CO_2$ at 37 °C. For SK-N-MC cells, seeded in 24-well plates and grown till 70–80% confluence, the cells in each well were treated with 0.3 mL of transfection medium and incubated for 24 h. The transfection medium contains 2 µL of Lipofectamine 2000 (Invitrogen) and 1 µg of plasmid DNA (pEGFP-Htt1-550$_{CAG89}$) containing 89 repeating units of glutamine (Q89) expressing codons as reported previously[7], formulated in complete Dulbecco's modified eagle medium (DMEM; Gibco) supplemented with 10% fetal bovine serum (FBS; Gibco), 1% penicillin–streptomycin (P/S; Gibco).

*Part 1: Cellular uptake by ICP-MS*

Both SK-N-MC and bEnd.3 cells were incubated with 0.5 mL of 200 nM $Au_3$@$PEG_{1k}$ NPs (in complete culture medium) for 24 h. After that, cells were trypsinized with 200 µL of 0.25% trypsin-EDTA (Gibco) at 37 °C for 5 min, with 20 µL of the trypsinized cells used for staining with trypan blue (Gibco) and counting. The remaining 180 µL of trypsinized cells was dried in an oven at 60°C overnight, digested with 0.5 mL of aqua regia [3:1 v/v ratio of 38% HCl (RCI Labscan) and 68% $HNO_3$ (VMR Chemical)] for 1 h, and diluted to a 2% HCl, 2% $HNO_3$ solution with Nanopure water. Calibration standards of known gold concentration were prepared to convert counts of gold ions to known gold concentrations for ICP-MS measurements.

To confirm cell viability upon cellular entry, cells were incubated with 200 nM of $Au_3$@$PEG_{1k}$ NPs in DMEM with 10% FBS. The lactate dehydrogenase (LDH) assay was performed on untransfected bEnd.3 cells or 24 h post-transfection of SK-N-MC cells, per the manufacturer's instructions (CytoTox 96 Non-Radioactive Cytotoxicity Assay Kit, Promega). LDH is a cytosolic enzyme expressed in eukaryotic cells, released to cell culture medium when cell membrane is damaged by cytotoxic insults. The LDH assay measures LDH activity[8], directly proportional to the number of dead/damaged cells.

*Part 2: Cellular uptake by confocal microscopy*

Seeded on 35-mm confocal dishes or 24-well plates and grown in till 70–80% confluence, cells were incubated with 0.5 mL of 50 µg/mL $Au_x$@$PEG_y$ NP for 24 h. After five rinses with phosphate-

buffered saline (PBS; pH 7.2; 5 min each), cells were fixed with 0.5 mL of 4% paraformaldehyde (PFA; Sigma) overnight, rinsed with PBS for three times, stained with 0.5 mL of 4',6-diamidino-2-phenylindole (DAPI; Invitrogen) in PBS (5 µg/mL) for 30 min, washed with 1 mL of Nanopure water for five times, and finally stained using the LI Silver (LIS) Enhancement Kit (Invitrogen). After mixing the silver enhancement solutions, Solution A (silver salt) and Solution B (initiator) at a 1:1 ratio right before use, the cells were incubated with 300 µL of the mixture in each well, incubated at RT for 30 min, rinsed with Nanopure water, and mounted on a glass slide. Confocal images were acquired in reflection mode with a 40× objective under 670 nm excitation[9].

***In vitro* model for measuring active cellular transport**

The key objective is not to report the absolute mass of gold NP penetrating the BBB *in vitro* model, because some *in vitro* BBB models may have issues over tightness between paracellular gaps. Rather, we wish to ascertain the relative importance of active cellular transport to the level of BBB penetration *in vitro*. Here, we included (i) an experimental group in which cells were treated with both NPs and an inhibitor of a certain cellular uptake pathway and (ii) a control group in which cells were treated with NPs but not the inhibitor. Dividing the difference in gold content between both groups by the gold content of Group (ii) yields a ratio for quantifying the role of active cellular transport without the confounding factor of passive leakage through paracellular gaps that might bias data interpretation.

*Model 1: Transwell model of bEnd.3 cells*

Mouse bEnd.3 cell-based Transwell BBB model is commonly utilized in the field of brain nanomedicine[10,11]. 5 x $10^4$ bEnd.3 cells were seeded in each 24-well Transwell insert (0.3 $cm^2$; Falcon, 353095) and cultured in 0.5 mL of complete culture medium in the upper chamber and 1 mL of complete culture medium in the lower chamber for 7–14 days until the TEER value of the bEnd.3 endothelium exceeded 30 $\Omega \cdot cm^3$ [as measured by EVOM2 (World Precision Instruments)] [12]. Next, cells seeded in the upper chamber were pretreated with 300 µL of complete culture medium that contains different concentrations of chemical blockers for 1 h[13,14]. These blockers include filipin (10 µg/mL [15], Sigma-Aldrich), dynasore (13 µg/mL, Cayman Chemical), chlorpromazine (5 µg/mL, Tokyo Chemical Industry), amiloride (500 µg/mL, Sigma-Aldrich), and sodium azide (3.3 mg/mL, Sigma-Aldrich). After removing the inhibitor-containing medium, 300 µL of medium that contains the same inhibitor at the original concentration and 400 µg/mL

$Au_3@PEG_{1k}$ NPs were added to the cells. After 3 h, the medium in the lower chamber were collected for ICP-MS analysis while cells and medium in the upper chamber were collected for measuring cytotoxicity using the CytoTox 96 Non-Radioactive Cytotoxicity Assay Kit (Promega).

*Model 2: Transwell model of hCMEC/D3 cells*

Human hCMEC/D3 cell-based Transwell BBB model is commonly utilized in the field of brain nanomedicine[11,16]. Here, the protocols were similar to those from Model 1 except for two points. The complete culture medium was switched to Human Large Vessel Endothelial Cell Basal Medium (formerly Medium 200; Thermo Fisher) supplemented with low serum growth supplement (Thermo Fisher), and the Transwell insert was coated with collagen Type I at an areal density of 5 $\mu g/cm^2$ (Thermo Fisher). Cell culture continued for 7–14 days until the TEER value of the hCMEC/D3 endothelium exceeded 30 $\Omega \cdot cm^3$ [16]. For the inhibition studies, the same protocols were largely similar to those from Model 1 except the blockers included filipin and sodium azide. The same cytotoxicity studies also followed those from Model 1.

*Model 3: Integrated biomimetic array chip (IBAC) M1 of brain endothelium seeded with H9 embryonic stem cell (ESC)-induced brain microvascular endothelial cells (iBMECs)*

An advanced BBB *in vitro* model, with enhanced tightness over bEnd.3 and hCMEC/D3 cells, was utilized for evaluating the active transport of NPs across the BBB. H9 ESCs (Cellapy) were differentiated into iBMECs per the internal standard operating procedures of Daxiang Biotech. Next, the iBMECs were seeded below the porous membrane (0.135 $cm^2$) in an IBAC M1 array chip (Daxiang Biotech), a gravity-driven, fluid-flow, pumpless, high-throughput chip (**Figure S22**) for constructing single organ models with barrier functions, *e.g.*, BBB[17]. The iBMEC-seeded chip was kept under gentle shaking (MR100110, Daxiang) in a humidified 5% $CO_2$ incubator at 37 °C until the TEER value of the iBMEC endothelium exceeded 200 $\Omega \cdot cm^3$ to ensure the formation of tight junctions. TEER was measured using electrode probes (MT100111, Daxiang Biotech) of the resistance meter (MT100110, Daxiang Biotech). 0.2 mL of PSC-induced brain microvascular endothelial cell maturation B medium (LS100123, Daxiang Biotech), containing $Au_3@PEG_{1k}$ NP and inhibitor at the same concentrations as Model 1, was continuously flowing in the reservoir of the IBAC M1 array chip for 3 h. The blockers included filipin and sodium azide. After that, the medium above the porous membrane (organoid chamber; 0.1 mL) was collected for ICP-MS analysis, while the cells and medium in the reservoir were collected for cytotoxicity tests.

## *In vivo* organ-level distribution of NPs

*Part 1: Distribution of NPs as a function of disease stage*

R6/2 mice and healthy littermates of different ages (Week 4, 6, 8, or 10) received a single i.v. injection of 0.7 mg of $Au_x$@$PEG_{1k}$ NPs [formulated in 0.1 mL of 5% dextrose in water (D5W)]. As $Au_x$@$PEG_y$ NPs have different sizes, the equivalent number of NPs per injected dose was different for each size, $1.76 \times 10^{15}$ NPs for $Au_3$@$PEG_{1k}$ NP and $3.38 \times 10^{13}$ NPs for $Au_{13}$@$PEG_{1k}$ NP. After sacrificing the mice 24 h post-injection, the brain and other organs or tissues (*e.g.*, muscles) were extracted and digested with 1 mL of aqua regia for 4 d at RT, unless otherwise stated. (Whole livers were digested with 3 mL of aqua regia). The digested samples were diluted to a 2% HCl, 2% $HNO_3$ solution with Nanopure water and then passed through a 0.1 μm acid-resistant mixed cellulose ester (MCE; cellulose nitrate and cellulose acetate) filter using a 10-mL syringe. As R6/2 brains became progressively smaller with more severe neurodegeneration[7], the amount of gold in the brain was normalized to μg of gold per gram of tissue (μg/g) to eliminate bias due to the difference in tissue mass. On blood pharmacokinetics, Week 10 R6/2 mice were i.v. injected with 0.1 mL of D5W containing 0.7 mg of $Au_x$@$PEG_y$ NPs. At various time points post-injection, 5 μL of blood was drawn by the tail vein using a 27 G needle and digested in 1 mL of aqua regia for ICP-MS measurements.

*Part 2: Distribution of NPs at efficacy evaluation*

Healthy littermates and R6/2 mice received weekly i.v. injections of 0.1 mL of D5W containing 0.7 mg of $Au_3$@$PEG_{1k}$ NPs from the age of Week 6 to 10. At sacrifice (Week 11), tissues were harvested, weighted, and digested with aqua regia for ICP-MS measurements.

*Part 3: Long-term clearance*

The procedures largely followed Part 2, except that the mice were housed for 3 additional months and sacrificed at Week 24, the terminal age for sacrifice per LASEC guidance.

## *In vivo* intracranial tissue-level distribution of NPs

*Part 1: Distribution as a function of disease stage*

R6/2 mice and healthy littermates of different ages (Week 4, 6, 8, or 10) received a single i.v. injection of 0.7 mg of $Au_x$@$PEG_{1k}$ NPs (formulated in 0.1 mL of D5W). After sacrificing the mice 24 h post-injection, one half of the brain was dissected into five different parts [cortex (CTX),

striatum (STR), hippocampus (HP), cerebellum (CB), and other regions (Others)] as published[18] and digested in 1 mL of aqua regia for ICP-MS analysis as above.

To visualize the intracranial location of gold NPs, we employed confocal immunofluorescence to label different cell types and confocal reflectance imaging to track the gold NP. Images of silver-enhanced coronal brain tissue sections were obtained using a Leica TCS SP8 confocal microscope using a 40× objective, overlayed with other fluorescence channels. Identification of the CTX and STR regions was based on the Allen Brain Atlas (http://mouse.brain-map.org/). Where necessary, large brain confocal images were obtained by stitching adjoint images taken with a 40× objective using the LAS X software.

(i) Immunofluorescence: PBS-perfused brains were harvested, fixed in 10% formalin (3.7% w/v) for 24 h, and exchanged with 15% sucrose (w/v) in PBS at 4 ºC until the brains sank. They were frozen in Shandon Cryomatrix frozen embedding medium (Thermo Fisher Scientific) and cut into sections of 40 μm thick. After floating into PBS-containing 6 well plates, the cryosections were rinsed for three times (5 min each) and immersed in 1–1.5 mL of storage solution [30% (w/v) sucrose (TCI) and 30% (w/v), 30% (v/v) ethylene glycol (sigma) in 0.1 M phosphate buffer][19] for storage at –80 °C. Using the floating technique, cryosections were brought to RT, washed with PBS for three times, blocked with 3% bovine serum albumin (BSA, Rockland) with 0.3% Triton X-100 (Thermo Fisher Scientific) in PBS for 1 h, and stained with primary antibodies in blocking buffer at 4 °C overnight. The primary antibodies include rat monoclonal against CD31 (Invitrogen, 14-0311-82; 1 μg/mL), guinea pig polyclonal against NeuN (Sigma-Aldrich, ABN90; 1:1000) rabbit polyclonal antibody against ionized calcium-binding adaptor molecule 1 (iba1; Fujifilm, 019-19741; 1 μg/mL), mouse monoclonal against HD exon-1 67Q aggregate (MW8; DSHB, AB_528297; 1.25 ng/mL), and rabbit polyclonal against zonula occludens-1 (ZO-1; 61-7300, Invitrogen; 1.25 μg/mL). After rinses with PBS, the sections were stained with secondary antibodies at RT for 1 h: Alexa Fluor (AF) 488-conjugated goat anti-guinea pig (Invitrogen, A-11073; 5 μg/mL), AF546-conjugated goat anti-rabbit (Invitrogen, A-11035; 5 μg/mL), AF633-conjugated goat anti-mouse (Invitrogen, A-21052; 5 μg/mL), or AF647-conjugated goat anti-rat (Invitrogen, A-21247; 5 μg/mL). Slides were stained by 0.5 mL of 1 μg/mL DAPI for 10 min at RT, mounted with antifade mounting medium (Phygene, PH0429), and visualized under a confocal laser scanning microscope at identical imaging settings, in reflection mode for detecting Au NP reflectance. The excitation wavelengths of DAPI, AF488, AF546, AF633 are 405, 499, 561, 631

nm, respectively. The corresponding emission wavelengths are 415−470, 506−600, 571−630, and 640−740 nm, respectively.

(ii) Reflectance imaging: After three rinses with Nanopure water (5 min each) in 12-well plates, the cryosections were stained by the LI Silver (LIS) Enhancement Kit (Invitrogen). See details procedures in Part 2 *in vitro* cellular uptake. Confocal images were acquired in reflection mode with a 40× objective under 670 nm excitation[9]. For 3D confocal imaging, a 63× objective was used, and the Leica SP8 LAS X software was used to construct the videos.

*Part 2: Distribution of NPs at efficacy evaluation*

Healthy littermates and R6/2 mice received weekly i.v. injections of 0.1 mL of D5W containing 0.7 mg of $Au_3$@$PEG_{1k}$ NPs from the age of Week 6 to 10. At sacrifice (Week 11), the brain was dissected for ICP-MS analysis and confocal imaging as stated in the preceding paragraph (Part 1).

*Part 3: Long-term clearance*

The procedures largely followed Part 2, except that the sacrifice point was at the age of Week 24.

**Entry of NPs to the HD brain**

*Part 1: Passive diffusion*

Week 10 healthy littermates or R6/2 mice received an i.v. injection of 150 µL of 2% (w/v) Evans blue (Sigma). After sacrificing the mice 30 min post-injection, the mice were perfused with PBS via an intracardiac puncture using a 29G needle. Next, the brain was homogenized in 300 µL of lysis buffer containing 25% trichloroacetic acid (TCA; Sigma) in PBS. After incubating the brain lysate or a standard (2% Evans blue in lysis buffer) without tissue homogenate at 4 ºC overnight, the samples were centrifuged at 1000 × g at 4 ºC for 30 min. The fluorescence of the supernatant (and standards) was measured by SpectraMax M3 (Molecular Devices) using the excitation and emission wavelengths of 620 nm and 680 nm, respectively. As R6/2 brains became progressively smaller with more severe neurodegeneration[7], the fluorescence in the brain was normalized to arbitrary units (AU) per gram of tissue to eliminate bias due to the difference in tissue mass.

*Part 2: Active cellular uptake*

Week 10 R6/2 mice received an i.v. injection of 20 µL of dimethyl sulfoxide (DMSO; Sigma) containing 25 µg of filipin (Macklin, ~1 mg/kg) via the tail vein. After 1 min, 0.1 mL of D5W containing 0.7 mg of $Au_3$@$PEG_{1k}$ NPs was i.v. injected into the other tail vein. After sacrificing

the mice 24 h post-injection, the brain was dissected into tissue compartments and processed for ICP-MS measurements.

*Part 3: Confocal immunofluorescence*

At sacrifice, the brain was excised from NP-treated R6/2 mice. Half of the brain was formalin-fixed, cryopreserved by sucrose, sectioned, and stained by DAPI. Tissue sections were stained by primary antibodies against iba1 and NeuN to indicate microglia and neurons respectively. For each section-containing slide, 2 pictures (290 μm × 290 μm; 40× under a confocal microscope) were taken such that 4 pictures in both CTX and STR were counted for analysis of each mouse. 500 $NeuN^+$ cells in both CTX and STR were selected, and NP-positive cells were counted. 200 $iba1^+$ cells were selected for counting as they are less abundant than $NeuN^+$ cells. $Iba1^+$ area was measured by the ImageJ software. The total number of $MW8^+$ aggregates in each image was counted and divided by the total number of $NeuN^+$ cells. The average value of 4 pictures per mouse was displayed using a stacked bar chart with scatter plot points.

*Part 4: Crossing the blood-CSF barrier*

PBS-perfused brains were harvested, fixed in 10% formalin (3.7% w/v) for 24 h, and exchanged with 30% sucrose (w/v) in PBS at 4 ºC until the brains sank. The perfused brains were frozen in Shandon Cryomatrix frozen embedding medium (Thermo Fisher Scientific) and cut into sagittal sections of 10 μm thick and mounted on Superfrost PlusTM Adhesion microscope slides (Thermo Scientific). After washing with PBS for 5 min, the sagittal sections were blocked in with 3% bovine serum albumin (BSA, Rockland) with 0.3% Triton X-100 (Thermo Fisher Scientific) in PBS for 1 h, and stained with primary antibodies in blocking buffer at 4 °C overnight. The primary antibodies include rabbit polyclonal against CD31 (Abcam, ab28364; 0.07 μg/mL) and mouse monoclonal against E-cadherin (BD Transduction, 610181; 1.25 μg/mL). After rinses with PBS, the sections were stained with secondary antibodies at RT for 2 h: AF 488-conjugated goat anti-mouse (Invitrogen, A-11029; 2 μg/mL) and AF 647-conjugated goat anti-rabbit (Invitrogen, A-21245; 2 μg/mL). Slides were stained by 1 μg/mL DAPI for 10 min at RT, mounted with antifade mounting medium (Phygene, PH0429), and visualized under a confocal laser scanning microscope at identical imaging settings, in reflection mode for detecting Au NP reflectance. The excitation wavelengths of DAPI, AF488, AF647 are 405, 499, 650 nm, respectively. The corresponding emission wavelengths are 415−470, 506−600 and 660−770 nm, respectively.

**Efficacy and mechanistic evaluation by imaging and molecular assays**

*Part 1: Confocal immunofluorescence*

At sacrifice, the brain was excised from NP-treated healthy littermates and R6/2 mice. Half of the brain was formalin-fixed, cryopreserved by sucrose, sectioned, and stained by DAPI. Tissue sections were stained by primary antibodies against MW8, iba1, NeuN that indicate HD exon-1 67Q aggregate, microglia, and neurons, respectively. For each section-containing slide, 2 pictures (290 µm × 290 µm; 40× under a confocal microscope) were taken such that 4 pictures in both CTX and STR were counted for analysis of each mouse. Iba1$^+$ area was measured by the ImageJ software. Total number of MW8$^+$ aggregates in each image was counted and divided by the total number of NeuN$^+$ cells. The averaged value of 4 pictures per mouse was displayed using a stacked bar chart with scatter plot points.

*Part 2: Lipid peroxidation*

CTX and STR tissues were homogenized (Biospec Products) in 300 or 600 µL of pre-cooled 20 mM Tris (pH 7.4) containing Pierce protease inhibitors (Thermo Fisher Scientific), respectively. After collecting the protein supernatants by centrifugation at 10,000 × g for 5 min at 4 ºC, 100 µL of the supernatants was used for determining the concentration of malonaldehyde (MDA) and 4-hydroxynonenal (HNE) using the Lipid Peroxidation Assay Kit (Bioquochem, KB03002).

*Part 3: NAD$^+$/NADH ratio*

CTX and STR tissues were homogenized in 300 or 600 µL of pre-cooled T-PER™ tissue protein extraction reagent (Thermo Fisher Scientific) containing Pierce protease inhibitors and phosphatase inhibitor cocktail (Bio-Platform) on ice, respectively. After incubating the homogenized CTX and STR tissues on ice for 30 min for complete lysis, the protein supernatant was collected by centrifugation at 20,000 × g for 10 min at 4 ºC. NAD$^+$/NADH ratio was determined using the NAD/NADH Assay Kit (Abcam, ab176723) by measuring the fluorescence at excitation and emission wavelengths of 540 and 590 nm, respectively, using SpectraMax M3 (Molecular Devices).

*Part 4: ATP quantification*

CTX and STR tissues were respectively homogenized in 300 or 600 µL of pre-cooled NET lysis buffer (20 mM Tris, 100 mM NaCl, 1 mM EDTA, 0.5% Triton X) containing Pierce protease inhibitors. After incubating the homogenized tissues on ice for 30 min for complete lysis, the protein supernatant was collected by centrifugation at 10,000 × g for 5 min at 4 ºC. ATP standard

ranging from 1 nM to 1 μM prepared in NET lysis buffer as above. The ATP Determination Kit (Invitrogen) was used to determine the ATP concentrations of the samples per the manufacturer's instructions by measuring the luminescence using SpectraMax M3 (Molecular Devices).

*Part 5: Western blot analysis*

Initially, 25 μL of tissue lysate or different concentrations of serially diluted bovine serum albumin (BSA, Pierce) were transferred into a 96-well plate. After adding 200 μL of freshly prepared BCA Protein Assay working reagent (Pierce) to each well, the samples were incubated at 37 °C for 30 min. Absorbance at 562 nm was measured using microplate reader SpectraMax M3 (Molecular Devices). Protein concentrations were calculated using BSA as a standard of calibration. Next, 20 μg of tissue lysate was electrophoresed through a 10% TGX denaturing polyacrylamide gel (BioRad) and transferred to a polyvinylidene difluoride membrane (BioRad) at 25 V for 10 min using a semi-dry transfer system (Power Blotter System, Invitrogen). After blocking in 5 mL of 5% BSA (Rockland) in Tris-buffered saline-Tween 20 (TBST) buffer for 1 h, the blots were incubated with 5 mL of primary antibodies diluted in TBST containing 5% BSA overnight at 4°C, including rabbit against phospho-MAPK 14 (p-p38α; Invitrogen, MA5-15177; 71 ng/mL), rabbit against MAPK 14 (p38α; Cell signaling; 23 ng/mL), rabbit against nucleotide-binding domain, leucine-rich–containing family, pyrin domain–containing-3 (NLRP-3; Invitrogen, SC06-23; 2 μg/mL), rabbit against beta-tubulin (β-tubulin; Abcam, ab108342; 46.5 ng/mL), rabbit against cleaved gasdermin D (GSDMD; Cell signaling, 36425, 18 ng/mL), rabbit against phospho-pyruvate dehydrogenase α1 (p-PDH α1, Cell signaling, 37115; 63 ng/mL), rabbit against pyruvate dehydrogenase (PDH, C54G1; Cell signaling, 3205; 15 ng/mL), rabbit polyclonal zonula occludens-1 (ZO-1; 61-7300, Invitrogen; 0.25 μg/mL). After three PBS rinses, the blot was incubated with 5 mL of 1 μg/mL goat secondary antibody (conjugated with horse radish peroxidase) against rabbit (1706515; Bio-Rad) diluted in TBST containing 5% non-fat milk for 1 h. The membranes were treated with Clarity™ Western ECL Substrate (Bio-Rad) and the protein bands were visualized by the ChemiDoc Touch Imaging System (Bio-Rad).

*Parts 6 and 7: ELISA and caspase-1 activity*

60 mg of CTX and 30 mg of STR tissues were homogenized in 0.6 and 0.3 μL of pre-cooled T-PER™ tissue protein extraction reagent (Thermo Fisher Scientific; 78510) containing Pierce protease inhibitors and phosphatase inhibitor cocktail (Bio-Platform; BP103) on ice, respectively. After incubating the homogenate on ice for 30 min for complete lysis, the protein supernatant was

collected by centrifugation at 10,000 × g for 5 min at 4 ºC. For ELISA, the protein supernatant was diluted by 5 folds before adding to the ELISA kits for mouse TNF-α, mouse IL-1β, mouse IL-4, and mouse IL-6 (MAX™ Deluxe Sets, Biolegend). For caspase-1 activity, the protein supernatant was diluted by 3 folds before adding to the Caspase-1 Assay Kit (abcam; ab39412).

### *In vivo* toxicity

*Part 1: Long-term change in behavior*

Upon treatment from Weeks 6 to 10, both healthy littermates and R6/2 mice took the rotarod test every other week from Weeks 13 to 23 to monitor any adverse effects on animal behavior. Survival was monitored from Weeks 6 to 24.

*Part 2: Histology*

Following NP treatment on Week 10 or long-term monitoring on Week 23, R6/2 mice were sacrificed on Weeks 11 or 24, respectively. The brain and other major organs were fixed in 10% buffered formalin (3.7% w/v) for 24 h and stored in PBS (0.1 M) at 4°C until tissue dehydration. Fixed tissues were dehydrated in ethanol, cleared in xylene, and embedded in paraffin blocks. Paraffin-embedded tissue sections (5 µm) were cut and mounted on Superfrost PlusTM Adhesion microscope slides (Thermo Fisher Scientific). Tissue sections were deparaffinized in xylene (5 min × 3 times), rehydrated through a series of ethanol (100%, 90%, 70%; 3 min × 2 times at each concentration) and Nanopure water (5 min × 3 times), and stained with Harris hematoxylin (Sigma, HHS16) for 3 min and eosin (Sigma, 45260) for 30 s. To evaluate tissue morphology, bright-field images were taken with a Nikon Eclipse Ti microscope.

To evaluate immunogenicity by histology, immunohistochemistry (IHC) staining was performed on paraffin sections with antigen retrieval. Tissue sections were deparaffinized and rehydrated. After incubation in citrate buffer (10 mM citric acid, pH = 6), the slides were heated in the microwave oven for 3 min under high power (~95–100 °C) and for 20 min under low power. After cooling for 30 min, the slides were rinsed in distilled water twice and in PBS for 5 min. The slides were blocked with 2.5% normal horse serum (Vector Laboratories) for 2 h and incubated with 60 µL of primary antibodies [10 µg/mL for IFN-γ (eBioscience, 14-7311-81); 1 µg/mL for TNF-α (Antibodies Online, AA 181-235); 1 µg/mL for IL-6 (Antibodies Online, AA 111-150)] at 4 °C overnight. Slides were washed in PBS, treated with 3% $H_2O_2$ (Merck Millipore) for 30 min, rinsed, incubated with 50 µL of secondary antibodies [ImmPRESS™ HRP Polymer Detection Kit, Vector

Laboratories] for 30 min, and developed sequentially using 3,3'–diaminobenzidine (DAB) enzyme substrate (ImmPACT™ DAB, Vector Laboratories; 2 min for IFN-γ; 30 s for TNF-α; 1 min for IL-6). Slides were counterstained with Mayer's hematoxylin for 3 min, washed in distilled water, dried in 90% ethanol, and mounted with xylene–based mounting medium (DPX Mountant; Sigma, 06522). Bright–field images were taken with a Nikon Eclipse Ni (DS–Ri2) microscope.

*Part 3: Blood biochemistry, ELISA and cytokine profiling*

~1 mL of blood, collected via an intracardiac puncture, was stored in plain tubes for biochemistry tests and in EDTA-coated tubes (Becton Dickinson) for hematology analysis. Blood samples were kept on ice and sent to PathLab Medical Laboratories (Hong Kong) for analysis on the same day. 100 µL of plasma was collected for anti-PEG IgG ELISA (FineTest, EM2149) per manufacturer's instruction, and 100 µL of plasma was sent to H · Wayen Biotechnologies for cytokine profiling.

For cytokine profiling, plasma samples were first centrifuged at 10000 rpm for 10 min before taking the supernatant for 5-fold dilution with sample diluent. Next, 50 µL of diluted plasma sample (or internal standards and blank assay buffer) was added to each well of a 96-well plate, followed by adding 50 µL of diluted microbeads into the same well. The plate was sealed with a film and incubated on a plate shaker at 850 rpm, protected from light, at RT for 30 min. After three rinses, each well was incubated with 50 µL of diluted detection antibody (1:10) for 30 min. After removing the detection antibody and three more rinses, 50 µL of diluted streptavidin-PE (100X) was added to each well, sealed with a film, and incubated on a plate shaker at 850 rpm, protected from light, at RT for 10 min. After adding 125 µL of assay buffer to each well for resuspension, the plate was sealed and shaken on a plate shaker at 850 rpm (protected from light) at RT, for 2 min. Lastly, the plate was transferred to a calibrated Bio-Plex instrument (Luminex) for analysis based on instructions from Bio-Plex Pro Mouse Chemokine Panel (Biorad).

**Ex vivo skeletal muscle and neuromuscular junction (NMJ) functional test**

Week 11 R6/2 HD mice and age-matched healthy littermates were subject to skeletal muscle and NMJ functional tests ex vivo (800A, Aurora Scientific)[4]. Upon anaesthesia, the triceps surae-sciatic nerve complex was isolated from the right or left hindlimb of the mouse, ensuring intactness of the nerve terminals and NMJ structure. The surgical dissection to isolate the sciatic nerve from the surrounding tissue was performed in a dish with chilled (4 °C) simulated cerebrospinal fluid (CSF) buffer[4]. The isolated muscle-nerve complex was immersed in a synthetic interstitial fluid

(SIF) buffer [123 mM NaCl, 3.5 mM KCl, 0.7 mM $MgSO_4$, 1.7 mM $NaH_2PO_4$, 2.0 mM $CaCl_2$, 9.5 mM $NaC_6H_{11}O$ (sodium gluconate), 5.5 mM glucose, 7.5 mM sucrose, and 10 mM N-2-hydroxyethylpiperazine-N′-2-ethanesulfonic acid (HEPES); pH 7.4±0.05][4], supplied with carboxygen (95% $O_2$, 5% $CO_2$) for maintaining viability, and mounted onto the ex vivo apparatus between two parallel electrodes. The parallel electrodes stimulate muscle contraction directly, while the suction electrode induces muscle contraction indirectly by stimulating the sciatic nerve. The specific length at which the muscle fiber develops the largest isometric force, or a force of contraction without shortening, is regarded as the optimal length ($L_o$) of the muscle. A single pulse applied to muscle fiber results in a single contraction or "twitch", while continuous or sustained contraction is termed "tetanic" contraction[4]. The decline in maximal force production in response to contractile stimuli is defined as muscle fatigue, induced by repeated tetanic stimuli to assess the skeletal muscle fatigability[4]. In this test, the $L_0$ was determined by stimulating muscle contraction at different muscle lengths with a small increment (0.2 mm), until the response twitch force was stabilized. At the $L_0$, the triceps surae muscle and sciatic nerve were stimulated by a single twitch stimulus with 1-min interval (muscle stimulus: 300 mA, 0.2 ms pulse width; nerve stimulus: 5 mA, 0.8 ms pulse width)[4]. After 1 min, the muscle and nerve were stimulated separately by a continuous tetanic stimulus with a 2-min interval (muscle stimulus: 300 mA, 300 ms duration, 0.2 ms pulse width, 50 Hz stimulation frequency; nerve stimulus: 5 mA, 300 ms duration, 0.8 ms pulse width, 50 Hz stimulation frequency). To assess fatigability, the triceps surae muscle was stimulated at 50 Hz at 0.7 s intervals for 100 consecutive cycles, a 15 min rest was allowed, and the sciatic nerve was stimulated with the same parameters. The intratetanic fatigue of muscle and NMJ were calculated from two consecutive tetanic pulse trainings, using the following equation[4]:

Intratetanic fatigue = $\frac{F_{lp}}{F_m} \times 100\%$

$F_{lp}$: force generated at the last pulse of stimulation in every 10 stimuli

$F_m$: the maximum force generated during the same pulse train in every 10 stimuli

**Skeletal muscle mass measurement**

Body compositional analysis of Week 11 R6/2 mice and age-matched healthy littermates was performed using dual-energy X-ray absorptiometry (DXA) imaging (Kubtec PARAMETER 2D). After anesthesia, mice were placed in prone position with four limbs extended away from the body. Imaging scans were taken, and lean mass percentage was calculated by the DIGIMUS software.

# Appendix I: Background

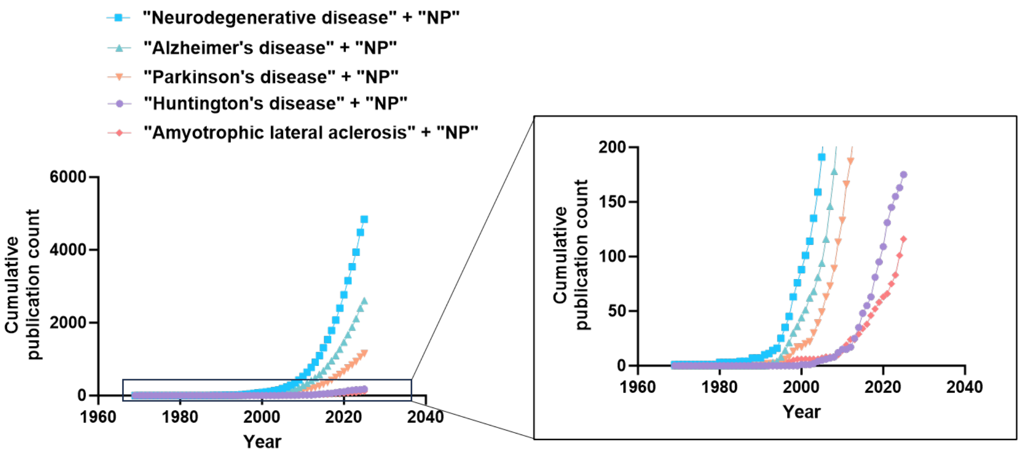


**Figure S1.** Trends of nanoparticles (NPs) for managing neurodegenerative diseases in the literature in terms of cumulative publication count excluding reviews from 1969 to July 2025 based on defined keyword searches in PubMed. Note that NP here refers to keywords of "nanoparticle", "liposome" or "micelle". All curves refer to NP-based applications *in vitro* and *in vivo*, including but not limited to diagnostics, therapy, and imaging. The blue curve refers to NP-based applications for all neurodegenerative diseases. The green, orange, purple, and red curves refer to NP-based applications specifically for Alzheimer's diseases, Parkinson's diseases, Huntington's diseases, and amyotrophic lateral sclerosis, respectively.

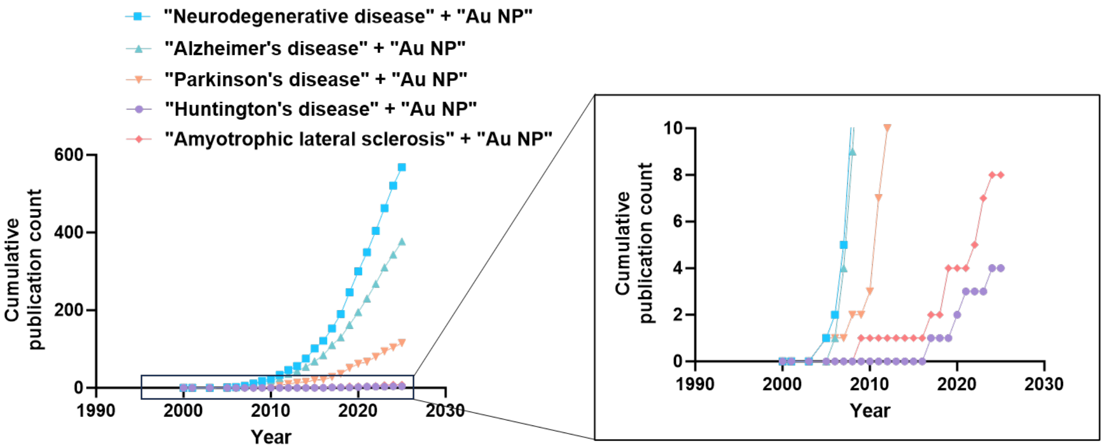


**Figure S2.** Trends of gold nanoparticles (NPs) for managing neurodegenerative diseases in the literature in terms of cumulative publication count (excluding reviews) from 1969 to July 2025 based on defined keyword searches in PubMed. The first year with non-zero publication is 2005. Note that NP here refers to keywords of "nanoparticle", "liposome" or "micelle". All curves refer to gold NP-based applications *in vitro* and *in vivo*, including but not limited to diagnostics, therapy, and imaging. The blue curve refers to gold NP-based applications for all neurodegenerative diseases. The green, orange, purple, and red curves refer to gold NP-based applications specifically for Alzheimer's diseases, Parkinson's diseases, Huntington's diseases, and amyotrophic lateral sclerosis, respectively.

**Table S1.** Recent work done using nanoparticles (NPs) to treat preclinical models of Huntington's disease (HD) since July 2020.[a] For earlier past research reports, please refer to our published review[20]:

| NP | Physicochemical properties[b, c] | Active ingredient | Mechanism of action | Key *in vivo* efficacy results | Disease model; repeated number of polyglutamine (polyQ); method of disease model development | Route of administration and dosage details[d] | Brain region (s) in the disease site | Ref. |
|---|---|---|---|---|---|---|---|---|
| Gene therapy | | | | | | | | |
| Lipid-enabled and unlocked nucleic acid (LUNAR) modified RNA REPU910 NP | NA | siRNA (REPU910) against *CAG* repeats | *HTT* mRNA targeting | Reduced HTT proteins | R6/2 mouse model; 160–190Q; transgenic | On postnatal Day 1, mice were injected with NPs (1800 ng/mice/once, i.c.); Kill on postnatal Day 4. | Cortex, cerebellum | 21 |
| Phosphatidylserine binding motif (G58)-peptide of transactivator of transcription (TAT) and peptide of flock house nodovirus domain (TF) Extracellular vesicles (EVs) | $D_H$: 100–200 nm ζ: NA | siRNA against *HTT* gene | *HTT* mRNA targeting | Reduced *HTT* mRNA, and HTT protein inclusion | Q140 mouse model; 140Q; transgenic | At the age of 1 year, mice were injected with NPs (0.5 mg siRNA/kg/week for 4 wk, i.v.). Kill at 72 h post-injection. | Cortex, cerebellum, midbrain | 22 |
| Apolipoprotein A-I nanodisks (ApoA-I NDs) | $D_H$: 12.4 nm ζ: NA | ASO against *HTT* gene | *HTT* mRNA targeting | Reduced *HTT* mRNA | BACHD mouse model; 97Q; transgenic | At the age of 2 months, mice were injected with NDs (10–60 mg/kg, i.v.). Kill 2 h post-injection. | Cortex, striatum, hippocampus, cerebellum | 23 |
| Small peptides | | | | | | | | |
| Au NPs-JLD1 (an amphiphilic peptide)-polyethyleneimine (PEI) | $D_H$: ~22 nm | JLD1 | HTT protein deaggregation | Improved motor function of larva by locomotor activity assay. | HD larva model; 108Q; transgenic | On Day 1, the third instar larvae were fed with NPs (3 nM/feeding for days). | NA | 24 |

| NP | Physicochemical properties[b, c] | Active ingredient | Mechanism of action | Key *in vivo* efficacy results | Disease model; repeated number of polyglutamine (polyQ); method of disease model development | Route of administration and dosage details[d] | Brain region (s) in the disease site | Ref. |
|---|---|---|---|---|---|---|---|---|
| | | | | | | Killing time not available | | |
| Small molecules | | | | | | | | |
| Nano Ivabradine (IVA) in liposome | NA | IVA | Boosted expression of autophagy in Rhes/m-Tor pathways | Reversed motor disabilities, improved memory function and overcame the psychiatric changes | HD rat model; nonpathogenic number of Q; 3-NP induced | From Days 1 to 14, rats (108–240 g) were injected with 3-NP (20 mg/kg/day, i.p.).<br><br>From Days 15 to 21, rats were injected with NPs (1 mg/kg/every other day for 7 d, i.v.). Kill on Day 24. | Striatum | 25 |
| PLGA-encapsulated epigallocatechin-3-gallate (EGCG) and ascorbic acid | $D_H$: 125 ± 5 nm ζ: −16 ± 2 mV | EGCG and ascorbic acid | Anti-inflammation | Reducing motor disturbances and depression-like behavior; mitigated neuroinflammation and prevented neuronal loss | HD mouse model; nonpathogenic number of Q; 3-NP induced | From Days 1 to 5, mice (age of 6 weeks) were injected with 3-NP (70 mg/kg/day for 5 d). From Days 1 to 5, NPs were injected (50 mg/kg for 5 d, i.p.). Kill on day 5. | Cortex, striatum, hippocampus | 26 |
| Piperine-loaded casein micelles | NA (SEM size of ~50 μm) ζ: NA | Piperine | Oxidative stress relief, anti-inflammation | Improved track plots, reduced oxidative stress markers and inflammation cytokine | HD zebra fish; nonpathogenic number of Q; 3-NP induced | On Day 1, fully grown zebra fishes were injected with 3-NP (60 mg/kg, i.p.). From Days 2 to 5, zebra fishes were injected with | NA | 27 |

| NP | Physicochemical properties[b, c] | Active ingredient | Mechanism of action | Key *in vivo* efficacy results | Disease model; repeated number of polyglutamine (polyQ); method of disease model development | Route of administration and dosage details[d] | Brain region (s) in the disease site | Ref. |
|---|---|---|---|---|---|---|---|---|
| | | | | | | NPs (25 mg/kg/day). Kill on Day 6. | | |
| Self-therapeutic nanoparticles | | | | | | | | |
| $Au_3$@$PEG_{1k}$ NP | $D_H$: 10.7 ± 0.7<br>ζ: -9.6 ± 0.5 | $Au_3$@$PEG_{1k}$ NP | Inhibition of p-p38α phosphorylation and PDK1 activities; HTT protein deaggregation | Improved motor function of disease mice by rotarod and open field test; reduced pyroptosis cell death; prolong survival; reduced cytokines (TNF-α, IL-1β, IL-4, IL-6) | R6/2 mouse model; 120Q; transgenic | From age of Week 6 to 10, NPs were injected (0.7 mg/week or 136 mg/kg/week for 5 wk, i.v.). Kill at age of Week 11. | Cortex, striatum | This work |
| $C_{60}(OH)_{30}$ and $C_{120}O(OH)_{44}$ fullerenols | $C_{60}(OH)_{30}$<br>$D_H$: 144 ± 44 nm and 814 ± 143 nm<br>ζ: −15.2 ± 6.3 mV<br>$C_{120}O(OH)_{44}$<br>$D_H$: 14 ± 4 nm and 516 ± 45 nm<br>ζ: −28.8 ± 6.3 mV | $C_{60}(OH)_{30}$ and $C_{120}O(OH)_{44}$ fullerenols | Oxidative stress relief | Reduced neuronal cell death | UAS-HTT.128Q.FL *Drosophila melanogaster*; 128Q; transgenic | Drosophila were fed with NP every 2 d (0.2 mg/mL of fullerenols $C_{60}(OH)_{30}$, or 0.01 mg/mL $C_{120}O(OH)_{44}$). Kill on Days 5, 15, 25 or survival. | NA | *28* |
| $C_{60}$ fullerenes | NA | $C_{60}$ fullerene | Oxidative stress relief | Reduced apoptotic and ferroptotic cell death | HD rat model; nonpathogenic number of Q; 3-NP induced | From Days 3 to 5, rats (220–260 g) were injected with 3-NP (30 mg/kg/day, i.p.) | Brain mitochondria | *29* |

| NP | Physicochemical properties[b, c] | Active ingredient | Mechanism of action | Key *in vivo* efficacy results | Disease model; repeated number of polyglutamine (polyQ); method of disease model development | Route of administration and dosage details[d] | Brain region (s) in the disease site | Ref. |
|---|---|---|---|---|---|---|---|---|
| | | | | | | Pre-treatment:<br>From Days 1 to 5, rats were injected with NPs (0.5 mg/kg/day for 5 d, i.p.). Kill 24 h post-injection.<br><br>Post-treatment:<br>From Days 6 to 10, rats were injected with NPs (0.5 mg/kg/day for 5 d, i.p.). Kill 24 h post-injection. | | |
| C-$Mn_3O_4$ NPs | $D_H$: 21.5 ± 4.1 nm<br>ζ: −12.2 ± 0.6 mV | C-$Mn_3O_4$ nanozyme | Oxidative stress relief | Improved motor, anxiety and depression-like behavior, mitochondrial function, reduce ferroptotic like cell death | HD C57BL/6j model; nonpathogenic number of Q; 3-NP induced | From Days 1 to 4, mice (age of 6–8 weeks) were induced by 3-NP (10 mg/kg). From Days 5 to 20, the C57BL/6j were i.p. injected with NPs. | Cerebellum basal ganglia | 30 |
| Other treatments of peripheral system indications (not the brain) but using HD-related disease models | | | | | | | | |
| Palmitoyl-oleoyl-phosphatidylcholine (POPC)-and | NA | HA-1077 (a ROCK inhibitor) | HTT protein deaggregation | Improved retinal function with increased conemediated | R6/2 mouse model; 120Q; transgenic | On Day 1, mice (age of Week 5) were injected with NPs (40 pmol NP/mouse/once, | Retina | 31 |

| NP | Physicochemical properties[b, c] | Active ingredient | Mechanism of action | Key *in vivo* efficacy results | Disease model; repeated number of polyglutamine (polyQ); method of disease model development | Route of administration and dosage details[d] | Brain region (s) in the disease site | Ref. |
|---|---|---|---|---|---|---|---|---|
| cholesterol-based liposomes | | | | electroretinography (ERG) response amplitudes. | | intravitreal). Kill after 2 weeks. | | |

[a]Abbreviations: $D_H$ = hydrodynamic diameter; ζ = zeta potential; REPU910 = CAG-siRNA with a UNA modification at positions 9 and 10; 3-NP = 3-nitropropionic acid; i.v. = intravenous injection; i.n. = intranasal injection; i.c. = intracranial injection; i.p. intraperitoneal injection; SEM = scanning electron microscope; PDK1 = pyruvate dehydrogenase kinase 1; NA = not available. [b]The physicochemical properties are expressed as a range of values, ± standard deviation, or just the mean of the data depending on data availability in the cited work. [c]If multiple physicochemical properties of the NPs were studied in the cited work, only the optimal NP properties are shown here. [d]If multiple doses or treatment schedules were attempted, only the optimized treatment plans are shown here.

**Table S2.** Self-therapeutic gold nanoparticles (NPs) for alleviating neurodegenerative diseases[a] preclinically or clinically in Figure S2.[b]

| NP | Physicochemical properties[c, d] | Biological pathway or target | Mechanism of action | Key *in vivo* efficacy / clinical results | Neurodegenerative disease (disease model; method of disease model development; gene of manipulation if any) or disease patient (clinical trial) | Route of administration and dosage[e] | Studied region (s) in the disease site | Ref. |
|---|---|---|---|---|---|---|---|---|
| Huntington's disease | | | | | | | | |
| $Au_3$@$PEG_{1k}$ NP | $D_{TEM}$: 3.3 ± 0.6 nm $D_H$: 10.7 ± 0.7 nm ζ: -9.6 ± 0.5 mV | p38α phosphorylation, PDK1 activities and HTT protein | Inhibition of p-p38α phosphorylation and PDK1 activities; HTT protein deaggregation | Improved motor function of disease mice by rotarod and open field test; reduced pyroptosis cell death; prolong survival; reduced cytokines (TNF-α, IL-1β, IL-4, IL-6) | R6/2 mouse model; 120Q; transgenic | From age of Week 6 to 10, NPs were injected (0.7 mg/week or 136 mg/kg/week for 5 wk, i.v.). Kill at age of Week 11. | Cortex, striatum | This work |
| Alzheimer's disease (AD) | | | | | | | | |
| Citrate-capped Au NPs | $D_{TEM}$: ~20 nm $D_H$: NA ζ: NA | NA | Oxidative stress relief; anti-neuroinflammation | Improved cognitive functions by Barnes maze | AD rat model; Okadaic acid-induced | On Day 1, rats (250–300 g) were injected with OA (100 | Cortex, hippocampus | [32] |

| NP | Physicochemical properties[c, d] | Biological pathway or target | Mechanism of action | Key *in vivo* efficacy / clinical results | Neurodegenerative disease (disease model; method of disease model development; gene of manipulation if any) or disease patient (clinical trial) | Route of administration and dosage[e] | Studied region (s) in the disease site | Ref. |
|---|---|---|---|---|---|---|---|---|
| | | | | task; reduced Tau phosphorylation; increased cytokines (IL-1β and IL-4); reduced ROS; increased antioxidative activities | | μg/mice/once, i.c.) From Days 2 to Day 22, NPs were injected (2.5 mg/kg/48 h for 10 times; i.p.). Kill on Day 24. | | |
| Citrate-capped Au NPs | $D_{TEM}$: ~20 nm $D_H$: NA ζ: NA | NF-κB | Oxidative stress relief; anti-neuroinflammation | Improved cognitive functions by Barnes maze task; reduced ROS; improved mitochondrial potential; increased antioxidative activities; | AD rat model; STZ-induced | On Day 1, the rats (250–300 g) were injected with STZ (3mg/kg, i.c., 2 μL/hemisphere). From Day 3 to Day 24, NPs were injected (2.5 mg/kg/48 | NA | 33 |

| NP | Physicochemical properties[c, d] | Biological pathway or target | Mechanism of action | Key *in vivo* efficacy / clinical results | Neurodegenerative disease (disease model; method of disease model development; gene of manipulation if any) or disease patient (clinical trial) | Route of administration and dosage[e] | Studied region (s) in the disease site | Ref. |
|---|---|---|---|---|---|---|---|---|
| | | | | reduced cytokines (IL-1β) | | h for 10 times, i.p.) Kill on Day 26. | | |
| Glutathione (GSH)-capped chiral Au NPs | $D_{TEM}$: 3.3 ± 0.4 nm<br>$D_H$: 2.5 ± 1.0 nm<br>ζ: NA | Aβ42 plaque | Aβ plaque deaggregation | Reduced apoptotic cell death; reduced soluble and insoluble Aβ plaque; improved cognitive function by Morris water maze | AD mouse model; transgenic; *APP/PS1* | On Week 1, mice (age of 5 months, male) were injected with NPs (25 mg/kg/weeks for 4 wk, i.v.). Kill on Week 5 /after Morris water maze. | Hippocampus | 34 |

| NP | Physicochemical properties[c, d] | Biological pathway or target | Mechanism of action | Key *in vivo* efficacy / clinical results | Neurodegenerative disease (disease model; method of disease model development; gene of manipulation if any) or disease patient (clinical trial) | Route of administration and dosage[e] | Studied region (s) in the disease site | Ref. |
|---|---|---|---|---|---|---|---|---|
| Cysteine-capped Au NP or Levodopa-capped Au NP | $D_{TEM}$: ~3 nm<br>$D_H$: NA<br><br>Cysteine-capped Au NP ζ: ~-32 mV<br><br>Levodopa-capped Au NP ζ: ~-25 mV | Aβ plaque | Aβ plaque deaggregation | Improved cognitive functions by spontaneous alteration test and novel object recognition | AD mouse model; STZ-induced | On Day 1, mice (25–40 g) were injected with STZ (3 mg/kg/once, i.c.). After 1 week, mice were injected with NPs (20 mg/kg/every 72 h for 4 doses, i.v.). Killing time not available. | NA | 35 |
| PEG-capped chiral Au NP | $D_{TEM}$: ~150 nm<br>$D_H$: NA<br>ζ: ~24 mV | NA | Alternation of gut microbiota | Reduced Aβ plaque; improved cognitive functions by Morris water maze and Novel object | AD (3xTg AD mouse model; transgenic; *APP*/*PS1*/*Tau*) AD (*APP*/*PS1* AD mouse model; | From Months 1 to 3, mice (age of 10 months) were injected with NPs (10 mg/kg/day, oral gavage). | Hippocampus | 36 |

| NP | Physicochemical properties[c, d] | Biological pathway or target | Mechanism of action | Key *in vivo* efficacy / clinical results | Neurodegenerative disease (disease model; method of disease model development; gene of manipulation if any) or disease patient (clinical trial) | Route of administration and dosage[e] | Studied region (s) in the disease site | Ref. |
|---|---|---|---|---|---|---|---|---|
| | | | | recognition; reduced cytokines (TNF-α, IL-1β, IL-6, IL-18) and reduced pyroptosis | transgenic; *APP*/*PS1*) | Kill at Month 3. | | |
| Citrate-capped Au NPs | $D_{TEM}$: 5 nm $D_H$: 15 nm < ζ: ~48 ± 11 mV | Aβ plaque | Aβ plague inhibition or delay dissociation of Aβ plague | Improved the acquisition and retention of spatial learning and memory by Morris water maze; increased BDNF, cAMP response element binding protein, CREB, and stromal | AD (AD rat model; i.c. injection of Aβ) | After i.c. injection of Aβ, from Days 1 to 16, rats were injected with NPs (2 to 200 ng/rat/4 days, i.c.). Kill at Day 24.<br><br>After i.c. injection of Aβ, from Days 1 to 16, rats were injected with NPs (2 to | Hippocampus | [37] |

| NP | Physicochemical properties[c, d] | Biological pathway or target | Mechanism of action | Key *in vivo* efficacy / clinical results | Neurodegenerative disease (disease model; method of disease model development; gene of manipulation if any) or disease patient (clinical trial) | Route of administration and dosage[e] | Studied region (s) in the disease site | Ref. |
|---|---|---|---|---|---|---|---|---|
| | | | | interaction molecules | | 200 μg/kg/4 days, i.p.). Kill at Day 24. | | |
| Parkinson's disease (PD) | | | | | | | | |
| Citrate-capped Au NPs | $D_{TEM}$: ~20 nm<br>$D_H$: NA<br>ζ: NA | NA | Oxidative stress relief; restoring cellular factors | Improved motor function by rotarod test; reduced ROS; increased antioxidative activities; reduced oxidative damage; restoring neurotrophins | PD mouse model; reserpine-induced | From Days 1 to 5, mice (20–30 g) were injected with reserpine (0.25 mg/kg/48 h for 3 times), s.c.). Meanwhile, NPs were injected (2.5 mg/kg/day for 5 d, i.p.). Kill on Day 6. | Cortex, striatum, hippocampus | 38 |
| N-isobutyryl-L-cysteine (NIBC)-capped Au NPs | $D_{TEM}$: 1.5 ± 0.5 nm<br>$D_H$: 2.5 ± 1.0 nm<br>ζ: NA | α-Synuclein fibrillation | α-Synuclein deaggregation | Reduced apoptotic cell death; reduced α-synuclein fibrillation; | PD mouse model; MPTP-induced | From Days 1 to 7, mice (age of 8 weeks) were injected with MPTP | Striatum | 39 |

| NP | Physicochemical properties[c, d] | Biological pathway or target | Mechanism of action | Key *in vivo* efficacy / clinical results | Neurodegenerative disease (disease model; method of disease model development; gene of manipulation if any) or disease patient (clinical trial) | Route of administration and dosage[e] | Studied region (s) in the disease site | Ref. |
|---|---|---|---|---|---|---|---|---|
| | | | | improved motor function by open field, swimming test, rotarod test; increased in tyrosine hydroxylase (TH) expression | | (30 mg/kg/day for 7 days, i.p.). Meanwhile, half an hour in advance, NPs (20 or 5 mg/kg/day for 7 d, i.p.). Kill on Day 8. | | |
| Paeonia moutan-capped Au NPs | $D_{TEM}$: ~100 nm<br>$D_H$: NA<br>ζ: NA | NA | Oxidative stress relief; anti-inflammation | Improved motor function by footprint, grip strength, rotarod test; reduced cytokines (TNF-α, IL-1β, IL-6); reduced pro-inflammatory | PD mouse model; MPTP-induced | From Days 1 to 5, mice (25–30 g) were injected with MPTP (30 mg/kg/day for 5 d, i.p.). From Days 10 to 14, NPs were injected (20 mg/kg, i.p.). | NA | 40 |

| NP | Physicochemical properties[c, d] | Biological pathway or target | Mechanism of action | Key *in vivo* efficacy / clinical results | Neurodegenerative disease (disease model; method of disease model development; gene of manipulation if any) or disease patient (clinical trial) | Route of administration and dosage[e] | Studied region (s) in the disease site | Ref. |
|---|---|---|---|---|---|---|---|---|
| | | | | proteins; reduced ROS | | Kill on Day 15. | | |
| (11-mercaptoundecyl)-N,N,N-trimethylammonium bromide (MUTAB)-PEG-coated elongated porous gold nanorods (Au NpRs) | Length: ~3 µm<br>$D_H$: NA<br>ζ: 39.8 ± 0.8 mV | NA | Direct lineage reprogramming of dopaminergic neurons | Increased *FOXA2*; improved motor behavior by apomorphine-induced rotating behaviors | PD mouse model; 6-OHDA-induced | On Day 1, mice (age of 12 weeks) were injected with 6-OHDA (100 µg/mice/once, i.c.). On Day 24, NPs were injected (13.8 pg/mice/once, i.c.). Kill on Day 66. | Striatum | [41] |
| Clinical trials | | | | | | | | |
| CNM-Au8, bicarbonate buffered Au NP | $D_{TEM}$: ~11 nm<br>$D_H$: ~ 20 nm<br>ζ: ~ -18 mV | NA | Catalyzing the conversion of NADH to $NAD^+$ | Prolonged survival in ALS patients. | ALS patients (NCT04098406, NCT05299658, NCT04297683 | ALS patients drank 30 mg NP daily for 36 weeks. | NA | [42] |

| NP | Physicochemical properties[c, d] | Biological pathway or target | Mechanism of action | Key *in vivo* efficacy / clinical results | Neurodegenerative disease (disease model; method of disease model development; gene of manipulation if any) or disease patient (clinical trial) | Route of administration and dosage[e] | Studied region (s) in the disease site | Ref. |
|---|---|---|---|---|---|---|---|---|
| | | | | Increased in $NAD^+$ to NADH ratio in the brain of PD patients. | and NCT04414345)<br><br>PD patients (NCT03815916) | PD patients drank 30 or 60 mg NP daily for 12 weeks. | | |

[a]Neurodegenerative diseases here refer to AD, PD, HD, and amyotrophic lateral sclerosis (ALS) only. [b]Abbreviations: $D_{TEM}$ = physical diameter of the gold core measured by TEM; $D_H$ = hydrodynamic diameter measured by DLS; ζ = zeta potential; Au NP = gold nanoparticles; PDK1 = pyruvate dehydrogenase kinase 1; STZ = streptozotocin; Aβ = amyloid-beta; MPTP = 1-methyl-4-phenyl-1,2,3,6-tetrahydropyridine; 6-OHDA = 6-hydroxydopamine; i.v. = intravenous injection; i.n. = intranasal injection; i.c. = intracranial injection; i.p. intraperitoneal injection; NA = not available. [c]The physicochemical properties are expressed as a range of values, ± standard deviation, or just the mean of the data depending on data availability in the cited work. [d] If multiple physicochemical properties of the NPs were studied in the cited work, only the optimal NP properties are shown here. [e] If multiple doses or treatment schedules were attempted, only the optimized treatment plans are shown here.

# Appendix II: Nanoparticle Characterization

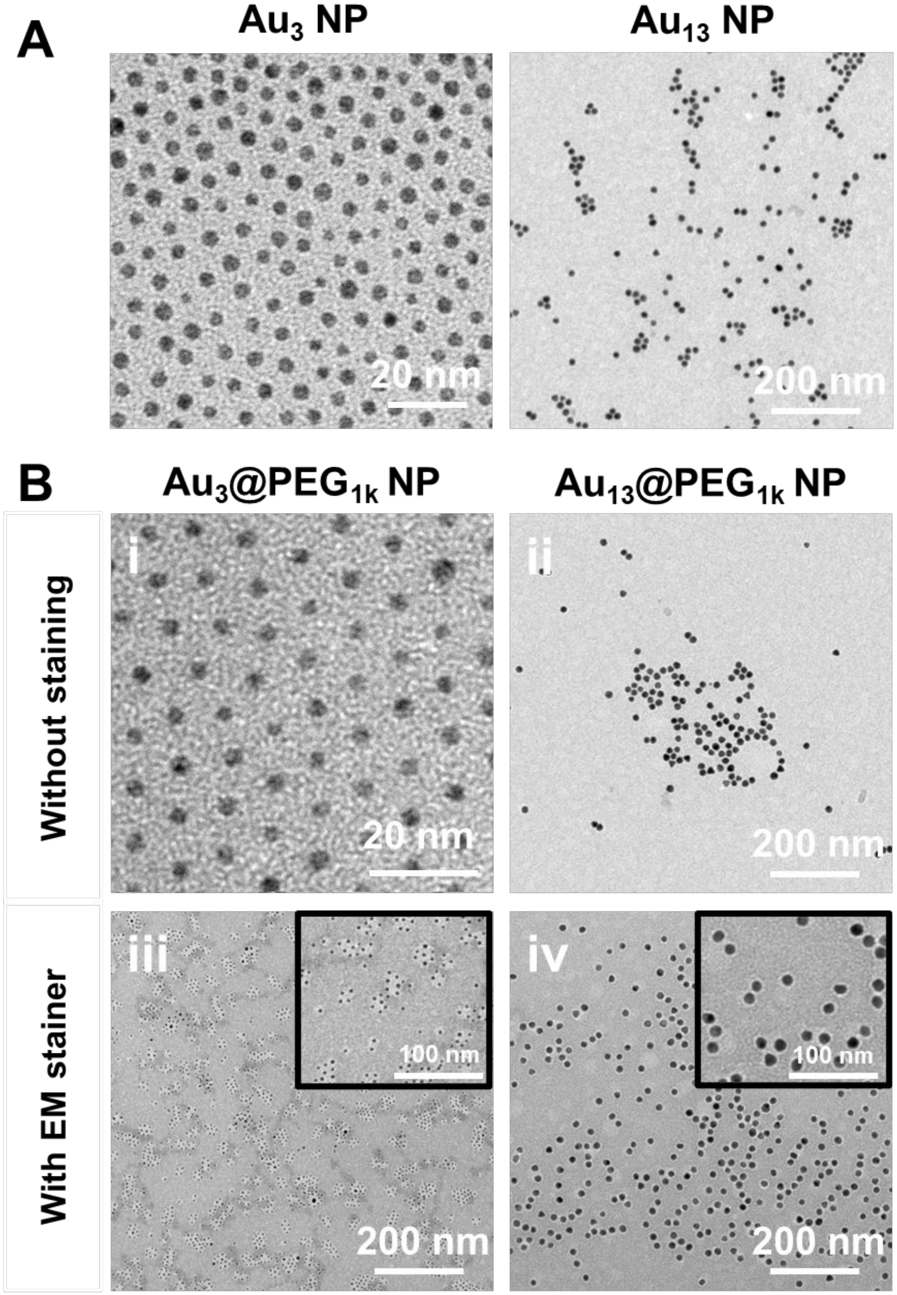


**Figure S3.** Representative TEM images of gold NPs. (A) TEM images show unmodified citrate-capped $Au_3$ NP of ~3 nm in size (left) and $Au_{13}$ NPs ~13 nm in size (right). They confirm the size and shape uniformity of $Au_x$ NPs. (B) Representative TEM images of (i) $Au_3$@$PEG_{1k}$ and (ii) $Au_{13}$@$PEG_{1k}$ NPs without negative staining, and (iii) $Au_3$@$PEG_{1k}$ and (iv) $Au_{13}$@$PEG_{1k}$ NPs with negative staining to add contrast between the PEG and background. Insets show stained NPs at a higher magnification. Successful negative stain would produce contrast between the background and NPs. The PEG shell appears as a light halo around the gold core because of its lower electron scattering power relative to the gold core and the surrounding heavy-metal background stain.

**Table S3.** Physicochemical properties of $Au_x@PEG_y$ NPs. x = diameter of Au core (nm); y = molecular weight of PEG (Da); PDI = polydispersity index.

| NP | Physical diameter of Au core (nm) [a] | Physical diameter of Au core + PEG shell (nm) [a] | Hydrodynamic diameter (nm) in water at RT [b] | ζ potential (mV) in 1 mM KCl at RT | Polydispersity index |
|---|---|---|---|---|---|
| $Au_3$ NP | 3.3 ± 0.6 | N. A. | 5.5 ± 0.6 | -27.9 ± 15.0 | 0.01 ± 0.01 |
| $Au_{13}$ NP | 12.7 ± 1.0 | N. A. | 17.0 ± 2.4 | -36.1 ± 0.8 | 0.05 ± 0.03 |
| $Au_3@PEG_{1k}$ NP | 3.0 ± 0.5 | 8.8 ± 1.0 | 10.7 ± 0.7 | -9.6 ± 0.5 | 0.03 ± 0.02 |
| $Au_{13}@PEG_{1k}$ NP | 11.6 ± 1.0 | 17.8 ± 1.1 | 22.5 ± 0.1 | -8.2 ± 1.3 | 0.05 ± 0.01 |
| Dextran 70 kDa | N. A. | N. A. | 10.2 ± 1.0 | -5.41 ± 0.4 | 0.14 ± 0.3 |

[a] Physical diameters of the gold core and gold core + PEG shell were measured by TEM with negative staining by EM Stainer. [b] Hydrodynamic diameters, polydispersity indices (PDI), and zeta (ζ) potentials were measured by dynamic light scattering (DLS) at room temperature (RT). All reported data represent mean ± SD from three independent measurements.

**Table S4.** PEG loading on $Au_x@PEG_y$ NP using the Ellman's assay (Indirect measurement) and TGA (Direct measurement).

| NP | Ellman's assay (Indirect measurement) | | TGA (Direct measurement) | |
|---|---|---|---|---|
| | Loading (strand/NP) | Loading density (strands/nm$^2$) | Loading (strand/NP) | Loading density (strands/nm$^2$) |
| $Au_3@PEG_{1k}$ NP | 182 ± 22 | 5.0 ± 0.6 | 135 | 3.7 |
| $Au_{13}@PEG_{1k}$ NP | 2229 ± 253 | 4.4 ± 0.5 | 2072 | 4.1 |

All reported data represent mean ± SD from three independent measurements. For TGA analysis, there were no technical replicates.

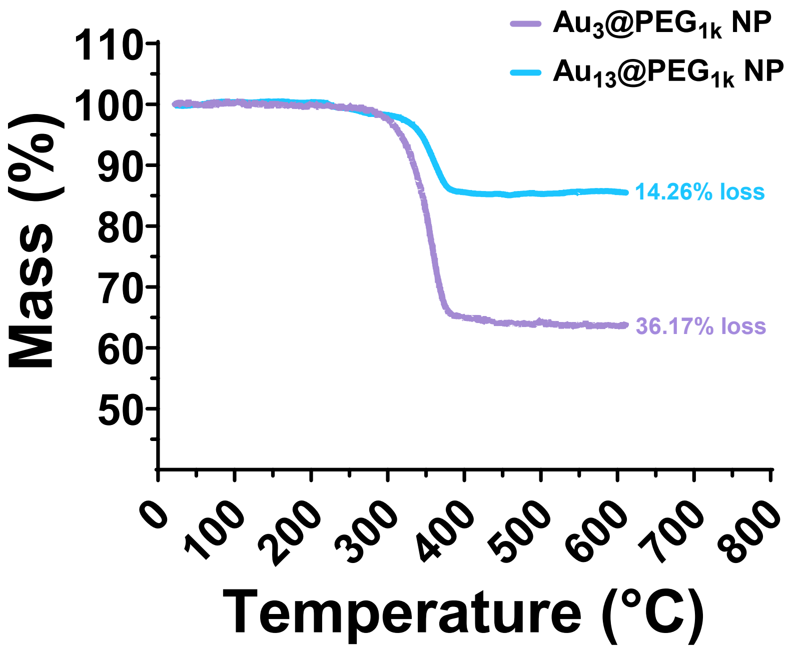


**Figure S4.** TGA of the lost PEG contents during the heating of $Au_3$@$PEG_{1k}$ (purple) and $Au_{13}$@$PEG_{1k}$ (blue) NPs as a function of temperature under dry nitrogen gas.

**Table S5.** Hydrodynamic diameter ($D_H$) of $Au_x$@$PEG_y$ NPs upon incubation in water, artificial CSF, or 50% fetal bovine serum (FBS) at 37 °C for 24 h, or in plasma upon 24 h post-i.v. injection to Week 10 R6/2 HD mice.

| **Medium** | **$Au_3$@$PEG_{1k}$ NP** | | **$Au_{13}$@$PEG_{1k}$ NP** | |
|---|---|---|---|---|
| | **$D_H$ (nm)** | **PDI** | **$D_H$ (nm)** | **PDI** |
| **Water** | 10.7 ± 0.7 | 0.03 ± 0.02 | 22.5 ± 0.1 | 0.05 ± 0.03 |
| **Artificial CSF** | 11.6 ± 0.4 | 0.03 ± 0.001 | 22.6 ± 0.4 | 0.01 ± 0.01 |
| **50% FBS** | 11.7 ± 1.7 | 0.23 ± 0.06 | 23.25 ± 2.9 | 0.74 ± 0.01 |
| **R6/2 blood plasma** | 13.8 ± 2.6 | 0.08 ± 0.04 | 26.3 ± 1.4 | 0.49 ± 0.14 |

$D_H$ was measured by dynamic light scattering. PDI = polydispersity index. All reported data represent mean ± SD from three independent measurements.

By DLS measurements, the hydrodynamic sizes of $Au_x$@$PEG_y$ NPs were slightly larger after exposure to artificial CSF or serum-containing medium (50% FBS) at 37 °C for 24 h, or 24 h post i.v. injection to Week 10 R6/2 mice. $Au_3$@$PEG_{1k}$ NPs became slightly larger from 10.7 ± 0.7 nm to 11.6 ± 0.4 nm (in artificial CSF), to 11.7 ± 1.7 nm (in 50% FBS), to 13.8 ± 2.6 nm (in plasma of Week 10 R6/2 mice). DLS measurements did not reveal drastic increase in HD after serum incubation, proof of colloidal stability.

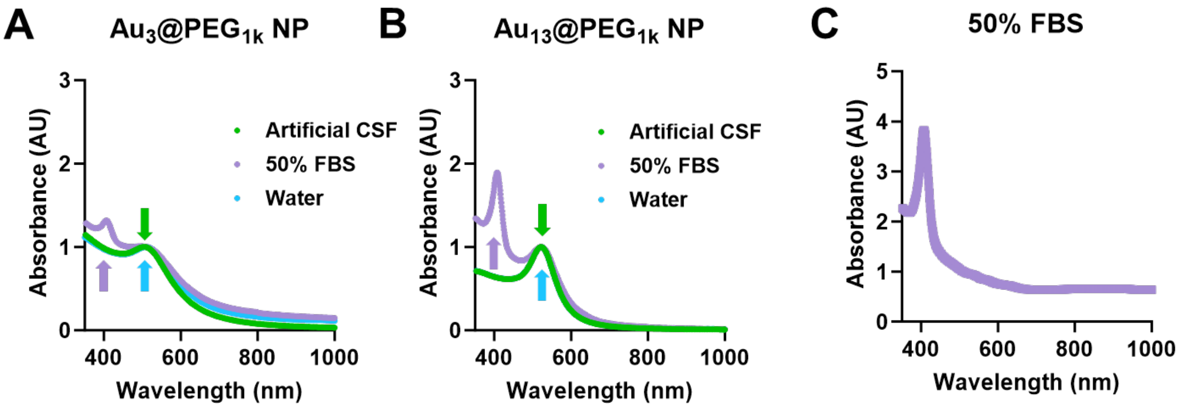


**Figure S5.** UV-vis spectra of $Au_x$@$PEG_y$ NPs after incubation in water, artificial CSF and 50% fetal bovine serum (FBS) at 37°C for 24 h. Blue arrow: Localized surface plasmon resonance (LSPR) peaks of NPs in water. Green arrow: Localized surface plasmon resonance (LSPR) peaks of NPs in artificial CSF. Purple arrow: Peak of 50% FBS. We confirmed the colloidal stability of $Au_x$@$PEG_y$ NPs in artificial CSF or 50% FBS. (A)–(B) The LSPR peak of $Au_x$@$PEG_y$ NPs at 509 and 522 nm (characteristic LSPR peak wavelengths of stable 3-nm and 13-nm gold NPs, respectively) did not change drastically and no peak of NP aggregation in the 600–1000 nm range was observed upon incubation in serum. (C) UV-vis spectrum of blank 50% FBS in PBS without Au NPs. Strong protein peaks at ~400 nm resemble the ~400 nm peaks in (A) and (B). Note that the LSPR peaks of $Au_x$@$PEG_y$ NPs in the plasma of Week 10 R6/2 mice were not detectable by UV-vis spectrophotometry due to the strong absorption of the plasma.

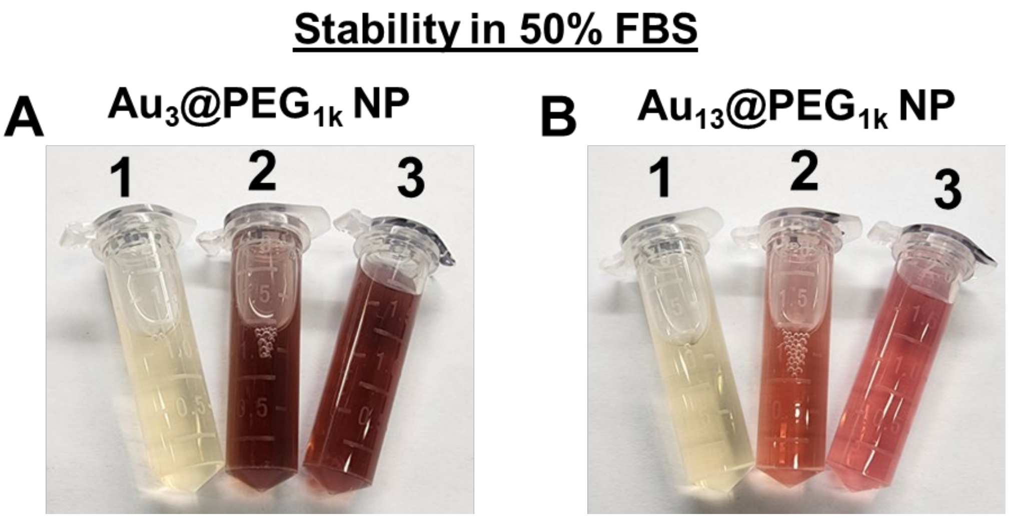


**Figure S6.** (A) Photographs of $Au_3$@$PEG_{1k}$ NP upon incubation in 50% FBS. (**1**) 50% FBS 24 h post-incubation at 37 ºC. (**2**) $Au_3$@$PEG_{1k}$ NP in 50% FBS 24 h post-incubation at 37 ºC. (**3**) $Au_3$@$PEG_{1k}$ NP in water. There was no significant color change of $Au_3$@$PEG_{1k}$ NP in FBS, indicating colloidal stability. (B) Photographs of $Au_{13}$@$PEG_{1k}$ NP upon incubation in 50% FBS. (**1**) 50% FBS 24 h post-incubation at 37 ºC. (**2**) $Au_{13}$@$PEG_{1k}$ NP in 50% FBS 24 h post-incubation at 37 ºC. (**3**) $Au_{13}$@$PEG_{1k}$ NP in water. There was no significant color change of $Au_{13}$@$PEG_{1k}$ NP in FBS.

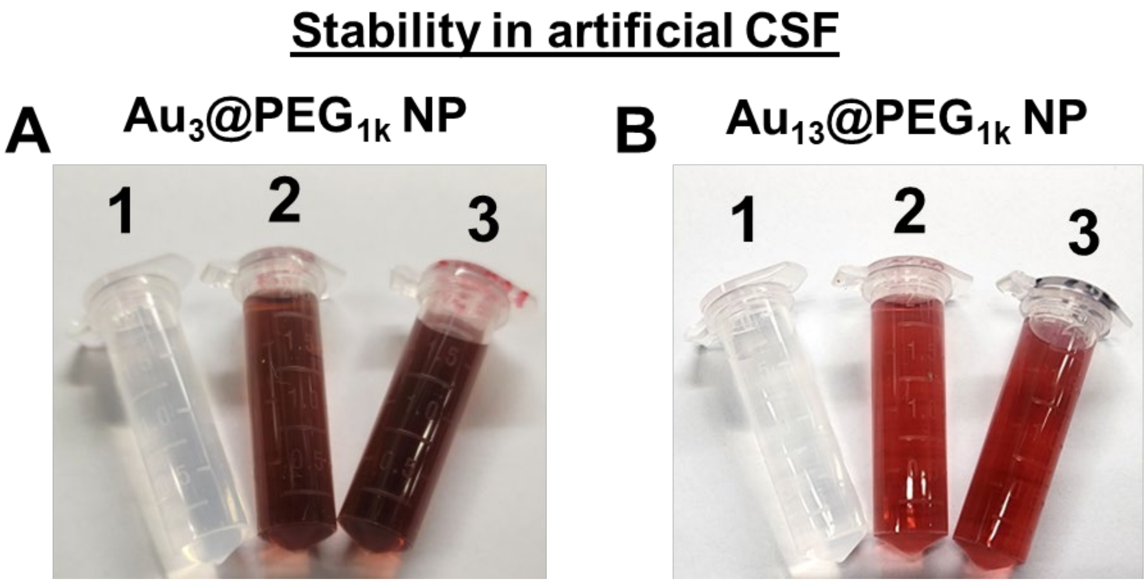


**Figure S7.** (A) Photographs of $Au_3@PEG_{1k}$ NP upon incubation in artificial CSF. (**1**) Artificial CSF 24 h post-incubation at 37 ºC. (**2**) $Au_3@PEG_{1k}$ NP in artificial CSF 24 h post-incubation at 37 ºC. (**3**) $Au_3@PEG_{1k}$ NP in water. There was no significant color change of $Au_3@PEG_{1k}$ NP in artificial, indicating colloidal stability. (B) Photographs of $Au_{13}@PEG_{1k}$ NP upon incubation in artificial CSF. (**1**) Artificial CSF 24 h post-incubation at 37 ºC. (**2**) $Au_{13}@PEG_{1k}$ NP in artificial CSF 24 h post-incubation at 37 ºC. (**3**) $Au_{13}@PEG_{1k}$ NP in water. There was no significant color change of $Au_{13}@PEG_{1k}$ NP in artificial CSF.

# Appendix III: In Vitro Cellular Uptake

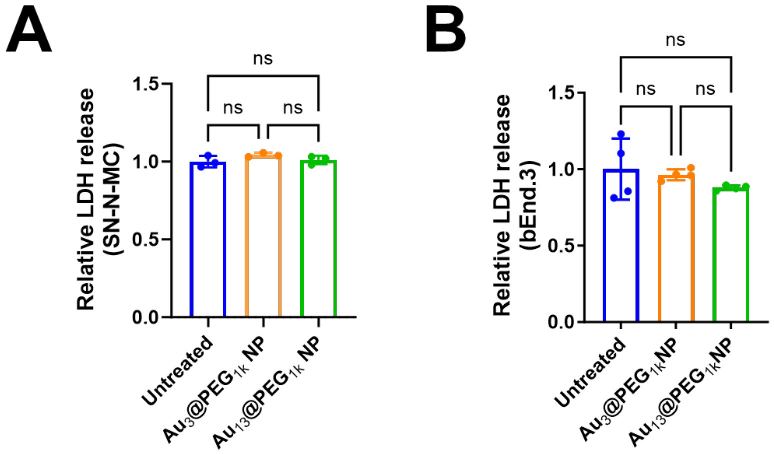


**Figure S8.** *In vitro* cell viability of $Au_x@PEG_y$ NP on (A) SK-N-MC and (B) bEnd.3 cells by the LDH assay. Both cell types were incubated with either 50 µg/mL $Au_3@PEG_{1k}$ NP (orange) or $Au_{13}@PEG_{1k}$ NP (green) for 24 h; untreated cells served as controls (blue). The amount of LDH released was normalized with that in the untreated cells. Data are from n = 3–4, across 1 experiment. Statistical significance was evaluated using One-Way ANOVA with Tukey's post hoc test for

multiple comparisons. ns = no significant difference. All bars and error bars represent mean ± SD. $Au_x@PEG_y$ NP was not cytotoxic to both cell types.

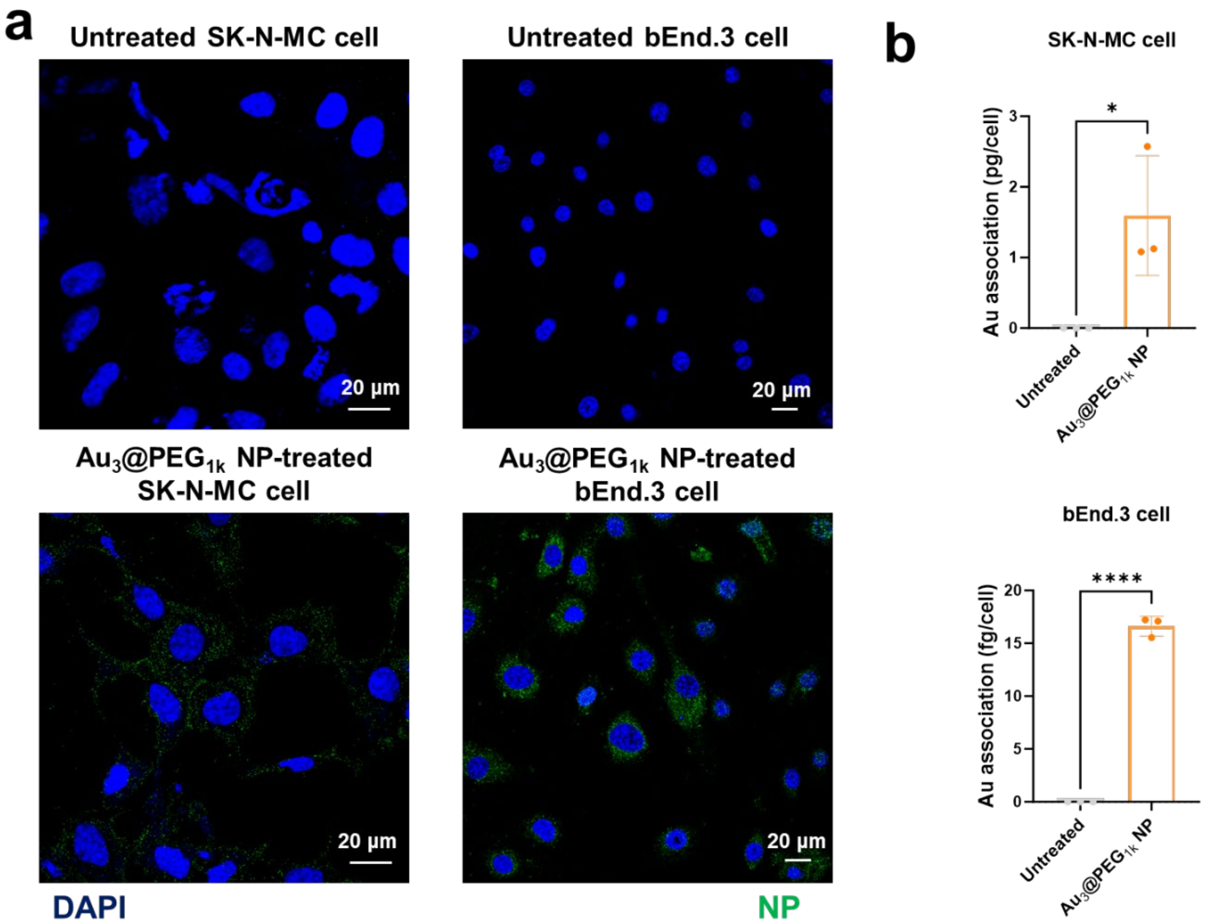


**Figure S9.** *In vitro* uptake of $Au_3@PEG_{1k}$ NPs by bEnd.3 brain endothelial cells and 89-polyglutamine-repeats (Q89)-expressing SK-N-MC neurons (the model cell type of HD). Cells were incubated with 50 μg/mL $Au_3@PEG_{1k}$ NPs for 24 h, and untreated HD cells served as controls. (A) Confocal reflectance images showed the entry of $Au_3@PEG_{1k}$ NPs (green) to cells. Blue: nucleus. (B) ICP-MS measurements verified the association of $Au_3@PEG_{1k}$ NPs (orange) with cells. Data are from n = 3, across 1 experiment. Statistical significance was evaluated using unpaired Student's t-test. *$P < 0.05$; ****$P < 0.0001$. All bars and error bars represent mean ± SD.

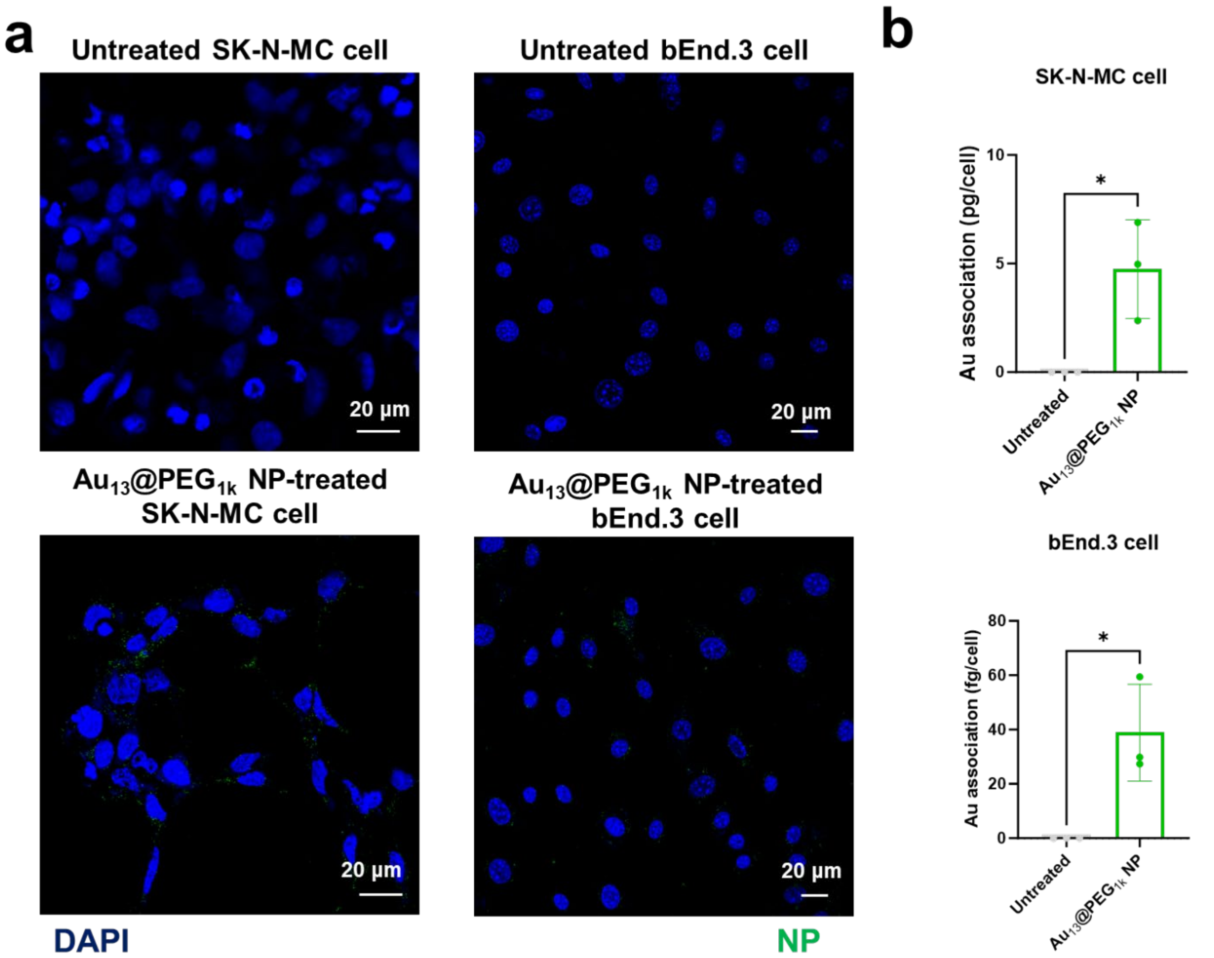


**Figure S10.** *In vitro* uptake of $Au_{13}$@$PEG_{1k}$ NPs by bEnd.3 brain endothelial cells and 89-polyglutamine-repeats (Q89)-expressing SK-N-MC cells (the model cell type of HD). Cells were incubated with 50 µg/mL $Au_{13}$@$PEG_{1k}$ NPs for 24 h, and untreated HD cells served as controls. (A) Confocal reflectance images showed the entry of $Au_{13}$@$PEG_{1k}$ NPs (green) to cells. Blue: nucleus. (B) ICP-MS measurements verified the association of $Au_{13}$@$PEG_{1k}$ NPs (green) with cells. Data are from n = 3, across 1 experiment. Statistical significance was evaluated using unpaired Student's t-test. $*P < 0.05$. All bars and error bars represent mean ± SD.

# Appendix IV: Organ/Tissue-level Distribution and Nanoparticle Stability

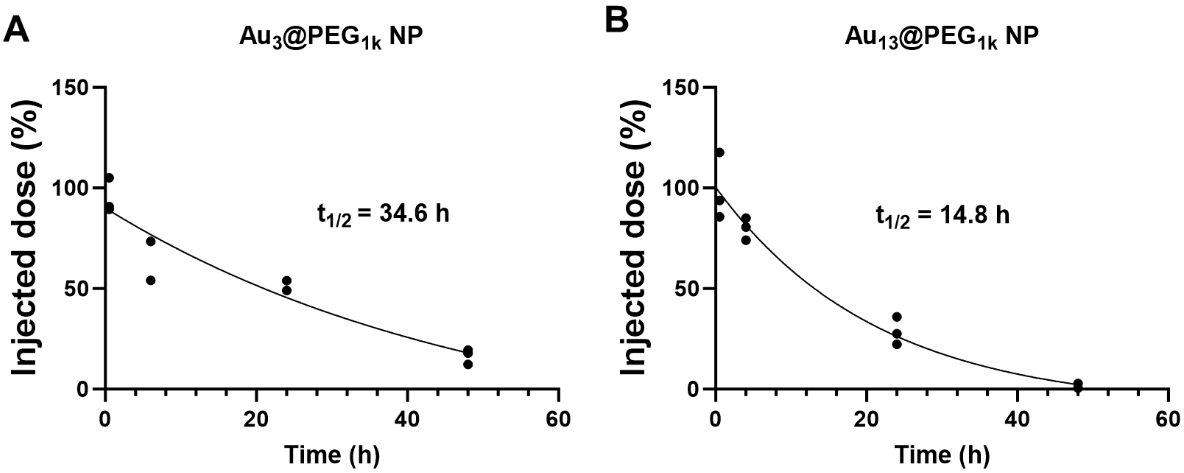


**Figure S11.** Blood pharmacokinetics of $Au_x@PEG_y$ NP upon an i.v. injection into Week 10 R6/2 HD mice. ICP-MS measurements revealed that (A) $Au_3@PEG_{1k}$ NP showed more prolonged blood circulation than (B) $Au_{13}@PEG_{1k}$ NP. Blood half-life time was calculated using one phase elimination model. Data are from n = 2–3, across 1 experiment.

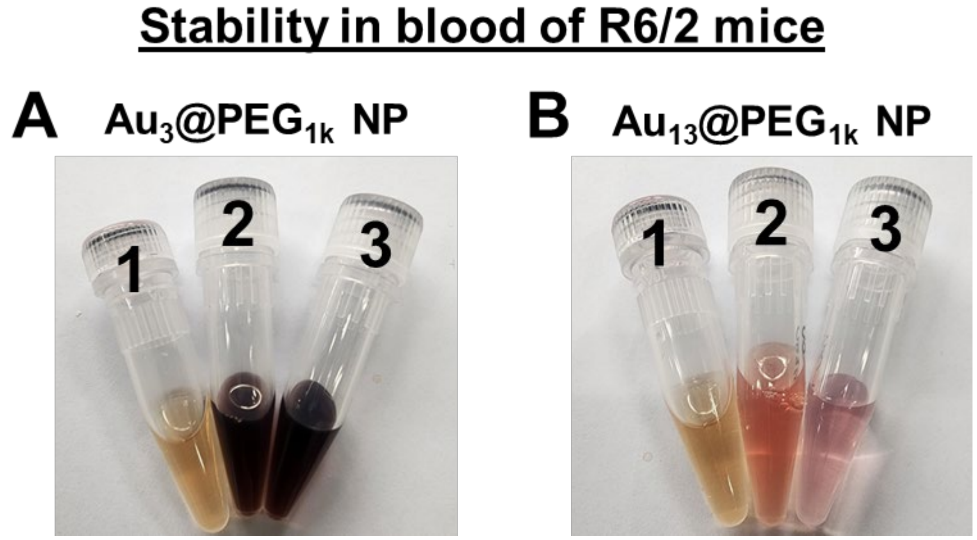


**Figure S12.** Photographs of $Au_3@PEG_{1k}$ NP upon injection into Week 10 R6/2 HD mice. (A) (**1**) Plasma of uninjected mice is yellow. (**2**) $Au_3@PEG_{1k}$ NP in plasma 24 h post-injection; there was no drastic change in NP color. (**3**) $Au_3@PEG_{1k}$ NP in water. (B) (**1**) Plasma of uninjected mice is yellow. (**2**) $Au_{13}@PEG_{1k}$ NP in plasma 24 h post-injection; there was no drastic change in NP color, indicating colloidal stability. (**3**) $Au_3@PEG_{1k}$ NP in water.

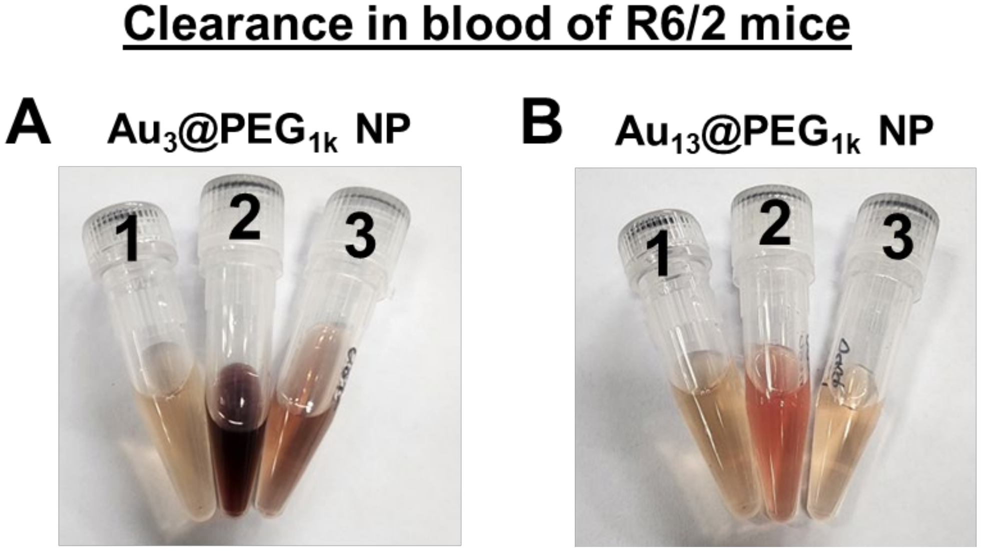


**Figure S13.** (A) Photographs of $Au_3@PEG_{1k}$ NP upon injection in Week 10 R6/2 HD mice. (**1**) Plasma of uninjected mice is yellow. (**2**) $Au_3@PEG_{1k}$ NP in plasma 24 h post-injection; there was no drastic change in NP color. (**3**) $Au_3@PEG_{1k}$ NP in plasma 48 h post-injection; the paler color suggests pronounced *in vivo* NP clearance from blood, a result consistent with the blood half-life of $Au_3@PEG_{1k}$ NPs (~35 h). (B) Photographs of $Au_{13}@PEG_{1k}$ NP upon injection in Week 10 R6/2 HD mice. (**1**) Plasma of uninjected mice is yellow. (**2**) $Au_{13}@PEG_{1k}$ NP in plasma 24 h post-injection; there was no drastic change in NP color, indicating colloidal stability. (**3**) $Au_{13}@PEG_{1k}$ NP in plasma 48 h post-injection; the vanished red color suggests near complete *in vivo* NP clearance from blood, a result consistent with the blood half-life of $Au_{13}@PEG_{1k}$ NPs (~15 h).

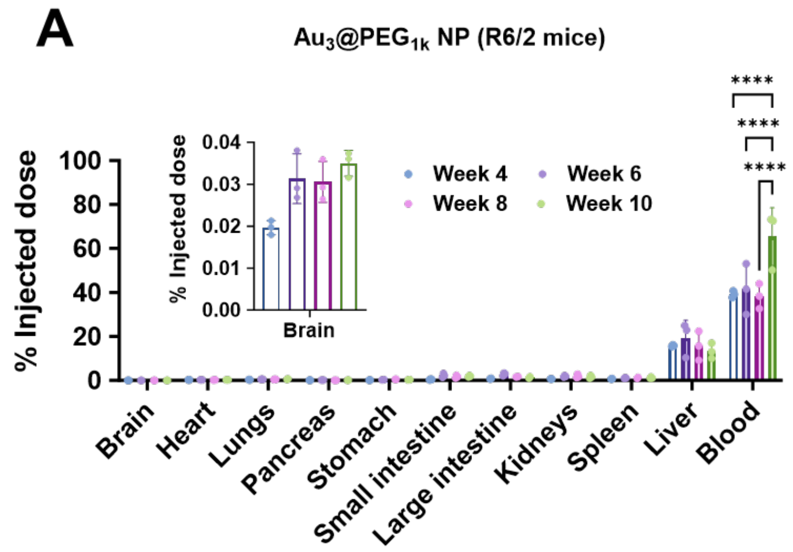
A
$Au_3@PEG_{1k}$ NP (R6/2 mice)
% Injected dose
% Injected dose
Brain
Week 4
Week 6
Week 8
Week 10
Brain
Heart
Lungs
Pancreas
Stomach
Small intestine
Large intestine
Kidneys
Spleen
Liver
Blood
****
****
****

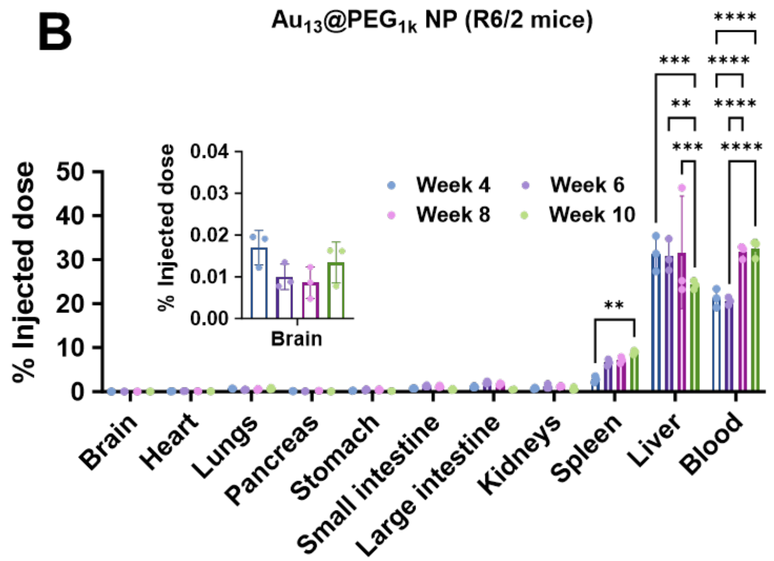
B
$Au_{13}@PEG_{1k}$ NP (R6/2 mice)
% Injected dose
% Injected dose
Brain
Week 4
Week 6
Week 8
Week 10
Brain
Heart
Lungs
Pancreas
Stomach
Small intestine
Large intestine
Kidneys
Spleen
Liver
Blood
**
***
**
***
****
****
****
****

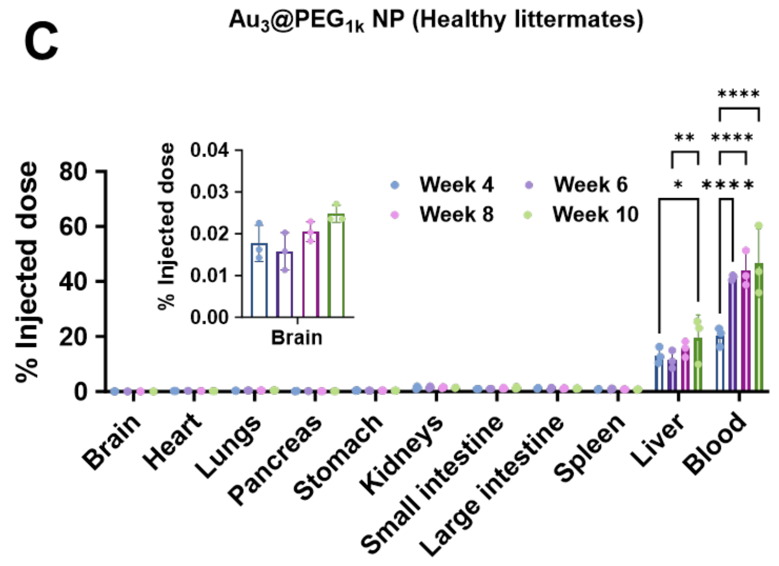
C
$Au_3@PEG_{1k}$ NP (Healthy littermates)
% Injected dose
% Injected dose
Brain
Week 4
Week 6
Week 8
Week 10
Brain
Heart
Lungs
Pancreas
Stomach
Kidneys
Small intestine
Large intestine
Spleen
Liver
Blood
**
*
****
****
****

**Figure S14.** Organ-level distribution of (A) $Au_3$@$PEG_{1k}$ NP and (B) $Au_{13}$@$PEG_{1k}$ NP upon i.v. injection into Week 4 (blue), 6 (purple), 8 (pink) and 10 (green) R6/2 HD mice 24 h post-injection. (C) Organ-level distribution of $Au_3$@$PEG_{1k}$ NP upon i.v. injection into age-matched healthy littermates. The bulk gold content in each organ was detected using ICP-MS in terms of % injected dose. Data are from n = 3, across 6 experiments. Statistical significance was evaluated using Two-Way ANOVA with Tukey's post hoc test for multiple comparisons. **P* < 0.05; ***P* < 0.01; ****P* < 0.001; *****P* < 0.0001. All bars and error bars represent mean ± SD.

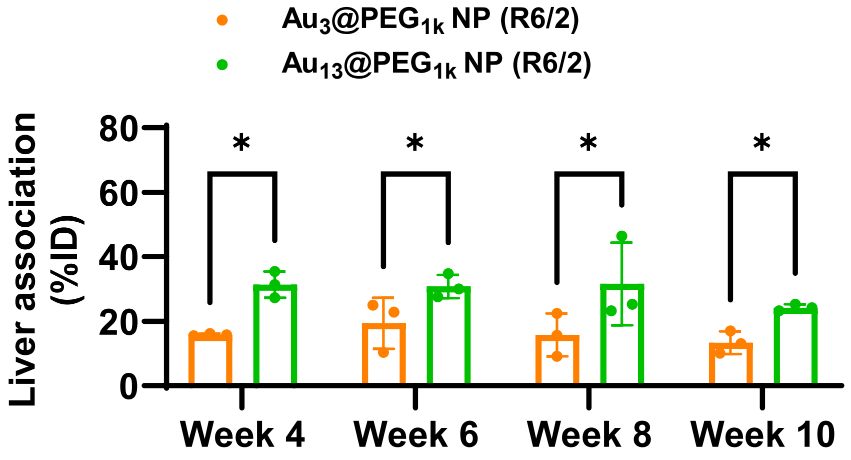


**Figure S15.** Liver association of $Au_3$@$PEG_{1k}$ (orange) and $Au_{13}$@$PEG_{1k}$ (green) NPs in R6/2 HD mice 24 h post-i.v. injection. A smaller NP size yields less liver association for all ages of HD mice tested. The bulk gold content in the liver was detected using ICP-MS in terms of % injected dose. Data are from n = 3, across 6 experiments. Statistical significance was evaluated using Student's t-test. **P* < 0.05. All bars and error bars represent mean ± SD.

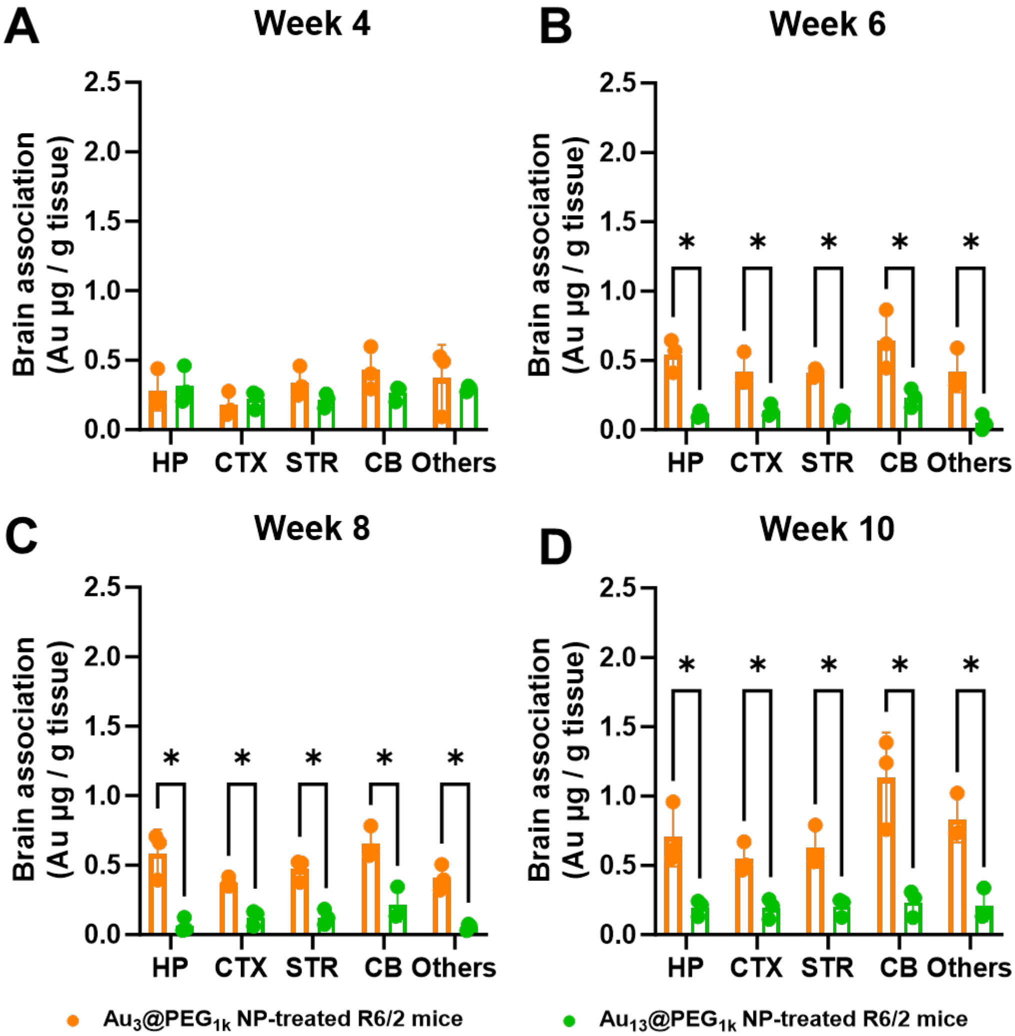


**Figure S16.** Delivery of $Au_3@PEG_{1k}$ NP (orange) and $Au_{13}@PEG_{1k}$ NP (green) to the brain of R6/2 HD mice as a function of disease stage 24 h post-injection. Hippocampus (HP), cortex (CTX), striatum (CTX), cerebellum (CB) and other regions (Others). (A) At the age of Week 4, there was no drastic difference between both NP sizes across different brain regions. Data are from n = 3, across 1 experiment. (B, C, D) At the ages of Weeks 6, 8, and 10, there was more abundant uptake of $Au_3@PEG_{1k}$ NPs than $Au_{13}@PEG_{1k}$ NPs across all brain regions. Data are from n = 3, across 1 experiment. All the statistical significance was evaluated using unpaired Student's t-test. As the HD disease stage progressed, uptake of $Au_3@PEG_{1k}$ NPs in the brain of R6/2 mice increased. $*P < 0.05$. All bars and error bars represent mean ± SD.

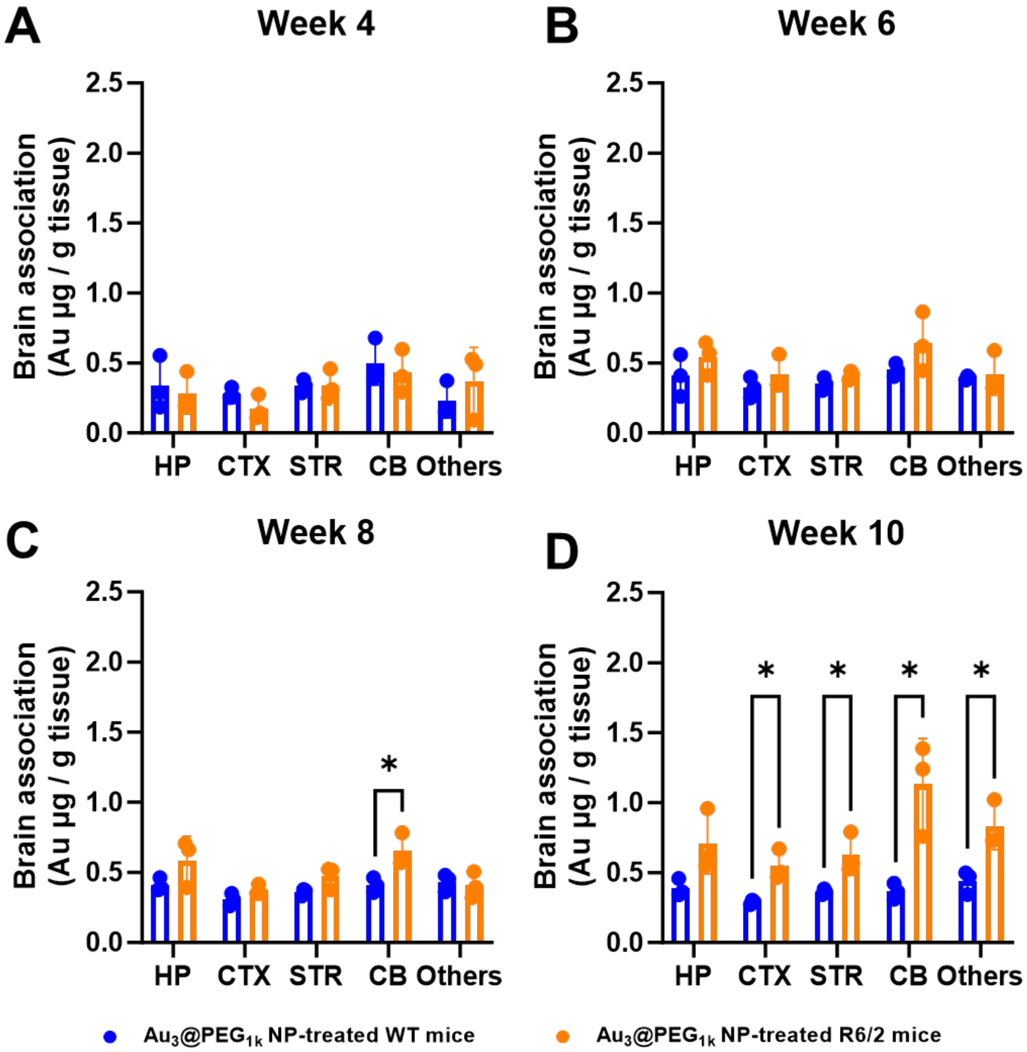


**Figure S17.** Delivery of $Au_3@PEG_{1k}$ NP to the brain of R6/2 HD mice (orange) or control healthy littermates (WT; blue) as a function of disease stage 24 h post-injection. Hippocampus (HP), cortex (CTX), striatum (CTX), cerebellum (CB) and other regions (Others). (A, B, C) At the age of Weeks 4, 6, and 8, there was no drastic difference between both mouse strains across different brain regions (except for CB at Week 8). Data are from n = 3, across 1 experiment. (D), At the age of Week 10, there was more abundant NP uptake in the CTX, STR, CB, Others of R6/2 mice than WT mice. Data are from n = 3, across 1 experiment. All the statistical significance was evaluated using unpaired Student's t-test. As HD disease stage progressed, uptake of $Au_3@PEG_{1k}$ NPs in the brain of R6/2 mice increased. *$P < 0.05$. All bars and error bars represent mean ± SD.

# Appendix V: Transport Mechanism of the Nanoparticle

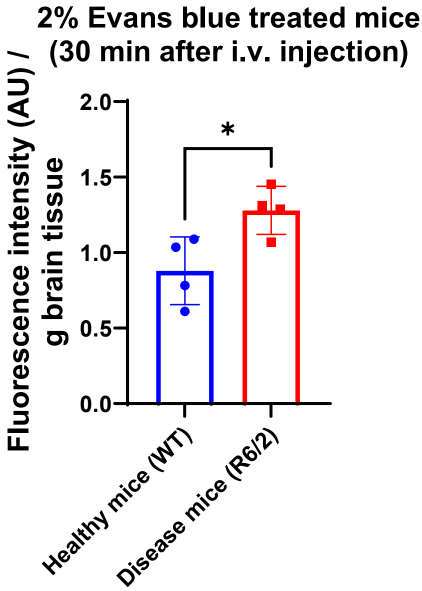


**Figure S18.** Permeability of the blood-brain barrier of uninjected healthy littermates (WT; blue) and Week 10 R6/2 HD mice (red). At the age of Week 10, mice were i.v. injected with Evans blue and sacrificed 30 min post-injection. There were slightly stronger fluorescence signals in the brain lysate of R6/2 mice than WT mice. Data are from n = 4, across 2 experiments. The statistical significance was evaluated using unpaired Student's t-test. $*P < 0.05$. All bars and error bars represent mean ± SD.

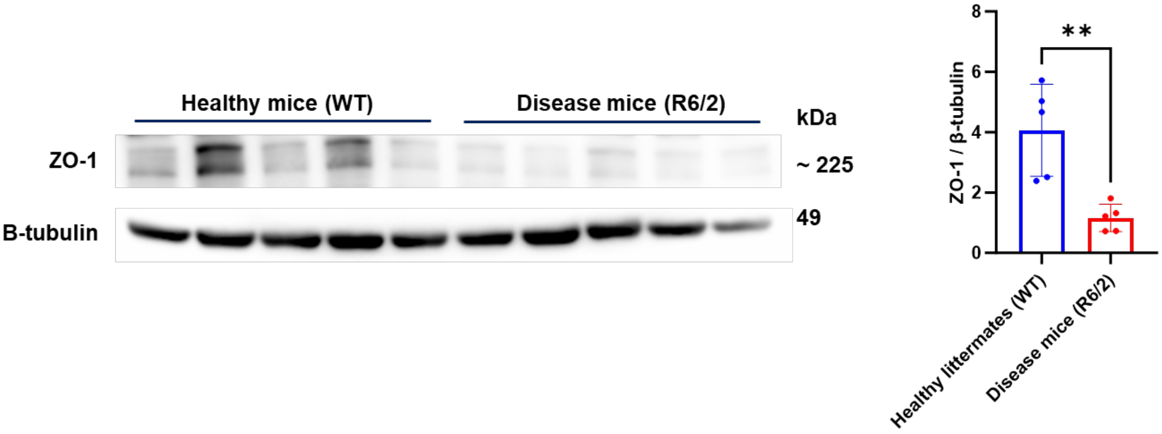


**Figure S19.** Western blot analysis revealed the downregulation of the tight junction protein ZO-1 in the whole brain of Week 10 R6/2 disease mice (red) when compared to age-match, healthy wildtype (WT) littermates (blue). Data are from n = 5, across one experiment. Statistical significance was evaluated using Student's t-test. $**P < 0.01$. All bars and error bars represent mean ± SD.

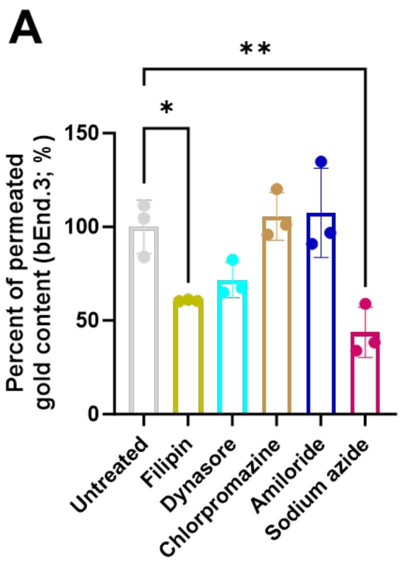


**List of pharmacological inhibitors**

| Target | Blocker | Final concentration |
|---|---|---|
| Lipid raft/caveolae | Filipin | 10 μg/mL |
| Dynamin | Dynasore | 13 μg/mL |
| Clathrin | Chlorpromazine | 5 μg/mL |
| Macropinocytosis | Amiloride | 500 μg/mL |
| Energy dependent | Sodium azide | 3.3 mg/mL |

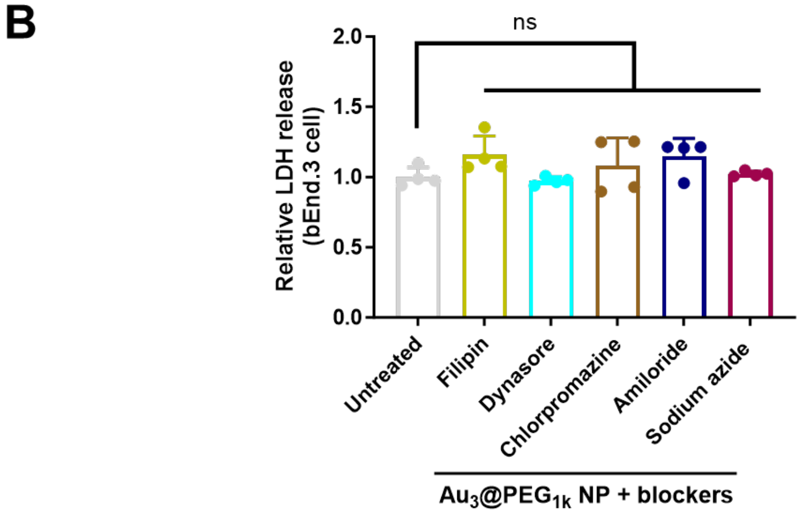


**Figure S20.** Role of active cellular transport in the penetration of $Au_3$@$PEG_{1k}$ NPs across an advanced *in vitro* flow-based BBB model seeded with human ESC-induced brain microvascular endothelial cells (iBMECs) in an IBAC M1 microchip. (A) Schematic illustration of IBAC M1, a gravity-driven, fluid-flow, pumpless, high-throughput microchip of the BBB. (B) After pretreating iBMECs with pharmacological blockers of various active cellular uptake pathways [filipin or sodium azide] for 1 h, the cells were further treated with medium containing both 400 µg/mL NPs and blockers for 3 more h. Pretreatment with filipin and sodium azide drastically reduced NP transport across the *in vitro* brain endothelium, indicating caveolae-mediated transport. "100%" indicates the total amounts of NP penetrating through the BBB without the addition of pharmacological blockers. Data are from n = 3, across one experiment. (C) Cytotoxicity of the *in vitro* BBB endothelium upon treatment of various pharmacological blockers by the LDH assay. The amount of LDH released by NP-treated cells was normalized to that of untreated cells. Data are from n = 4, across one experiment. Statistical significance was evaluated using One-Way ANOVA with Dunnett's post hoc test for multiple comparisons. *$P < 0.05$; **$P < 0.01$; ns = no significant difference. All bars and error bars represent mean ± SD.

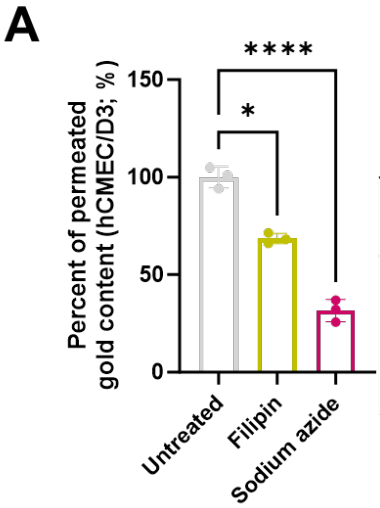


**List of pharmacological inhibitors**

| Target | Blocker | Final concentration |
|---|---|---|
| Lipid raft/caveolae | Filipin | 10 µg/mL |
| Energy dependent | Sodium azide | 3.3 mg/mL |

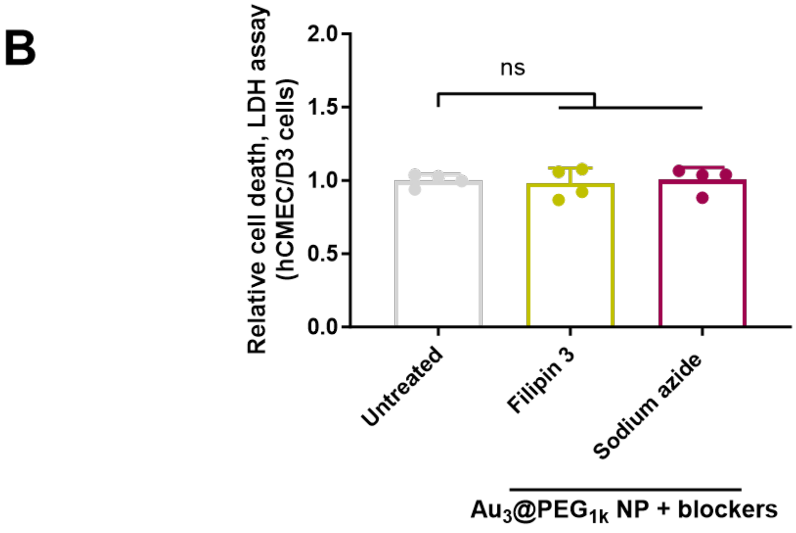


**Figure S21.** Role of active cellular transport in the penetration of $Au_3$@$PEG_{1k}$ NPs across a basic *in vitro* Transwell BBB model seeded with human hCMEC/D3 cells. (A) After pretreating hCMEC/D3 cells with pharmacological blockers of various active cellular uptake pathways [filipin or sodium azide] for 1 h, the cells were further treated with medium containing both 400 µg/mL NPs and blockers for 3 more h. Pretreatment with filipin and sodium azide drastically reduced NP transport across the *in vitro* brain endothelium, indicating caveolae-mediated transport. "100%" indicates the total amounts of NP penetrating through the BBB without the addition of pharmacological blockers. Data are from n = 3, across one experiment. (B) Cytotoxicity of the *in vitro* BBB endothelium upon treatment of various pharmacological blockers by the LDH assay. The amount of LDH released by NP-treated cells was normalized to that of untreated cells. Data are from n = 4, across one experiment. Statistical significance was evaluated using One-Way ANOVA with Dunnett's post hoc test for multiple comparisons. *$P < 0.05$; **$P < 0.01$; ns = no significant difference. All bars and error bars represent mean ± SD.

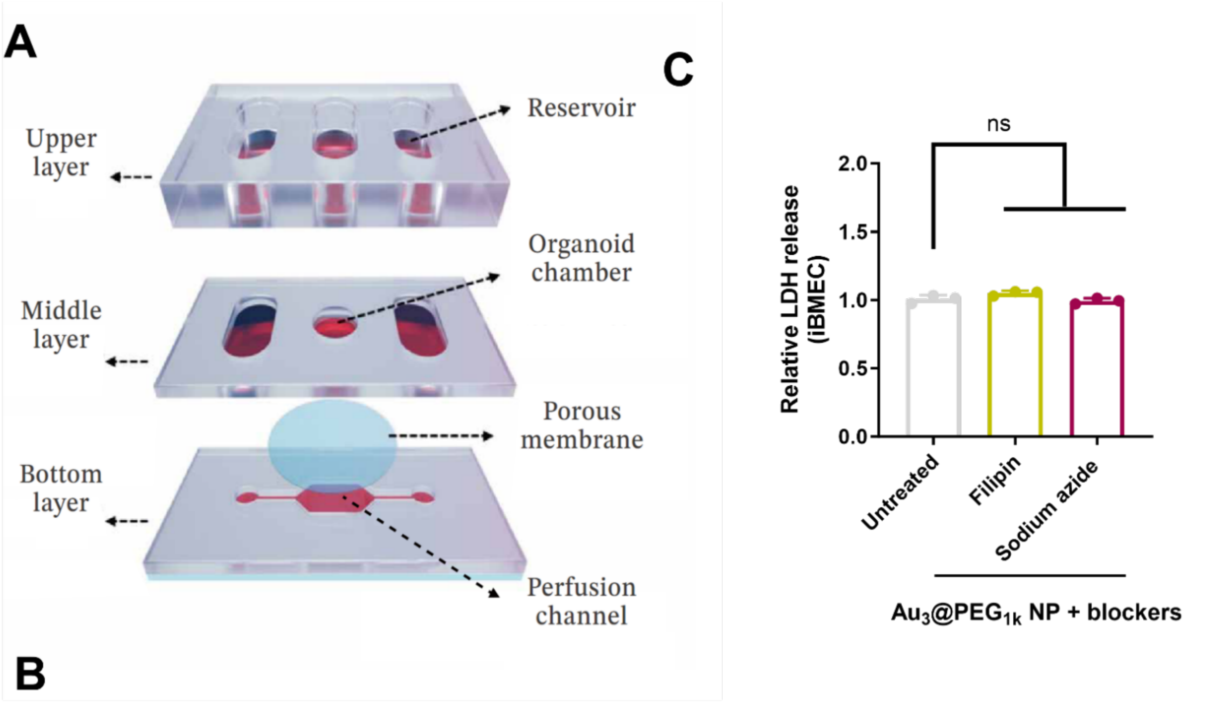


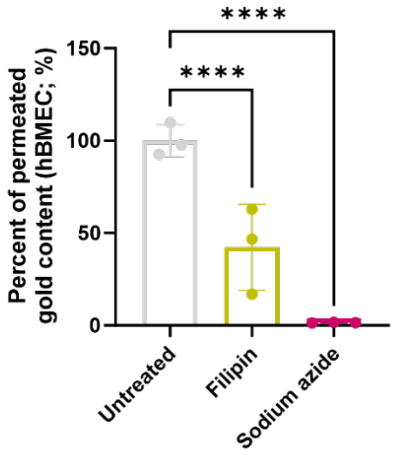


List of pharmacological inhibitors

| Target | Blocker | Final concentration |
|---|---|---|
| Lipid raft/caveolae | Filipin | 10 μg/mL |
| Energy dependent | Sodium azide | 3.3 mg/mL |

**Figure S22.** Role of active cellular transport in the penetration of Au$_3$@PEG$_{1k}$ NPs across an advanced *in vitro* flow-based BBB model seeded with human ESC-induced brain microvascular endothelial cells (iBMECs) in an IBAC M1 microchip. (A) Schematic illustration of IBAC M1, a gravity-driven, fluid-flow, pumpless, high-throughput microchip of the BBB. (B) After pretreating iBMECs with pharmacological blockers of various active cellular uptake pathways [filipin or sodium azide] for 1 h, the cells were further treated with medium containing both 400 μg/mL NPs and blockers for 3 more h. Pretreatment with filipin and sodium azide drastically reduced NP transport across the *in vitro* brain endothelium, indicating caveolae-mediated transport. "100%" indicates the total amounts of NP penetrating through the BBB without the addition of pharmacological blockers. Data are from n = 3, across one experiment. (C) Cytotoxicity of the *in vitro* BBB endothelium upon treatment of various pharmacological blockers by the LDH assay. The amount of LDH released by NP-treated cells was normalized to that of untreated cells. Data are from n = 4, across one experiment. Statistical significance was evaluated using One-Way ANOVA with Dunnett's post hoc test for multiple comparisons. *$P < 0.05$; **$P < 0.01$; ns = no significant difference. All bars and error bars represent mean ± SD.

# Appendix VI: Nanoparticle in the Brain Parenchyma

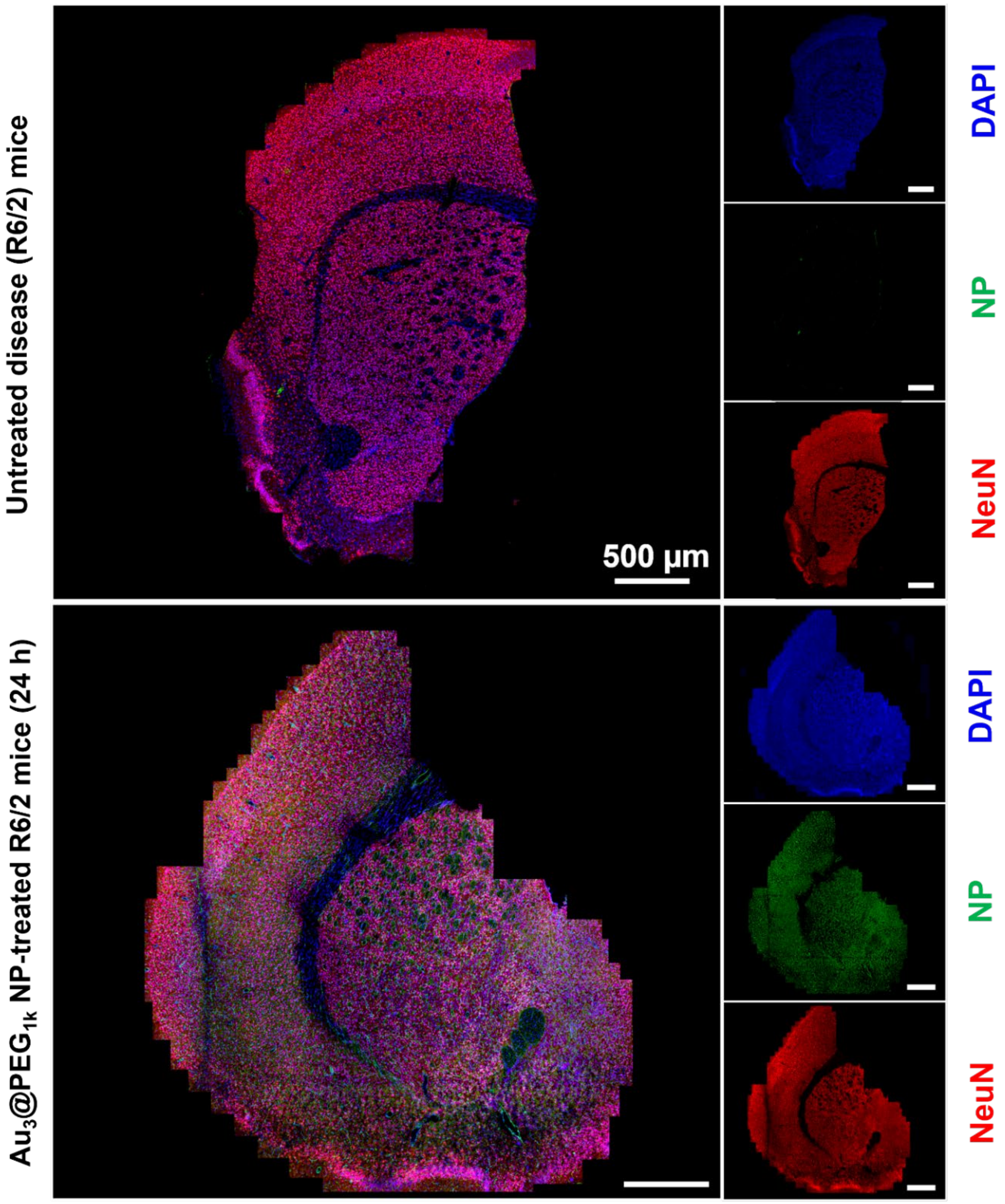


**Figure S23.** Large, stitched confocal reflectance images of the brain coronal cryosections of untreated Week 10 R6/2 HD mice and $Au_3$@$PEG_{1k}$ NP-treated Week 10 R6/2 mice 24 h post-i.v. injection. Left panel shows a large image stitched from ~200 individual images each taken with a 40× objective. Right panels show individual fluorescence or reflection channels. $Au_3$@$PEG_{1k}$ NP was distributed throughout the brain. Red: neuron (NeuN); Blue: nucleus (DAPI); Green: gold NP.

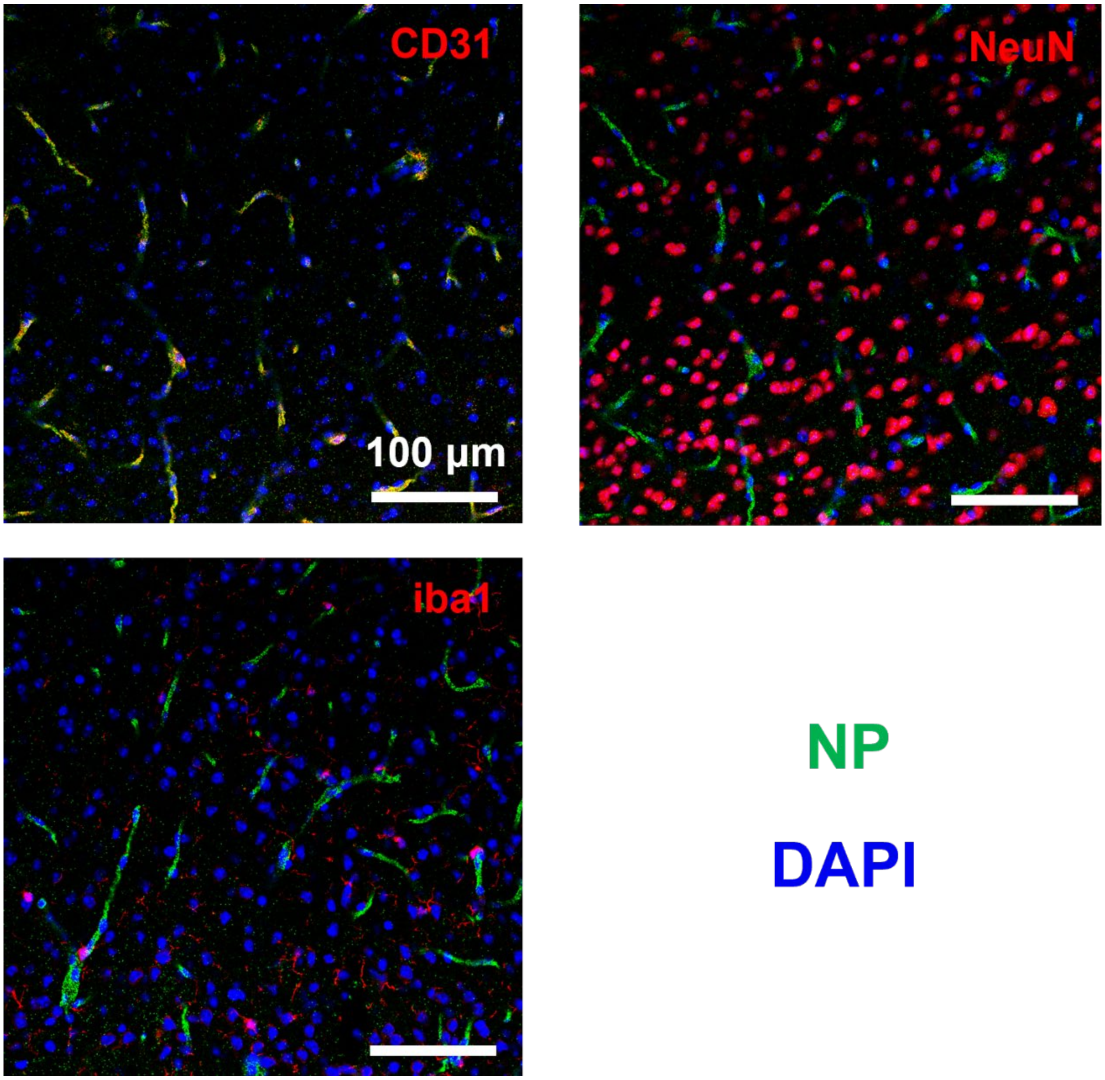


**Figure S24.** Confocal reflectance images of cortex (CTX) 24 h post-injection of $Au_3$@$PEG_{1k}$ NPs (green) into Week 10 R6/2 HD mice. Red: brain endothelial cells (CD31), neurons (NeuN), activated microglia (iba1); Blue: nucleus (DAPI). All NPs can enter the CTX of R6/2 HD mice.

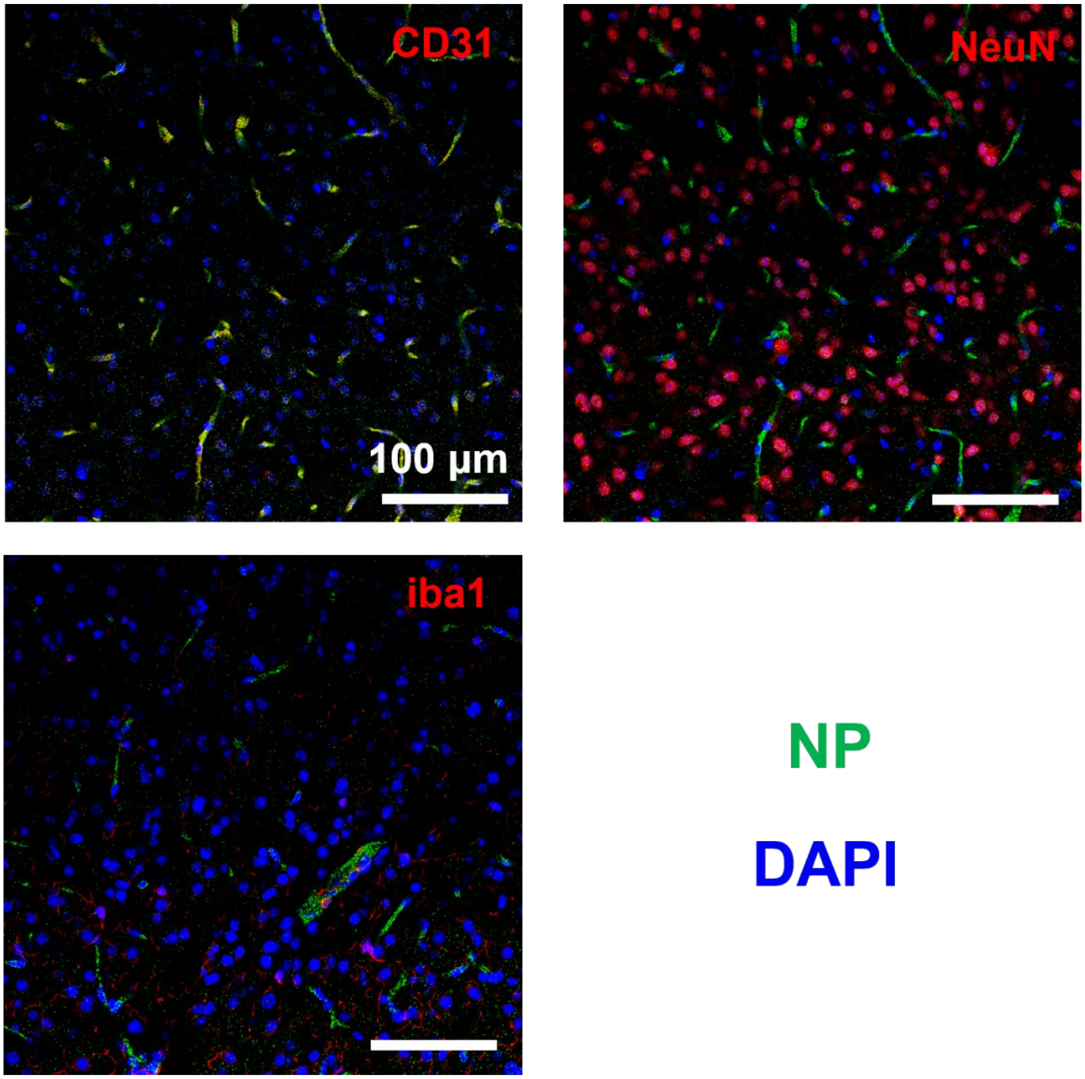


**Figure S25.** Confocal reflectance images of striatum (STR) 24 h post-injection of $Au_3@PEG_{1k}$ NPs (green) into Week 10 R6/2 HD mice. Red: brain endothelial cells (CD31), neurons (NeuN), activated microglia (iba1); Blue: nucleus (DAPI). All NPs can enter the STR of R6/2 HD mice.

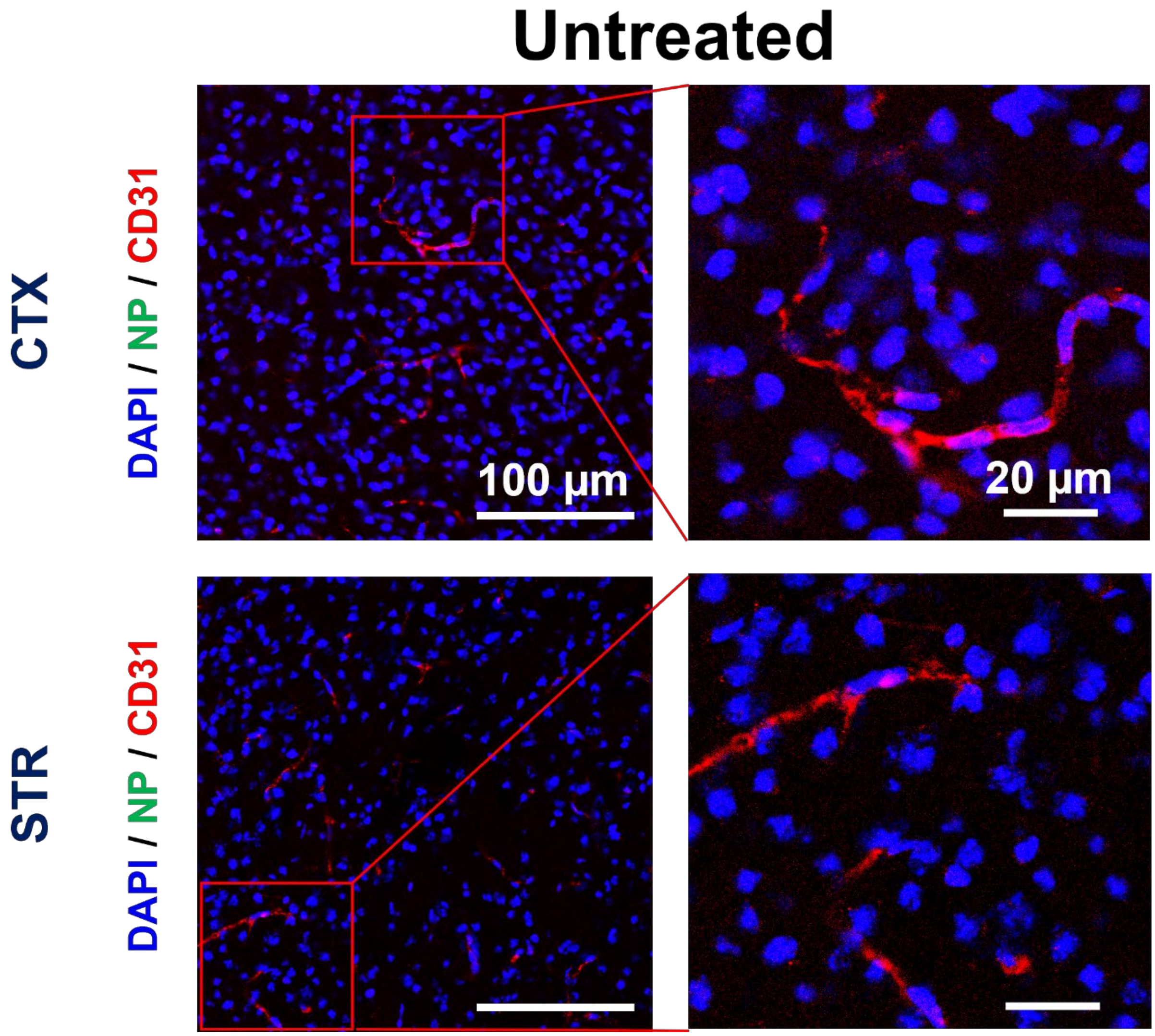


**Figure S26.** Confocal reflectance images of the cortex (CTX) and striatum (STR) of untreated R6/2 HD mice. The right column features the enlarged images of the boxed area in the left column. Red: brain endothelial cells (CD31); Blue: nucleus (DAPI); Green: gold NPs (green). No obvious non-specific signals arose from silver staining.

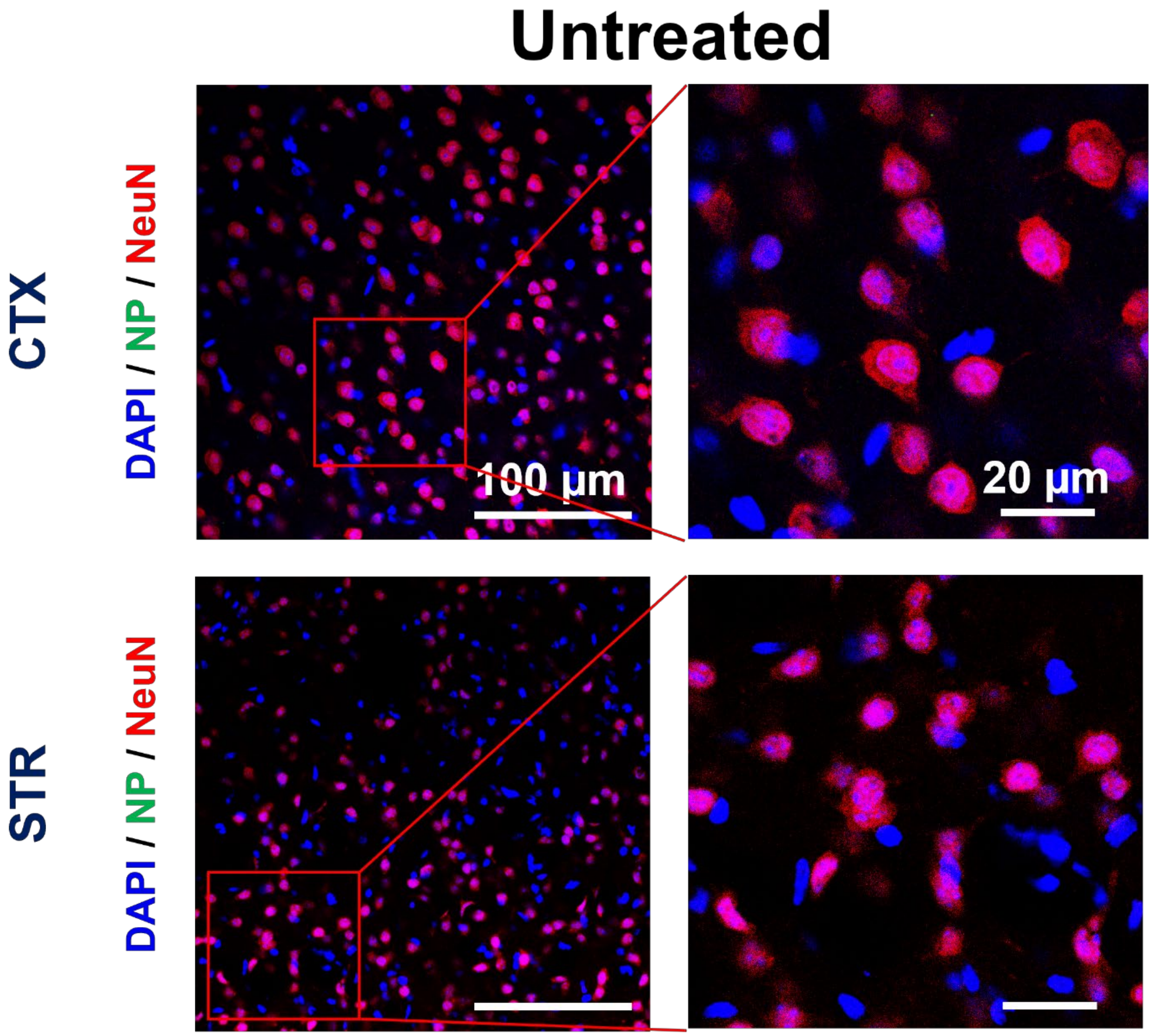


**Figure S27.** Confocal reflectance images of the cortex (CTX) and striatum (STR) of untreated R6/2 HD mice. The right column features the enlarged images of the boxed area in the left column. Red: neurons (NeuN); Blue: nucleus (DAPI); Green: gold NPs (green). No obvious non-specific signals arose from silver staining.

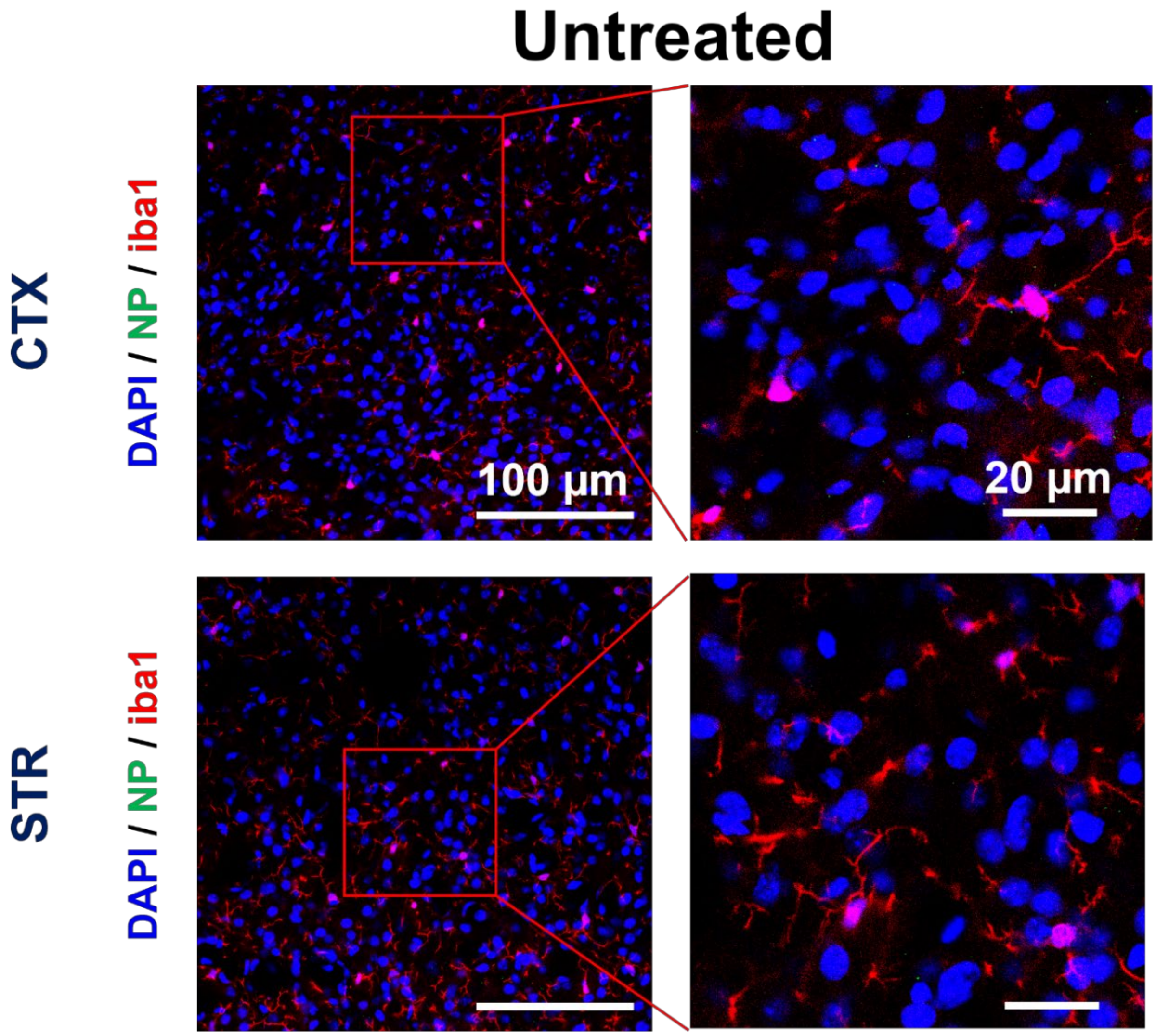

**Figure S28.** Confocal reflectance images of the cortex (CTX) and striatum (STR) of untreated R6/2 HD mice. The right column features the enlarged images of the boxed area in the left column. Red: activated microglia (iba1); Blue: nucleus (DAPI); Green: gold NPs (green). No non-specific signals arose from silver staining.

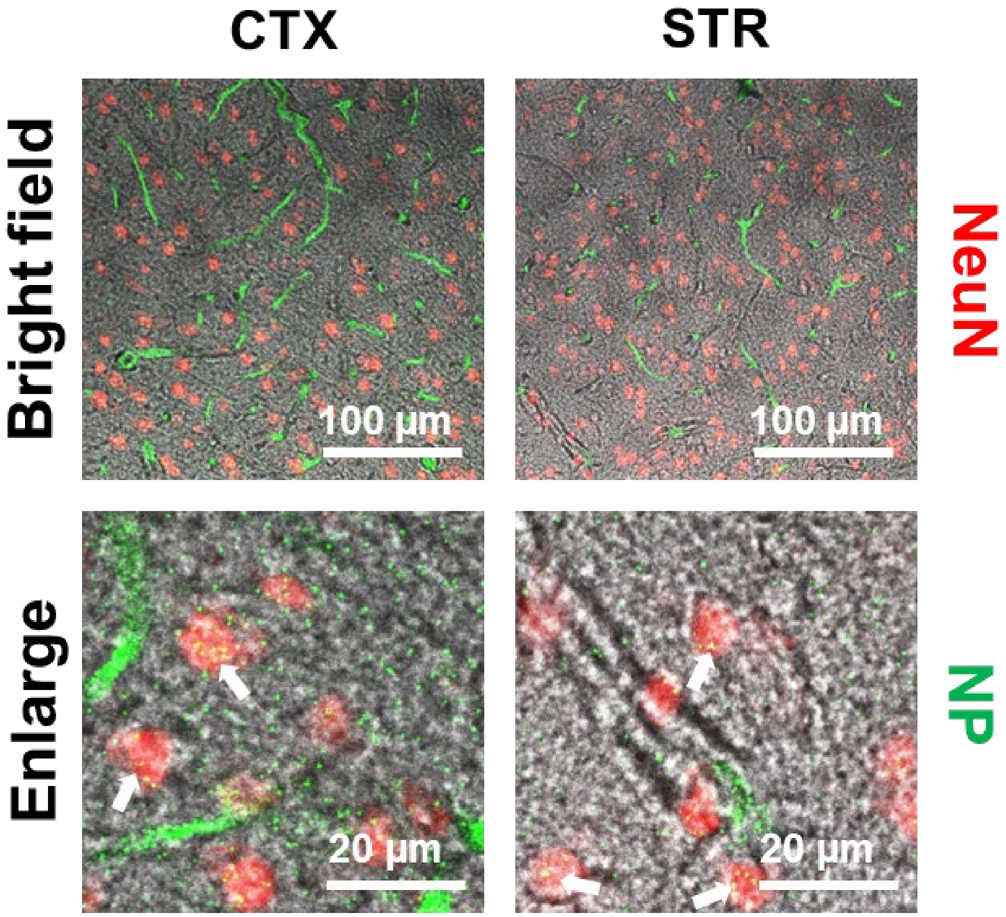


**Figure S29.** Confocal reflectance images (merged with bright field) of the cortex (CTX) and striatum (STR) of Week 10 R6/2 HD mice 24 h post-injection of $Au_3@PEG_{1k}$ NP (green). Red: neurons (NeuN) Blue: nucleus (DAPI). White arrows point to NPs inside NeuN+ neurons.

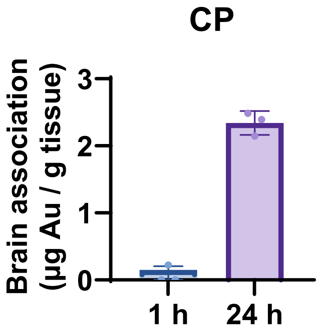


**Figure S30.** Delivery of $Au_3@PEG_{1k}$ NP to choroid plexus (CP) of Week 10 R6/2 HD mice 1 h or 24 h post-i.v. injection. Density of gold content in CP was detectable as early as 1 h post-injection and became higher 24 h post-injection. CP is the site of the blood-CSF barrier, so the detectable gold signals in CP suggest passage of gold NPs across or accumulation in the blood-CSF barrier. In terms of gold density in the brain tissue, CP is higher than other brain compartments, possibly due to the localized deposition of gold NP in the blood-CSF barrier and smaller mass of CP. Data are from n = 3, across two experiments. All bars and error bars represent mean ± SD.

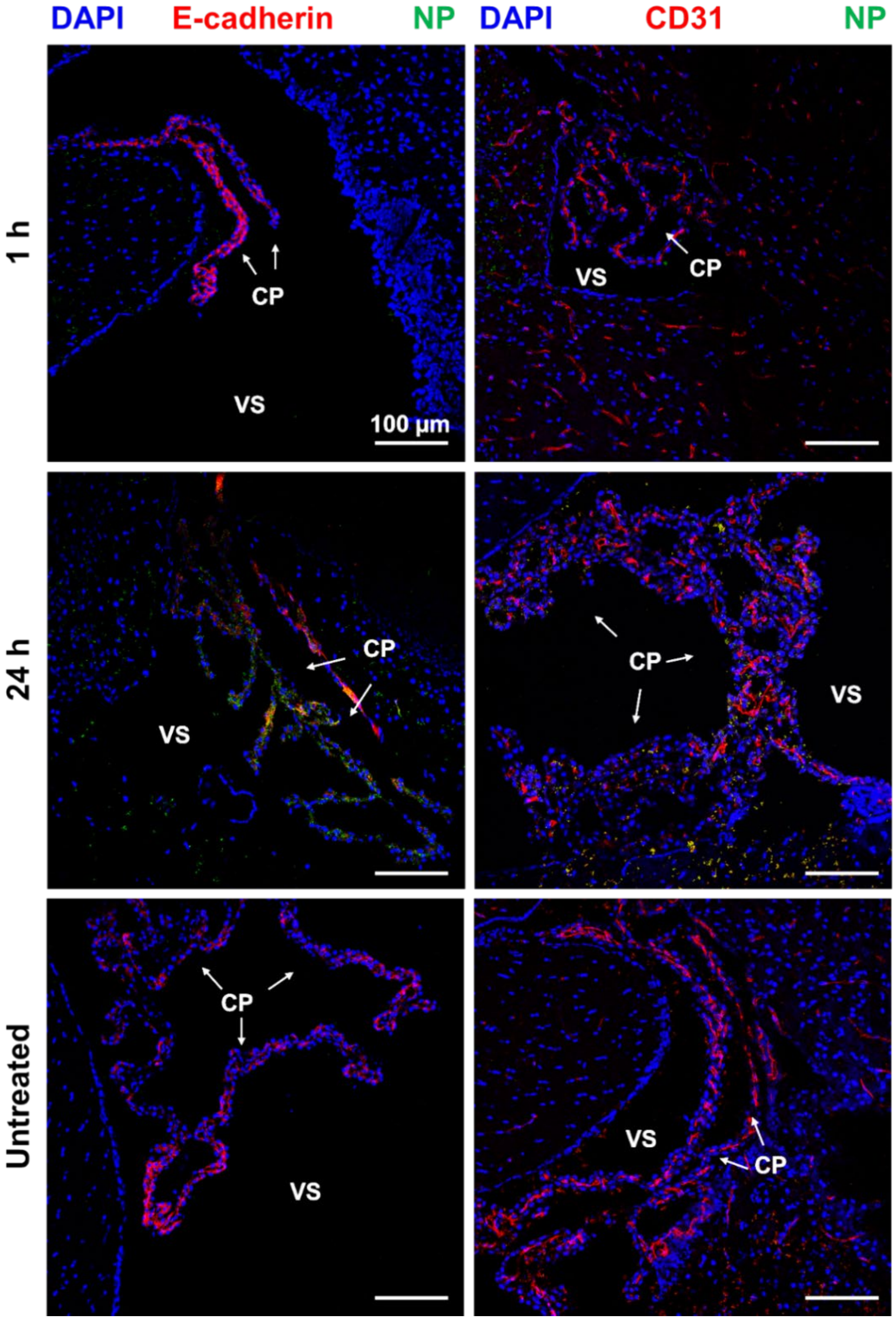


**Figure S31.** Confocal reflectance images of the sagittal brain cryosections of Week 10 R6/2 mice 1 h or 24 h post-i.v. injection of $Au_3$@$PEG_{1k}$ NP. (Left) Immunostaining of epithelial cells (E-cadherin; red), coupled with the villi shape of the structure, verified the location of choroid plexus (CP) and revealed the accumulation of $Au_3$@$PEG_{1k}$ NP (green) to the CP over time, suggesting NP filtration across the blood-CSF barrier. (Right) Immunostaining of endothelial cells (CD31; red) in the brain parenchyma adjacent to CP revealed the accumulation of $Au_3$@$PEG_{1k}$ NP (green), suggesting NP penetration of the BBB. These data suggest NP brain entry of $Au_3$@$PEG_{1k}$ NP via the blood-CSF barrier. Blue: nucleus (DAPI). VS = ventricular space.

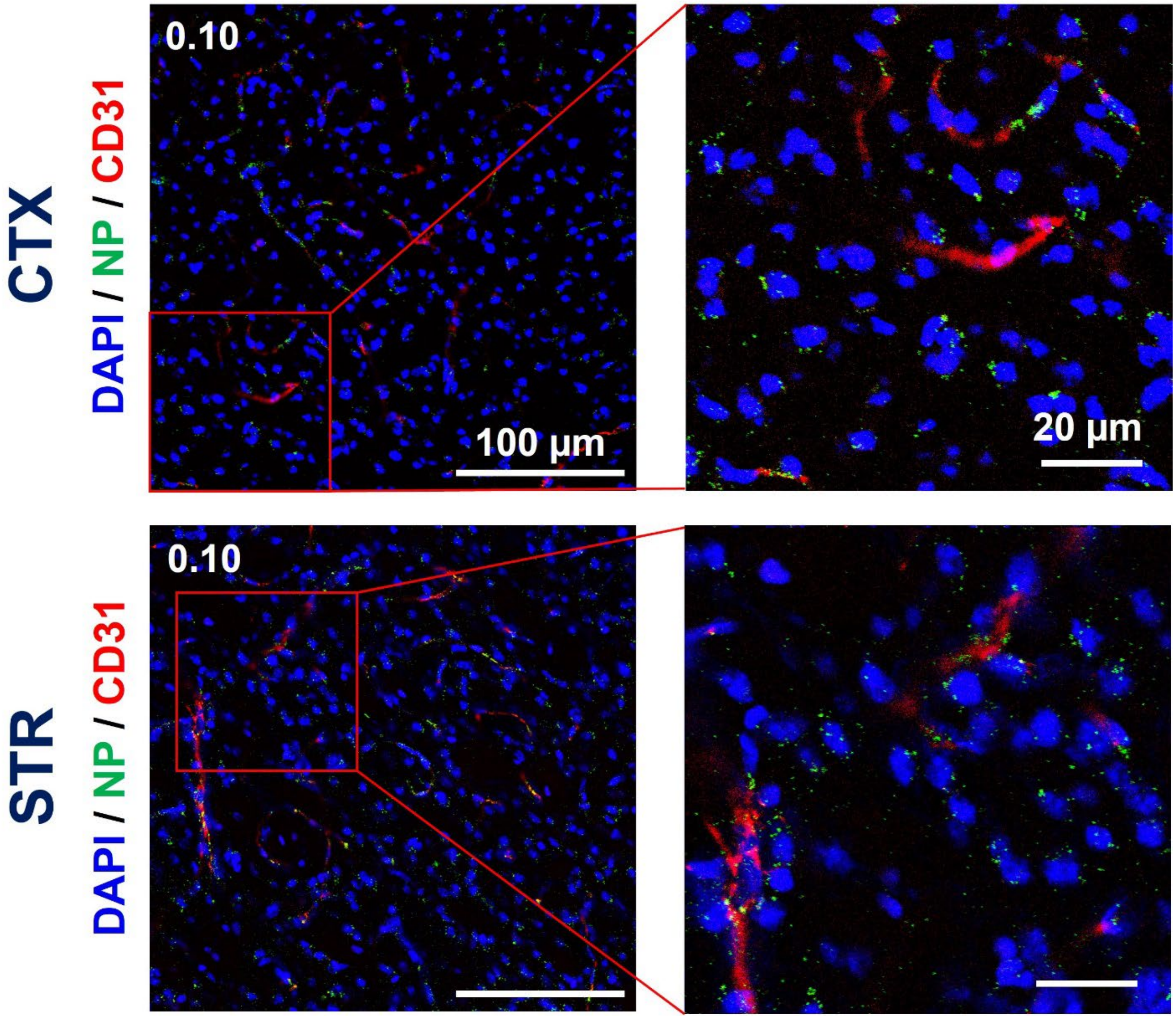


**Figure S32.** Confocal reflectance images of the cryosections of the cortex (CTX) and striatum (STR) of $Au_3@PEG_{1k}$ NP-treated R6/2 mice sacrificed at the point of efficacy evaluation (after five weekly injections) based on the schematic shown in **Figure 2A**. The right column features the enlarged images of the boxed area in the left column. Red: brain endothelial cells (CD31); Blue: nucleus (DAPI); Green: gold NPs (green).

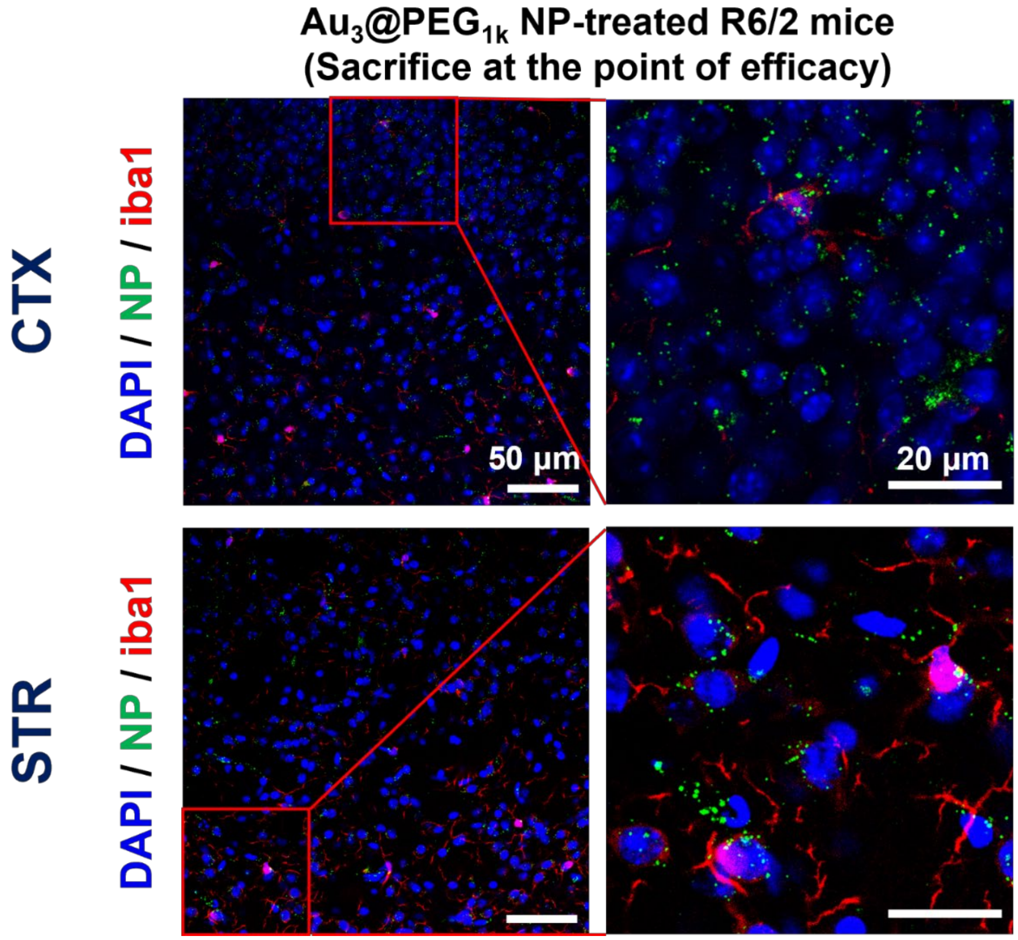


**Figure S33.** Confocal reflectance images of the cryosections of the cortex (CTX) and striatum (STR) of $Au_3@PEG_{1k}$ NP-treated R6/2 mice sacrificed at the point of efficacy evaluation (after five weekly injections) based on the schematic shown in **Figure 2A**. The right column features the enlarged images of the boxed area in the left column. Red: activated microglia (iba1); Blue: nucleus (DAPI); Green: gold NPs (green).

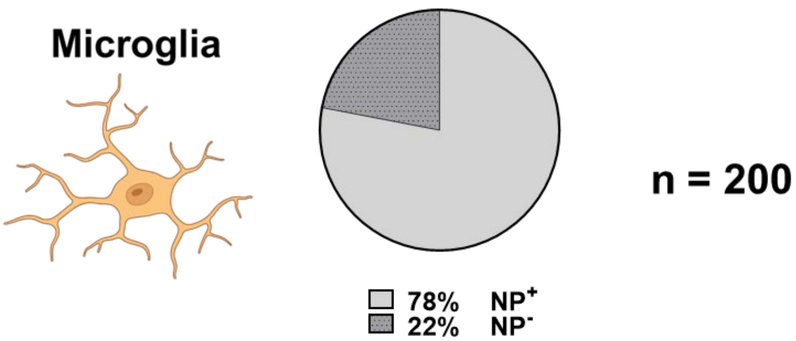


**Figure S34.** Manual counting of $Au_3@PEG_{1k}$ NP-containing microglia in multiple brain cryosections. By counting 200 microglia in both cortex and striatum of $Au_3@PEG_{1k}$ NP-treated R6/2 mice sacrificed at the point of efficacy evaluation (after five weekly injections) based on the schematic shown in **Figure 2A**, we found that 78% of the microglia contained gold reflectance signals. This result indicates the presence of gold NPs in the brain parenchyma.

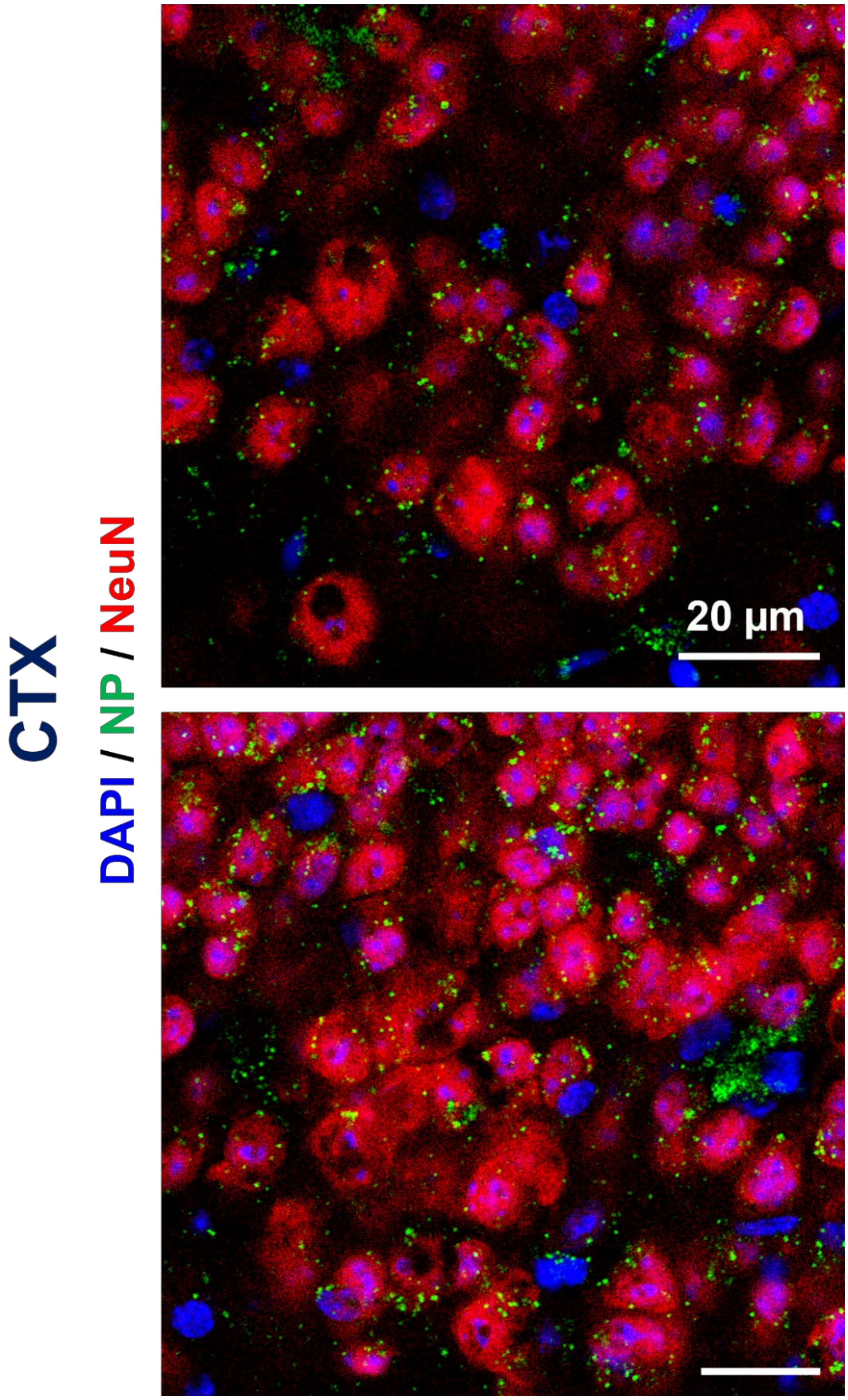


**Figure S35.** Additional enlarged confocal reflectance images of the cryosections of the cortex (CTX) of $Au_3@PEG_{1k}$ NP-treated R6/2 mice sacrificed at the point of efficacy evaluation (after five weekly injections) based on the schematic shown in **Figure 2A**. Red: neurons (NeuN); Blue: nucleus (DAPI); Green: gold NPs (green). These high-magnification images, similar to that in **Figure 2C** but from different brain sections, depict neurons that contribute to the overall manual counting of the 500 $NeuN^+$ neurons.

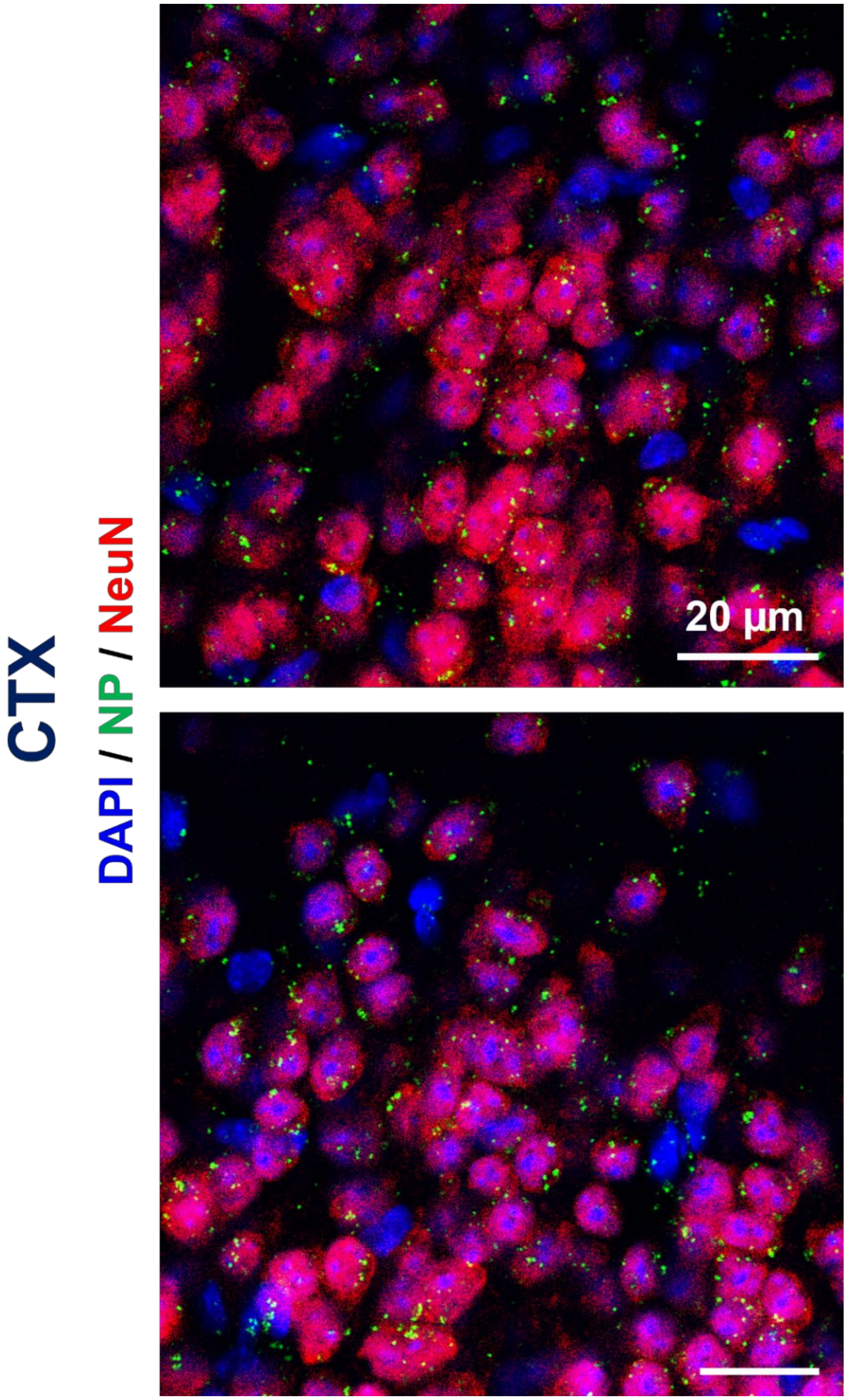


**Figure S36.** Additional enlarged confocal reflectance images of the cryosections of the cortex (CTX) of $Au_3$@$PEG_{1k}$ NP-treated R6/2 mice sacrificed at the point of efficacy evaluation (after five weekly injections) based on the schematic shown in **Figure 2A**. Red: neurons (NeuN); Blue: nucleus (DAPI); Green: gold NPs (green). These high-magnification images, similar to that in **Figure 2C** but from different brain sections, depict neurons that contribute to the overall manual counting of the 500 $NeuN^+$ neurons.

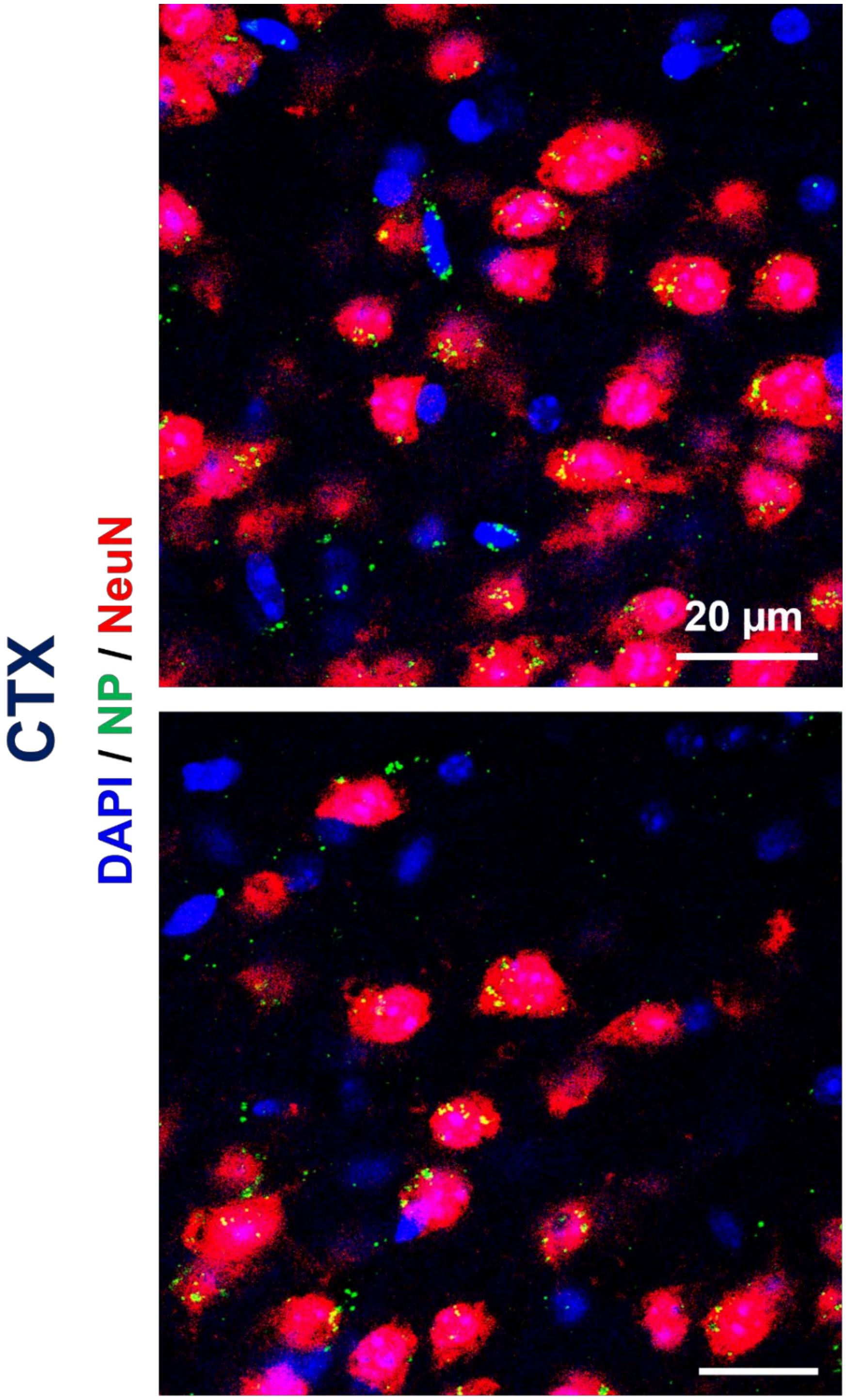


**Figure S37.** Additional enlarged confocal reflectance images of the cryosections of the cortex (CTX) of $Au_3@PEG_{1k}$ NP-treated R6/2 mice sacrificed at the point of efficacy evaluation (after five weekly injections) based on the schematic shown in **Figure 2A**. Red: neurons (NeuN); Blue: nucleus (DAPI); Green: gold NPs (green). These high-magnification images, similar to that in **Figure 2C** but from different brain sections, depict neurons that contribute to the overall manual counting of the 500 $NeuN^+$ neurons.

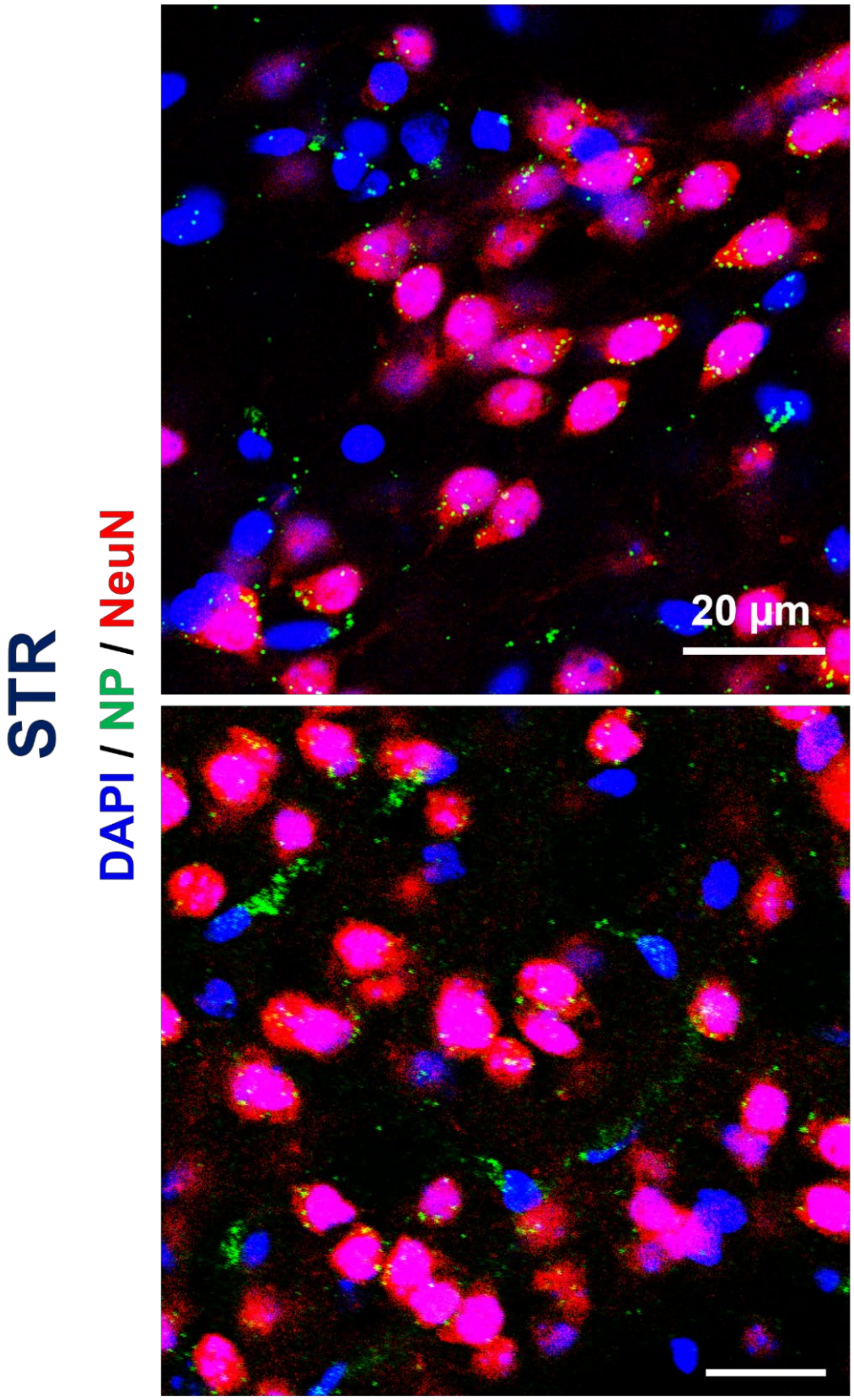


**Figure S38.** Additional enlarged confocal reflectance images of the cryosections of the striatum (STR) of $Au_3@PEG_{1k}$ NP-treated R6/2 mice sacrificed at the point of efficacy evaluation (after five weekly injections) based on the schematic shown in **Figure 2A**. Red: neurons (NeuN); Blue: nucleus (DAPI); Green: gold NPs (green). These high-magnification images, similar to that in **Figure 2C** but from different brain sections, depict neurons that contribute to the overall manual counting of the 500 $NeuN^+$ neurons.

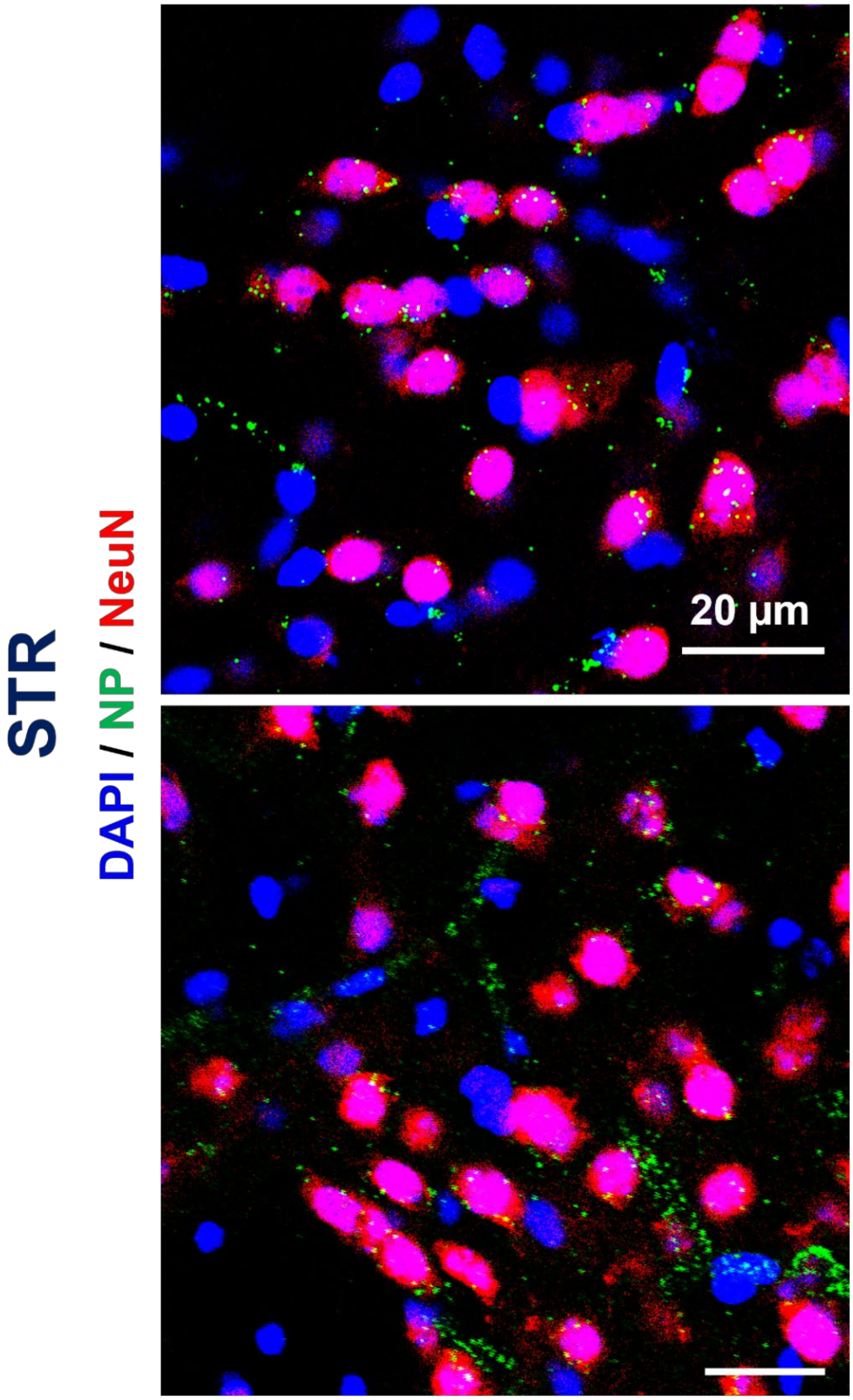


**Figure S39.** Additional enlarged confocal reflectance images of the cryosections of the striatum (STR) of $Au_3$@$PEG_{1k}$ NP-treated R6/2 mice sacrificed at the point of efficacy evaluation (after five weekly injections) based on the schematic shown in **Figure 2A**. Red: neurons (NeuN); Blue: nucleus (DAPI); Green: gold NPs (green). These high-magnification images, similar to that in **Figure 2C** but from different brain sections, depict neurons that contribute to the overall manual counting of the 500 $NeuN^+$ neurons.

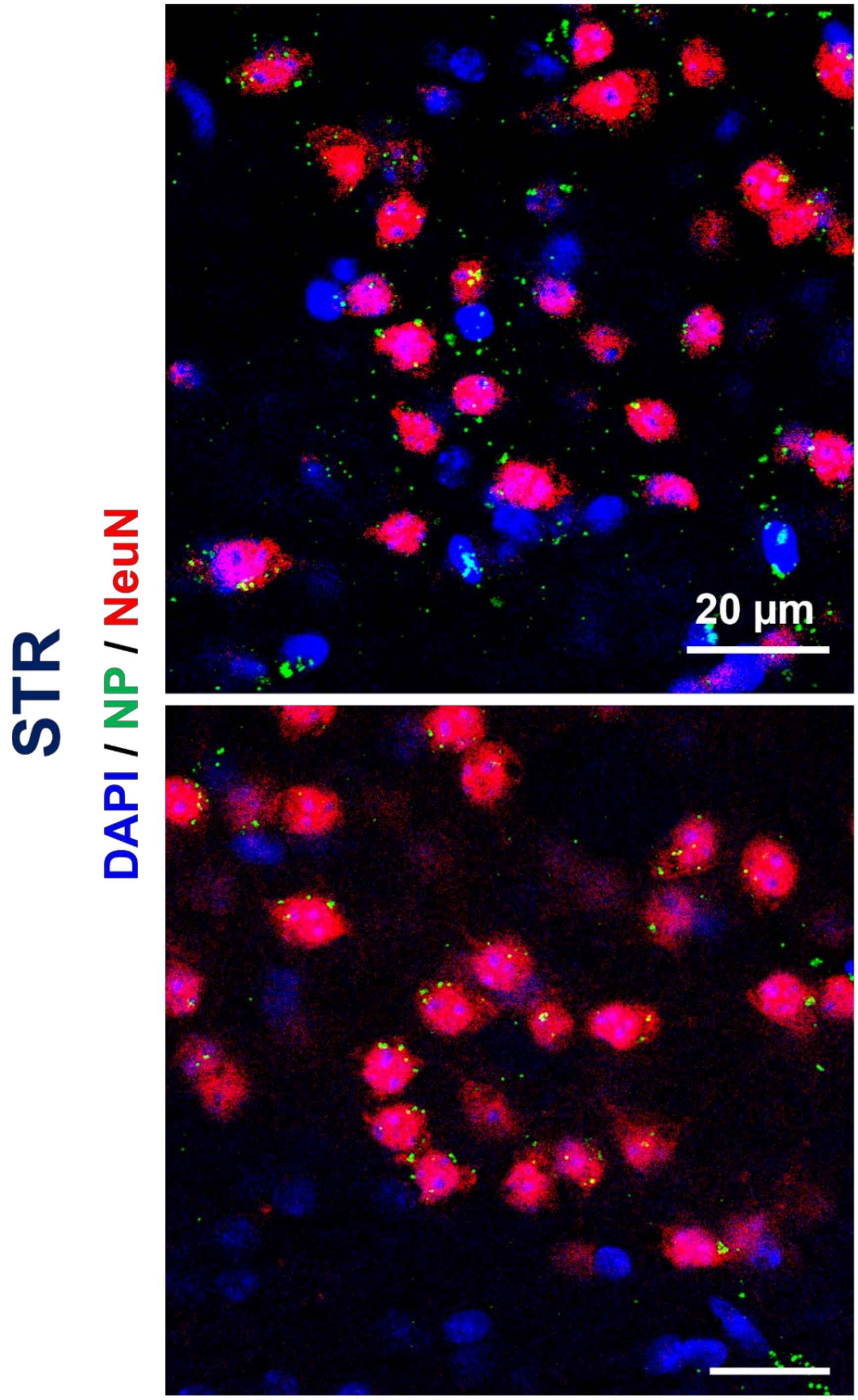


**Figure S40.** Additional enlarged confocal reflectance images of the cryosections of the striatum (STR) of $Au_3$@$PEG_{1k}$ NP-treated R6/2 mice sacrificed at the point of efficacy evaluation (after five weekly injections) based on the schematic shown in **Figure 2A**. Red: neurons (NeuN); Blue: nucleus (DAPI); Green: gold NPs (green). These high-magnification images, similar to that in **Figure 2C** but from different brain sections, depict neurons that contribute to the overall manual counting of the 500 NeuN$^+$ neurons.

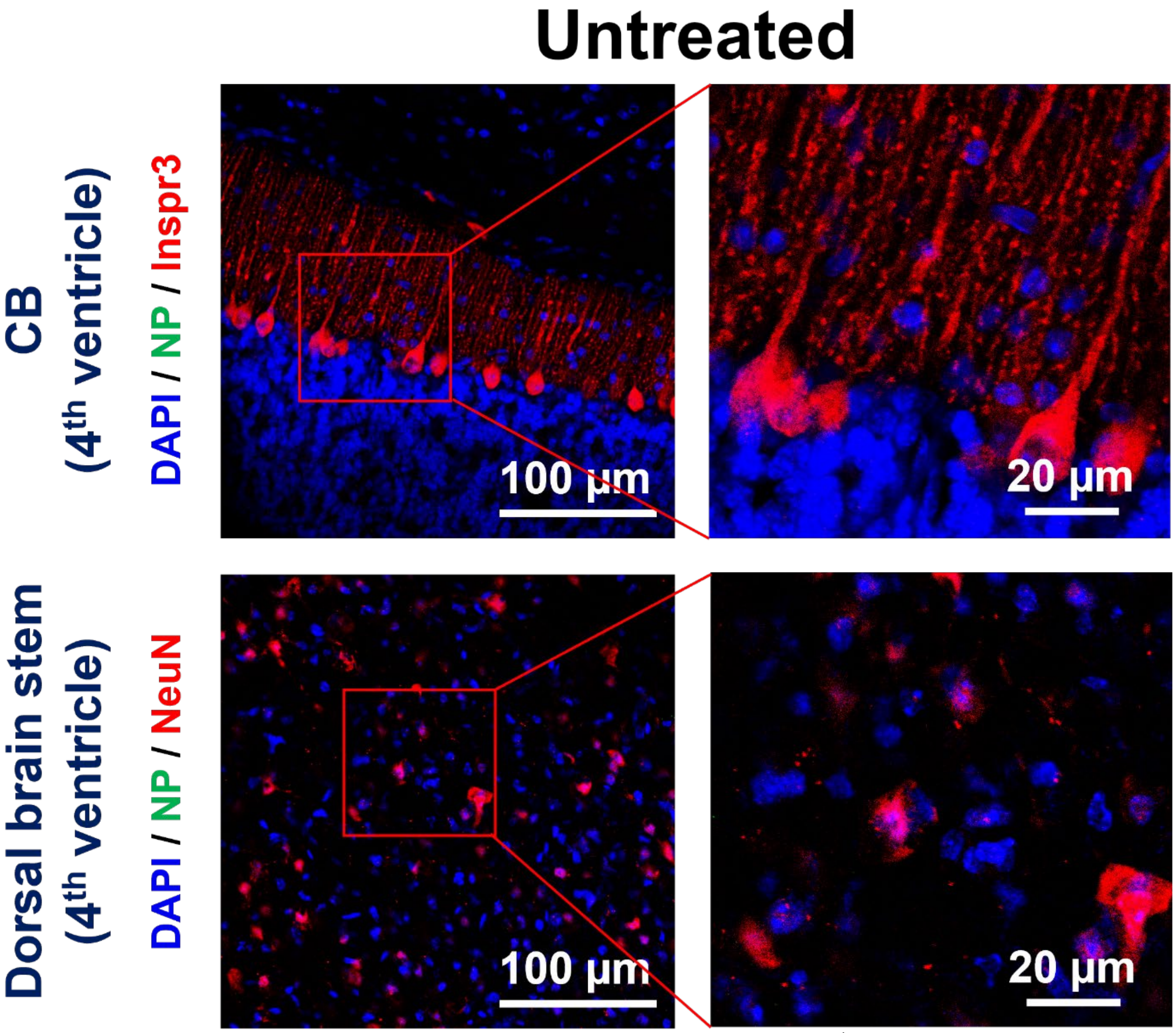


**Figure S41**. Confocal reflectance images of the cryosections of the 4$^{th}$ ventricle [cerebellum (CB) and dorsal brain stem] of untreated R6/2 mice sacrificed at the point of efficacy evaluation (after five weekly injections) based on the schematic shown in **Figure 2A**. The right column features the enlarged images of the boxed area in the left column. Red: Purkinje cell (Inspr3), neuron (NeuN); Blue: nucleus (DAPI). There was no obvious gold reflectance signal (green).

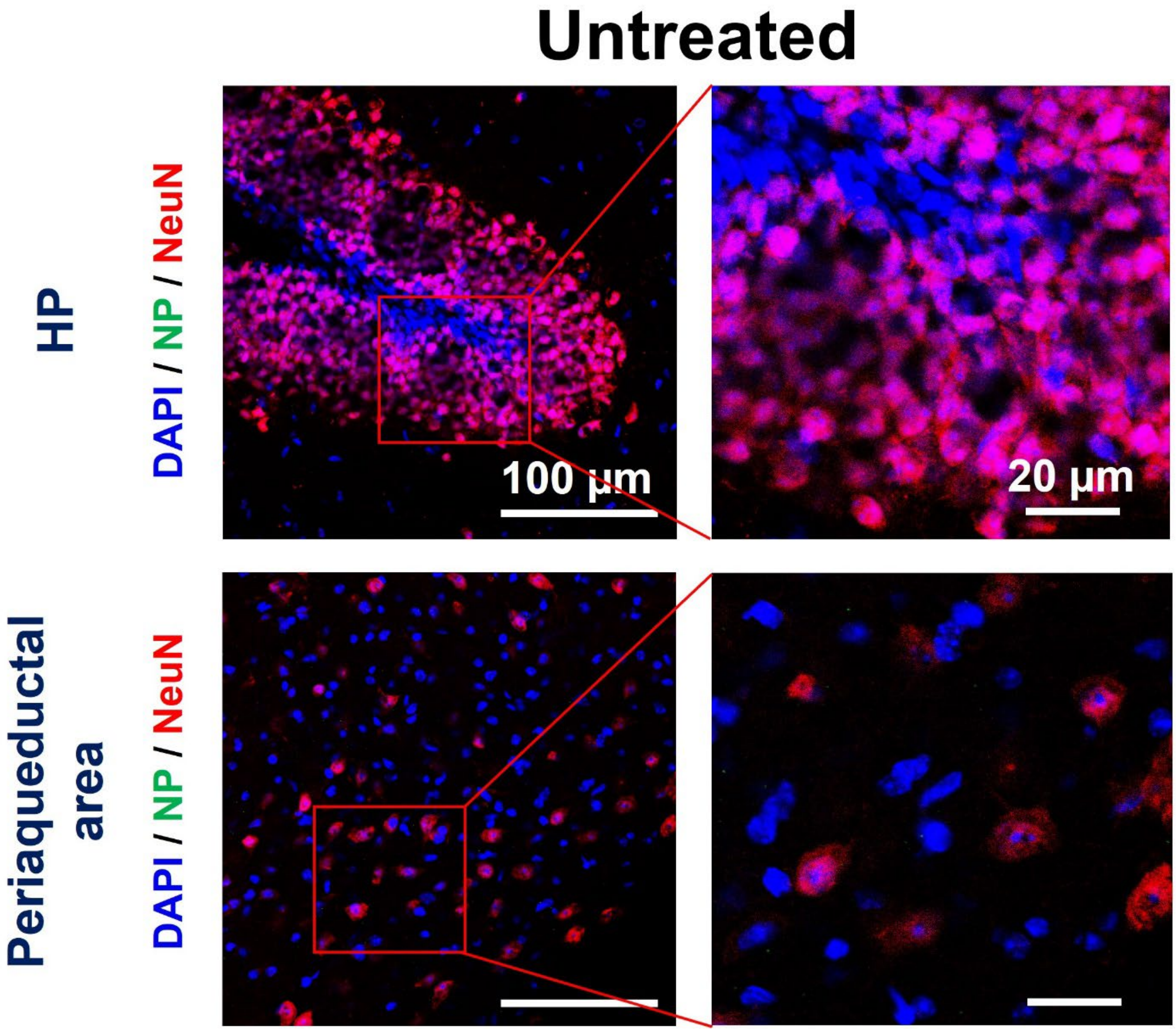


**Figure S42.** Confocal reflectance images of the cryosections of the hippocampus (HP) and periaqueductal area of untreated R6/2 mice sacrificed at the point of efficacy based on the schematic shown in **Figure 2A**. The right column features the enlarged images of the boxed area in the left column. Red: neurons (NeuN); Blue: nucleus (DAPI); Green: gold NPs (green).

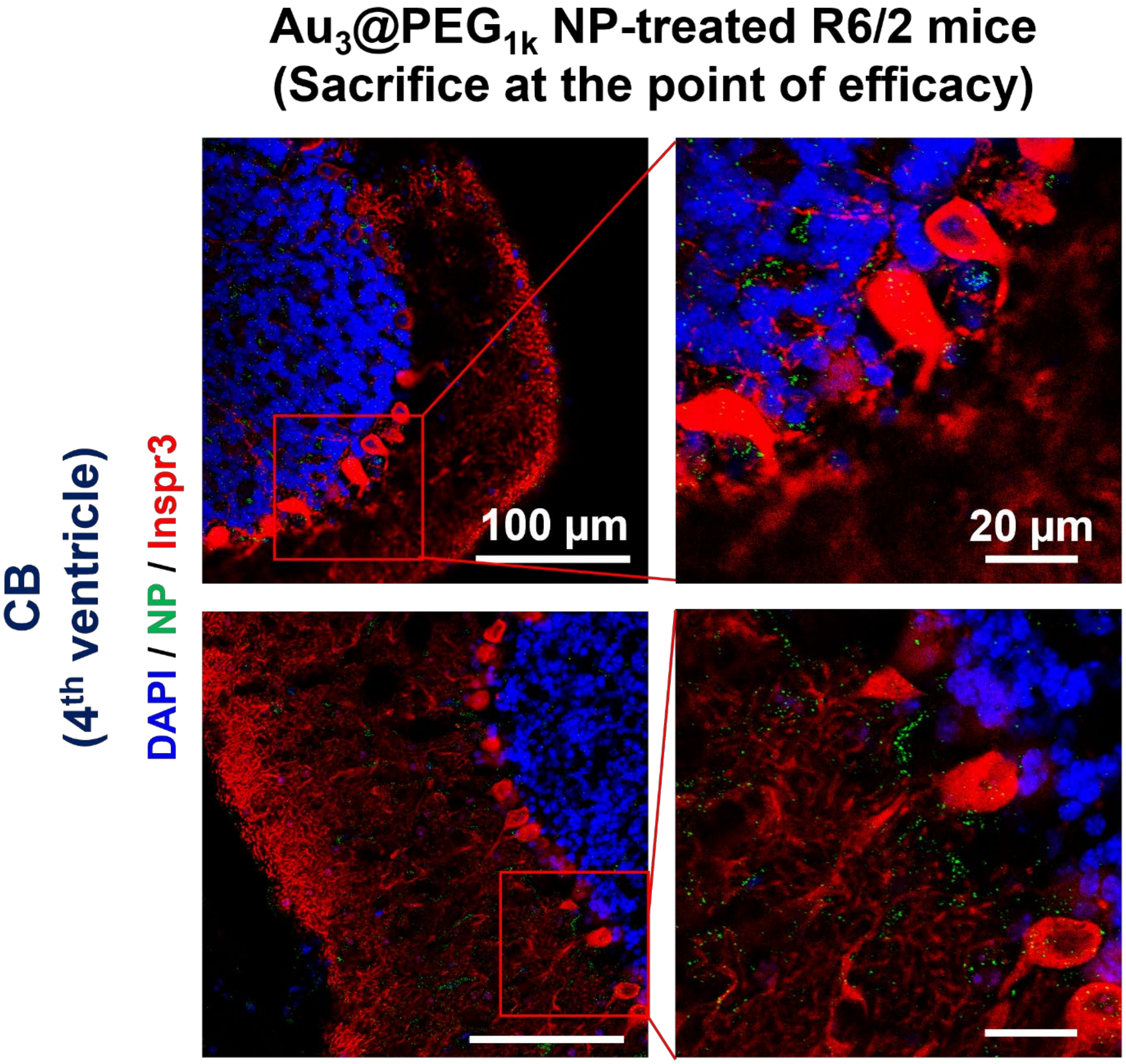


**Figure S43.** Confocal reflectance images of the cryosections of the cerebellum (CB) around the 4th ventricle of $Au_3$@$PEG_{1k}$ NP-treated R6/2 mice sacrificed at the point of efficacy evaluation (after five weekly injections) based on the schematic shown in **Figure 2A**. The right column features the enlarged images of the boxed area in the left column. Red: Purkinje cell (Inspr3), neurons (NeuN); Blue: nucleus (DAPI); Green: gold NPs (green).

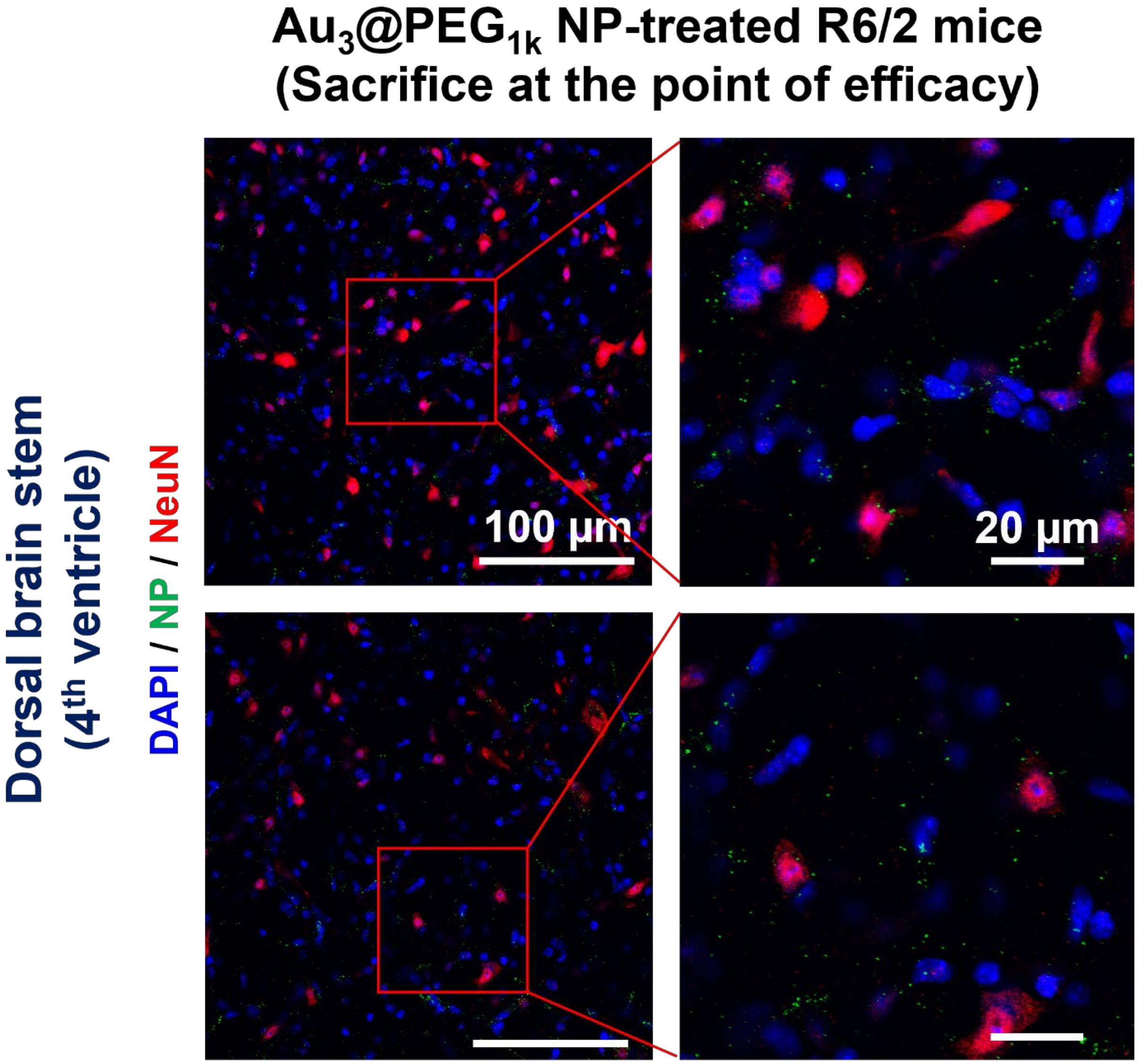


**Figure S44.** Confocal reflectance images of the cryosections of the dorsal brain stem around the 4th ventricle of $Au_3$@$PEG_{1k}$ NP-treated R6/2 mice sacrificed at the point of efficacy evaluation (after five weekly injections) based on the schematic shown in **Figure 2A**. The right column features the enlarged images of the boxed area in the left column. Red: neurons (NeuN); Blue: nucleus (DAPI); Green: gold NPs (green).

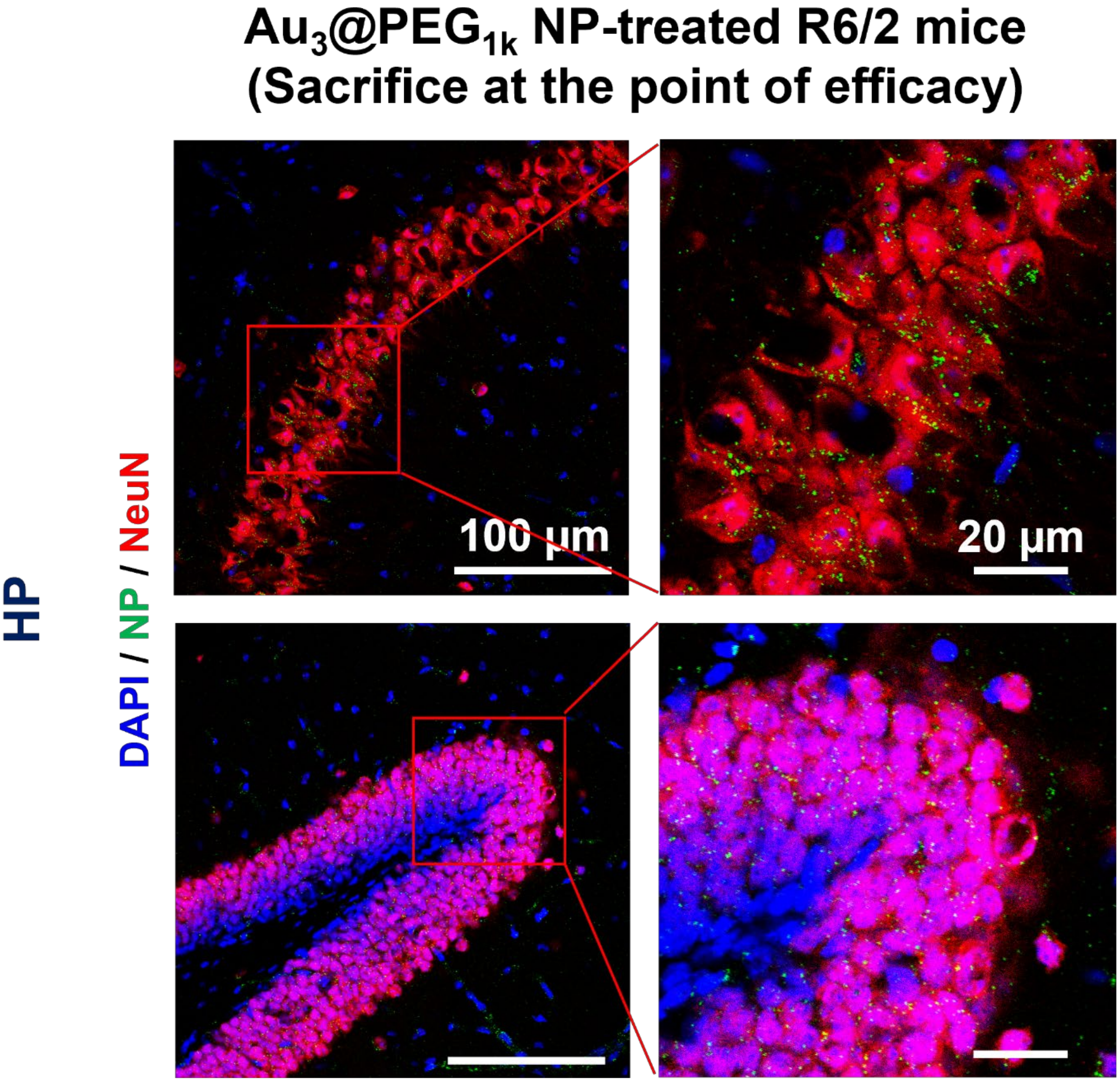


**Figure S45.** Confocal reflectance images of the cryosections of the hippocampus (HP) of $Au_3$@$PEG_{1k}$ NP-treated R6/2 mice sacrificed at the point of efficacy evaluation (after five weekly injections) based on the schematic shown in **Figure 2A**. The right column features the enlarged images of the boxed area in the left column. Red: neurons (NeuN); Blue: nucleus (DAPI); Green: gold NPs (green).

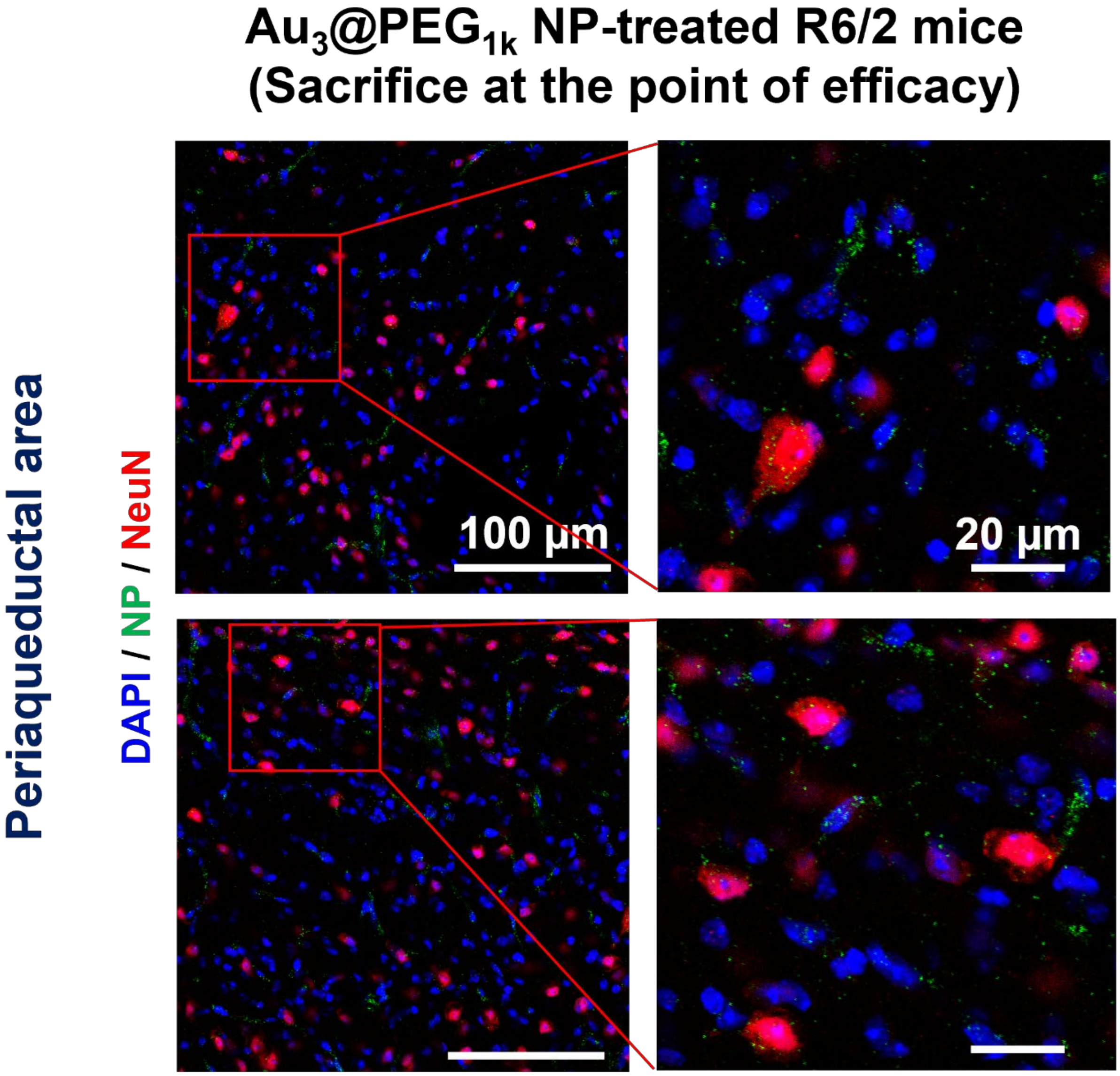


**Figure S46.** Confocal reflectance images of the cryosections of the periaqueductal area of $Au_3@PEG_{1k}$ NP-treated R6/2 mice sacrificed at the point of efficacy evaluation (after five weekly injections) based on the schematic shown in **Figure 2A**. The right column features the enlarged images of the boxed area in the left column. Red: neurons (NeuN); Blue: nucleus (DAPI); Green: gold NPs (green).

# Appendix VII: Efficacy

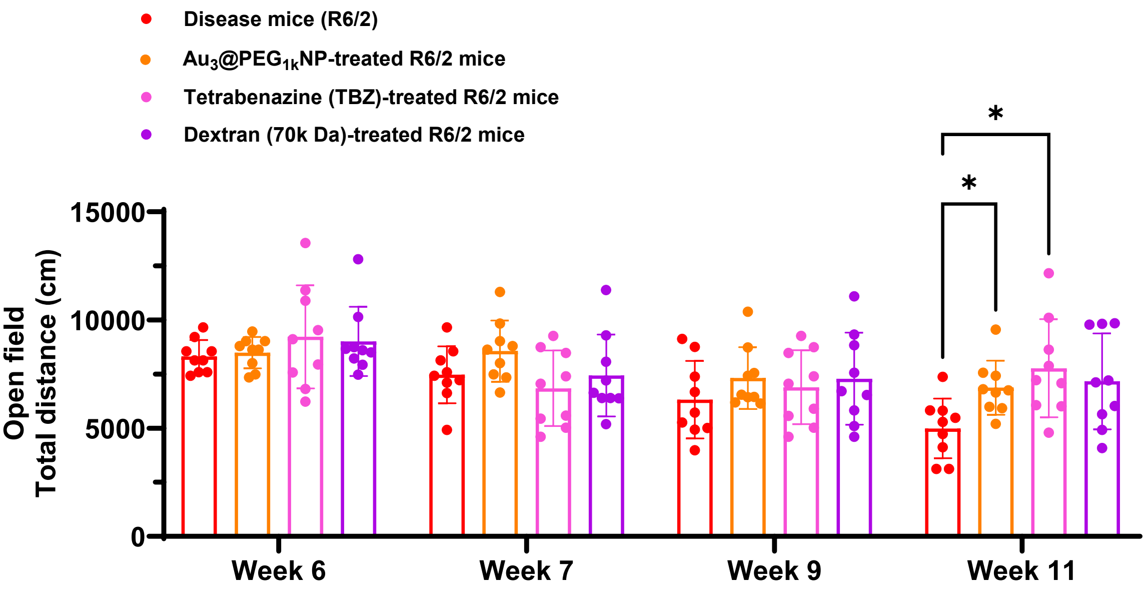


**Figure S47.** Open field test of untreated (red), $Au_3@PEG_{1k}$ NP-treated (orange), tetrabenazine (TBZ)-treated (pink), 70 kDa dextran-treated (**Figure S28**; purple) R6/2 mice as a function of age, based on the treatment plan in **Figure 2A**. At the age of Week 11, both $Au_3@PEG_{1k}$ NPs and TBZ improved the total distance travelled in 30 min relative to untreated control, while 70 kDa dextran kDa)-treated R6/2 mice did not for all ages tested. Data are from n = 9, across five experiments. Statistical significance was evaluated using Two-Way ANOVA with Tukey's post hoc test for multiple comparisons. *$P < 0.05$. All bars and error bars represent mean ± SD. There was little difference in efficacy between gold NPs and TBZ.

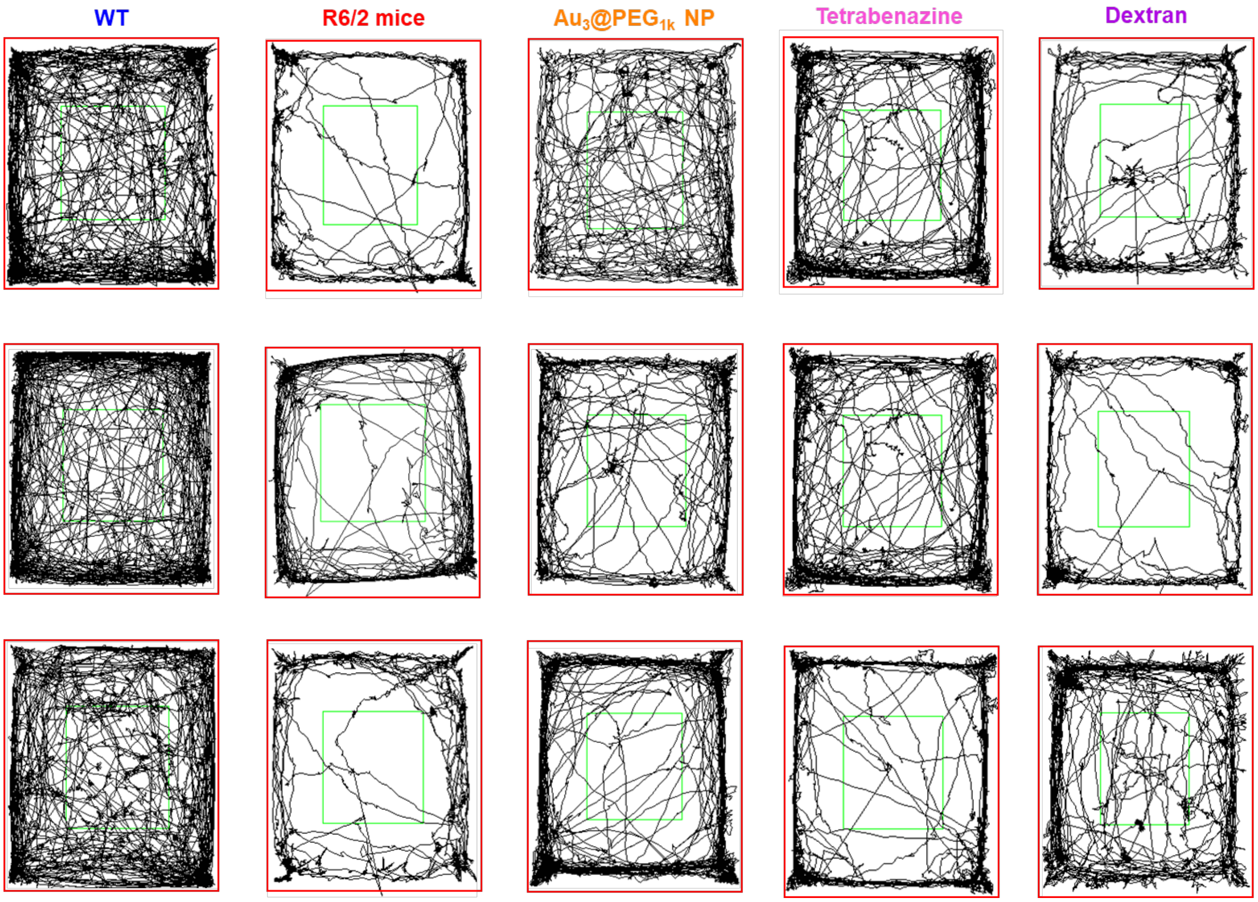


**Figure S48.** Trajectories of untreated (red), $Au_3$@$PEG_{1k}$ NP-treated (orange), tetrabenazine (TBZ)-treated (pink), 70 kDa dextran-treated (purple) R6/2 mice or untreated healthy littermates (WT) after completing an open field test for 30 min based on the treatment plan in **Figure 2A**. Within the same group (column), each row shows a representative trajectory of a different animal. In each panel, the red border indicates the boundary of the open field, and the smaller green box indicates the central region of the open field.

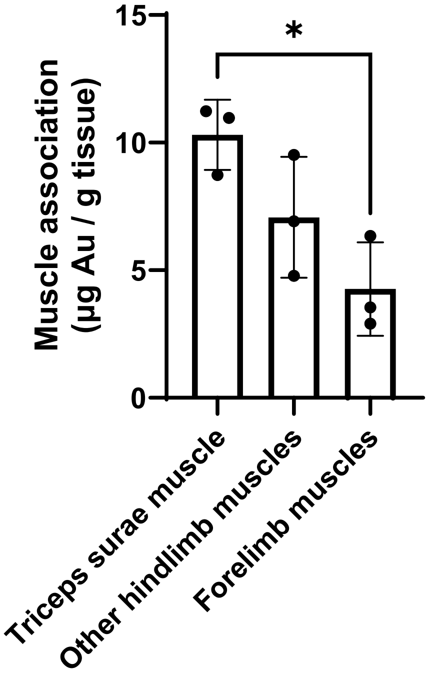


**Figure S49.** $Au_3@PEG_{1k}$ NP in skeletal muscles 24 h post i.v. injection in Week 10 R6/2 HD mice. Gold contents in muscles were detected using ICP-MS and expressed in terms of gold mass per tissue mass. Data are from n = 3, across two experiments. Statistical significance was evaluated using One-Way ANOVA with Tukey's post hoc test for multiple comparisons. *$P < 0.05$. All bars and error bars represent mean ± SD. Tricep surae muscle (part of the hindlimb) was used for ex vivo functional tests of the muscle and neuromuscular junction due to its highest gold contents per g tissue of all muscles tested.

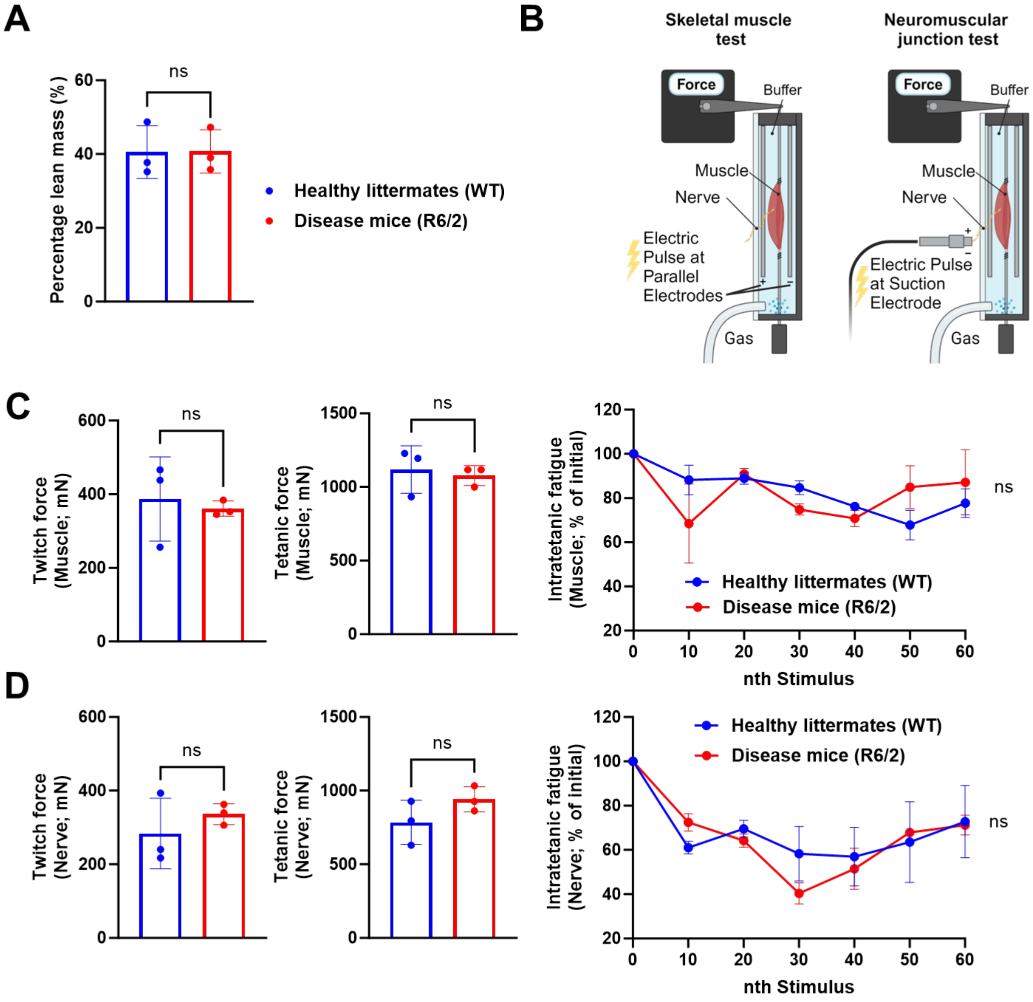


**Figure S50.** Ex vivo functional test of the muscle and neuromuscular junction of Week 11 R6/2 HD mice and age-matched healthy littermates. (A) Body compositional analysis by dual-energy X-ray absorptiometry (DXA) scans revealed no difference in lean mass percentage between untreated healthy littermates (WT, blue) and untreated R6/2 mice (red). (B) Schematic illustration of an ex vivo muscle functional test system. The triceps surae-sciatic nerve complex was immersed into a carboxygenated synthetic intestinal fluid (SIF) buffer bath. An electric pulse was applied by two parallel electrodes for stimulating muscle (left) or a suction electrode for stimulating nerve (right). A single pulse applied to muscle fiber results in a single contraction or "twitch", while continuous or sustained contraction is termed "tetanic" contraction[43]. The decline in maximal force production in response to contractile stimuli is defined as muscle fatigue, induced by repeated tetanic stimuli to assess the skeletal muscle fatigability. (C) Ex vivo triceps surae muscle twitch force, tetanic force and intratetanic fatigue of untreated Week 11 R6/2 HD mice (red) and untreated age-matched healthy littermates (WT, blue) were not different, indicating limited muscle degeneration in HD mice. (D) Ex vivo triceps surae-sciatic nerve twitch force, tetanic force and intratetanic fatigue of untreated healthy littermates (WT, blue) and untreated disease mice (R6/2, red) at the age of Week 11 were not different, indicating limited degeneration of neuromuscular junction in HD mice. Data are from n = 3, across one experiment. Statistical significance was evaluated using unpaired Student's t-test. ns = no significant difference ($P > 0.05$). All bars and error bars represent mean ± SD.

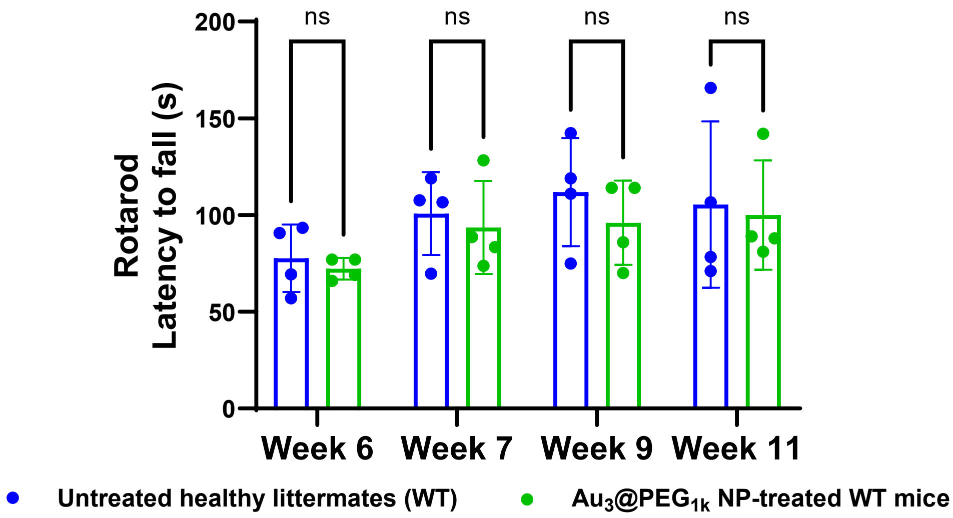


**Figure S51.** Rotarod test of untreated (blue) and $Au_3$@PEG$_{1k}$ NP-treated (green) healthy littermates (WT) as a function of age, based on the treatment plan in **Figure 2A**. Data are from n = 4, across one experiment. Statistical significance was evaluated using Two-Way ANOVA with Šidák post hoc test for multiple comparisons. ns = no significant difference ($P > 0.05$). All bars and error bars represent mean ± SD. NP treatment did not change the behavior of WT mice.

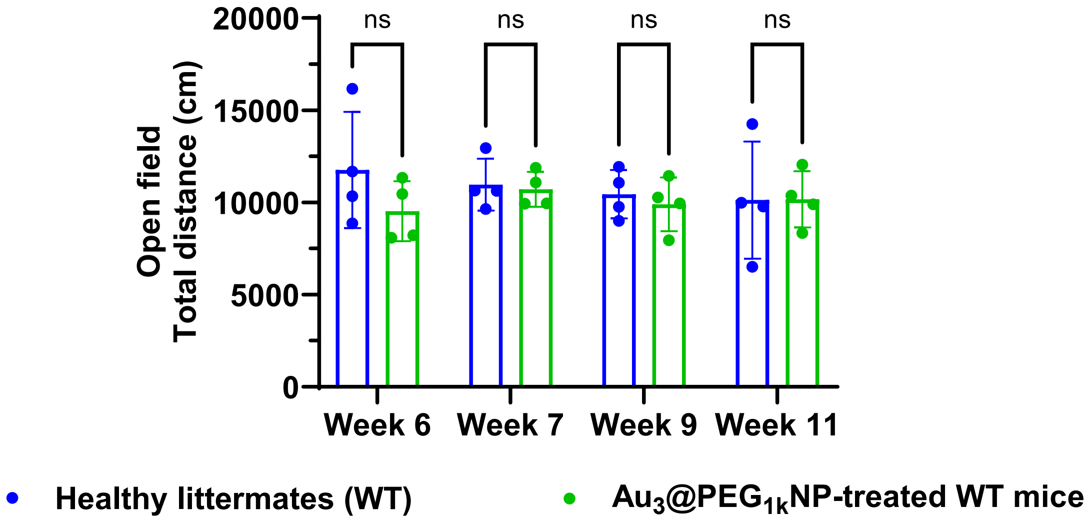


**Figure S52.** Open field test of untreated (blue) and $Au_3$@PEG$_{1k}$ NP-treated (green) healthy littermates (WT) as a function of age, based on the treatment plan in **Figure 2A**. Data are from n = 4, across one experiment. Statistical significance was evaluated using Two-Way ANOVA with Šidák post hoc test for multiple comparisons. ns = no significant difference ($P > 0.05$). All bars and error bars represent mean ± SD. NP treatment did not change the behavior of WT mice.

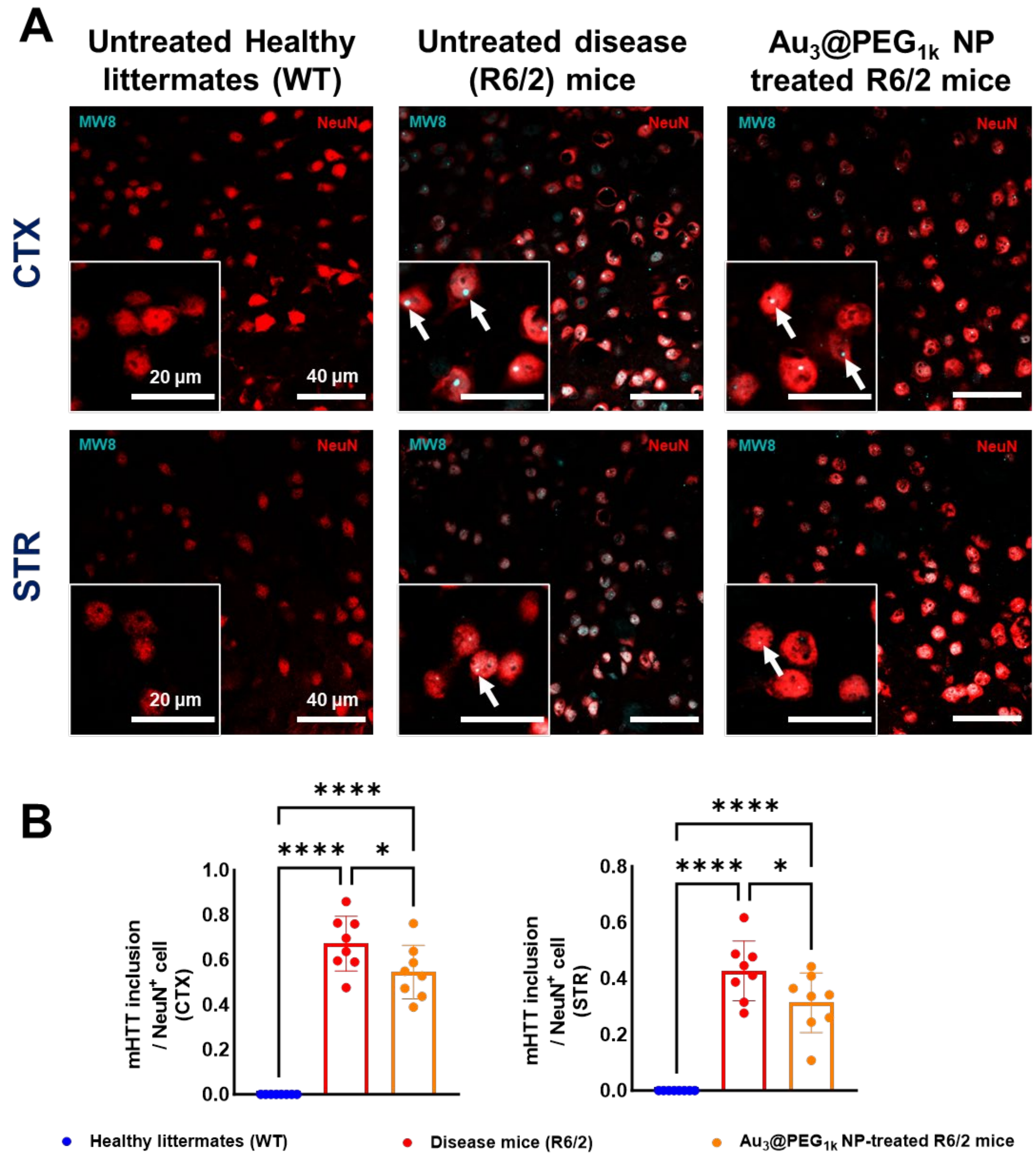


**Figure S53.** $Au_3@PEG_{1k}$ NP-treatment reduced mHTT aggregates in R6/2 mice. (A) Confocal images of MW8-stained mHTT inclusion in the CTX and STR of untreated healthy littermates (WT), untreated R6/2 HD mice and $Au_3@PEG_{1k}$ NP-treated R6/2 HD mice based on the treatment plan in **Figure 2A**. White arrow: mHTT inclusion; Cyan: mHTT (MW8); Red: neurons (NeuN). (B) Quantification of the number of mHTT inclusion in NeuN-positive cells in CTX and STR of untreated WT (blue), untreated R6/2 HD mice (red) and $Au_3@PEG_{1k}$ NP-treated R6/2 HD mice (orange). The statistical significance was evaluated using One-Way ANOVA with Tukey's post hoc test for multiple comparisons. Data are from n = 8, across three experiments. *$P < 0.05$; **$P < 0.01$; ***$P < 0.001$; ****$P < 0.0001$. All bars and error bars represent mean ± SD.

# Appendix VIII: Toxicology

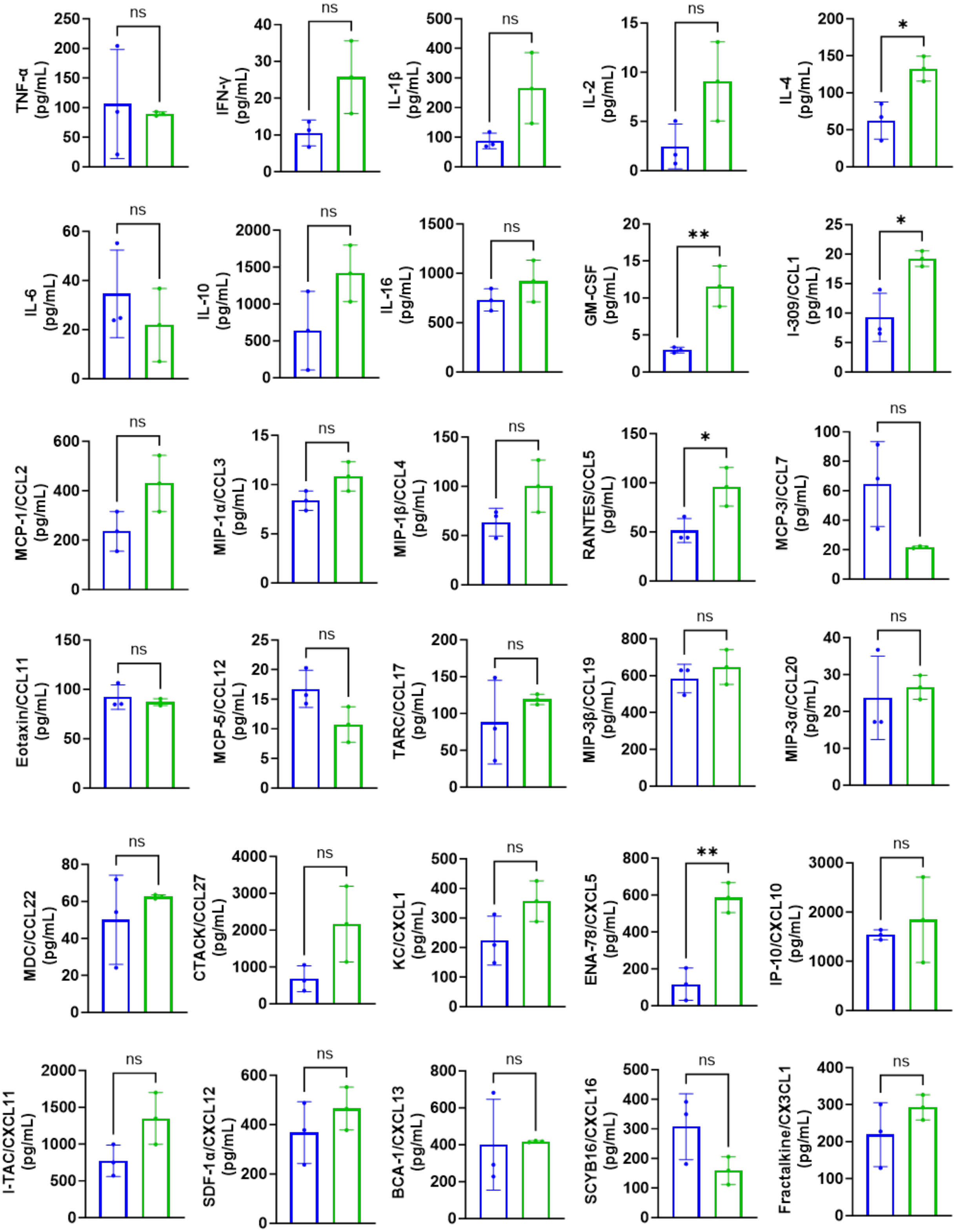

**Figure S54**. Cytokine profiling of plasma of Au3@PEG$_{1k}$ NP-treated WT mice (green) relative to that of untreated WT mice (blue) after five weekly injections (treatment in **Fig. 2A)**. Only 5 out of 25 cytokines or chemokines were significantly upregulated in the Au3@PEG$_{1k}$ NP group, proof of limited immunogenicity. Statistical significances were evaluated using Student's t-test. **$P < 0.01$. All bars and error bars represent mean ± SD.

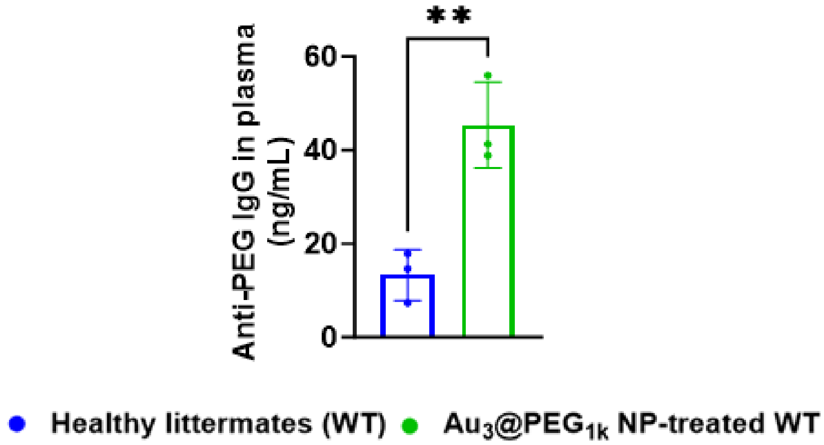


**Figure S55**. ELISA revealed the upregulation of IgG antibodies against PEG in the blood of Au3@PEG$_{1k}$ NP-treated WT (green) relative to that of untreated WT (blue) after five weekly injections (treatment in **Fig. 2A)**. Data are from n = 3, across 1 experiment. Statistical significances were evaluated using Student's t-test. **$P < 0.01$. All bars and error bars represent mean ± SD.

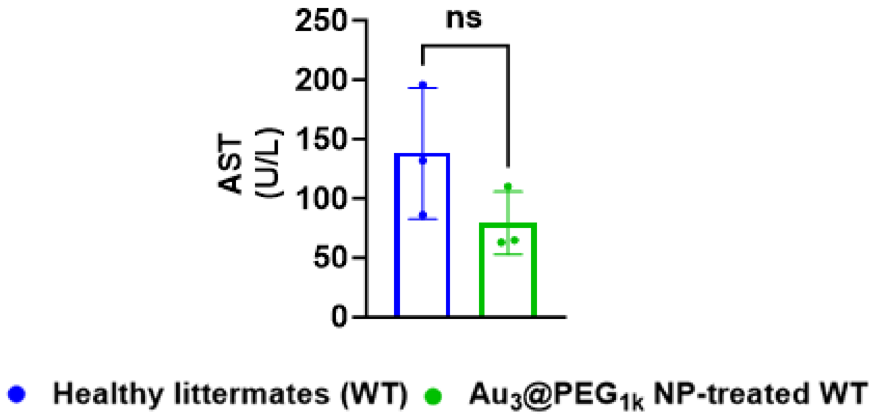


**Figure S56**. Serum level of aspartate aminotransferase (AST) in Au3@PEG$_{1k}$ NP-treated WT mice did not significantly alter relative to untreated WT mice. Data are from n = 3, across 1 experiment. Statistical significances were evaluated using Student's t-test. **$P < 0.01$. ns = no significant difference. All bars and error bars represent mean ± SD.

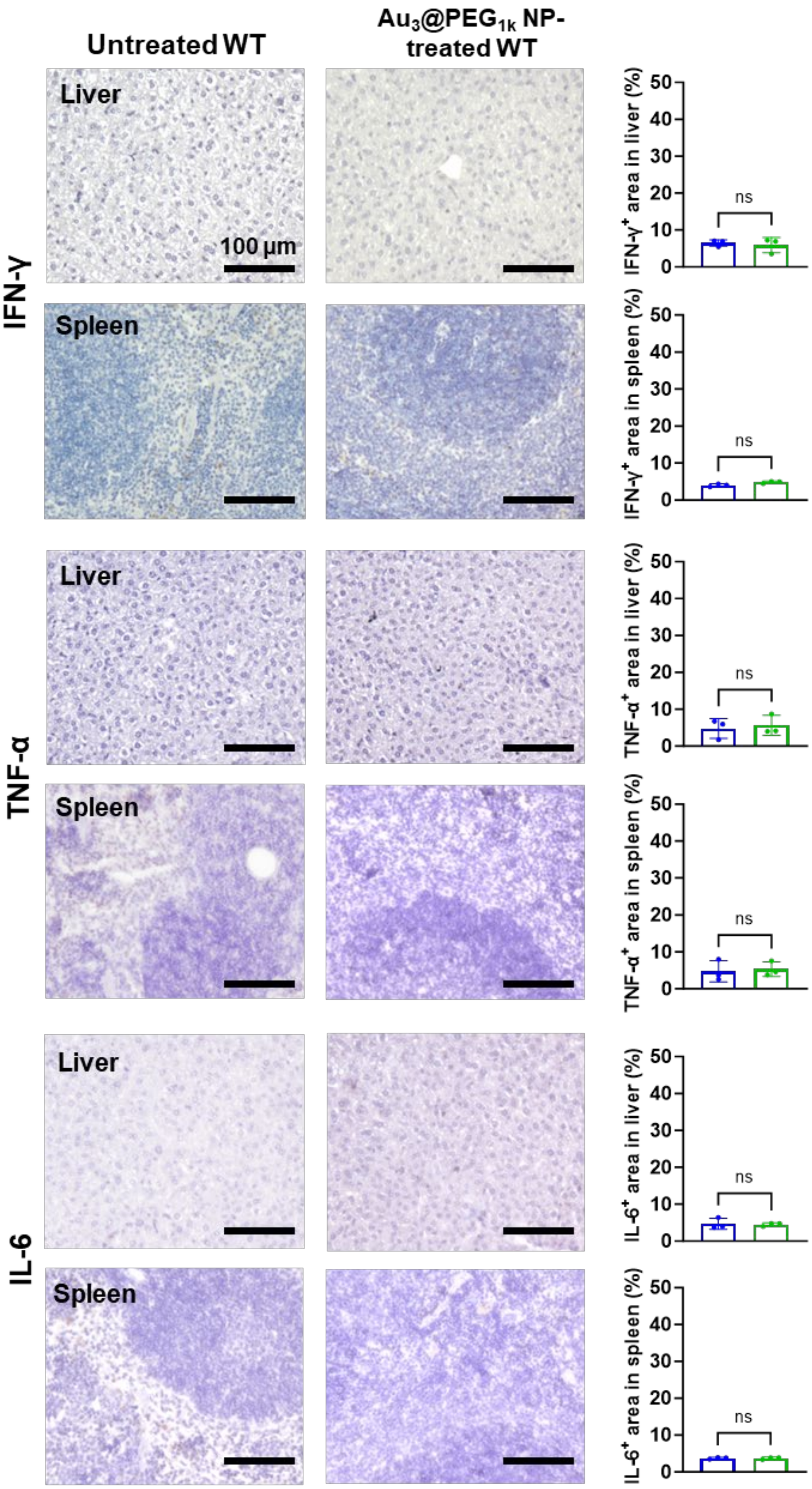


**Figure S57**. IHC of proinflammatory cytokines IFN-γ, TNF-α and IL-6 in the liver and spleen revealed no significant changes following five weekly injections of $Au_3$@$PEG_{1k}$ NP. Data are from n = 3, across 1 experiment. All statistical significances were evaluated using Student's t-test. ns = no significant difference. All bars and error bars represent mean ± SD.

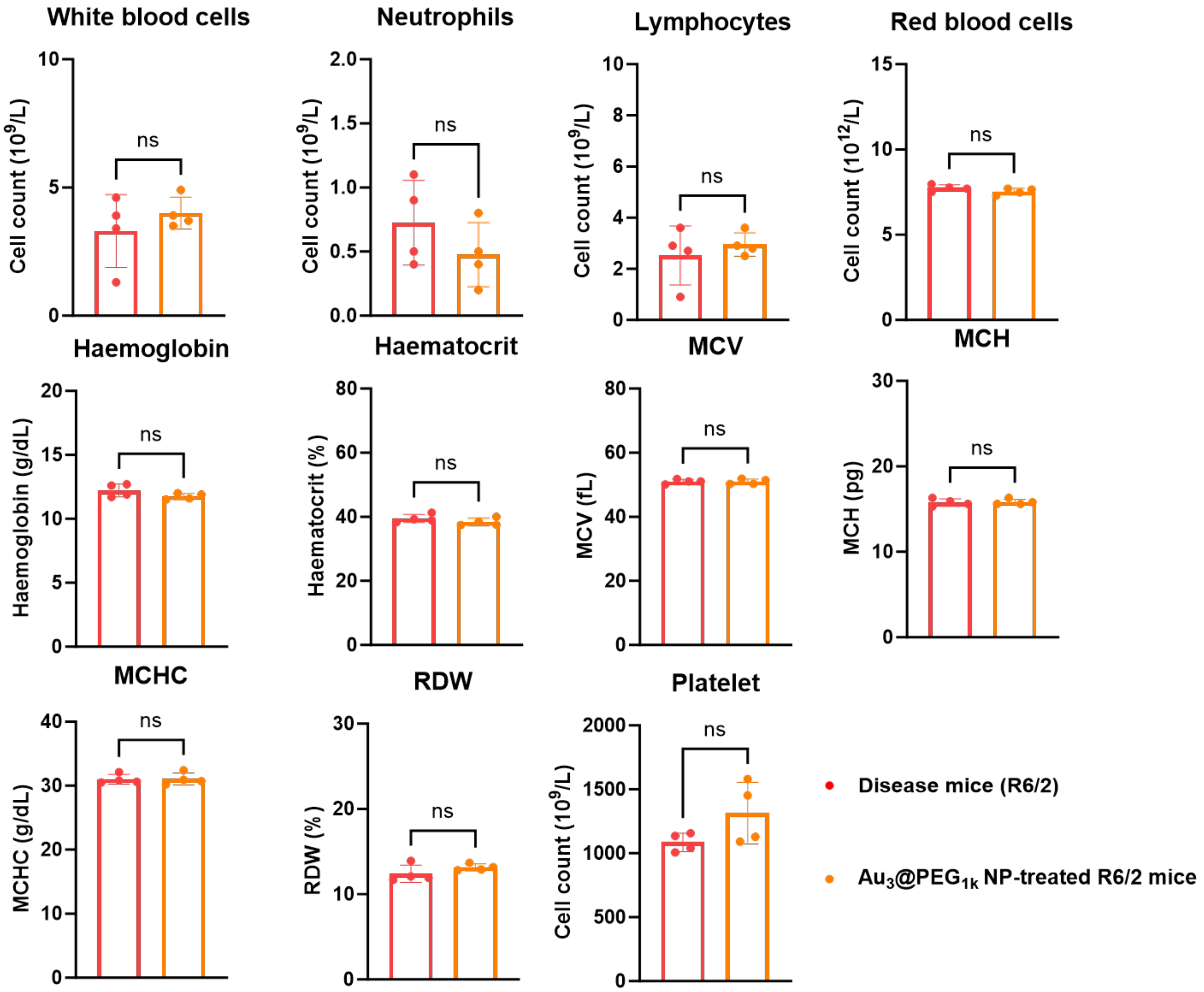


**Figure S58.** Hematology of Week 11 R6/2 HD mice that were untreated (red) or treated by weekly injections of $Au_3$@$PEG_{1k}$ NPs from Weeks 6 to 10 (orange). The markers include white blood cell count, neutrophil count, lymphocyte count, red blood cell count (RBC), hemoglobin, hematocrit, mean corpuscular volume (MCV), mean corpuscular hemoglobin (MCH), mean corpuscular hemoglobin concentration (MCHC), red cell distribution width (RDW), and platelet count. Data are from n = 4, across one experiment. Statistical significance was evaluated using unpaired Student's t-test. ns = no significant difference. All bars and error bars represent mean ± SD.

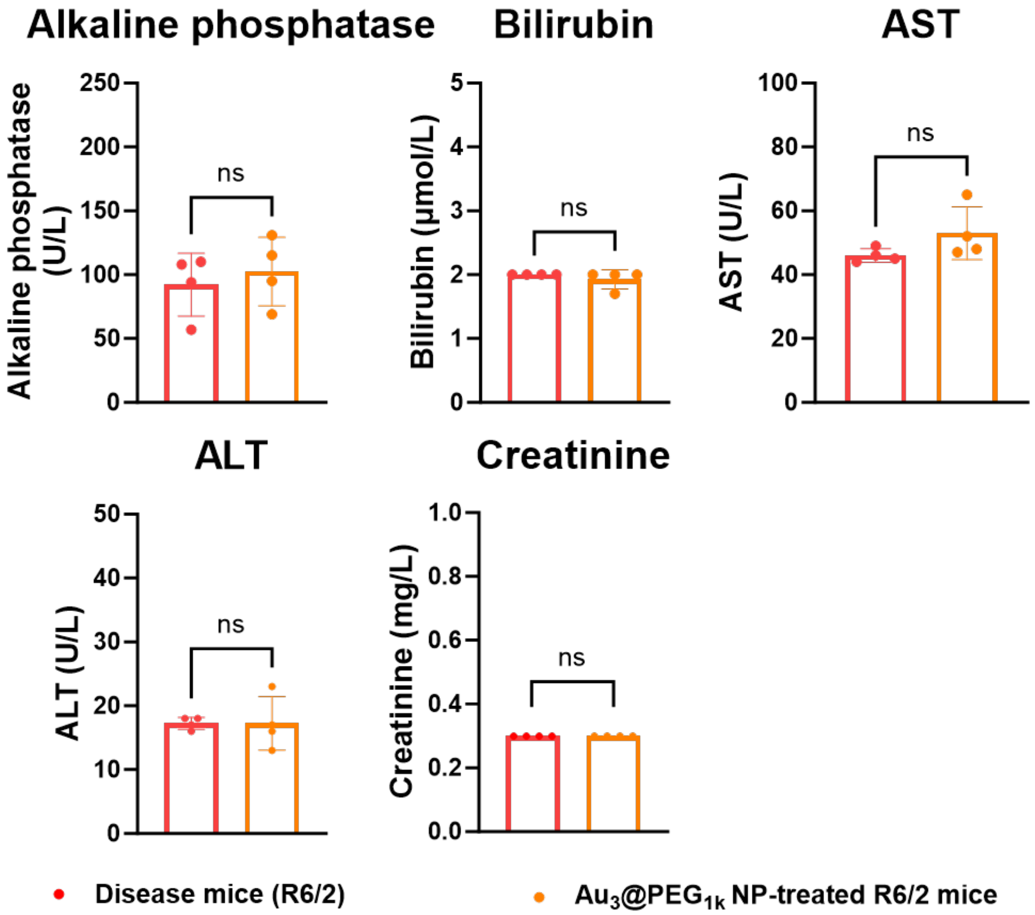


**Figure S59.** Hepatotoxicity and renotoxicity analysis of Week 11 R6/2 HD mice that were untreated (red) or treated by weekly injections of $Au_3$@$PEG_{1k}$ NPs from Weeks 6 to 10 (orange). Serum markers include alkaline phosphatase, total bilirubin, aspartate aminotransferase (AST), alanine transaminase (ALT) and creatinine. Data are from n = 4, across one experiment. Statistical significance was evaluated using unpaired Student's t-test. ns = no significant difference ($P > 0.05$). All bars and error bars represent mean ± SD.

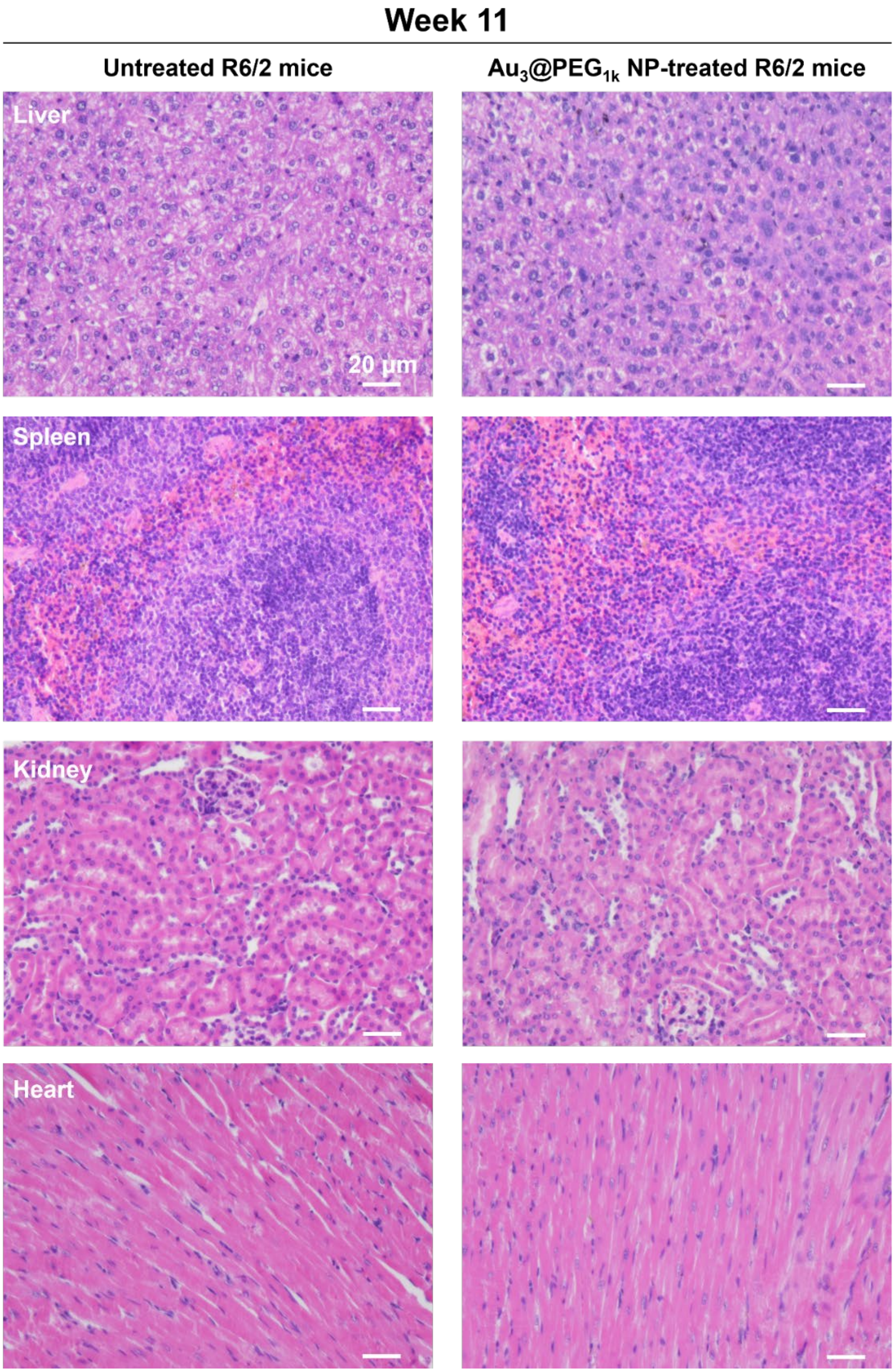


**Figure S60.** Histological images of major organs in Week 11 R6/2 mice following treatment plan in **Figure 2A**. $Au_3$@$PEG_{1k}$ NP did not alter the tissue morphology in liver, spleen, kidney and heart. Representative images from 2 tissue sections from n= 4 mice/group, across one experiment.

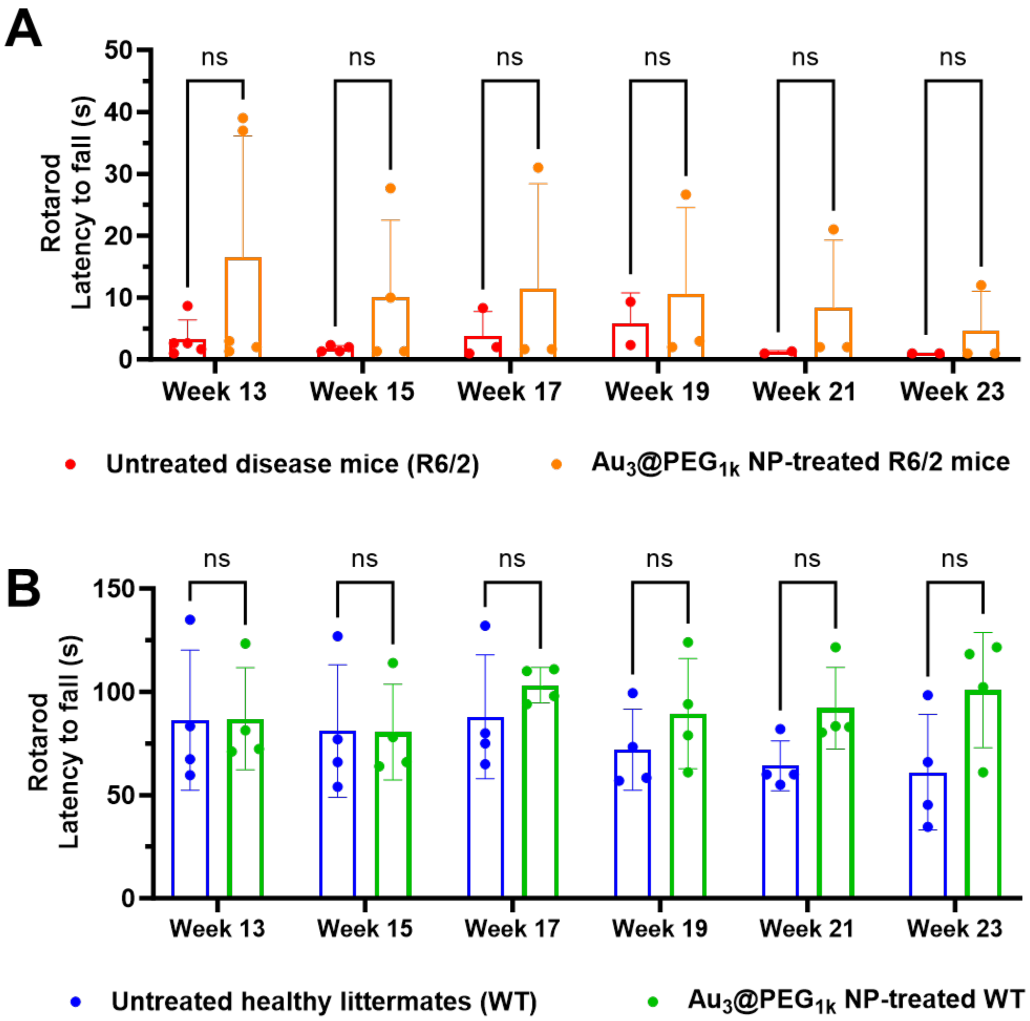


**Figure S61.** End-stage rotarod tests of (A) untreated R6/2 mice (red), $Au_3@PEG_{1k}$ NP-treated R6/2 HD mice (orange), (B) untreated healthy littermates (WT, blue) and $Au_3@PEG_{1k}$ NP-treated WT as a function of age, following weekly NP injections from Weeks 6 to 10 based on the treatment plan in **Figure 3A**. No significant difference was observed between all groups across all ages tested. Data are from n = 4–5, across one experiment. Statistical significance was evaluated using Two-Way ANOVA with Šidák post hoc test for multiple comparisons. ns = no significant difference ($P > 0.05$). All bars and error bars represent mean ± SD.

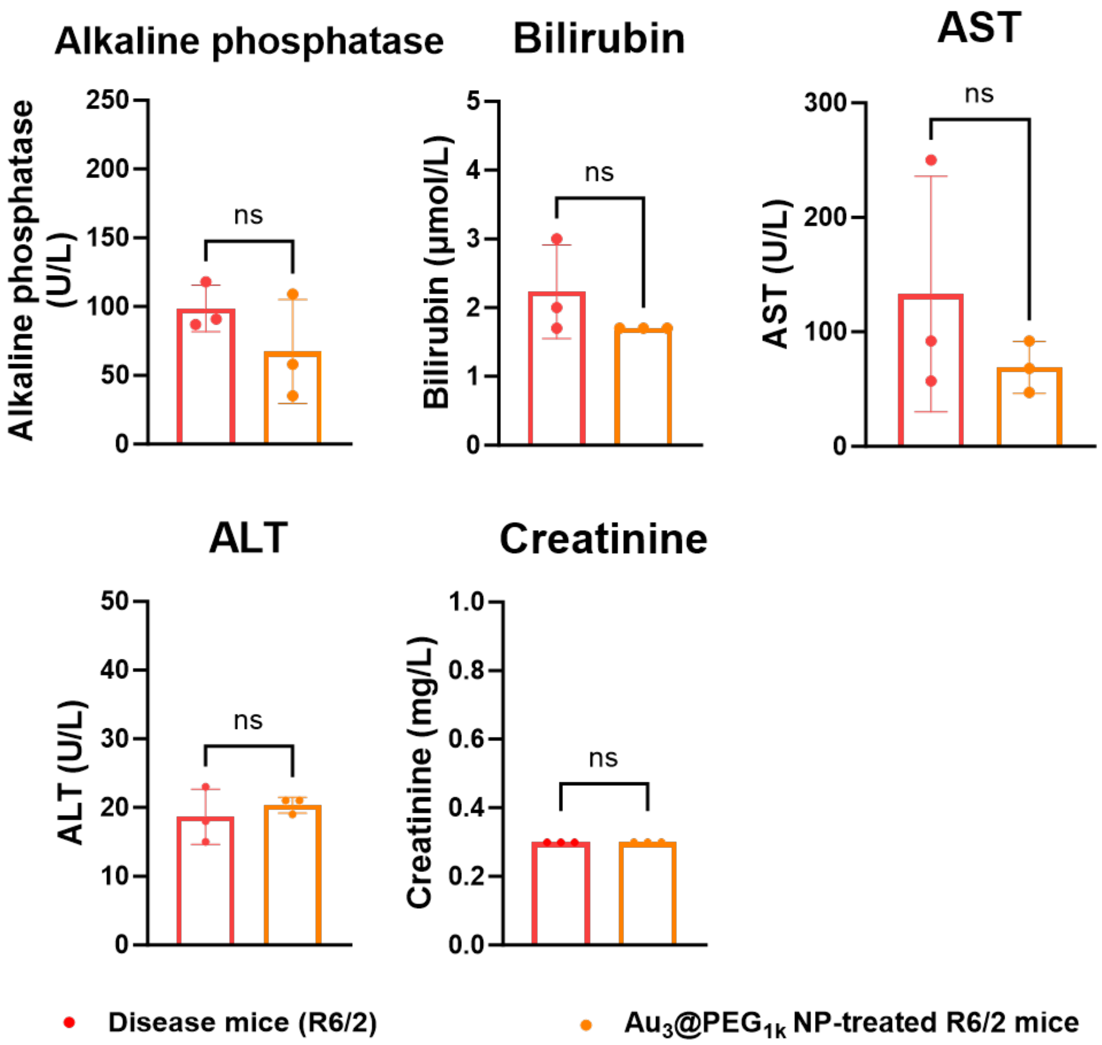


**Figure S62.** Hepatotoxicity and renotoxicity of Week 24 R6/2 HD mice that were untreated (red) or treated by $Au_3$@$PEG_{1k}$ NPs from Weeks 6 to 10 (orange), based on serum markers following the treatment plan in **Figure 3A**. Legend: Aspartate aminotransferase (AST), alanine transaminase (ALT). Data are from n = 4, across one experiment. Statistical significance was evaluated using unpaired Student's t-test. ns = no significant difference ($P > 0.05$). All bars and error bars represent mean ± SD.

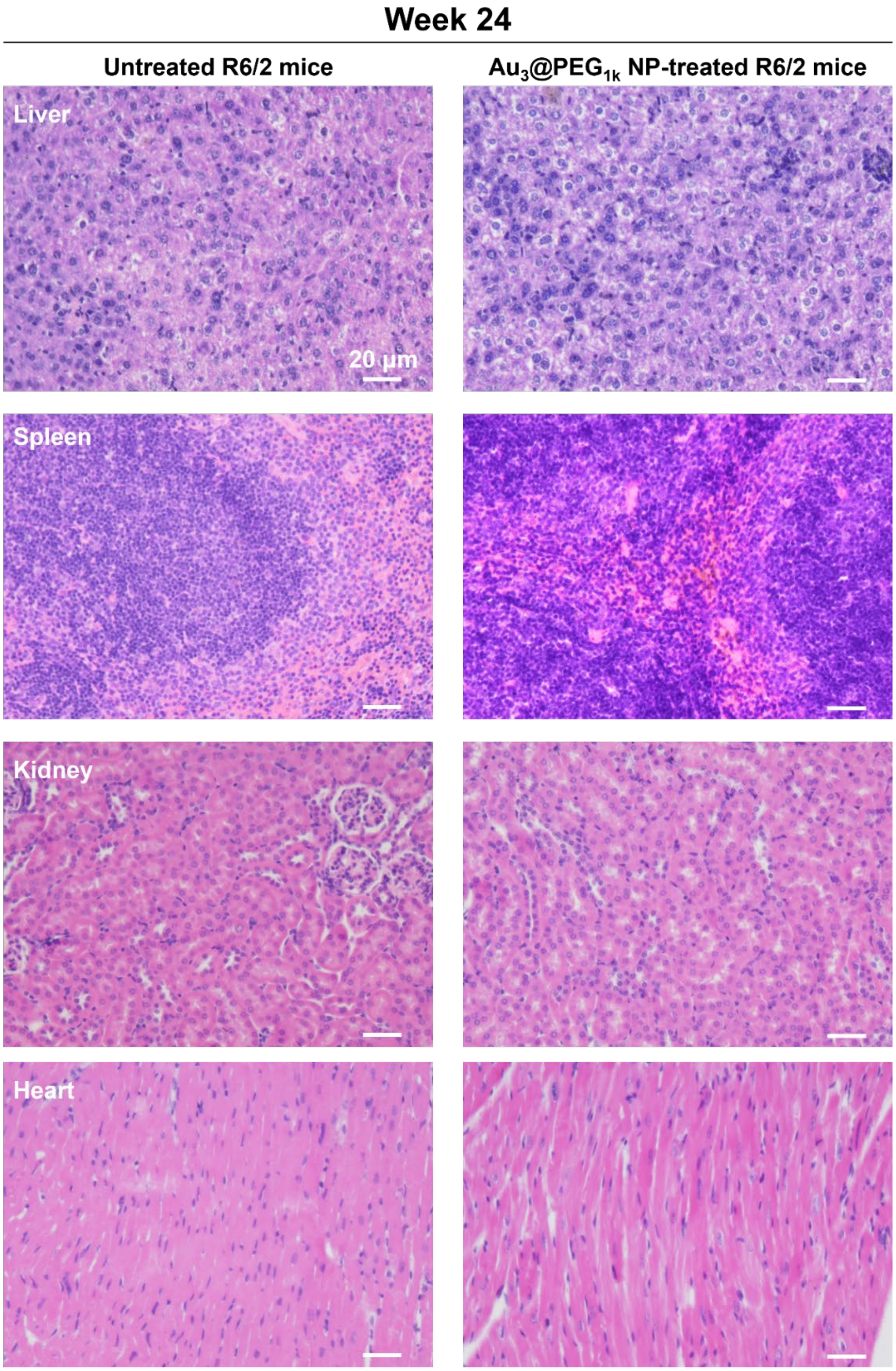


**Figure S63.** Histological images of major organs in Week 24 R6/2 mice following treatment plan in **Figure 3A**. $Au_3@PEG_{1k}$ NP treatment did not alter the tissue morphology in liver, spleen, kidney and heart. Representative images from 2 tissue sections from n = 3 mice/group, across one experiment.

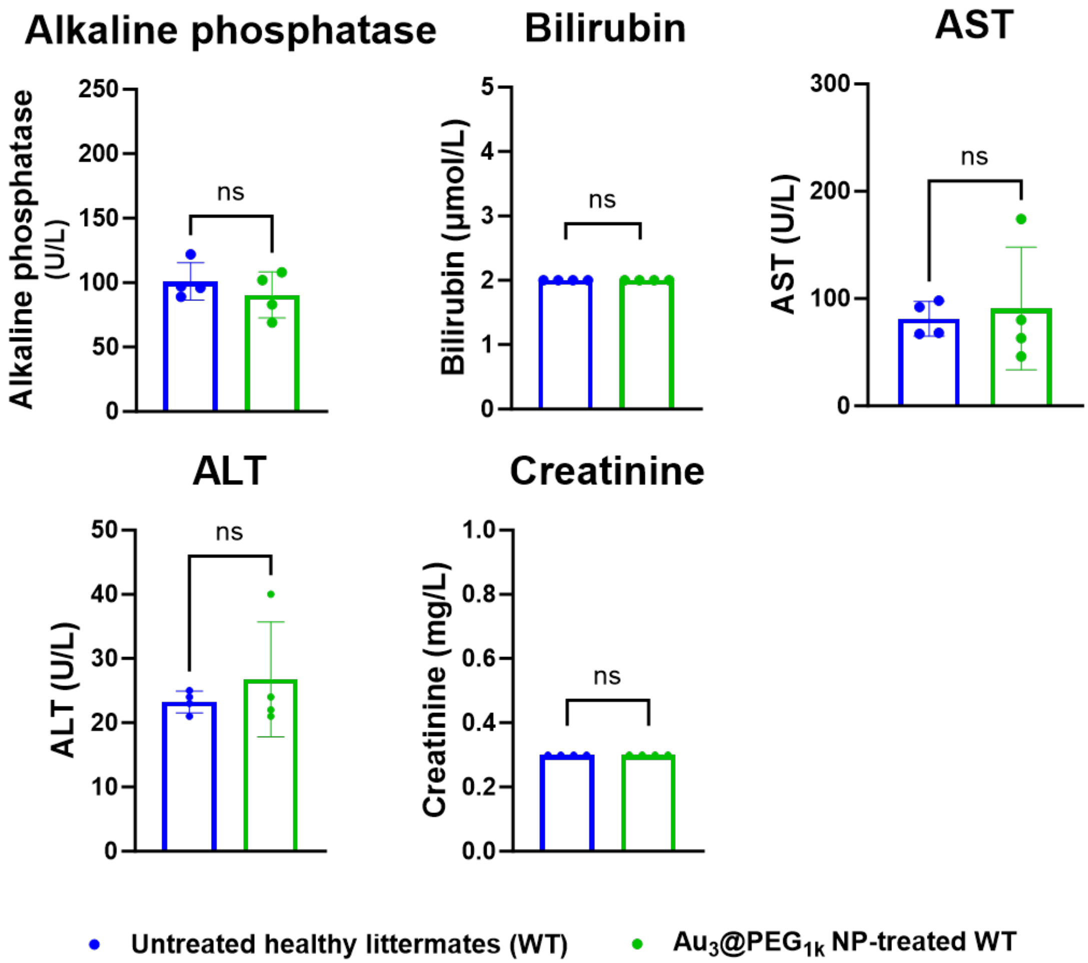


**Figure S64.** Hepatotoxicity and renotoxicity of Week 24 healthy littermates (WT) that were untreated (blue) or treated $Au_3$@$PEG_{1k}$ NPs from Weeks 6 to 10 (green), based on serum markers, following the treatment plan in **Figure 3A**. Legend: Aspartate aminotransferase (AST), alanine transaminase (ALT). Data are from n = 4, across one experiment. Statistical significance was evaluated using unpaired Student's t-test. ns = no significant difference ($P > 0.05$). All bars and error bars represent mean ± SD.

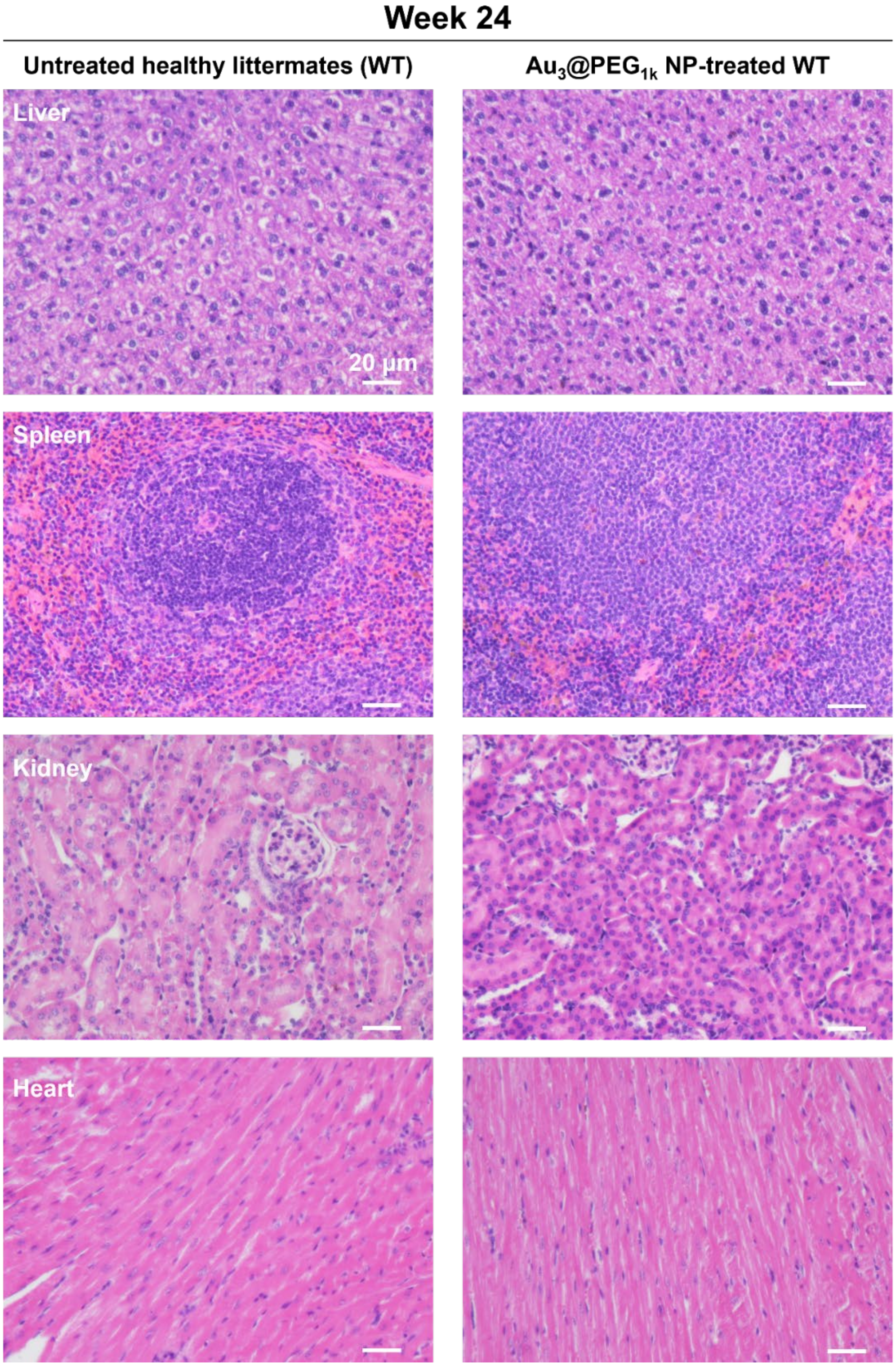


**Figure S65.** Histological images of major organs in Week 24 healthy littermates following treatment plan in **Figure 3A**. $Au_3$@$PEG_{1k}$ NP treatment did not alter the tissue morphology in liver, spleen, kidney and heart. Representative images from 2 tissue sections from n = 4 mice/group, across one experiment.

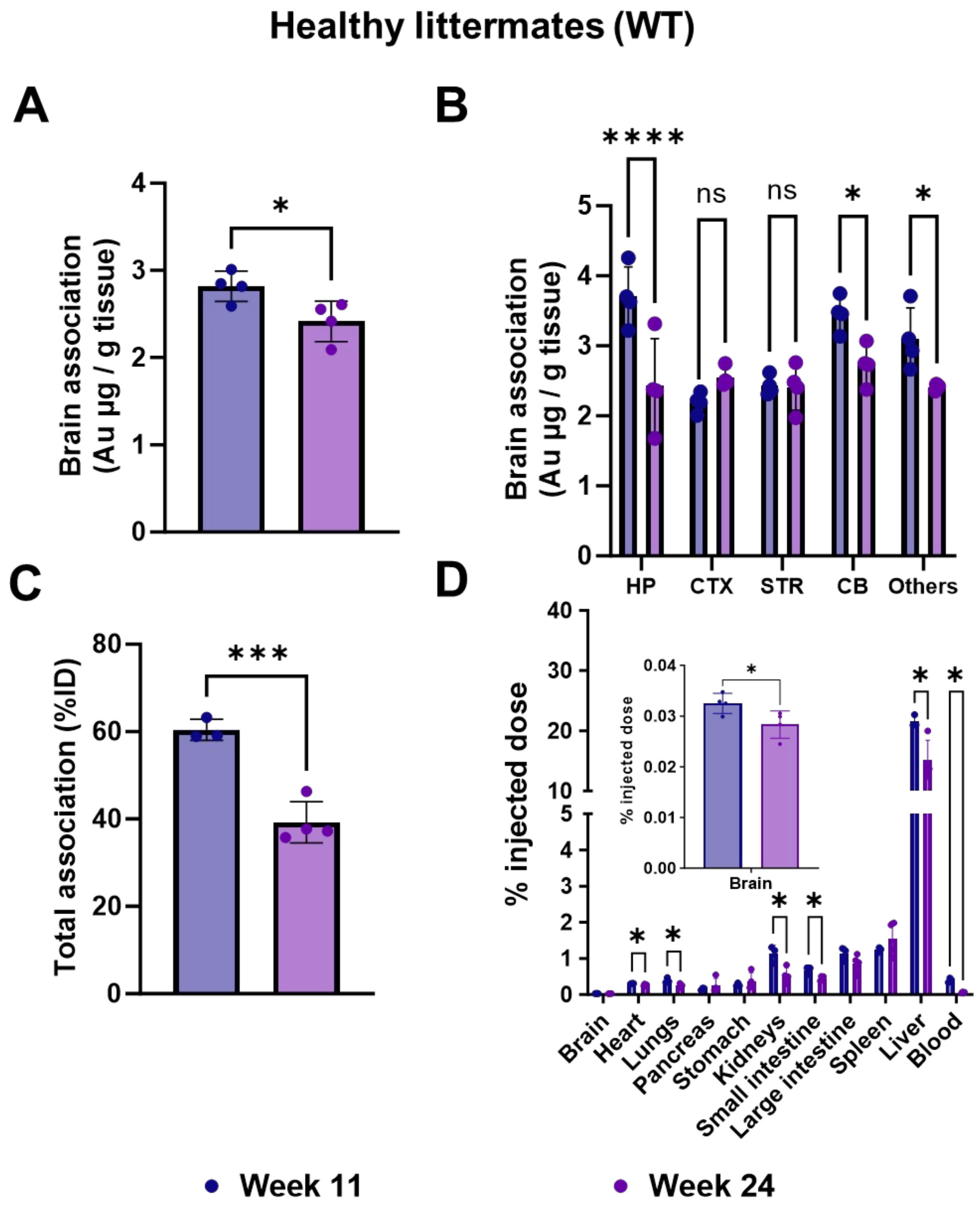


**Figure S66.** ICP-MS results showed that, when compared to treatment completion at the age of Week 11 (following weekly NP injections from Weeks 6 to 10; blue), the gold contents in (A), the whole brain and (B), some brain compartments [hippocampus (HP), cerebellum (CB), and other regions (Others)] of $Au_3@PEG_{1k}$ NP-treated healthy littermates (WT) dropped at the age of Week 24 (purple) in but not all [cortex (CTX) and striatum (STR)]. (C) The sum of all remaining gold contents in the bodies of WT mice on Week 24 was lower than that on Week 11. %ID = % injected dose. (D) For many organs, there was less gold content in WT and on Week 24 than Week 11. Data are from n = 3–4, across two experiments. Statistical significance was evaluated using unpaired Student's t-test. *$P < 0.05$; **$P < 0.01$; ***$P < 0.001$; ns = no significant difference ($P > 0.05$). All bars and error bars represent mean ± SD.

# Appendix IX: Proteomics

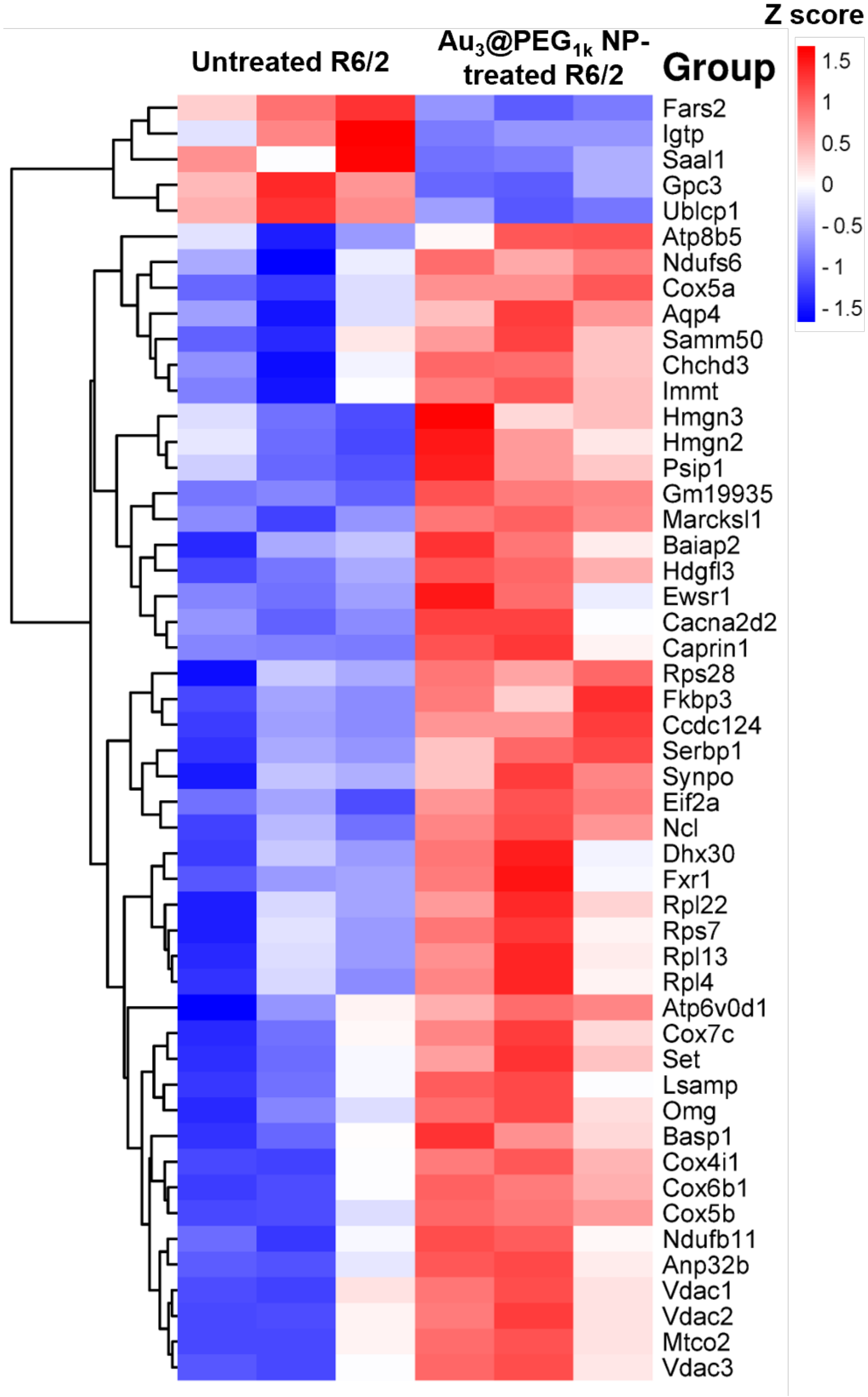


**Figure S67**. Heat map of 52 differentially expressed proteins (DEPs) in the HD brain of R6/2 mice based on the comparison of $Au_3$@$PEG_{1k}$ NP group to untreated group. Treatment timeline is shown in **Figure 2A**. The cut-off fold change (FC) was FC ≥ 1.5 or FC ≤ 1/1.5 with a *P* value < 0.05. Data are from n =3, across one experiment.

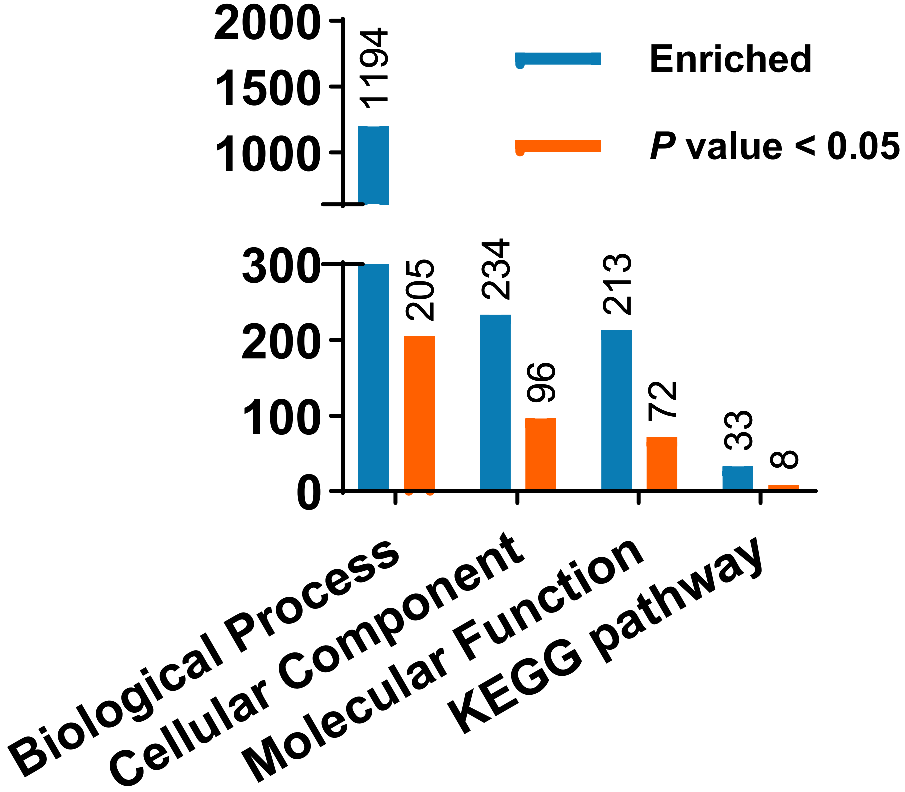


**Figure S68.** Number of enriched gene ontology (GO) terms and Kyoto Encyclopedia of Genes and Genomes (KEGG) pathways in the HD brain of R6/2 mice based on the comparison of $Au_3$@$PEG_{1k}$ NP group to untreated group. Treatment timeline is shown in **Figure 2A**. Enriched biological process, cellular components, molecular functions with fold changes > 1.5 or < 1/1.5) are shown in blue, and those with $P < 0.05$ are shown in orange. There were significant enrichments observed in 205 biological processes, 96 cellular components, 72 molecular functions, but only 8 KEGG pathways. Data are from n = 3, across one experiment.

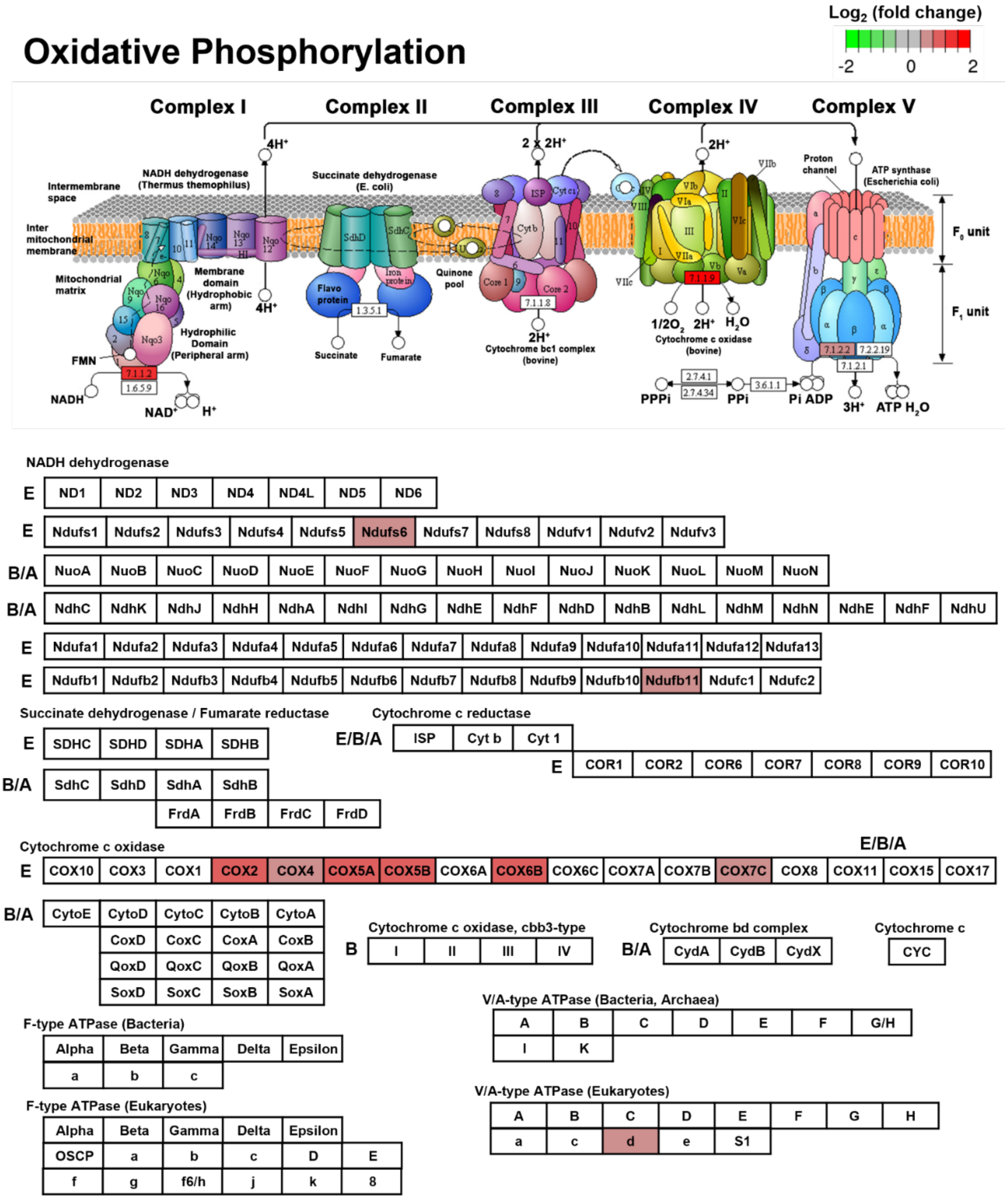


**Figure S69.** Map of the most significant enriched Kyoto Encyclopedia of Genes and Genomes (KEGG) pathway oxidative phosphorylation identified from DEPs obtained by comparing Au3@PEG1k NP-treated R6/2 to untreated R6/2 HD mice following the treatment schematic shown in **Figure 2A**. Data are from n =3, across one experiment.

**Table S6.** Enriched Kyoto Encyclopedia of Genes and Genomes (KEGG) pathway-related neurodegenerative diseases of the brain of $Au_3@PEG_{1k}$ NP-treated R6/2 mice following the treatment schematic shown in **Figure 2A**.

| **KEGG pathway** | ***P* value** | **Proteins** |
|---|---|---|
| Oxidative Phosphorylation | $8.1\times10^{-9}$ | **Cox5a, Cox5b, Cox6b1, Mtco2, Ndufb11, Cox4i1, Cox7c, Atp6v0d1, Ndufs6** |
| Parkinson disease | $2.7\times10^{-7}$ | **Cox5a, Cox5b, Cox6b1, Mtco2, Ndufb11, Cox4i1,** Vdac1, **Cox7c**, **Ndufs6** |
| Huntington disease | $1.1\times10^{-6}$ | **Cox5a, Cox5b, Cox6b1, Mtco2, Ndufb11, Cox4i1**, Vdac1, **Cox7c, Ndufs6** |
| Alzheimer disease | $5.7\times10^{-5}$ | **Cox5a, Cox5b, Cox6b1, Mtco2, Ndufb11, Cox4i1, Cox7c, Ndufs6** |

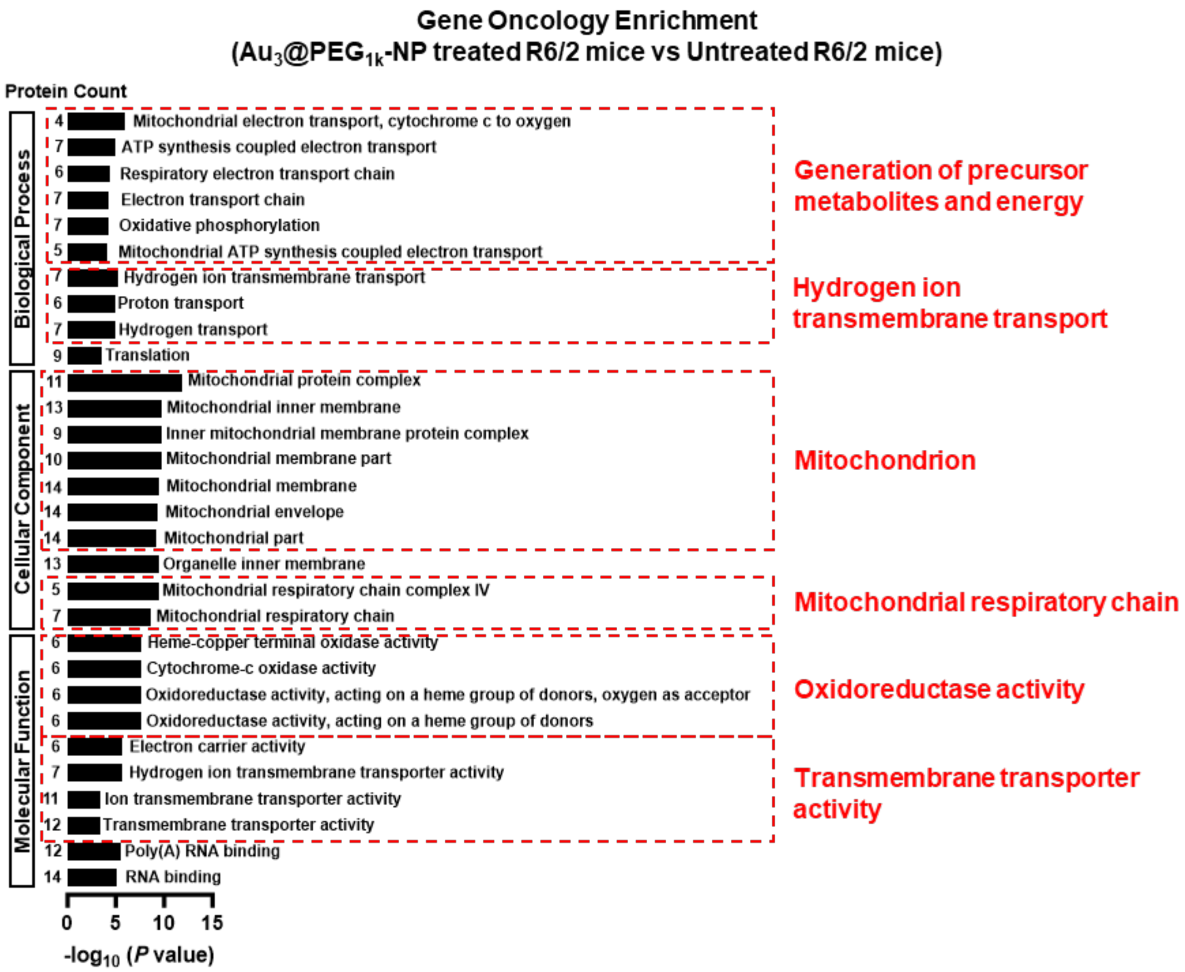


**Figure S70.** Top ten enriched GO terms identified from DEPs in the HD brain of R6/2 mice based on the comparison of $Au_3$@$PEG_{1k}$ NP group to untreated group. The treatment timeline is shown in **Figure 2A**. The three categories of GO terms are biological process, cellular component, and molecular function. The dashed box surrounds the more specific child terms of the less specific, higher-order GO term (red) per the ancestor chart in https://www.ebi.ac.uk/QuickGO/. Data are from n = 3, across one experiment.

**Table S7.** Relationship between the top ten GO terms in biological process and DEPs by comparing $Au_3@PEG_{1k}$ NP-treated to untreated R6/2 HD mice following the treatment in **Figure 2A**. The DEPs were determined by criteria of statistical significance (*P* < 0.05) with the cut-off fold change (FC) of FC ≥ 1.5 or FC ≤ 1/1.5. Data are from n =3, across one experiment.

| GO term | Proteins |
|---|---|
| Mitochondrial electron transport, cytochrome c to oxygen | Cox5a, Cox5b, Cox4i1, Cox7c |
| ATP synthesis coupled electron transport | Cox5a, Cox5b, Mtco2, Cox4i1, Cox7c, Ndufs6 |
| Respiratory electron transport chain | Cox5a, Cox5b, Mtco2, Cox4i1, Cox7c, Ndufs6 |
| Electron transport chain | Cox5a, Cox5b, Mtco2, Cox4i1, Cox7c, Ndufs6 |
| Oxidative phosphorylation | Cox5a, Cox5b, Mtco2, Cox4i1, Cox7c, Ndufs6 |
| Mitochondrial ATP synthesis coupled electron transport | Cox5a, Cox5b, Cox4i1, Cox7c, Ndufs6 |
| Hydrogen ion transmembrane transport | Cox5a, Cox5b, Mtco2, Cox4i1, Cox6b1, Cox7c, Atp6v0d1 |
| Proton transport | Cox5a, Cox5b, Mtco2, Cox4i1, Cox6b1, Cox7c, Atp6v0d1 |
| Hydrogen transport | Cox5a, Cox5b, Mtco2, Cox4i1, Cox6b1, Cox7c, Atp6v0d1 |
| Translation | Rpl4, Rps7, Rpl13, Rpl22, Rps28, Caprin1, Ncl, Fxr1, Eif2a |

**Table S8.** Relationship between the top ten GO terms in cellular component and DEPs by comparing $Au_3@PEG_{1k}$ NP-treated to untreated R6/2 HD mice following the treatment in **Figure 2A**. The DEPs were determined by criteria of statistical significance (*P* < 0.05) with the cut-off fold change (FC) of FC ≥ 1.5 or FC ≤ 1/1.5. Data are from n =3, across one experiment.

| GO term | Proteins |
|---|---|
| Mitochondrial protein complex | Cox5a, Cox5b, Mtco2, Cox4i1, Cox7c, Ndufs6, Ndufb11, Vdac1, Chchd3, Immt, Samm50 |
| Mitochondrial inner membrane | Cox5a, Cox5b, Mtco2, Cox4i1, Cox6b1, Cox7c, Ndufs6, Ndufb11, Vdac1, Vdac2, Chchd3, Immt, Samm50 |
| Inner mitochondrial membrane protein complex | Cox5a, Cox5b, Mtco2, Cox4i1, Cox7c, Ndufs6, Ndufb11, Chchd3, Immt |
| Mitochondrial membrane part | Cox5a, Cox5b, Mtco2, Cox4i1, Cox7c, Ndufs6, Ndufb11, Chchd3, Immt, Samm50 |
| Mitochondrial membrane | Cox5a, Cox5b, Mtco2, Cox4i1, Cox6b1, Cox7c, Ndufs6, Ndufb11, Vdac1, Vdac2, Vdac3, Chchd3, Immt, Samm50 |
| Mitochondrial envelope | Cox5a, Cox5b, Mtco2, Cox4i1, Cox6b1, Cox7c, Ndufs6, Ndufb11, Vdac1, Vdac2, Vdac3, Chchd3, Immt, Samm50 |
| Mitochondrial part | Cox5a, Cox5b, Mtco2, Cox4i1, Cox6b1, Cox7c, Ndufs6, Ndufb11, Vdac1, Vdac2, Vdac3, Chchd3, Immt, Samm50 |
| Organelle inner membrane | Cox5a, Cox5b, Mtco2, Cox4i1, Cox6b1, Cox7c, Ndufs6, Ndufb11, Vdac1, Vdac2, Chchd3, Immt, Samm50 |
| Mitochondrial respiratory chain complex IV | Cox5a, Cox5b, Mtco2, Cox4i1, Cox7c |
| Mitochondrial respiratory chain | Cox5a, Cox5b, Mtco2, Cox4i1, Cox7c, Ndufs6, Ndufb11 |

**Table S9.** Relationship between the top ten GO terms in molecular function and DEPs by comparing $Au_3$@$PEG_{1k}$ NP-treated to untreated R6/2 HD mice following the treatment in **Figure 2A**. The DEPs were determined by criteria of statistical significance ($P < 0.05$) with the cut-off fold change (FC) of FC ≥ 1.5 or FC ≤ 1/1.5. Data are from n =3, across one experiment.

| **GO term** | **Proteins** |
|---|---|
| Heme-copper terminal oxidase activity | Cox5a, Cox5b, Mtco2, Cox4i1, Cox6b1, Cox7c |
| Cytochrome-c oxidase activity | Cox5a, Cox5b, Mtco2, Cox4i1, Cox6b1, Cox7c |
| Oxidoreductase activity, acting on a heme group of donors, oxygen as acceptor | Cox5a, Cox5b, Mtco2, Cox4i1, Cox6b1, Cox7c |
| Oxidoreductase activity, acting on a heme group of donors | Cox5a, Cox5b, Mtco2, Cox4i1, Cox6b1, Cox7c |
| Electron carrier activity | Cox5a, Cox5b, Mtco2, Cox4i1, Cox6b1, Cox7c |
| Hydrogen ion transmembrane transporter activity | Cox5a, Cox5b, Mtco2, Cox4i1, Cox6b1, Cox7c, Atp6v0d1 |
| Ion transmembrane transporter activity | Cox5a, Cox5b, Mtco2, Cox4i1, Cox6b1, Cox7c, Atp6v0d1, Vdac1, Vdac2, Vdac3, Cacna2d2 |
| Transmembrane transporter activity | Cox5a, Cox5b, Mtco2, Cox4i1, Cox6b1, Cox7c, Atp6v0d1, Vdac1, Vdac2, Vdac3, Cacna2d2, Aqp4 |
| Poly(A) RNA binding | Rpl4, Rps7, Rpl13, Rpl22, Rps28, Immt, Caprin1, Serbp1, Ccdc124, Fxr1, Ncl, Psip1 |
| RNA binding | Rpl4, Rps7, Rpl13, Rpl22, Rps28, Immt, Caprin1, Serbp1, Ccdc124, Fxr1, Ncl, Psip1, Ewsr1, Eif2a |

# Appendix X: Kinome Profiling and Validation

**Table S10.** List of kinases with >85% inhibition in their activities after treatment with ~200 nM of $Au_3@PEG_{1k}$ NP (with corresponding kinase comparing against with ~200 nM of 70 kDa dextran) using the Z'-LYTE™ Kinase Assay Kit (Thermo Fisher Scientific).[a] Cumulative publication count till August 2024 based on defined keyword searches between "Huntington's disease" and the corresponding row of kinase in PubMed.

| **Kinase** | **[ATP] tested (μM)** | **Mean Percent Inhibition (%)** | | **HD-related publication count[a]** |
|---|---|---|---|---|
| | | $Au_3@PEG_{1k}$ NP | 70 kDa Dextran NP | |
| MAPK14 (p38α) | Cascade: 100 | 100 | 50 | 54 |
| PDK1 Direct | Km app: 27 | 85 | 1 | 7 |
| MAP2K6 (MKK6) | Cascade: 100 | 100 | 3 | 2 |
| PLK2 | Km app: 29.6 | 99 | 10 | 2 |
| CAMK4 (CaMKIV) | Km app: 18 | 91 | 9 | 2 |
| GRK4 | Km app: 12 | 92 | 64 | 1 |
| NEK1 | Km app: 118.7 | 86 | 9 | 1 |
| FGR | Km app: 10 | 90 | 47 | 0 |
| PLK3 | Km app: 47.8 | 87 | 50 | 0 |
| MELK | Km app: 30 | 100 | 53 | 0 |
| SRMS (Srm) | Km app: 126.9 | 89 | 15 | 0 |
| RPS6KA6 (RSK4)_1hr | Km app: 30 | 99 | 31 | 0 |

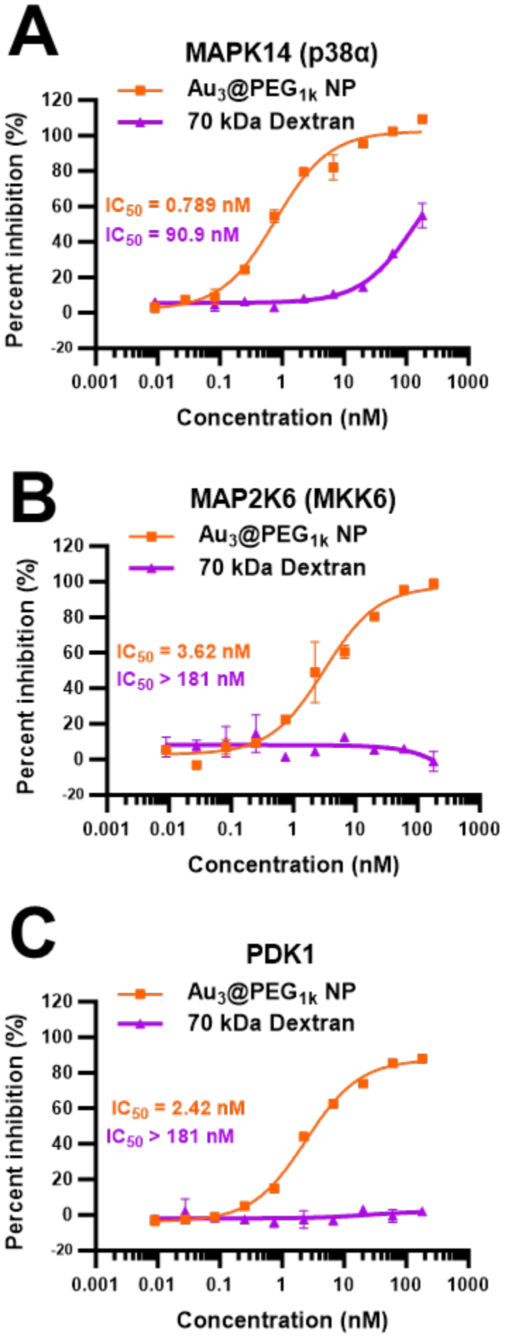


**Figure S71.** 10-point titration curves and $IC_{50}$ values of $Au_3@PEG_{1k}$ or 70 kDa dextran NP on (A) MAPK14 (p38α), (B) MAP2K6 (MKK6; upstream of p38α), and (C) PDK1 respectively. 70 kDa dextran is a "size-matched" negative control of $Au_3@PEG_{1k}$ NP; both are ~11 nm in diameter.

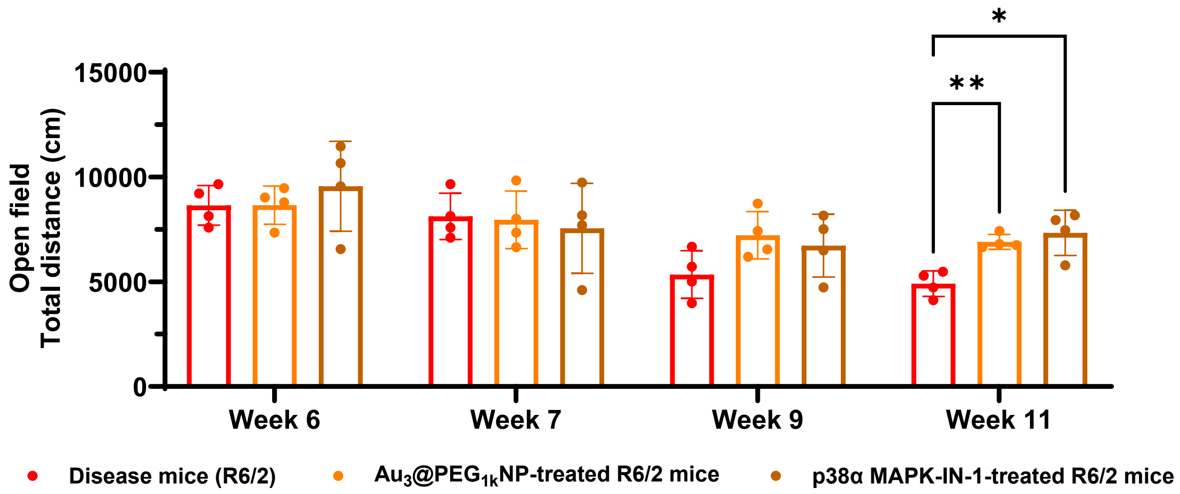


**Figure S72.** Open field test of untreated (red), $Au_3$@$PEG_{1k}$ NP-treated (orange), and p38α MAPK-IN-1 (an inhibitor of p38α)-treated (brown) R6/2 mice as a function of age, based on the treatment plan in **Figure 2A**. At Week 11, both $Au_3$@$PEG_{1k}$ NP- and p38α MAPK-IN-1 treatments improved the total distance travelled in 30 min than untreated. Data are from n = 4, across five experiments. Statistical significance was evaluated using Two-Way ANOVA with Tukey's post hoc test for multiple comparisons. *$P < 0.05$, **$P < 0.01$. All bars and error bars represent mean ± SD.

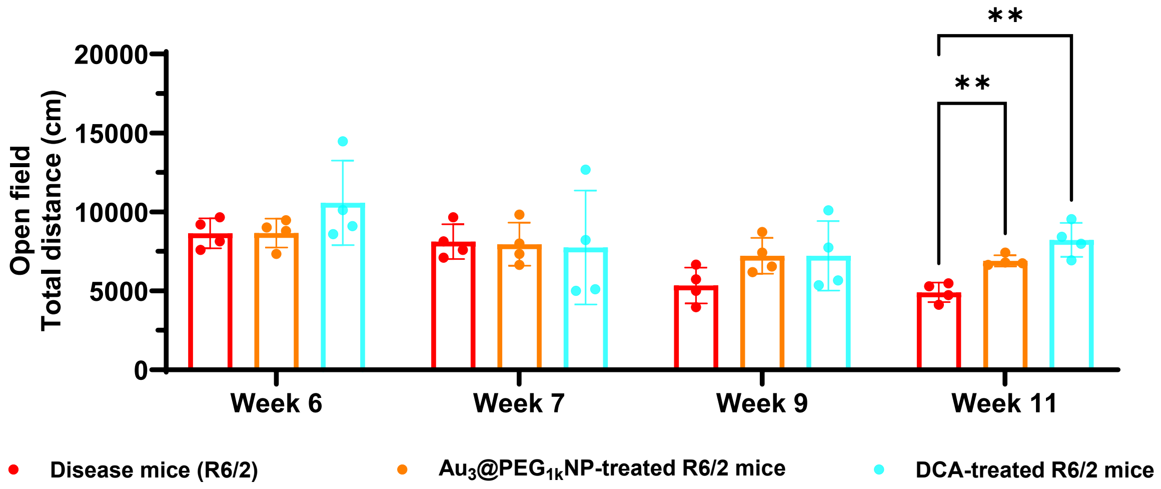


**Figure S73.** Open field test of untreated (red), $Au_3$@$PEG_{1k}$ NP-treated (orange), and dichloroacetate (DCA; an inhibitor of PDK1)-treated (cyan) R6/2 mice as a function of age, based on the treatment plan in **Figure 2A**. At Week 11, both $Au_3$@$PEG_{1k}$ NP- and DCA treatments improved the total distance travelled in 30 min than untreated. Data are from n = 4, across five experiments. Statistical significance was evaluated using Two-Way ANOVA with Tukey's post hoc test for multiple comparisons. **$P < 0.01$. All bars and error bars represent mean ± SD.

# Appendix XI: Neuroinflammation

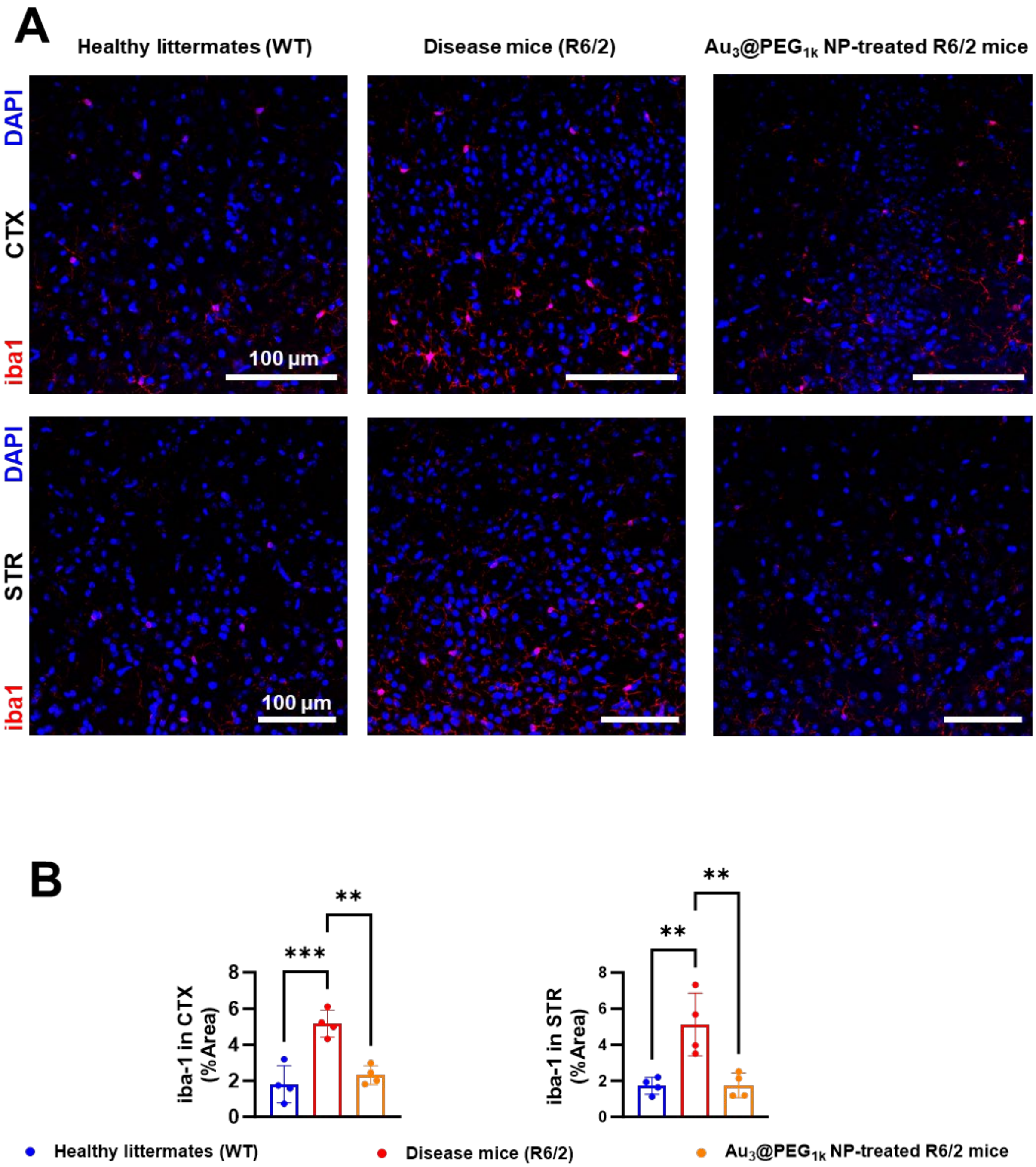


**Figure S74.** $Au_3@PEG_{1k}$ NP reduced neuroinflammation in the cortex (CTX) and striatum (STR) of R6/2 mice. (A) Confocal images of iba1-stained (left; red) CTX and STR cryosections of $Au_3@PEG_{1k}$ NP-treated R6/2 mice area. Blue: nucleus (DAPI). (B) Quantification of the iba1-positive area in (A). Data are from n = 4, across two experiments. Statistical significance was evaluated using One-Way ANOVA with Tukey's post hoc test for multiple comparisons. **P* < 0.05; ***P* < 0.01; ****P* < 0.001. All bars and error bars represent mean ± SD.

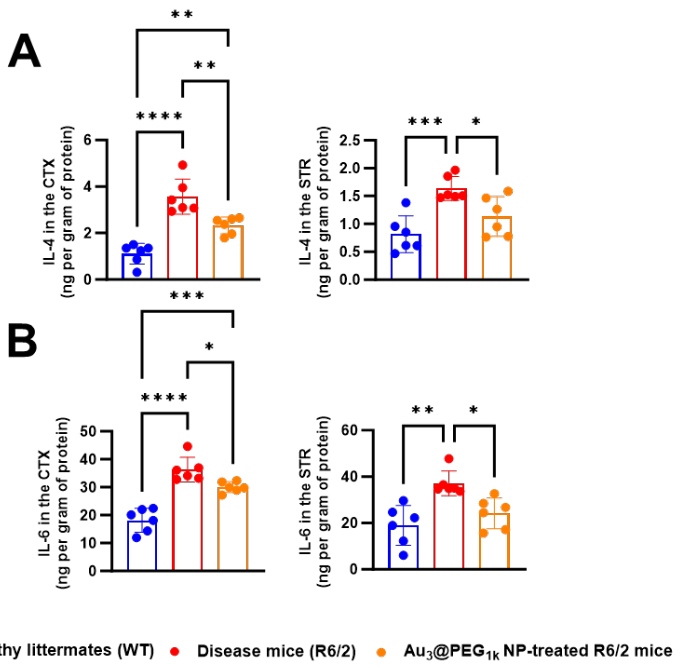


**Figure S75.** ELISA analysis of (A) IL-4 and (B) IL-6 revealed inhibition of HD-related cytokines upon $Au_3$@$PEG_{1k}$ NP treatment of R6/2 mice (orange) when compared to untreated R6/2 mice (red) in CTX and STR. Data are from n = 6, across one experiment. All statistical significance was evaluated using One-Way ANOVA with Tukey's post hoc test for multiple comparisons. $^{*}P < 0.05$; $^{**}P < 0.01$; $^{***}P < 0.001$, $^{****}P < 0.0001$. All bars and error bars represent mean ± SD.

# Appendix XII: Others

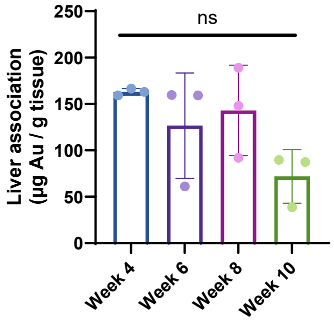


**Figure S76.** Liver accumulation of $Au_3$@$PEG_{1k}$ NP upon i.v. injection into Week 4 (blue), 6 (purple), 8 (pink) and 10 (green) R6/2 mice 24 h post-injection. The bulk gold content in each organ was detected using ICP-MS and expressed in terms of gold mass per tissue mass. Data are from n = 3, across 6 experiments. Statistical significance was evaluated using One-Way ANOVA with Tukey's post hoc test for multiple comparisons. ns = no significant difference. All bars and error bars represent mean ± SD. These absolute values are high when compared to previous work, possibly because of our high gold NP dose to mitigate liver clearance in terms of %ID and achieve therapeutic efficacy; this NP dose is similar to that of chiral gold NP for treating Alzheimer's disease.

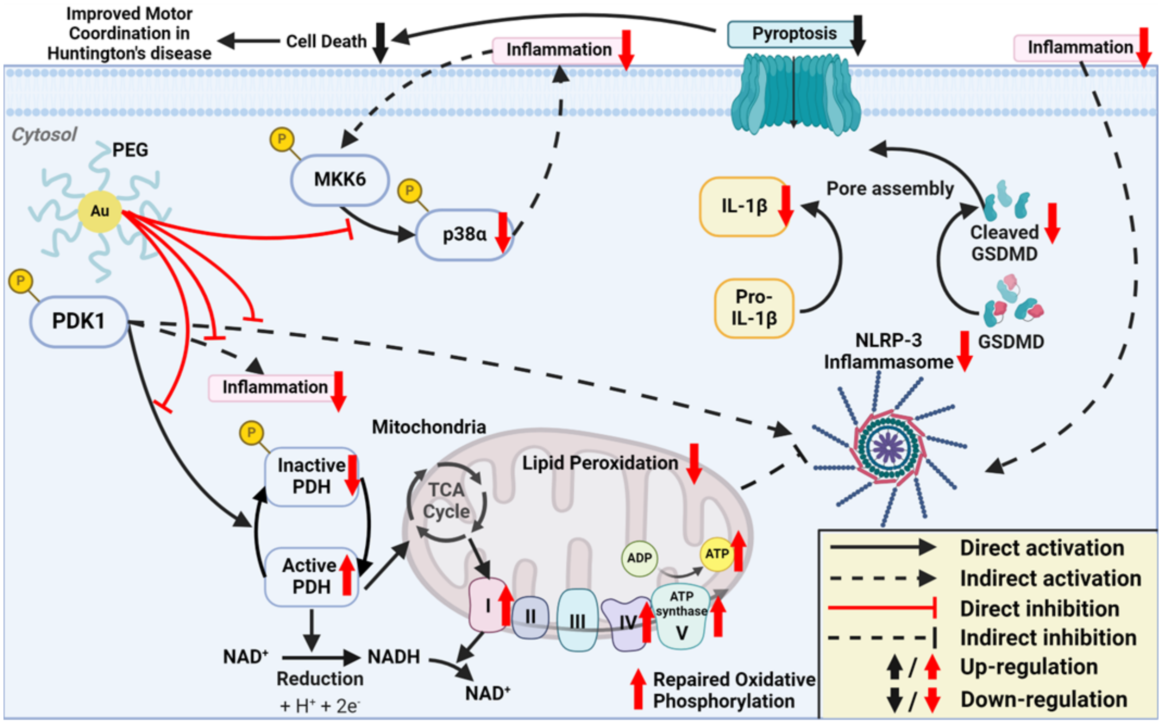


**Figure S77.** Proposed therapeutic mechanism of $Au_3@PEG_{1k}$ NPs in HD mice. Schematic illustration of proposed therapeutic mechanisms of $Au_3@PEG_{1k}$ NP to HD followed by weekly i.v. injections of $Au_3@PEG_{1k}$ NPs into R6/2 mice. Red arrows indicate the up/down-regulated protein/pathway state or action of $Au_3@PEG_{1k}$ NPs as suggested in **Figure 4–7**; black arrows indicate the up/down-regulated protein/pathway state or relationship between proteins/pathways suggested in the literature. In HD, it has been demonstrated in the literature that the transcription of m*HTT* gene gives rise to the phosphorylation of PDK1 which phosphorylates PDH to its inactive form[44,45]. As a result, the cells of HD will undergo anaerobic respiration instead of proceeding to oxidative phosphorylation and this will lead to reduced ATP production[44]. Furthermore, mitochondrial dysfunction will further lead to oxidative stress and NLRP-3 inflammasome-mediated neuroinflammation via activation of caspase 1. The release of IL-1β will further elicit inflammation and the cleavage of GSDMD by activated caspase 1 will further form pores on cell death, causing pyroptosis-mediated cell death[46]. In addition, the upregulation of MAPK signalling pathways [*i.e.*, phosphorylation of mitogen-activated protein kinase kinase 6 (MKK6), p38α and Mitogen-activated protein kinase-activated protein kinase 2 (MK2)] will further trigger neuroinflammation[47]. To revert pyroptosis-mediated cell death, $Au_3@PEG_{1k}$ NP can upregulate oxidative phosphorylation and protect mitochondria from triggering pyroptosis by inhibition of PDK1. $Au_3@PEG_{1k}$ NP can also inhibit MAPK signalling pathways and the associated neuroinflammation. The figure was created using BioRender.

**SI References**


(1) Piella, J.; Bastús, N. G.; Puntes, V. Size-Controlled Synthesis of Sub-10-Nanometer Citrate-Stabilized Gold Nanoparticles and Related Optical Properties. *Chem. Mater.* 2016, *28*, 1066–1075.

(2) Frens, G. Controlled Nucleation for the Regulation of the Particle Size in Monodisperse Gold Suspensions. *Nat. Phys. Sci.* 1973, *241*, 20–22.

(3) Sawada, T.; Fukuta, H.; Serizawa, T. Preparation of Biocomposite Soft Nanoparticles Composed of Poly(Propylene Oxide) and the Polymer-Binding Peptides. *Processes* 2020, *8*, 859.

(4) Cui, C.; Bao, Z.; Chow, S.; Wang, Q.; Chai, S.; Xu, Z.; Jiang, Q.; Cheung, W. An Optimized Ex Vivo Protocol for Quantitative Electrophysiological Assessment of Neuromuscular Junctions and Skeletal Muscle Function Using the Aurora System. *Bio. Protoc.* 2025, *15,* e5353.

(5) Ho, L. W. C.; Yin, B.; Dai, G.; Choi, C. H. J. Effect of Surface Modification with Hydrocarbyl Groups on the Exocytosis of Nanoparticles. *Biochem.* 2021, *60*, 1019–1030.

(6) Benoit, D. N.; Zhu, H.; Lilierose, M. H.; Verm, R. A.; Ali, N.; Morrison, A. N.; Fortner, J. D.; Avendano, C.; Colvin, V. L. Measuring the Grafting Density of Nanoparticles in Solution by Analytical Ultracentrifugation and Total Organic Carbon Analysis. *Anal. Chem.* 2012, *84*, 9238–9245.

(7) Peng, S.; Guo, P.; Lin, X.; An, Y.; Sze, K. H.; Lau, M. H. Y.; Chen, Z. S.; Wang, Q.; Li, W.; Sun, J. K.-L.; Ma, S. Y.; Chan, T.-F.; Lau, K.-F.; Ngo, J. C. K.; Kwan, K. M.; Wong, C.-H.; Lam, S. L.; Zimmerman, S. C.; Tuccinardi, T.; Zuo, Z.; Au-Yeung, H. Y.; Chow, H.-M.; Chan, H. Y. E. CAG RNAs Induce DNA Damage and Apoptosis by Silencing NUDT16 Expression in Polyglutamine Degeneration. *Proc. Natl. Acad. Sci. U. S. A.* 2021, *118*, e2022940118.

(8) Kumar, P.; Nagarajan, A.; Uchil, P. D. Analysis of Cell Viability by the Lactate Dehydrogenase Assay. *Cold Spring Harb. Protoc.* 2018, *2018*, 465–468.

(9) Kim, C. S.; Li, X.; Jiang, Y.; Yan, B.; Tonga, G. Y.; Ray, M.; Solfiell, D. J.; Rotello, V. M. Cellular Imaging of Endosome Entrapped Small Gold Nanoparticles. *MethodsX* 2015, *2*, 306–315.

(10) Tylawsky, D. E.; Kiguchi, H.; Vaynshteyn, J.; Gerwin, J.; Shah, J.; Islam, T.; Boyer, J. A.; Boué, D. R.; Snuderl, M.; Greenblatt, M. B.; Shamay, Y.; Raju, G. P.; Heller, D. A. P-Selectin-Targeted Nanocarriers Induce Active Crossing of the Blood–Brain Barrier via Caveolin-1-Dependent Transcytosis. *Nat. Mater.* 2023, *22*, 391–399.

(11) Zha, S.; Liu, H.; Li, H.; Li, H.; Wong, K.-L.; All, A. H. Functionalized Nanomaterials Capable of Crossing the Blood–Brain Barrier. *ACS Nano* 2024, *18*, 1820–1845.

(12) Yang, S.; Mei, S.; Jin, H.; Zhu, B.; Tian, Y.; Huo, J.; Cui, X.; Guo, A.; Zhao, Z. Identification of Two Immortalized Cell Lines, ECV304 and BEnd3, for in Vitro Permeability Studies of Blood-Brain Barrier. *PLoS One* 2017, *12*, e0187017.

(13) Liu, Y.; Choi, C. K. K.; Hong, H.; Xiao, Y.; Kwok, M. L.; Liu, H.; Tian, X. Y.; Choi, C. H. J. Dopamine Receptor-Mediated Binding and Cellular Uptake of Polydopamine-Coated Nanoparticles. *ACS Nano* 2021, *15*, 13871–13890.

(14) Ho, L. W. C.; Yung, W. Y.; Sy, K. H. S.; Li, H. Y.; Choi, C. K. K.; Leung, K. C. F.; Lee, T. W. Y.; Choi, C. H. J. Effect of Alkylation on the Cellular Uptake of Polyethylene Glycol-Coated Gold Nanoparticles. *ACS Nano* 2017, *11*, 6085–6101.

(15) Gálvez, B. G.; Matías-Román, S.; Yáñez-Mó, M.; Vicente-Manzanares, M.; Sánchez-Madrid, F.; Arroyo, A. G. Caveolae Are a Novel Pathway for Membrane-Type 1 Matrix Metalloproteinase Traffic in Human Endothelial Cells. *Mol. Biol. Cell.* 2004, *15*, 678–687.
(16) Zhang, W.; Refaat, A.; Li, H.; Zhu, D.; Tong, Z.; Nicolazzo, J. A.; Peng, B.; Bai, H.; Esser, L.; Voelcker, N. H. Optimizing Angiopep-2 Density on Polymeric Nanoparticles for Enhanced Blood–Brain Barrier Penetration and Glioblastoma Targeting: Insights from *in Vitro* and *in Vivo* Experiments. *Adv. Funct. Mater.* 2025, *35*, 2425165.
(17) Xiao, R.-R.; Jing, B.; Yan, L.; Li, J.; Tu, P.; Ai, X. Constant-Rate Perfused Array Chip for High-Throughput Screening of Drug Permeability through Brain Endothelium. *Lab Chip.* 2022, *22*, 4481–4492.
(18) Spijker, S. Dissection of Rodent Brain Regions. In *Neuroproteomics*; Li, K. W., Ed.; Humana Press: Totowa, NJ, 2011; pp 13–26.
(19) Potts, E. M.; Coppotelli, G.; Ross, J. M. Histological-Based Stainings Using Free-Floating Tissue Sections. *J. Vis. Exp.* 2020, *162*, e61622.
(20) Lee, L. K. C.; Leong, L. I.; Liu, Y.; Luo, M.; Chan, H. Y. E.; Choi, C. H. J. Preclinical Nanomedicines for Polyglutamine-Based Neurodegenerative Diseases. *Mol. Pharmaceutics.* 2021, *18*, 610–626.
(21) Hirunagi, T.; Sahashi, K.; Tachikawa, K.; Leu, A. I.; Nguyen, M.; Mukthavaram, R.; Karmali, P. P.; Chivukula, P.; Tohnai, G.; Iida, M.; Onodera, K.; Ohyama, M.; Okada, Y.; Okano, H.; Katsuno, M. Selective Suppression of Polyglutamine-Expanded Protein by Lipid Nanoparticle-Delivered SiRNA Targeting CAG Expansions in the Mouse CNS. *Mol. Ther. Nucleic Acids* 2021, *24*, 1–10.
(22) Dar, G. H.; Mendes, C. C.; Kuan, W. L.; Speciale, A. A.; Conceição, M.; Görgens, A.; Uliyakina, I.; Lobo, M. J.; Lim, W. F.; EL Andaloussi, S.; Mäger, I.; Roberts, T. C.; Barker, R. A.; Goberdhan, D. C. I.; Wilson, C.; Wood, M. J. A. GAPDH Controls Extracellular Vesicle Biogenesis and Enhances the Therapeutic Potential of EV Mediated SiRNA Delivery to the Brain. *Nat. Commun.* 2021, *12*, 6666.
(23) Caron, N. S.; Aly, A. E.-E.; Findlay Black, H.; Martin, D. D. O.; Schmidt, M. E.; Ko, S.; Anderson, C.; Harvey, E. M.; Casal, L. L.; Anderson, L. M.; Rahavi, S. M. R.; Reid, G. S. D.; Oda, M. N.; Stanimirovic, D.; Abulrob, A.; McBride, J. L.; Leavitt, B. R.; Hayden, M. R. Systemic Delivery of Mutant Huntingtin Lowering Antisense Oligonucleotides to the Brain Using Apolipoprotein A-I Nanodisks for Huntington Disease. *J. Control. Release.* 2024, *367*, 27–44.
(24) Wahyuningtyas, D.; Chen, W. H.; He, R. Y.; Huang, Y. A.; Tsao, C. K.; He, Y. J.; Yu, C. Y.; Lu, P. C.; Chen, Y. C.; Wang, S. H.; Ng, K. C.; Po-Wen Chen, B.; Wei, P. K.; Shie, J. J.; Kuo, C. H.; Sun, Y. H.; Jen-Tse Huang, J. Polyglutamine-Specific Gold Nanoparticle Complex Alleviates Mutant Huntingtin-Induced Toxicity. *ACS Appl. Mater. Interfaces.* 2021, *13*, 60894–60906.
(25) Saad, M. A.; Ahmed, M. A. E.; Elbadawy, N. N.; Abdelkader, N. F. Nano-Ivabradine Averts Behavioral Anomalies in Huntington's Disease Rat Model via Modulating Rhes/m-Tor Pathway. *Prog. Neuropsychopharmacol. Biol. Psychiatry* 2021, *111*, 110368.
(26) Cano, A.; Ettcheto, M.; Espina, M.; Auladell, C.; Folch, J.; Kühne, B. A.; Barenys, M.; Sánchez-López, E.; Souto, E. B.; García, M. L.; Turowski, P.; Camins, A. Epigallocatechin-3-Gallate PEGylated Poly(Lactic-Co-Glycolic) Acid Nanoparticles Mitigate Striatal Pathology and Motor Deficits in 3-Nitropropionic Acid Intoxicated Mice. *Nanomedicine(Lond)* 2021, *16*, 19–35.

(27) Nagdiya, D.; Arora, S.; Kumar, V.; Kumar, D.; Singh, A.; Singh, C. Application of Casein Micelles for Targeting Huntington's Disease in Experimental Zebrafish Model. *Mol. Neurobiol.* 2024.

(28) Bolshakova, O. I.; Borisenkova, A. A.; Golomidov, I. M.; Komissarov, A. E.; Slobodina, A. D.; Ryabova, E. V.; Ryabokon, I. S.; Latypova, E. M.; Slepneva, E. E.; Sarantseva, S. V. Fullerenols Prevent Neuron Death and Reduce Oxidative Stress in Drosophila Huntington's Disease Model. *Cells* 2023, *12*, 170.

(29) Gonchar, O. O.; Maznychenko, A. V.; Klyuchko, O. M.; Mankovska, I. M.; Butowska, K.; Borowik, A.; Piosik, J.; Sokolowska, I. C60 Fullerene Reduces 3-Nitropropionic Acid-Induced Oxidative Stress Disorders and Mitochondrial Dysfunction in Rats by Modulation of P53, Bcl-2 and Nrf2 Targeted Proteins. *Int. J. Mol. Sci.* 2021, *22*, 5444.

(30) Adhikari, A.; Mondal, S.; Das, M.; Biswas, P.; Pal, U.; Darbar, S.; Bhattacharya, S. S.; Pal, D.; Saha-Dasgupta, T.; Das, A. K.; Mallick, A. K.; Pal, S. K. Incorporation of a Biocompatible Nanozyme in Cellular Antioxidant Enzyme Cascade Reverses Huntington's like Disorder in Preclinical Model. *Adv. Healthc. Mater.* 2021, *10*, 2001736.

(31) Li, M.; Yasumura, D.; Ma, A. A. K.; Matthes, M. T.; Yang, H.; Nielson, G.; Huang, Y.; Szoka, F. C.; LaVail, M. M.; Diamond, M. I. Intravitreal Administration of HA-1077, a ROCK Inhibitor, Improves Retinal Function in a Mouse Model of Huntington Disease. *PLoS One* 2013, *8*, e56026.

(32) Tramontin, N. dos S.; da Silva, S.; Arruda, R.; Ugioni, K. S.; Canteiro, P. B.; Silveira, G. de B.; Mendes, C.; Silveira, P. C. L.; Muller, A. P. Gold Nanoparticles Treatment Reverses Brain Damage in Alzheimer's Disease Model. *Mol. Neurobiol.* 2020, *57*, 926–936.

(33) Muller, A. P.; Ferreira, G. K.; Pires, A. J.; de Bem Silveira, G.; de Souza, D. L.; Brandolfi, J. de A.; de Souza, C. T.; Paula, M. M. S.; Silveira, P. C. L. Gold Nanoparticles Prevent Cognitive Deficits, Oxidative Stress and Inflammation in a Rat Model of Sporadic Dementia of Alzheimer's Type. *Mater. Sci. Eng. C. Mater. Biol. Appl.* 2017, *77*, 476–483.

(34) Hou, K.; Zhao, J.; Wang, H.; Li, B.; Li, K.; Shi, X.; Wan, K.; Ai, J.; Lv, J.; Wang, D.; Huang, Q.; Wang, H.; Cao, Q.; Liu, S.; Tang, Z. Chiral Gold Nanoparticles Enantioselectively Rescue Memory Deficits in a Mouse Model of Alzheimer's Disease. *Nat. Commun.* 2020, *11*, 4790.

(35) Resmi, A. N.; Rekha, C. R.; Dhushyandhun, M. E.; Elangovan, S.; Shenoy, S. J.; Gulia, K. K.; Jayasree, R. S. Bifunctional Cysteine Gold Nanoclusters for β-Amyloid Fibril Inhibition and Fluorescence Imaging: A Distinctive Approach to Manage Alzheimer's Disease. *J. Mater. Chem. B* 2023, *11*, 4715–4724.

(36) Guo, X.; Li, C.; Zhang, J.; Sun, M.; Xu, J.; Xu, C.; Kuang, H.; Xu, L. Chiral Nanoparticle-Remodeled Gut Microbiota Alleviates Neurodegeneration via the Gut–Brain Axis. *Nat. Aging* 2023, *3* (11), 1415–1429.

(37) Sanati, M.; Khodagholi, F.; Aminyavari, S.; Ghasemi, F.; Gholami, M.; Kebriaeezadeh, A.; Sabzevari, O.; Hajipour, M. J.; Imani, M.; Mahmoudi, M.; Sharifzadeh, M. Impact of Gold Nanoparticles on Amyloid β-Induced Alzheimer's Disease in a Rat Animal Model: Involvement of STIM Proteins. *ACS Chem. Neurosci.* 2019, *10*, 2299–2309.

(38) da Silva Córneo, E.; de Bem Silveira, G.; Scussel, R.; Correa, M. E. A. B.; da Silva Abel, J.; Luiz, G. P.; Feuser, P. E.; Silveira, P. C. L.; Machado-de-Ávila, R. A. Effects of Gold Nanoparticles Administration through Behavioral and Oxidative Parameters in Animal Model of Parkinson's Disease. *Colloids Surf. B: Biointerfaces.* 2020, *196*, 111302.

(39) Gao, G.; Chen, R.; He, M.; Li, J.; Wang, L.; Sun, T. Gold Nanoclusters for Parkinson's Disease Treatment. *Biomaterials* 2019, *194*, 36–46.
(40) Xue, J.; Liu, T.; Liu, Y.; Jiang, Y.; Seshadri, V. D. D.; Mohan, S. K.; Ling, L. Neuroprotective Effect of Biosynthesised Gold Nanoparticles Synthesised from Root Extract of Paeonia Moutan against Parkinson Disease – *In Vitro* & *In Vivo* Model. *J. of Photoch. and Photob. B: Bio.* 2019, *200*, 111635.
(41) Lee, S.; Shim, H. S.; Park, H. J.; Chang, Y.; Han, Y. eun; Oh, S. J.; Lee, W.; Im, H.; Seol, Y. H.; Ryu, H.; Kang, H.; Lee, Y. K.; Park, S.; Yoo, J. Elongated Nanoporous Au Networks Improve Somatic Cell Direct Conversion into Induced Dopaminergic Neurons for Parkinson's Disease Therapy. *Acta Biomater.* 2022, *151*, 561–575.
(42) Wang, Z.; Henriques, A.; Rouvière, L.; Callizot, N.; Tan, L.; Hotchkin, M. T.; Rossignol, R.; Mortenson, M. G.; Dorfman, A. R.; Ho, K. S.; Wang, H. A Mechanism Underpinning the Bioenergetic Metabolism-Regulating Function of Gold Nanocatalysts. *Small* 2024, *20*, 2304082.
(43) Odell, M. Electric Shocks and Electrocution, Clinical Effects and Pathology. In *Encyclopedia of Forensic and Legal Medicine: Second Edition*; Elsevier, 2016; pp 419–427.
(44) Vallée, A.; Lecarpentier, Y.; Guillevin, R.; Vallée, J.-N. Aerobic Glycolysis in Amyotrophic Lateral Sclerosis and Huntington's Disease. *Rev. Neurosci.* 2018, *29*, 547–555.
(45) Levina, A.; Fleming, K. D.; Burke, J. E.; Leonard, T. A. Activation of the Essential Kinase PDK1 by Phosphoinositide-Driven Trans-Autophosphorylation. *Nat. Commun.* 2022, *13*, 1874.
(46) Meyers, A. K.; Wang, Z.; Han, W.; Zhao, Q.; Zabalawi, M.; Duan, L.; Liu, J.; Zhang, Q.; Manne, R. K.; Lorenzo, F.; Quinn, M. A.; Song, Q.; Fan, D.; Lin, H.-K.; Furdui, C. M.; Locasale, J. W.; McCall, C. E.; Zhu, X. Pyruvate Dehydrogenase Kinase Supports Macrophage NLRP3 Inflammasome Activation during Acute Inflammation. *Cell Rep.* 2023, *42*, 111941.
(47) D'Mello, S. R. When Good Kinases Go Rogue: GSK3, P38 MAPK and CDKs as Therapeutic Targets for Alzheimer's and Huntington's Disease. *Int. J. Mol. Sci.* 2021, *22*, 5911.

\
